# Stereoselective Synthesis and Application of Gibberellic Acid-Derived Aminodiols

**DOI:** 10.3390/ijms231810366

**Published:** 2022-09-08

**Authors:** Zein Alabdeen Khdar, Tam Minh Le, Zsuzsanna Schelz, István Zupkó, Zsolt Szakonyi

**Affiliations:** 1 Institute of Pharmaceutical Chemistry, University of Szeged, Interdisciplinary Excellent Center, Eötvös utca 6, H-6720 Szeged, Hungary; 2 Stereochemistry Research Group of the Hungarian Academy of Sciences, Eötvös utca 6, H-6720 Szeged, Hungary; 3 Institute of Pharmacodynamics and Biopharmacy, University of Szeged, Interdisciplinary Excellent Center, H-6720 Szeged, Hungary

**Keywords:** gibberellic acid, allo-gibberic acid, 3-amino-1,2-diols, antiproliferative activity, diterpene

## Abstract

A series of gibberellic acid-based aminodiols was designed and synthesized from commercially available gibberellic acid. Exposure of gibberellic acid to hydrochloric acid under reflux conditions resulted in aromatization followed by rearrangement to form allo-gibberic acid. The key intermediate, ethyl allo-gibberate, was prepared according to literature methods. Epoxidation of key intermediate and subsequent ring-opening of the corresponding epoxide with different nucleophiles resulted in *N*-substituted aminodiols. The regioselective ring closure of *N*-benzyl-substituted aminodiol with formaldehyde was also investigated. All aminodiol derivatives were well characterized using modern spectroscopic techniques and evaluated for their antiproliferative activity against a panel of human cancer cell lines. In addition, structure–activity relationships were examined by assessing substituent effects on the aminodiol systems. The results indicated that aminodiols containing aromatic rings on their nitrogen substituents displayed significant cytotoxic effects. Among these agents, *N*-naphthylmethyl-substituted aminodiols were found to be the most potent candidates in this series. One of these molecules exhibited a modest cancer selectivity determined by non-cancerous fibroblast cells. A docking study was also made to exploit the observed results.

## 1. Introduction

Cancer, characterized by uncontrolled growth and spread of abnormal cells, is the most feared disease second only to heart disease as a leading cause of death all over the world [1]. Therefore, the development of anticancer agents is the major focus for scientists across the world. Over the past few decades, extensive research has led to the development of a plethora of chemotherapeutic agents [2,3]. However, the limitations of current anticancer drugs, increased incidence, and rapid development of drug resistance have highlighted the need for the discovery of new anticancer agents [4], preferably with novel mechanisms of action. Natural products represent an attractive source of biologically active agents, since they may have different mechanisms compared to those of conventional drugs and could be of clinical importance in health care improvement [5]. These efforts have led to the discovery of various important clinical drugs, such as anticancer agents (e.g., taxol and doxorubicin), immunosuppressants (e.g., cyclosporine and doxorubicin), antimalarial agents (e.g., quinine and artemisinin), and lipid-level regulating drugs (e.g., lovastatin and relatives) [6]. Even today, natural products still serve as a fundamental source of diverse biological functions, facilitating the development of chemical biology and drug discovery [7,8].

Gibberellins, a large class of tetracyclic diterpenoid carboxylic acids, regulate several physiological processes throughout the whole plant life cycle [9]. Gibberellic acid (GA3), one of these gibberellins, is industrially produced by liquid cultivation of the ascomycetous fungus *Gibberella fujikuroi* [10]. GA3 is considered an essential hormone for plant growth [11,12], affecting seed germination, stem elongation, leaf area expansion, and maturation of plant sexual organs, as well as reducing the time to flowering [13,14,15]. Therefore, the plant hormone GA3 is widely used to increase the number and weight of fruits, induce organ differentiation, promote shoot elongation, and break seed dormancy in agricultural industries [16,17]. Gibberellic acid formed complex with Terbium could reduce impairment functions in liver and kidney tissues through scavenging against free radicals and antioxidant properties [18]. However, Gibberellic acid has lost the ability to scavenge reactive oxygen that lead to damage in rat tissue’s antioxidative system reasoning in cell death [19,20,21]. The carcinogenic effect and tumor formation of gibberellic acid have been documented after treatment of mice for 22 months [22,23]. Furthermore, GA3 could prevent testicular cell function in rats through loss of germ cells, derangement of the germinal cells, and reduction in the size of the seminiferous tubules and dystrophy of Leydig cells [24,25].

Besides itself pharmacological interest of Gibberellic acid, a series of Gibberellin derivatives bearing two *α*,*β*-unsaturated ketone units showed strong anticancer activities toward a number of human cancer cell lines including HT29, A549, HepG2, and MKN28 [26]. Furthermore, allo-gibberic acid derived with saturated linear amide or with *meta*-substituted benzyl ester functionalities could inhibit FGFR_1_ activation and KDR activation [27]. In addition, GA3-based amides also reduced cellular uptake of free cholesterol in prostate cancer cells, suggesting a novel role of gibberellic acid derivatives in deregulating cholesterol metabolism [28]. Moreover, recent studies have also shown that some aminoalcohols derived from gibberellic acid exhibit considerable potential against a diverse panel of multi-drug-resistant Gram-negative pathogens [29]. Stimulation by this result and continuation of our interest in structural modification of natural products for the development of anticancer agents have led us to the field of diterpene-based aminoalcohols and aminodiols [30,31]. Herein, we report the synthesis of new gibberellic acid-based 3-amino-1,2-diol derivatives and their in vitro antiproliferative evaluation against different human cancer cell lines. A docking-model study was also carried out for the most potent analog.

## 2. Results

### 2.1. Preparation of Epoxyalcohol **3** Based on Allo-Gibberic Acid

**Scheme 1:**
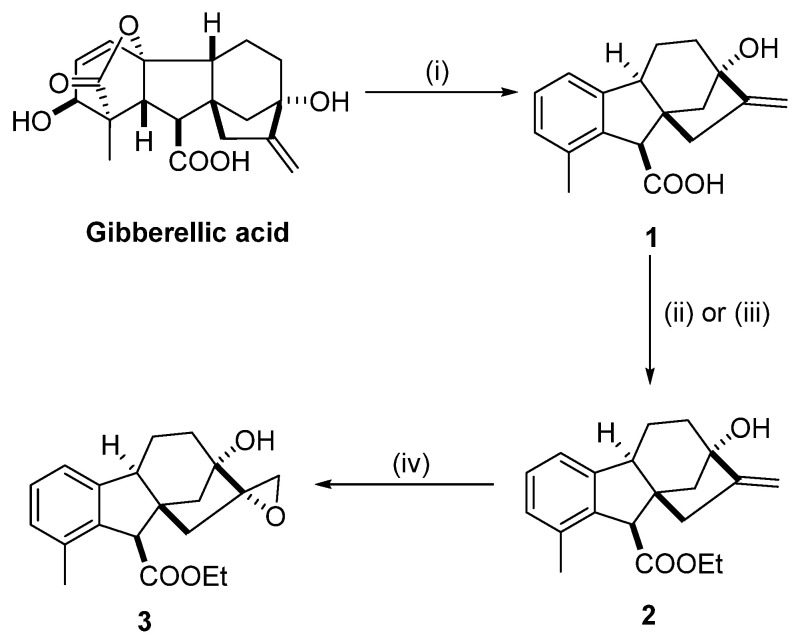
(**i**) HCl 1.2 M, 65 °C, 3 h, 70%; (**ii**) C_2_H_5_I (2 equ.), TBAF 1 M in THF (2.5 equ.), dry THF, Ar atm, 25 °C, 4 h, 95%; (**iii**) C_2_H_5_I (2.5 equ.), Cs_2_CO_3_ (2 equ.), MeCN, reflux, 1 h, 99%; (**iv**) *m*-CPBA 75% (2.5 equ.), dry DCM, 25 °C, 2 h, 62%.

The key intermediate, allo-gibberic acid **1**, was prepared from commercially available gibberellic acid according to literature methods [27,32]. The esterification of **1** was successfully performed by using ethyl iodide in the presence of a stoichiometric amount of tetrabutylammonium fluoride (Bu_4_NF) as the base resulting in ethyl ester **2**. The utility of TBAF, a cheap, non-toxic, air-stable eco-safe organocatalyst [33], plays two important roles in this esterification. First, the F^−^ anion derived from TBAF serves as an effective base for deprotonation of the carboxylic acid; second, the resulting Bu_4_N^+^ carboxylates are partially soluble in organic media and therefore the reaction of carboxylates with alkyl halides via the S_N_2 mechanism would be accelerated [34]. Furthermore, since the cesium ion is well known for its key properties, such as solubility in different organic solvents, high reactivity and large ionic radius [35], *O*-alkylation of **2** mediated by cesium carbonate (Cs_2_CO_3_) was efficiently carried out under mild conditions to give the corresponding ester **2** on a gram scale [36]. The esterification process applying C_2_H_5_I as reactant and Cs_2_CO_3_ was completed at ambient temperatures and delivered the desired ester smoothly and exclusively in high yield [37]. Epoxidation of the terminal alkene group in **2** with *m*-CPBA furnished *cis*-13,16-epoxy alcohol **3** in a stereospecific reaction [30]. The highly stereoselective formation of **3** was explained by hydrogen bonding between the electrophilic peracid oxygen and the olefin in the transition state [38] (Scheme 1).

### 2.2. Synthesis of Allo-Gibberic Acid-Based Aminodiol Derivatives

**Scheme 2:**
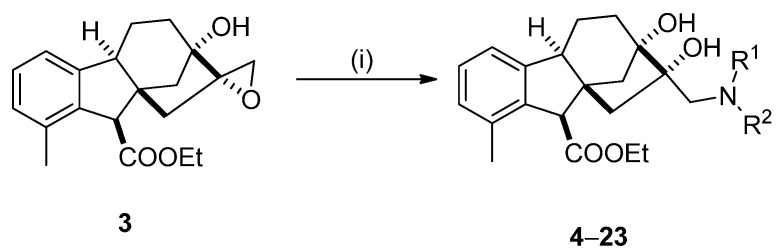
(**i**) R^1^R^2^NH (2 equ.), LiClO_4_ (1 equ.), MeCN, 80–90 °C, 24–48 h, 50–95%.

Since our earlier results demonstrated that substituents at the nitrogen of aminodiols exerted a definite influence on the efficiency of their antiproliferative activity [30], aminodiol library **4**–**23** was prepared by aminolysis of **3** with different primary and secondary amines in the presence of LiClO_4_ as catalyst (Scheme 2, Table 1) [30].

LiClO_4_ shows enhanced reactivity for the ring-opening of epoxides through the coordination of Li^+^ with the epoxide oxygen, rendering the epoxide more susceptible to nucleophilic attack by amines and, therefore, dramatically reducing reaction times and improving yields [39]. Although ClO_4_^−^ can be served as oxidative reagent, there is no oxidation under these condition (NMR determination) [39].

**Scheme 3:**
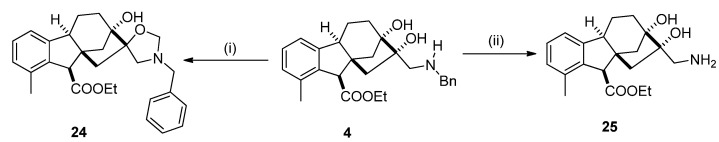
(**i**) 35% HCHO, Et_2_O, 25 °C, 1.5 h, 60%; (**ii**) 5% Pd/C, H_2_ (1 atm), *n*-hexane:EtOAc (1:1), 25 °C, 48 h, 63%.

Since rigidification dramatically improves inhibitory selectivity for antiproliferative effects [40], we attempted to incorporate the hydroxy groups of aminodiols into products with 1,3-oxazine or 1,3-spirooxazoline ring. When aminodiol **4** was reacted with HCHO under mild conditions, spiro-oxazolidine **24** was obtained in highly regioselective ring closure, whereas debenzylation via hydrogenolysis of derivative **4** over Pd/C produced primary aminodiol **25** in moderate yield (Scheme 3) [30].

### 2.3. Synthesis of Azole Derivatives Based on Allo-Gibberic Acid

**Scheme 4:**
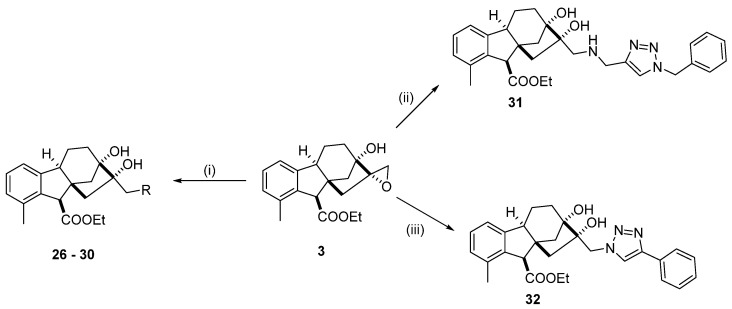
(**i**) Azoles (3 equ.), K_2_CO_3_ (2 equ.), DMF, 80–90 °C, 48 h, 45–75%; (**ii**) (a) Propagylamine (2 equ.), LiClO_4_ (1 equ.), MeCN, 80–90 °C, 48 h, 86%; (b) Benzylazide (1.5 equ.), CuSO_4_.5H_2_O (10% mol), sodium ascorbate (10% mol), *t*-BuOH:H_2_O = 2:1, 25 °C, 48 h, 40%; (**iii**) (a) NaN_3_ (2 equ.), NH_4_Cl (2 equ.), EtOH:H_2_O = 8:2, reflux, 48 h, 65%; (b) Phenylacetylene (2 equ.), CuSO_4_.5H_2_O (2% mol), sodium ascorbate (2% mol), EtOH:H_2_O = 8:2, 25 °C, 24 h, 98%.

As azoles are known to be endowed with a variety of biological activities [41], we have tried to perform the azole-mediated ring-opening of **3** with a variety of azoles (Table 2). Likewise amines, the oxirane ring could only be opened in the presence of K_2_CO_3_ owing to the lower reactivity of *N*-containing heterocycles. A possible reaction pathway through K_2_CO_3_-promoted azole nucleophilicity and subsequent nucleophilic addition to epoxide **3** afforded derivatives **26**–**30** [42]. The reactions were completely clean, furnishing azole-based aminodiol adducts with moderate to satisfactory yields (45–75%) (Scheme 4, Table 2).

Nowadays, CuAAC reaction (copper-catalyzed azide-alkyne cycloaddition) has become the main approach to access 1,2,3-triazoles with high regioselectivity [43]. In order to expand the family of allo-gibberic acid-based azole scaffolds, oxirane **3** was subjected to ring-opening reaction using propargyl amine in acetonitrile under reflux conditions and subsequent 1,3-dipolar cycloaddition reaction [30]. This method is typically called Huisgen cycloaddition [44]. In our case, the reaction of terminal acetylene bearing *N*-propargyl-substituted aminodiol with benzyl azide under Sharpless click chemistry conditions (CuSO_4_·5H_2_O and sodium ascorbate in *t*-BuOH/H_2_O (2:1)) afforded 1,4-disubstituted-1,2,3-triazole **31** in a regioselective manner in satisfactory yield [30]. On the other hand, various triazole derivatives of allo-gibberic acid were synthesized through azide-generated epoxide **3** and NaN_3_ followed by Cu(I)-catalyzed alkyne−azide [3 + 2] cycloaddition of the corresponding azide with phenylacetylene to produce target compound **32** in good yield (Scheme 4) [30].

### 2.4. Isomerization of the Ester Group

**Scheme 5:**
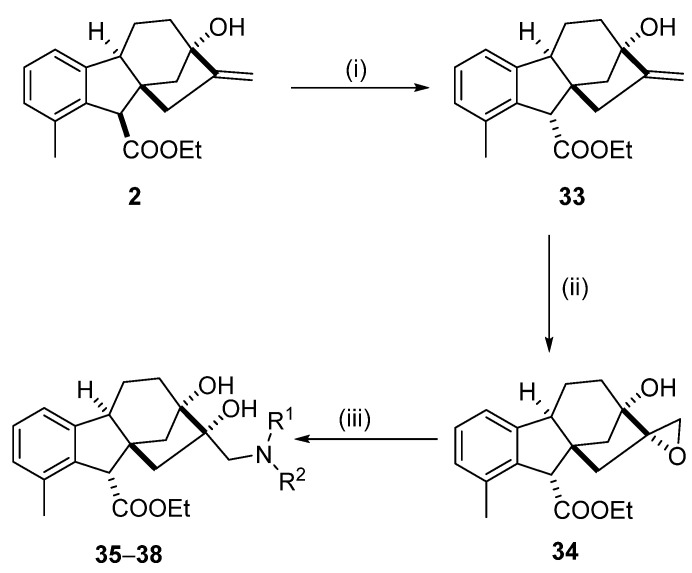
(**i**) NaOEt, EtOH, 25 °C, 12 h, 93%; (**ii**) *m*-CPBA 75% (2.5 equ.), dry DCM, 25 °C, 2 h, 76%. (**iii**) R^1^R^2^NH (2 equ.), LiClO_4_ (1 equ.), MeCN, 80–90 °C, 24–48 h, 60–86%.

**Figure 1:**
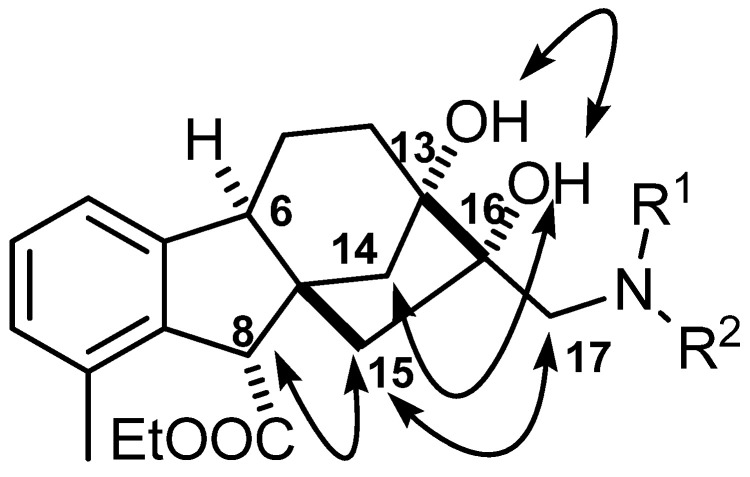
Determination of relative configuration of allo-gibberic acid-based aminodiols **35**–**38**.

Our previous work demonstrated that the configuration of the ester group has a significant effect on biological activity [45]. Therefore, to explore the role of the configuration of the carboxyl group, isomerization of ester **2** at the carboxyl function was carried out under alkaline conditions, resulting in ester **33** with (*S*)-configuration in excellent yield similar to literature results [46,47]. The missing NOE effect between the H-6 on the 5-membered ring and the proton at 8 position proves that the isomerization took place only at the carboxylic group (Figure 1). This rapid and quantitative isomerization allowed the gram-scale synthesis of ester **33**. Subsequently, ester **33** underwent similar reactions including epoxidation, then amine-mediated ring-opening of the corresponding epoxide to afford aminodiols **35**–**38** in moderate yields (Scheme 5, Table 3).

### 2.5. Determination of Relative Configuration of Allo-Gibberic Acid Derivatives

The relative stereochemistry of aminodiols **35**–**38** was proven through NOESY examinations. Significant NOE signals were found between the H-8 and H-15 together with H-15 and H-17, OH-16 and OH-13, as well as between OH-16 and H-14 protons (Figure 1).

**Figure 2:**
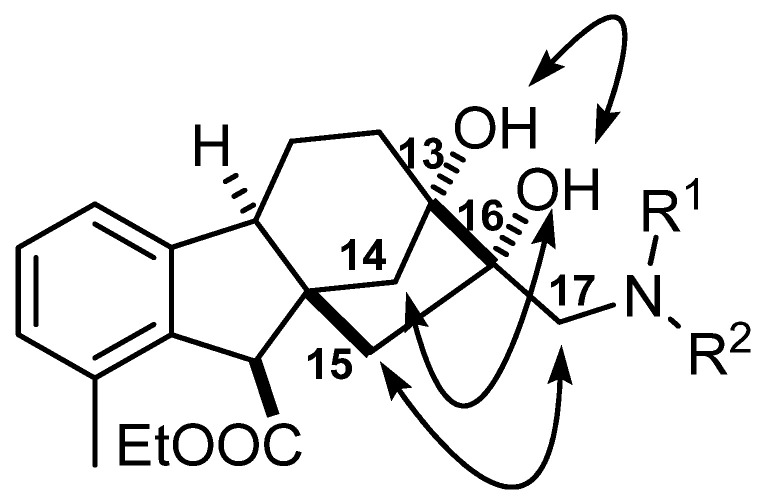
Determination of the relative configuration of allo-gibberic acid-based aminodiols **4**–**30**.

The relative configuration of compounds **4**–**30** was determined through NOESY experiments. Clear NOE signals were observed between OH-13 and OH-16, as well as between H-14 and OH-16 together with H-15 and H-17. Thus, the structure of aminodiols derived from allo-gibberic acid was determined as outlined in Figure 2.

Since neither aminolysis of parent oxirane **3** in alkaline condition nor the hydrogenolysis of *N*-benzyl analog **4** affected the absolute configuration, the relative configuration of the chiral centers of **4**–**25** and **26**–**30** is known to be the same as that of epoxide **3**.

### 2.6. In Vitro Antiproliferative Studies of Gibberellic Acid-Based Aminodiols

**Figure 3:**
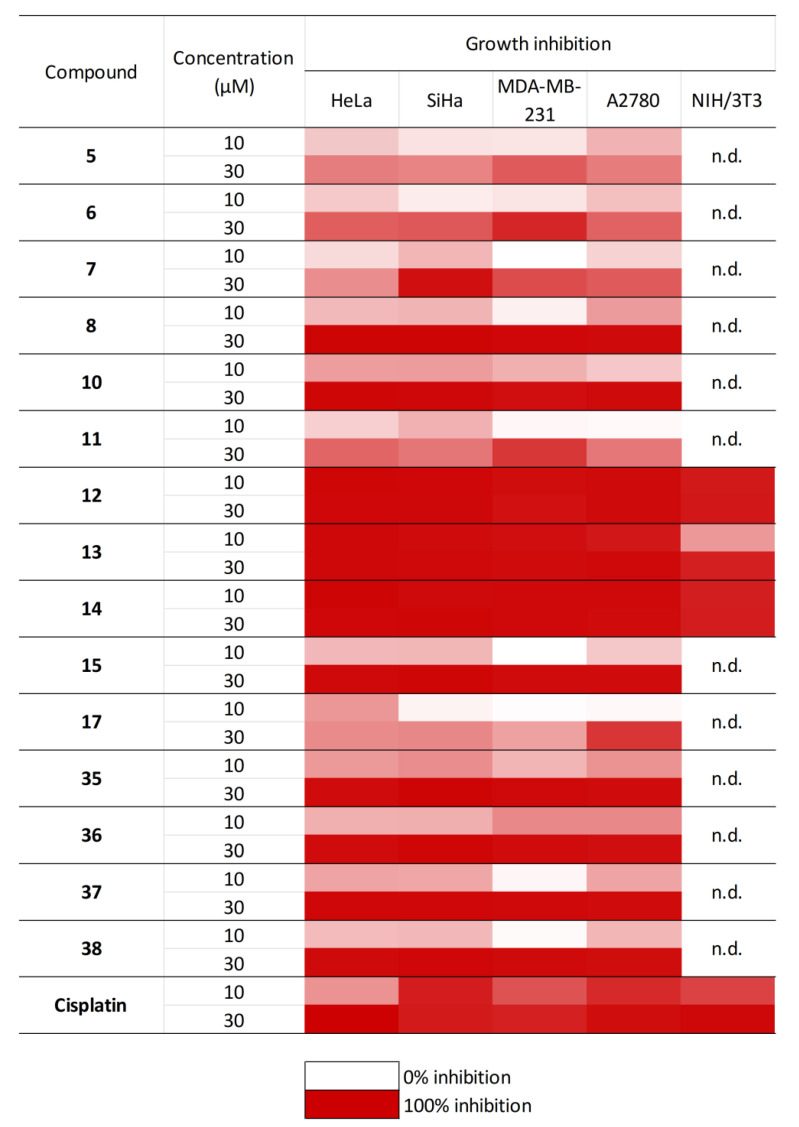
Antiproliferative properties of the selected allo-gibberic acid-based aminodiols against cancer cells and NIH/3T3 fibroblasts. n.d.: not determined.

The in vitro antiproliferative activities of the synthesized aminodiols **4**–**38** against a panel of different human cancer cell lines of gynecological origin, including cervical (SiHA and HeLa), breast (MCF7 and MDA-MB-231), and ovary (A2780) cancers were assayed by the MTT method [48]. Moreover, the most active molecules (**13**–**15**) were additionally tested using non-cancerous fibroblast cells to obtain data concerning their cancer selectivity. Cisplatin, a clinically applied anticancer agent, was used as a reference compound and the results are summarized in Figure 3 and Table S1 in Supporting Information. The obtained results indicated that *N*-benzyl-substituted aminodiols exhibit considerable cancer cell growth-inhibiting capacities. Among them, *N*-naphthylethyl-substituted aminodiol derivatives showed the most pronounced antiproliferative activities comparable to those of reference agent cisplatin. One of these agents, (**13**) exhibited a modest cancer selectivity with a higher calculated IC_50_ value on NIH/3T3 fibroblast cells (10.88 μM) than on the malignant cell lines (4.38–7.49 μM). Compounds **12** and **14** inhibited the growth of cancer cells and fibroblasts in the same concentration range. On the other hand, none of the prepared azole derivatives exerted relevant activity.

### 2.7. In Vitro Antioxidant Activity Studies of Gibberellic Acid-Based Aminodiols

Since oxidative stress and free radicals are generally regarded as crucial factors of carcinogenesis, antioxidants and free-radical scavengers can be considered to be useful agents for preventive or therapeutic intervention [49]. Moreover, a substantial part of natural products and their derivatives exert antioxidant or scavenging activity, this can be a relevant component of the bioactivity of the presented diterpene analogs. As a consequence, in vitro antioxidant activities of selected *N*-aliphatic- and *N*-aryl-substituted aminodiols were determined by 1,1-diphenyl-2-picrylhydrazyl (DPPH) assay. In order to obtain results concerning the relationship between the potential antioxidant properties and the antiproliferative actions of the tested molecules, three potent (**12**, **13**, **14**) and three substantially less active analogs (**16**, **25**, **30**) were tested. None of the six molecules elicited considerable activity in the applied concentration range (3–100 µM). In the same range, the reference agent Trolox exerted a pronounced scavenging activity with a calculated IC_50_ value of 20.2 µM (see relevant data in Figure S1, Supporting Information).

### 2.8. Molecular Docking

**Figure 4:**
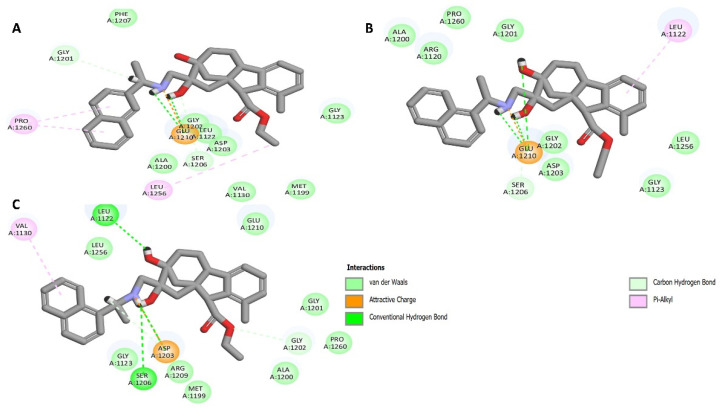
2D Diagram of the interactions between (**A**) compound **12**, (**B**) compound **13**, and (**C**) compound **14** with ALK ATP binding site.

Protein kinases are enzymes that function as components of signal transduction pathways, playing a central role in diverse biological processes, such as control of cell growth, metabolism, differentiation, and apoptosis [50]. Therefore, they have become an important target of many types of cancer cells and numerous new inhibitors targeting these enzymes have been developed as anticancer drugs [51]. The recent report highlighted the significant inhibitory activity of GA_3_ derivatives against RPTK (Receptor protein-Tyrosine Kinase) enzymes [27]. Besides that, many *ent*-kaurene type tetracyclic diterpenes, such as amethystoidin A [52], oridonin, and ponicidin [53], might possibly inhibit PSTK (Receptor serine/threonine kinases) activation. In order to identify whether the antiproliferative activity of prepared allo-gibberic acid derivatives was related to the activation and expression of RPTK or RSTK receptor, a docking study was employed to predict the possible target of the bioactive compounds (**12**, **13**, and **14**). To reach this goal, a variety of protein kinases, such as serine/threonine kinase (Pim-1, AKT and RAFI) and tyrosine kinases (MAP and ALK), were used as templates for the docking study (Table S2 in the supplementary file). The obtained results indicated that these compounds could have a high affinity toward Anaplastic lymphoma kinase (ALK) by forming strong bonds with the hinge region and the ATP binding site. The CDocker energy of the tested compounds varied between −43.4401 and −46.6684. Figure 4 shows the complexes of compounds **12**, **13**, and **14** with the ALK ATP binding site (PDB code: 3AOX, resolution: 1.75 Å) [54]. The docking results of the three studied compounds show the importance of the aminodiol function in forming hydrogen bonding and ionic interactions with the key amino acids. The results of in-silico ADMET study showed that all tested compounds might have good absorption properties with moderate ability to penetrate the BBB.

## 3. Discussion

Based on results acquired by the antiproliferative assay, the structure–activity relationship (SAR) was built up as follows.

The *N*-benzyl-substituted aminodiol showed moderate antiproliferative activity (approximately 30% at 10 µM). Either debenzylation or ring closure drastically reduced the potency. Moreover, the introduction of an alkyl group to the (*α*) position of the benzyl group showed almost no significant change in the activity regardless of the stereochemistry or length of the introduced alkyl group. Similarly, replacement of the benzyl substituent by *N*,*N*-dibenzyl or aliphatic groups eliminated the activity of the resultant compounds, suggesting the importance of the secondary amine moiety as a hydrogen bond donator (HBD).

Substituted *N*-benzyl derivatives with electron-withdrawing substituents (for example, fluorine) exerted a moderate increase in activity, while the electron-donating (methoxy) showed a similar effect against the tested cancer cell lines. The combination of (*R*)-(*α*)-methyl group and fluorine as aromatic substituent at the *para* position failed to show any remarkable antiproliferative activity.

The 1- or 2-Naphthyl-substituted aminodiols showed enhanced activity against all studied cell lines. The introduction of either (*R*) or (*S*) methyl group at the (*α*) position exerted a very potent improvement on activity against all tested cancer cell lines with an IC_50_ value as low as 4–7 μM, demonstrating the crucial role of the methyl group at (*α*) position for the design and synthesis of novel antiproliferative agents.

Considering the effect of the stereochemistry of the carboxyl group on antiproliferative activity, aminodiols with *R* configuration were found to be more effective compared to their corresponding isomers.

## 4. Materials and Methods

### 4.1. General Methods

Commercially available compounds were used as obtained from suppliers (Molar Chemicals Ltd., Halásztelek, Hungary; Merck Ltd., Budapest, Hungary, and VWR International Ltd., Debrecen, Hungary), while solvents were dried according to standard procedures. Optical rotations were measured in MeOH at 20 °C, with a Perkin-Elmer 341 polarimeter (PerkinElmer Inc., Shelton, CT, USA). Chromatographic separations and monitoring of reactions were carried out on Merck Kieselgel 60 (Merck Ltd., Budapest, Hungary). HR-MS flow injection analysis was performed with a Thermo Scientific Q Exactive Plus hybrid quadrupole-Orbitrap (Thermo Fisher Scientific, Waltham, MA, USA) mass spectrometer coupled to a Waters Acquity I-Class UPLC™ (Waters, Manchester, UK). Melting points were determined on a Kofler apparatus (Nagema, Dresden, Germany) and are uncorrected. ^1^H- and ^13^C JMOD NMR spectra were recorded on Brucker Avance DRX 500 spectrometer (Bruker Biospin, Karlsruhe, Baden Württemberg, Germany) [500 MHz (^1^H) and 125 MHz (^13^C), δ = 0 (TMS)]. Chemical shifts are expressed in ppm (δ) relative to TMS as the internal reference. J values are given by Hz. For ^13^C JMOD NMR spectra, quaternary carbon, carbonyl, and methylene signals have opposite phase to those of methine and methyl resonances. ^1^H, ^13^C JMOD, COSY, HSQC, HMBC, and NOESY NMR spectra of new compounds are available in the Supplementary Materials (Figures S2–S95).

Gibberellic acid is commercially available from Merck Co with *ee*% = 90% ([*α*${]}_{D}^{20}$ = +78.0, c = 2% in MeOH). Allo-gibberic acid (**1**) was prepared from gibberellic acid according to literature methods [32]. All spectroscopic data were similar to those described therein.

*Ethyl (2R,4b’S,7’S,9a’R,10’R)-7’-hydroxy-1’-methyl-4b’,6’,7’,10’-tetrahydro-5’H,9’H-spiro[oxirane-2,8’-[7,9a] methanobenzo[a]azulene]-10’-carboxylate (* **2** *)*

**Method A:** To the solution of allo-gibberic acid **1** (2.0 g, 7.03 mmol) in dry THF (75 mL) under argon atmosphere, 1.12 mL ethyl iodide (14.06 mmol, 2 equ.) and 17.0 mL TBAF 1M in THF (17.57 mmol, 2.5 equ.) were added. After stirring for 4 h at room temperature, the mixture was evaporated. The crude product was then diluted with water (40 mL) and extracted by DCM (3 × 50 mL). The organic phase was washed with brine, dried over sodium sulfate, and then evaporated under reduced pressure. The crude product was purified by column chromatography on silica gel (*n*-hexane: EtOAc = 2:1) to afford ester **2** as white crystals (92%).

**Method B:** To the solution of allo-gibberic acid **1** (5.0 g, 17.58 mmol) in MeCN (250 mL), ethyl iodide (3.5 mL, 43.95 mmol, 2.5 equ.) and cesium carbonate (11.45 g, 35.16 mmol, 2 equ.) were added. After treating under reflux conditions at 90 °C for 1 h, the reaction mixture was cooled to room temperature, then filtered to remove the precipitate. The solution was concentrated, then dissolved in 150 mL CH_2_Cl_2_. The CH_2_Cl_2_ layer was washed with saturated sodium bicarbonate (3 × 100 mL), dried over sodium sulfate and then evaporated. The crude product was purified by column chromatography on silica gel (*n*-hexane: EtOAc = 2:1) to afford ester **2** (97%).

Compound **2**: white crystals; m.p.: 77–78 °C; [*α*${]}_{D}^{20}$ = +106.2 (c 0.13, MeOH); ^1^H NMR (500 MHz, DMSO-*d_6_*): δ = 7.10 (1H, t, *J* = 7.5 Hz), 6.97 (1H, d, *J* = 9.1 Hz), 6.92 (1H, d, *J* = 9.14 Hz), 4.97 (1H, s), 4.91 (1H, s), 4.66 (1H, s), 4.18 (2H, dq, *J* = 1.6, 7.1 Hz), 3.96 (1H, s), 2.79 (1H, dd, *J* = 4.6, 12.4 Hz), 2.18–2.12 (1H, m), 2.10–2.04 (4H, m), 2.02–1.89 (2H, m), 1.82–1.76 (2H, m), 1.57–1.51 (1H, m), 1.43–1.33 (1H, m), 1.24 ppm (3H, t, *J* = 7.1 Hz); ^13^C NMR and JMOD (125 MHz, DMSO-*d_6_*): δ = 14.8, 19.8, 22.0, 34.4, 40.1, 48.2, 51.8, 53.3, 54.6, 60.7, 79.6, 103.2, 120.0, 127.5, 128.9, 134.8, 138.9, 145.1, 155.3, 171.3; HR-MS (ESI): *m/z* calcd for C_20_H_25_O_3_ [M + H]^+^: 313.1725; found: 313.1801.

*Ethyl (4bR,7S,9aS,10S)-7-hydroxy-1-methyl-8-methylene-4b,6,7,8,9,10-hexahydro-5H-7,9a-methanobenzo[a]azulene-10-carboxylate (* **33** *)*

A solution of ester **2** (2.0 g, 6.4 mmol) in dry EtOH (40 mL) was added to a solution of NaOEt (0.03 g Na, 1.35 mmol) in dry EtOH (10 mL) at 0 °C. After stirring at room temperature for 12 h (monitored by TLC), the solution was evaporated to 10 mL then Et_2_O (80 mL) was added. The mixture was extracted with ice-cold H_2_O (2 × 50 mL). The organic layer was dried over (Na_2_SO_4_), filtered and then evaporated. The crude product was purified by column chromatography on silica gel (*n*-hexane:EtOAc = 2:1) to produce the ester **33** as a yellow oily compound.

Yield: 93%; yellow oily compound; [*α*${]}_{D}^{20}$ = −54.1 (c 0.13, MeOH); ^1^H NMR (DMSO-*d6*, 500 MHz): δ = H 7.11 (1H, t, *J* = 7.4 Hz), 6.99–6.92 (2H, m), 4.99 (1H, s), 4.90 (1H, s), 4.64 (1H, s), 4.09 (2H, q, *J* = 6.9 Hz), 3.68 (1H, s), 3.18 (1H, dd, *J* = 4.6, 12.4 Hz), 2.22–2.16 (1H, m), 2.15 (3H, s), 2.13–2.05 (2H, m), 2.00 (1H, d, *J* = 11.3 Hz), 1.76 (1H, td, *J* = 5.7, 12.8 Hz), 1.62 (1H, d, J = 9.9 Hz), 1.56–1.50 (1H, m), 1.31 (1H, td, *J* = 5.3, 12.9 Hz), 1.18 (3H, t, *J* = 7.0 Hz); ^13^C NMR and JMOD (125 MHz, DMSO-*d_6_*): δ = 14.7, 18.7, 22.1, 40.4, 40.9, 46.8, 50.0, 52.6, 55.9, 60.5, 79.8, 102.8, 120.7, 127.8, 128.0, 134.4, 140.2, 145.6, 155.3, 171.6; HR-MS (ESI): *m/z* calcd for C_20_H_25_O_3_ [M + H]^+^: 313.1725; found: 313.1794.

*Ethyl (4bS,7S,8S,9aR,10R)-8-(aminomethyl)-7,8-dihydroxy-1-methyl-4b,6,7,8,9,10-hexahydro-5H-7,9a-methanobenzo[a]azulene-10-carboxylate (* **25** *)*

Aminodiol **4** (500 mg, 1.14 mmol) in *n*-hexane:EtOAc = 1:1 (10.0 mL) was added to a suspension of palladium-on-carbon (5% Pd/C, 0.1 g) in *n*-hexane:EtOAc = 1:1 (10 mL). After being stirred for 48 h under H_2_ atmosphere at room temperature (as monitored by TLC), the mixture was filtered through a Clite Pod. The solution was then evaporated to dryness and recrystallized in Et_2_O to provide primary aminodiol **25** as white crystals.

Yield: 50%; white crystals; m.p.: 92–94 °C; [*α*${]}_{D}^{20}$ = −45.3 (c 0.11, MeOH); ^1^H NMR (DMSO-*d_6_*, 500 MHz): δ = 7.10 (1H, t, *J* = 7.5 Hz), 6.96 (1H, d, *J* = 7.4 Hz), 6.92 (1H, d, *J* = 7.2 Hz), 4.27–4.12 (2H, m), 3.95 (1H, s), 2.76 (1H, dd, *J* = 5.8, 7.0 Hz), 2.66–2.56 (3H, m), 2.24–2.17 (1H, m), 2.09 (3H, s), 1.87 (1H, s), 1.76–1.7 (2H, m), 1.51 (1H, d, *J* = 10.1 Hz), 1.38 (2H, q, *J* = 13.9 Hz), 1.28 (3H, t, *J* = 6.7 Hz); ^13^C NMR and JMOD (125 MHz, DMSO-*d_6_*): δ = 14.8, 19.9, 22.2, 33.6, 38.3, 43.8, 46.0, 52.0, 53.6, 54.9, 60.5, 77.0, 79.8, 120.1, 127.5, 128.9, 134.8, 139.0, 145.2, 171.2; HR-MS (ESI): *m/z* calcd for C_20_H_28_NO_4_ [M + H]^+^: 346.1940; found: 346.2007.

### 4.2. General Procedure for Epoxidation

To the solution of **2** or **33** (2 g, 6.4 mmoL) in dry CH_2_Cl_2_ (100 mL), *m*-CPBA (3.95 g, 16 mmol, 2.5 equ., 70% purity) was added. After stirring for 2 h in dark at room temperature (indicated by TLC), the mixture was washed with a saturated solution of NaHCO_3_ (3 × 100 mL). The organic phase was then dried over Na_2_SO_4_, filtered, and evaporated. The crude product was purified by column chromatography (CHCl_3_: MeOH = 19:1) and subsequently recrystallized in an *n*-hexane: Et_2_O mixture.

*Ethyl (2R,4b’S,7’S,9a’R,10’R)-7’-hydroxy-1’-methyl-4b’,6’,7’,10’-tetrahydro-5’H,9’H-spiro[oxirane-2,8’-[7,9a] methanobenzo[a]azulene]-10’-carboxylate (* **3** *)*

Yield: 62%; white crystals; m.p.: 156–159 °C; [*α*${]}_{D}^{20}$ = +69.0 (c 0.13, MeOH); ^1^H NMR (DMSO-*d_6_*, 500 MHz): δ = 7.11 (1H, t, *J* = 7.4 Hz), 6.96 (2H, t, *J* = 8.4 Hz), 4.29 (1H, s), 4.26–4.12 (2H, m), 4.03 (1H, s), 2.79 (1H, dd, *J* = 3.8, 11.4 Hz), 2.72 (1H, d, *J* = 5.0 Hz), 2.61 (1H, d, *J* = 5.0 Hz), 2.27–2.17 (2H, m), 2.08 (3H, s), 1.82–1.68 (2H, m), 1.66–1.53 (3H, m), 1.42 (1H, d, *J* = 15.1 Hz), 1.26 (3H, t, *J* = 7.1 Hz); ^13^C NMR and JMOD (125 MHz, DMSO-*d_6_*): δ = 14.8, 19.8, 21.4, 34.8, 36.3, 46.9, 47.0, 51.3, 53.3, 54.6, 60.6, 64.3, 74.8, 120.1, 127.6, 129.0, 134.7, 138.8, 144.7, 171.2; HR-MS (ESI): *m/z* calcd for C_20_H_25_O_4_ [M + H]^+^: 329.1675; found: 329.1748.

*Ethyl (2R,4b’R,7’S,9a’R,10’S)-7’-hydroxy-1’-methyl-4b’,6’,7’,10’-tetrahydro-5’H,9’H-spiro[oxirane-2,8’-[7,9a]methanobenzo[a]azulene]-10’-carboxylate (* **34** *)*

Yield: 76%; colorless oily compound; [*α*${]}_{D}^{20}$ = −236.1 (c 0.10, MeOH); ^1^H NMR (DMSO-*d_6_*, 500 MHz): δ = 7.12 (1H, t, *J* = 7.4 Hz), 6.97 (2H, d, *J* = 7.3 Hz), 4.40 (1H, s), 4.11 (2H, q, *J* = 7.0 Hz), 3.74 (1H, s), 3.14 (1H, dd, *J* = 4.5, 12.3 Hz), 2.71 (1H, d, *J* = 5.2 Hz), 2.60 (1H, d, *J* = 5.0 Hz), 2.23–2.16 (1H, m), 2.15 (3H, s), 2.14–2.11 (1H, m), 1.80 (1H, d, *J* = 15.2 Hz), 1.70 (1H, td, *J* = 5.8, 12.5 Hz), 1.63 (2H, d, *J* = 13.7 Hz), 1.58–1.42 (2H, m), 1.20 (3H, t, *J* = 7.0 Hz); ^13^C NMR and JMOD (125 MHz, DMSO-*d_6_*): δ = 14.7, 18.7, 21.5, 36.5, 40.9, 45.7, 46.9, 49.7, 52.6, 56.0, 60.6, 64.4, 75.1, 120.8, 127.9, 128.1, 134.4, 140.2, 145.2, 171.6; HR-MS (ESI): *m/z* calcd for C_20_H_25_O_4_ [M + H]^+^: 329.1675; found: 329.1744.

### 4.3. General Procedure for Ring-Opening of Epoxide with Amines

To the solution of epoxide **3** (100 mg, 0.3 mmol) in MeCN (15.0 mL), LiClO_4_ (70 mg, 0.68 mmol, 2.25 equ.) and appropriate amines (0.68 mmol, 2.25 equ.) were added. The reaction mixture was treated under reflux conditions for 24 h at 70–80 °C followed by solvent evaporation. The residue was diluted with water (15 mL), then extracted with CH_2_Cl_2_ (3 × 15 mL). The crude product was purified by column chromatography on silica gel (CH_2_Cl_2_:MeOH = 19:1).

*Ethyl (4bS,7S,8S,9aR,10R)-8-((benzylamino)methyl)-7,8-dihydroxy-1-methyl-4b,6,7,8,9,10-hexahydro-5H-7,9a-methanobenzo[a]azulene-10-carboxylate (* **4** *)*

Prepared with benzylamine. Yield: 70%; white powder; m.p.: 97–99 °C; [*α*${]}_{D}^{20}$ = −66.9 (c 0.13, MeOH); ^1^H NMR (DMSO-*d_6_*, 500 MHz): δ = 7.27 (4H, d, *J* = 4.3 Hz), 7.23–7.18 (1H, m), 7.09 (1H, t, *J* = 7.4 Hz), 6.95 (1H, d, *J* = 7.6 Hz), 6.89 (1H, d, *J* = 7.3 Hz), 4.51 (1H, s), 4.26–4.06 (2H, m), 3.92 (1H, s), 3.90 (1H, s), 3.67 (2H, d, *J* = 4.0 Hz), 2.73 (1H, dd, *J* = 5.1, 12.0 Hz), 2.62–2.52 (2H, m), 2.50–2.45 (1H, m), 2.18–2.10 (1H, m), 2.07 (3H, s), 1.73–1.58 (2H, m), 1.48–1.29 (4H, m), 1.26 (3H, t, *J* = 13.6 Hz); ^13^C NMR and JMOD (125 MHz, DMSO-*d_6_*): δ = 14.8, 19.9, 22.3, 33.7, 39.2, 46.0, 51.8, 52.0, 53.5, 53.9, 54.9, 60.5, 77.3, 80.0, 120.0, 127.0, 127.4, 128.3, 128.3, 128.5, 128.6, 128.8, 134.7, 139.0, 141.1, 145.2, 171.2; HR-MS (ESI): *m/z* calcd for C_27_H_34_NO_4_ [M + H]^+^: 436.2410; found: 436.2483.

*Ethyl (4bS,7S,8S,9aR,10R)-7,8-dihydroxy-1-methyl-8-((((S)-1-phenylethyl)amino)methyl)-4b,6,7,8,9,10-hexahydro-5H-7,9a-methanobenzo[a]azulene-10 carboxylate (* **5** *)*

Prepared with (*S*)-(*α*)-methylbenzylamine. Yield: 88%; yellow oil; [*α*${]}_{D}^{20}$ = +41.5 (c 0.14, MeOH); ^1^H NMR (DMSO-*d_6_*, 500 MHz): δ = 7.26–7.23 (5H, m), 7.10 (1H, t, *J* = 7.4 Hz), 6.96 (1H, d, *J* = 7.5 Hz), 6.84 (1H, d, *J* = 7.2 Hz), 4.56 (1H, s), 4.27–4.12 (2H, m), 3.92 (1H, s), 3.87 (1H, s), 3.66 (1H, q, *J* = 6.4 Hz), 2.69 (1H, dd, *J* = 5.2, 11.9 Hz), 2.54 (1H, d, *J* = 12.8 Hz), 2.45 (1H, d, *J* = 12.5 Hz), 2.26 (1H, d, *J* = 10.5 Hz), 2.07 (3H, s), 2.04–2.01 (1H, m), 1.64 (1H, ddd, *J* = 6.0, 13.1, 13.1Hz), 1.50 (1H, dd, *J* = 4.6, 12.1 Hz), 1.45–1.36 (2H, m), 1.32–1.25 (4H, m), 1.24–1.19 (4H, m); ^13^C NMR and JMOD (125 MHz, DMSO-*d_6_*): δ = 14.9, 19.9, 22.1, 25.2, 33.7, 39.0, 46.0, 50.3, 52.0, 53.5, 54.8, 58.5, 60.5, 77.4, 79.9, 119.9, 126.8, 126.8, 127.0, 127.5, 128.6, 128.6, 128.8, 134.7, 139.0, 145.3, 146.4, 171.3; HR-MS (ESI): *m/z* calcd for C_28_H_36_NO_4_ [M + H]^+^: 450.2566; found: 450.2639.

*Ethyl (4bS,7S,8S,9aR,10R)-7,8-dihydroxy-1-methyl-8-((((R)-1-phenylethyl)amino)methyl)-4b,6,7,8,9,10-hexahydro-5H-7,9a-methanobenzo[a]azulene-10-carboxylate (* **6** *)*

Prepared with (*R*)-(*α*)-methylbenzylamine. Yield: 72%; white crystals; m.p.: 104–105 °C; [*α*${]}_{D}^{20}$ = −99.3 (c 0.13, MeOH); ^1^H NMR (DMSO-*d_6_*, 500 MHz): δ = 7.30 (4H, m), 7.20 (1H, m), 7.08 (1H, t, *J* = 7.4 Hz), 6.95 (1H, d, *J* = 7.8 Hz), 6.84 (1H, d, *J* = 8.1 Hz), 4.45 (1H, s), 4.27–4.13 (2H, m), 3.92 (1H, s), 3.87 (1H, s), 3.60 (1H, q, *J* = 5.9 Hz) 2.71 (1H, dd, *J* = 5.1, 12.0 Hz), 2.53 (1H, d, *J* = 10.6 Hz), 2.39 (2H, t, *J* = 2.5, 13.1Hz), 2.13–2.08 (1H, m) 2.07 (3H, s), 1.64 (1H, ddd, *J* = 6.6, 13.1, 14.1 Hz), 1.53 (1H, dd, *J* = 4.4, 12.7 Hz), 1.45–1.30 (4H, m), 1.26 (3H, t, *J* = 7.5 Hz), 1.21 (3H, d, *J* = 6.9 Hz); ^13^C NMR and JMOD (125 MHz, DMSO-*d_6_*): δ = 14.8, 19.9, 22.3, 24.5, 33.6, 39.2, 46.0, 50.3, 52.0, 53.5, 54.9, 58.5, 60.5, 77.2, 80.0, 120.0, 126.8, 126.9, 127.0, 127.4, 128.7, 128.7, 128.8, 134.7, 139.0, 145.2, 146.5, 171.2; HR-MS (ESI): *m/z* calcd for C_28_H_36_NO_4_ [M + H]^+^: 450.2566; found: 450.2637.

*Ethyl (4bS,7S,8S,9aR,10R)-7,8-dihydroxy-1-methyl-8-((((R)-1-phenylpropyl)amino)methyl)-4b,6,7,8,9,10-hexahydro-5H-7,9a-methanobenzo[a]azulene-10-carboxylate (* **7** *)*

Prepared with (*S*)-(−)-(*α*)-ethylbenzylamine. Yield: 72%; white crystals; m.p.: 55–57 °C; [*α*${]}_{D}^{20}$ = −97.0 (c 0.11, MeOH); ^1^H NMR (DMSO-*d_6_*, 500 MHz): δ = 7.30 (2H, t, *J* = 7.4 Hz), 7.26–7.19 (3H, m), 7.08 (1H, t, *J* = 7.4 Hz), 6.95 (1H, d, *J* = 7.7 Hz), 6.95 (1H, d, *J* = 7.2 Hz), 4.49 (1H, s), 4.28–4.12 (2H, m), 3.92 (1H, s), 3.90 (1H, s), 3.32 (1H, m), 2.71 (1H, dd, *J* = 4.9, 11.9 Hz), 2.56–2.51 (1H, m), 2.43–2.31 (2H, m), 2.13–2.08 (1H, m), 2.08 (3H, s), 1.69–1.59 (2H, m), 1.55–1.38 (4H, m), 1.36–1.24 (5H, m), 0.70 (3H, t, *J* = 7.2 Hz), ^13^C NMR and JMOD (125 MHz, DMSO-*d_6_*): δ = 11.1, 14.8, 19.9, 22.2, 30.5, 33.6, 39.2, 46.0, 50.3, 52.0, 53.5, 54.9, 60.5, 65.1, 77.2, 80.0, 120.0, 127.1, 127.4, 127.5, 127.5, 128.6, 128.6, 128.8, 134.7, 139.0, 144.8, 145.2, 171.2; HR-MS (ESI): *m/z* calcd for C_29_H_38_NO_4_ [M + H]^+^: 464.2723; Found: 464.2792.

*Ethyl (4bS,7S,8S,9aR,10R)-7,8-dihydroxy-1-methyl-8-((((S)-1-phenylpropyl)amino)methyl)-4b,6,7,8,9,10-hexahydro-5H-7,9a-methanobenzo[a]azulene-10-carboxylate (* **8** *)*

Prepared with (*R*)-(+)-(*α*)-ethylbenzylamine. Yield: 72%; white crystals; m.p.: 114–115 °C; [*α*${]}_{D}^{20}$ = −37.4 (c 0.11, MeOH), ^1^H NMR (DMSO-*d_6_*, 500 MHz): δ = 7.26–7.21 (5H, m), 7.10 (1H, t, *J* = 7.4 Hz), 6.96 (1H, d, *J* = 7.6 Hz), 6.83 (1H, d, *J* = 7.2 Hz), 4.56 (1H, s), 4.28–4.01 (2H, m), 3.91 (1H, s), 3.88 (1H, s), 3.44–3.38 (1H, m), 2.69 (1H, dd, *J* = 5.0, 11.8 Hz), 2.54 (1H, d, *J* = 10.5 Hz), 2.42 (1H, d, *J* = 11.3 Hz), 2.25 (1H, d, *J* = 11.3 Hz), 2.08 (3H, s), 2.05–2.01 (1H, m), 1.68–1.57 (2H, m), 1.53–1.35 (4H, m), 1.30–1.26 (4H, m), 1.22–1.13 (1H, m), 0.74 (3H, t, *J* = 7.3 Hz); ^13^C NMR and JMOD (125 MHz, DMSO-*d_6_*): δ = 11.1, 14.8, 19.9, 22.1, 31.3, 33.8, 39.0, 46.0, 50.4, 52.0, 53.5, 54.8, 60.5, 65.1, 77.4, 79.9, 119.9, 127.0, 127.1, 127.5, 127.5, 128.4, 128.4, 128.8, 134.7, 139.0, 144.8, 145.2, 171.3; HR-MS (ESI): *m/z* calcd for C_29_H_38_NO_4_ [M + H]^+^: 464.2723; Found: 464.2792.

*Ethyl (4bS,7S,8S,9aR,10R)-8-(((4-fluorobenzyl)amino)methyl)-7,8-dihydroxy-1-methyl-4b,6,7,8,9,10-hexahydro-5H-7,9a-methanobenzo[a]azulene-10-carboxylate (* **9*)***

Prepared with 4-fluorobenzylamine. Yield: 85%; orange crystals; m.p.: 253–254 °C; [*α*${]}_{D}^{20}$ = −44.3 (c 0.11, MeOH); ^1^H NMR (DMSO-*d_6_*, 500 MHz): δ = 7.53 (2H, dd, *J* = 5.6, 8.4 Hz), 7.25 (2H, t, *J* = 8.9 Hz), 7.12 (1H, t, *J* = 7.5 Hz), 6.95 (1H, d, *J* = 7.5 Hz), 6.93 (1H, d, *J* = 7.5 Hz), 5.27 (1H, s), 4.24–4.11 (3H, m), 4.12–4.07 (2H, s), 3.99 (1H, s), 3.02 (1H, d, *J* = 12.5 Hz), 2.82–2.76 (1H, dd, *J* = 6.72, 12.5 Hz), 2.74 (1H, d, *J* = 13.2 Hz), 2.5–2.49 (1H, m), 2.25–2.17 (1H, m), 2.08 (3H, s), 1.75 (1H, ddd, *J* = 7.1, 14.6, 13.7 Hz), 1.61–1.37 (5H, m), 1.27 (3H, t, *J* = 7.2 Hz); ^13^C NMR and JMOD (125 MHz, DMSO-*d_6_*): δ = 14.8, 19.9, 22.1, 33.4, 38.1, 44.8, 49.8, 50.5, 51.7, 54.0, 54.6, 60.6, 75.8, 79.6, 115.8, 116.0, 120.2, 127.5, 128.9, 133.1, 133.2, 134.8, 138.9, 144.9, 161.9, 163.8, 171.2; HR-MS (ESI): *m/z* calcd for C_27_H_33_FNO_4_ [M + H]^+^: 454.2315; found: 454.2392.

*Ethyl (4bS,7S,8S,9aR,10R)-8-((((S)-1-(4-fluorophenyl)ethyl)amino)methyl)-7,8-dihydroxy-1-methyl-4b,6,7,8,9,10-hexahydro-5H-7,9a-methanobenzo[a]azulene-10-carboxylate (* **10** *)*

Prepared with (*R*)-4-fluoro-(*α*)-methylbenzylamine. Yield: 92%; white crystals; m.p.: 84–86 °C, [*α*${]}_{D}^{20}$ = −34.9 (c 0.10, MeOH); ^1^H NMR (DMSO-*d_6_*, 500 MHz): δ = 7.29 (2H, t, *J* = 6.8 Hz), 7.09 (1H, t, *J* = 7.7 Hz), 7.04 (2H, t, *J* = 8.4 Hz), 6.96 (1H, d, *J* = 7.5 Hz), 6.84 (1H, d, *J* = 7.3 Hz), 4.55 (1H, s), 4.27–4.11 (2H, m), 3.92 (1H, s), 3.83 (1H, s), 3.67 (1H, q, *J* = 6.3 Hz), 2.69 (1H, dd, *J* = 5.0, 11.8 Hz), 2.53 (1H, d, *J* = 9.6 Hz), 2.44 (1H, d, *J* = 11.3 Hz), 2.23 (1H, d, *J* = 11.4 Hz), 2.07 (3H, s), 2.06–2.02 (1H, m), 1.69–1.60 (1H, m), 1.51 (1H, dd, *J* = 3.6, 13.4 Hz), 1.45–1.35 (2H, m), 1.30–1.13 (8H, m); ^13^C NMR and JMOD (125 MHz, DMSO-*d_6_*): δ = 14.8, 19.9, 22.1, 25.2, 33.7, 39.0, 46.0, 50.2, 51.9, 53.4, 54.8, 57.8, 60.5, 77.4, 79.9, 115.1, 115.3, 119.9, 127.5, 128.6, 128.6, 128.8, 134.7, 139.0, 145.2, 160.4, 162.3, 171.2; HR-MS (ESI): *m/z* calcd for C_28_H_35_FNO_4_ [M + H]^+^: 468.2472; found: 468.2546.

*Ethyl (4bS,7S,8S,9aR,10R)-7,8-dihydroxy-8-(((4-methoxybenzyl)amino)methyl)-1-methyl-4b,6,7,8,9,10-hexahydro-5H-7,9a-methanobenzo[a]azulene-10-carboxylate (* **11** *)*

Prepared with 4-methoxybenzylamine. Yield: 60%; white crystals; m.p.: 224–225 °C, [*α*${]}_{D}^{20}$ = −54.1 (c 0.13, MeOH); ^1^H NMR (DMSO-*d_6_*, 500 MHz): δ = 7.39 (2H, d, *J* = 8.5 Hz), 7.11 (1H, t, *J* = 7.4 Hz), 6.99–6.91 (4H, m), 5.24 (1H, s), 4.27–4.11 (2H, m), 4.05 (3H, s), 3.98 (1H, s), 3.76 (3H, s), 2.99 (1H, d, *J* = 12.5 Hz), 2.82–2.73 (2H, m), 2.54–2.50 (1H, m), 2.25–2.16 (1H, m), 2.08 (3H, s), 1.80–1.71 (1H, ddd, *J* = 6.1, 14.2, 12.7 Hz), 1.62–1.37 (5H, m), 1.28 (3H, t, *J* = 7.1 Hz); ^13^C NMR and JMOD (125 MHz, DMSO-*d_6_*): δ = 14.8, 19.9, 22.1, 33.4, 38.2, 44.9, 49.5, 50.8, 51.7, 54.0, 54.7, 55.6, 60.6, 75.8, 79.7, 114.5, 114.5, 120.3, 123.8, 127.6, 129.0, 132.4, 132.4, 134.8, 138.9, 144.9, 160.2, 171.2; HR-MS (ESI): *m/z* calcd for C_28_H_36_NO_5_ [M + H]^+^: 466.2515; found: 466.2588.

*Ethyl (4bS,7S,8S,9aR,10R)-7,8-dihydroxy-1-methyl-8-(((S)-1-(naphthalen-2-yl)ethyl)amino)methyl)-4b,6,7,8,9,10-hexahydro-5H-7,9a-methanobenzo[a]azulene-10-carboxylate (* **12** *)*

Prepared with (*S*)-(−)-1-(2-naphthyl)ethylamine. Yield: 85%; white crystals; m.p.: 71–72 °C, [*α*${]}_{D}^{20}$ = −103.2 (c 0.075, MeOH); ^1^H NMR (DMSO-*d_6_*, 500 MHz): δ = 7.89–7.82 (3H, m), 7.74 (1H, s), 7.52–7.43 (3H, m), 7.08 (1H, t, *J* = 7.4 Hz), 6.95 (1H, d, *J* = 7.5 Hz), 6.86 (1H, d, *J* = 7.2 Hz), 4.46 (1H, s), 4.27–4.11 (2H, m), 3.93–3.86 (2H, m), 3.81 (1H, s), 2.70 (1H, dd, *J* = 5.0, 12.1 Hz), 2.54–2.50 (1H, m), 2.47–2.35 (2H, m), 2.11–2.08 (1H, m), 2.07 (3H, s), 1.68–1.59 (1H, m), 1.54 (1H, dd, *J* = 3.6, 13.4 Hz), 1.46–1.39 (2H, m), 1.38–1.21 (8H, m); ^13^C NMR and JMOD (125 MHz, DMSO-*d_6_*): δ = 14.8, 19.9, 22.3, 24.3, 33.6, 39.2, 46.0, 50.3, 52.0, 53.5, 54.9, 58.6, 60.5, 77.3, 79.9, 120.1, 125.2, 125.3, 125.8, 126.4, 127.4, 127.9, 128.0, 128.4, 128.8, 132.7, 133.4, 134.7, 139.0, 144.8, 145.2, 171.3; HR-MS (ESI): *m/z* calcd for C_32_H_38_NO_4_ [M + H]^+^: 500.2723; found: 500.2795.

*Ethyl (4bS,7S,8S,9aR,10R)-7,8-dihydroxy-1-methyl-8-((((S)-1-(naphthalen-1-yl)ethyl)amino)methyl)-4b,6,7,8,9,10-hexahydro-5H-7,9a-methanobenzo[a]azulene-10-carboxylate(* **13*)***

Prepared with (*S*)-(−)-1-(1-naphthyl)ethylamine. Yield: 93%; white crystals; m.p.: 70–71 °C; [*α*${]}_{D}^{20}$ = −59.5 (c 0.11, MeOH); ^1^H NMR (DMSO-*d*_6_, 500 MHz): δ = 8.18 (1H, d, *J* = 7.9 Hz), 7.91 (1H, d, *J* = 7.6 Hz), 7.78 (1H, d, *J* = 8.1 Hz), 7.59 (1H, d, *J* = 6.8 Hz) 7.52–7.46 (3H, m), 7.08 (1H, t, *J* = 7.4 Hz), 6.95 (1H, d, *J* = 7.5 Hz), 6.87 (1H, d, *J* = 7.2 Hz), 4.51 (2H, d, *J* = 5.6 Hz), 4.27–4.11 (2H, m), 3.92 (2H, s), 2.72 (1H, dd, *J* = 5.0, 11.8 Hz), 2.60–2.46 (3H, m), 2.15–2.09 (1H, m), 2.08 (3H, s), 1.65–1.56 (2H, m), 1.48- 1.36 (4H, m), 1.34 (3H, d, *J* = 6.8 Hz), 1.27 (3H, t, *J* = 7.3 Hz); ^13^C NMR and JMOD (125 MHz, DMSO-*d_6_*): δ = 14.8, 19.9, 22.3, 23.9, 33.7, 39.2, 46.0, 50.5, 52.0, 53.5, 53.7, 54.9, 60.5, 77.4, 79.9, 120.1, 123.0, 123.5, 125.8, 126.1, 126.3, 127.2, 127.4, 128.8, 129.1, 131.3, 133.9, 134.7, 139.0, 142.2, 145.2, 171.3; HR-MS (ESI): *m/z* calcd for C_32_H_38_NO_4_ [M + H]^+^: 500.2723; found: 500.2801.

*Ethyl (7S,8S,9aR,10R)-7,8-dihydroxy-1-methyl-8-((((R)-1-(naphthalen-1-yl)ethyl)amino)methyl)-4b,6,7,8,9,10-hexahydro-5H-7,9a-methanobenzo[a]azulene-10-carboxylate (* **14** *)*

Prepared with (*R*)-(+)-1-(1-Naphthyl) ethylamine. Yield: 86%; white powder; m.p.: 73–76 °C, [*α*${]}_{D}^{20}$ = −123.3 (c 0.11, MeOH); ^1^H NMR (DMSO-*d_6_*, 500 MHz): δH 8.22 (1H, d, *J* = 7.9 Hz), 7.90 (1H, d, *J* = 7.6 Hz), 7.76 (1H, d, *J* = 8.1 Hz), 7.59 (1H, d, *J* = 7.1 Hz), 7.50–7.43 (2H, m), 7.38 (1H, t, *J* = 7.6 Hz), 7.11 (1H, t, *J* = 7.3 Hz), 6.97 (1H, d, *J* = 7.5 Hz), 6.84 (1H, d, *J* = 7.3 Hz), 4.57–4.47 (2H, m), 4.30–4.13 (2H, m), 3.92 (1H, s), 3.85 (1H, s), 2.69 (1H, dd, *J* = 4.5, 11.3 Hz), 2.57–2.51 (2H, m), 2.43–2.37 (1H, m), 2.09 (3H, s), 2.05–1.97 (1H, m), 1.66–1.58 (1H, td, *J* = 6.1, 13.5 Hz), 1.48–1.38 (4H, m), 1.36 (3H, d, *J* = 6.4 Hz), 1.28 (3H, t, *J* = 6.8 Hz), 1.25–1.18 (1H, m), ^13^C NMR and JMOD (125 MHz, DMSO-*d_6_*): δ = 14.8, 19.9, 22.2, 24.3, 33.7, 39.0, 46.0, 50.5, 52.0, 53.5, 54.2, 54.9, 60.5, 77.6, 79.9, 119.9, 123.0, 123.4, 125.7, 125.9, 126.1, 127.2, 127.4, 128.8, 129.1, 131.4, 134.0, 134.7, 139.0, 141.8, 145.3, 171.3; HR-MS (ESI): *m*/*z* calcd for C_32_H_38_NO_4_ [M + H]^+^: 500.2723; found: 500.2792.

*Ethyl (4bS,7S,8S,9aR,10R)-7,8-dihydroxy-1-methyl-8-(((naphthalen-1-ylmethyl)amino)methyl)-4b,6,7,8,9,10-hexahydro-5H-7,9a-methanobenzo[a]azulene-10-carboxylate (* **15** *)*

Prepared with 1-naphtylmethylamine. Yield: 75%; yellowish powder; m.p.: 71–74 °C, [*α*${]}_{D}^{20}$ = −44.2 (c 0.1, MeOH); ^1^H NMR (DMSO-*d_6_*, 500 MHz): δH 8.12 (1H, d, *J* = 7.9 Hz), 7.93–7.88 (1H, m), 7.80 (1H, d, *J* = 8.1 Hz), 7.53–7.45 (3H, m), 7.44–7.39 (1H, m), 7.09 (1H, t, *J* = 7.5 Hz), 6.95 (1H, d, *J* = 7.6 Hz), 6.90 (1H, d, *J* = 7.6 Hz), 4.50 (1H, s), 4.23–4.10 (4H, m), 3.92 (2H, s), 2.79–2.70 (2H, m), 2.64 (1H, d, *J* = 11.1 Hz), 2.54 (1H, d, *J* = 10.1 Hz), 2.19–2.13 (1H, m), 2.07 (3H, s), 1.71–1.61 (2H, m), 1.53–1.32 (4H, m), 1.25 (3H, t, *J* = 7.14 Hz), ^13^C NMR and JMOD (125 MHz, DMSO-*d_6_*): δ = 14.8, 19.9, 22.3, 33.8, 39.2, 46.0, 51.7, 52.0, 52.4, 53.5, 54.9, 60.5, 77.3, 80.1, 120.0, 124.3, 125.8, 126.0, 126.2, 126.3, 127.4, 127.7, 128.8, 128.8, 131.9, 133.8, 134.7, 136.8, 139.0, 145.2, 171.2; HR-MS (ESI): *m/z* calcd for C_31_H_36_NO_4_ [M + H]^+^: 486.2566; found: 486.2638.

*Ethyl (4bS,7S,8S,9aR,10R)-8-((benzyl(methyl)amino) methyl)-7,8-dihydroxy-1-methyl-4b,6,7,8,9,10-hexahydro-5H-7,9a-methanobenzo[a]azulene-10-carboxylate (* **16** *)*

Prepared with *N*-benzylmethylamine. Yield: 92%; white crystals; m.p.: 94–95 °C; [*α*${]}_{D}^{20}$ = −56.4 (c 0.13, MeOH); ^1^H NMR (DMSO-*d_6_*, 500 MHz): δ = 7.28–7.22 (5H, m), 7.11 (1H, t, *J* = 7.4 Hz), 6.96 (1H, d, *J* = 7.5 Hz), 6.86 (1H, d, *J* = 7.2 Hz), 4.38 (1H, s), 4.24–4.14 (3H, m), 3.92 (1H, s), 3.64 (1H, d, *J* = 13.0 Hz), 3.39 (1H, d, *J* = 13.3 Hz), 2.70 (1H, dd, *J* = 5.2, 12.1 Hz), 2.52–2.48 (1H, m), 2.44 (2H, s), 2.14 (3H, s), 2.07 (3H, s), 2.07–2.02 (1H, m) 1.65 (1H, ddd, *J* = 6.6, 13.1, 14.1 Hz), 1.58 (1H, dd, *J* = 5.6, 12.8 Hz), 1.52–1.39 (3H, m), 1.26 (3H, t, *J* = 7.2 Hz), 1.24–1.20 (1H, m); ^13^C NMR and JMOD (125 MHz, DMSO-*d_6_*): δ = 14.8, 19.9, 21.9, 33.4, 40.7, 43.8, 46.2, 52.0, 53.4, 54.9, 59.0, 60.5, 63.3, 77.0, 80.2, 120.1, 127.3, 127.4, 128.6, 128.6, 128.8, 129.2, 129.2, 134.7, 139.0, 139.5, 145.2, 171.2; HR-MS (ESI): *m/z* calcd for C_28_H_36_NO_4_ [M + H]^+^: 450.2566; found: 450.2637.

*Ethyl (4bS,7S,8S,9aR,10R)-8-((dibenzylamino)methyl)-7,8-dihydroxy-1-methyl-4b,6,7,8,9,10-hexahydro-5H-7,9a-methanobenzo[a]azulene-10-carboxylate (* **17** *)*

Prepared with dibenzylamine. Yield: 95%; yellow crystals; m.p.: 101.4–103 °C, [*α*${]}_{D}^{20}$ = −37.5 (c 0.16 MeOH); ^1^H NMR (DMSO-*d_6_*, 500 MHz): δ = 7.23 (10H, s), 7.13 (1H, t, *J* = 7.5 Hz), 6.97 (1H, d, *J* = 7.5 Hz), 6.69 (1H, d, *J* = 7.3 Hz), 4.63 (1H, s), 4.23- 4.12 (2H, m), 3.96 (2H, d, *J* = 13.3 Hz), 3.87 (1H, s), 3.76 (1H, s), 3.51–3.41 (2H, m), 3.20 (2H, d, *J* = 13.3 Hz), 2.59–2.50 (3H, m), 2.35 (1H, d, *J* = 13.4 Hz), 2.09 (3H, s), 1.56–1.36 (5H, m), 1.24 (3H, t, *J* = 7.1 Hz); ^13^C NMR and JMOD (125 MHz, DMSO-*d_6_*): δ = 14.8, 19.9, 33.9, 38.9, 46.2, 52.0, 53.4, 54.6, 55.0, 59.5, 60.4, 60.7, 72.7, 78.8, 80.3, 120.3, 127.2, 128.6, 128.8, 129.1, 134.5, 138.9, 140.2, 145.0, 171.2; HR-MS (ESI): *m/z* calcd for C_34_H_40_NO_4_ [M + H]^+^ 526.2879; found: 526.2951.

*Ethyl (4bS,7S,8S,9aR,10R)-8-((diethylamino)methyl)-7,8-dihydroxy-1-methyl-4b,6,7,8,9,10-hexahydro-5H-7,9a-methanobenzo[a]azulene-10-carboxylate (* **18** *)*

Prepared with diethylamine. Yield: 50%; yellow oil; [*α*${]}_{D}^{20}$ = −60.0 (c 0.11, MeOH), ^1^H NMR (DMSO-*d_6_*, 500 MHz): δ = 7.10 (1H, t, *J* = 7.4 Hz), 6.96 (1H, d, *J* = 7.3 Hz), 6.92 (1H, d, *J* = 6.8 Hz), 4.24–4.13 (2H, m), 3.95 (1H, s), 2.74 (1H, dd, *J* = 5.0, 12.4 Hz), 2.66–2.57 (4H, m), 2.53–2.49 (2H, m), 2.48 (1H, d, *J* = 10.5 Hz), 2.23–2.16 (1H, m), 2.07 (3H, s), 1.73–1.63 (2H, m), 1.56–1.36 (4H, m), 1.28–1.22 (3H, m), 0.95 (6H, t, *J* = 7.5 Hz); ^13^C NMR and JMOD (125 MHz, DMSO-*d_6_*): δ = 11.2, 11.2, 14.8, 19.8, 22.2, 32.7, 41.6, 41.9, 46.0, 47.4, 52.0, 53.5, 54.7, 54.8, 60.5, 75.2, 80.1, 120.1, 127.5, 128.9, 134.7, 138.9, 145.1, 171.3; HR-MS (ESI): *m/z* calcd for C_24_H_36_NO_4_ [M + H]^+^: 402.2566; found: 402.2632.

*Ethyl (4bS,7S,8S,9aR,10R)-7,8-dihydroxy-8-((isopropylamino)methyl)-1-methyl-4b,6,7,8,9,10-hexahydro-5H-7,9a-methanobenzo[a]azulene-10-carboxylate (* **19** *)*

Prepared with isopropylamine. Yield: 80%; white crystals, m.p.: 102–103 °C, [*α*${]}_{D}^{20}$ = −64.7 (c 0.11 MeOH), ^1^H NMR (DMSO-*d_6_*, 500 MHz): δ = 7.09 (1H, t, *J* = 7.4 Hz), 6.95 (1H, d, *J* = 7.3 Hz), 6.90 (1H, d, *J* = 7.3 Hz), 4.27–4.11 (2H, m), 3.92 (1H, s), 2.74 (1H, dd, *J* = 5.1, 12.0 Hz), 2.66–2.61 (1H, m), 2.60–2.52 (2H, m), 2.52–2.47 (1H, m), 2.23–2.13 (1H, m), 2.07 (3H, s), 1.74–1.67 (2H, m), 1.51–1.45 (2H, m), 1.40 (1H, d, *J* = 15.0 Hz), 1.32–1.24 (4H, m), 0.95 (3H, d, *J* = 6.3 Hz), 0.93 (3H, d, *J* = 6.3 Hz); ^13^C NMR and JMOD (125 MHz, DMSO-*d_6_*): δ = 14.8, 19.9, 22.4, 23.2, 23.5, 33.8, 39.3, 46.1, 49.0, 49.8, 52.1, 53.6, 54.9, 60.4, 77.0, 80.1, 120.0, 127.4, 128.8, 134.7, 139.0, 145.3, 171.3; HR-MS (ESI): *m/z* calcd for C_23_H_34_NO_4_ [M + H]^+^: 388.2410; found: 388.2479.

*Ethyl (4bS,7S,8S,9aR,10R)-7,8-dihydroxy-1-methyl-8-((prop-2-yn-1-ylamino)methyl)-4b,6,7,8,9,10-hexahydro-5H-7,9a-methanobenzo[a]azulene-10-carboxylate (* **20** *)*

Prepared with propagylamine. Yield: 86%; white crystals; m.p.: 111–112 °C, [*α*${]}_{D}^{20}$ = −86.7 (c 0.14, MeOH); ^1^H NMR (DMSO-*d_6_*, 500 MHz): δ = 7.09 (1H, t, *J* = 7.4 Hz), 6.95 (1H, d, *J* = 7.3 Hz), 6.90 (1H, d, *J* = 7.3 Hz), 4.56 (1H, s), 4.26–4.11 (2H, m), 3.93 (1H, s), 3.78 (1H, s), 3.37–3.32 (2H, m), 3.24 (1H, dd, *J* = 3.6, 17.3 Hz), 3.02 (1H, t, *J* = 2.3 Hz), 2.74 (1H, dd, *J* = 5.7, 11.6 Hz), 2.70 (1H, d, *J* = 11.4 Hz), 2.54 (1H, d, *J* = 9.6 Hz), 2.5–2.46 (1H, m), 2.23–2.18 (1H, m), 2.07 (3H, s), 1.74–1.68 (1H, m), 1.54–1.44 (2H, m), 1.35 (1H, d, *J* = 14.4 Hz), 1.30 (1H, d, *J* = 14.6 Hz), 1.26 (3H, t, *J* = 6.9 Hz); ^13^C NMR and JMOD (125 MHz, DMSO-*d_6_*): δ = 14.8, 19.9, 22.3, 33.7, 38.4, 39.2, 46.0, 51.1, 52.0, 53.5, 54.9, 60.5, 74.0, 77.3, 80.0, 83.5, 120.0, 127.4, 128.8, 134.7, 139.0, 145.2, 171.2. HR-MS (ESI): *m/z* calcd for C_23_H_30_NO_4_ [M + H]^+^: 384.2097; found: 384.2168.

*Ethyl (4bS,7S,8S,9aR,10R)-7,8-dihydroxy-1-methyl-8-(piperidin-1-ylmethyl)-4b,6,7,8,9,10-hexahydro-5H-7,9a-methanobenzo[a]azulene-10-carboxylate (* **21** *)*

Prepared with piperidine. Yield: 80%; orange crystals; m.p.: 84–86 °C, [*α*${]}_{D}^{20}$ = −52.9 (c 0.13 MeOH); ^1^H NMR (CDCl_3_, 500 MHz): δ = 7.14 (1H, t, *J* = 7.4 Hz), 7.01 (1H, d, *J* = 7.4 Hz), 6.91 (1H, d, *J* = 7.4 Hz), 4.36–4.24 (2H, m), 3.98 (1H, s), 3.37–3.25 (3H, m), 2.96 (1H, d, *J* = 13.0 Hz), 2.81 (1H, dd, *J* = 5.3, 11.8 Hz), 2.64 (1H, d, *J* = 10.5 Hz), 2.40–2.34 (1H, m), 2.17 (3H, s), 2.04–1.75 (9H. m), 1.70 (1H, d, *J* = 10.4 Hz), 1.56 (3H, d, *J* = 14.7 Hz), 1.50–1.39 (1H, m), 1.36 (3H, t, *J* = 7.0 Hz); ^13^C NMR and JMOD (125 MHz, CDCl_3_): δ = 14.5, 19.8, 21.7, 22.5, 22.6, 23.4, 23.4, 32.0, 41.9, 44.0, 45.9, 51.9, 54.2, 54.7, 60.2, 61.1, 74.1, 80.8, 120.0, 127.6, 129.3, 135.1, 138.1, 143.6, 171.3; HR-MS (ESI): *m/z* calcd for C_25_H_36_NO_4_ [M + H]^+^: 414.2566; found: 414.2633.

*Ethyl (4bS,7S,8S,9aR,10R)-7,8-dihydroxy-1-methyl-8-((4-methylpiperazin-1-yl)methyl)-4b,6,7,8,9,10-hexahydro-5H-7,9a-methanobenzo[a]azulene-10-carboxylate (* **22** *)*

Prepared with *N*-methyl piperazine. Yield: 55%; Brownish crystals, m.p.: 42–45 °C, [*α*${]}_{D}^{20}$ = −80.8 (c 0.12 MeOH); ^1^H NMR (CDCl_3_, 500 MHz): δ = 7.13 (1H, t, *J* = 7.4 Hz), 7.00 (1H, d, *J* = 7.6 Hz), 6.91 (1H, d, *J* = 7.3 Hz), 4.36–4.24 (2H, m), 3.96 (1H, s), 2.77 (1H, dd, *J* = 5.2, 12.1 Hz), 2.70–2.57 (3H, m), 2.52–2.26 (9H, m), 2.24 (3H, s), 2.17 (3H, s), 1.93–1.83 (1H, m), 1.75–1.64 (3H, m), 1.60–1.51 (2H, m), 1.35 (3H, t, *J* = 7.1 Hz); ^13^C NMR and JMOD (125 MHz, CDCl_3_): δ = 14.5, 19.9, 22.3, 31.2, 42.7, 45.9, 46.3, 52.3, 53.6, 53.7, 53.7, 55.0, 55.1, 59.5, 60.7, 74.6, 79.9, 79.9, 119.7, 127.3, 129.0, 135.0, 138.4, 144.5, 171.4; HR-MS (ESI): *m/z* calcd for C_25_H_37_N_2_O_4_ [M + H]^+^: 429.2675; Found: 429.2742.

*Ethyl (4bS,7S,8S,9aR,10R)-7,8-dihydroxy-1-methyl-8-(morpholinomethyl)-4b,6,7,8,9,10-hexahydro-5H-7,9a-methanobenzo[a]azulene-10-carboxylate (* **23** *)*

Prepared with morpholine. Yield: 80%; yellow oil; [*α*${]}_{D}^{20}$ = −79.5 (c 0.13 MeOH); ^1^H NMR (DMSO-*d_6_*, 500 MHz): δ = 7.11 (1H, t, *J* = 7.5 Hz), 6.96 (1H, d, *J* = 7.4 Hz), 6.92 (1H, d, *J* = 7.5 Hz), 4.25–4.16 (3H, m), 3.95 (1H, s), 3.54 (4H, s), 2.75 (1H, dd, *J* = 5.0, 12.0 Hz), 2.59–2.49 (3H, m), 2.48–2.34 (4H, m), 2.23–2.17 (1H, m), 2.09 (3H, s), 1.77–1.63 (2H, m), 1.54–1.38 (4H, m), 1.27 (3H, t, *J* = 6.99 Hz); ^13^C NMR and JMOD (125 MHz, DMSO-*d_6_*): δ = 14.8, 19.8, 22.2, 33.3, 40.9, 46.0, 52.0, 53.6, 54.8, 54.8, 54.9, 60.5, 60.6, 66.5, 66.5, 76.5, 80.2, 120.1, 127.5, 128.9, 134.7, 139.0, 145.2, 171.2; HR-MS (ESI): *m/z* calcd for C_24_H_34_NO_5_ [M + H]^+^: 416.2359; found: 416.2424.

*Ethyl (7S,8S,9aR,10S)-7,8-dihydroxy-1-methyl-8-((((S)-1-(naphthalen-2-yl)ethyl)amino)methyl)-4b,6,7,8,9,10-hexahydro-5H-7,9a-methanobenzo[a]azulene-10-carboxylate (* **35*)***

Prepared with (*S*)-(-)-1-(2-Naphthyl)ethylamine. Yield: 86%; white powder; m.p.: 56–59 °C, [*α*${]}_{D}^{20}$ = −210.0 (c 0.10, MeOH); ^1^H NMR (DMSO-*d_6_*, 500 MHz): δ = 7.88–7.80 (3H, m), 7.71 (1H, s), 7.50–7.44 (3H, m), 7.08 (1H, t, *J* = 7.5 Hz), 6.94 (1H, d, *J* = 7.4 Hz), 6.86 (1H, d, *J* = 7.4 Hz), 4.39 (1H, s), 4.08- 4.02 (3H, m), 3.78 (1H, q, *J* = 6.8 Hz), 3.60 (1H, s), 3.09 (1H, dd, *J* = 5.3, 12.1 Hz), 2.45–2.38 (3H, m), 2.14 (3H, s), 2.08–2.01 (1H, m), 1.66–1.52 (3H, m), 1.44 (1H, d, *J* = 14.6 Hz), 1.29 (4H, d, *J* = 6.8 Hz), 1.26–1.22 (1H, m), 1.18 (3H, t, *J* = 7.3 Hz), ^13^C NMR and JMOD (125 MHz, DMSO-*d_6_*): δ = 14.7, 18.7, 22.4, 24.3, 33.8, 44.5, 47.1, 50.1, 50.2, 52.6, 57.1, 58.5, 60.4, 77.8, 80.2, 120.8, 125.2, 125.3, 125.8, 126.3, 127.7, 127.8, 127.9, 128.0, 128.3, 132.7, 133.4, 134.2, 140.4, 144.1, 145.7, 171.7; HR-MS (ESI): *m/z* calcd for C_32_H_38_NO_4_ [M + H]^+^: 500.2723; found: 500.2791.

*Ethyl (7S,8S,9aR,10S)-7,8-dihydroxy-1-methyl-8-((((S)-1-(naphthalen-1-yl)ethyl)amino)methyl)-4b,6,7,8,9,10-hexahydro-5H-7,9a-methanobenzo[a]azulene-10-carboxylate (* **36** *)*

Prepared with (*S*)-(-)-1-(1-Naphthyl)ethylamine. Yield: 60%; white powder; m.p.: 80–83 °C, [*α*${]}_{D}^{20}$ = −153.6 (c 0.12, MeOH); ^1^H NMR (DMSO-*d_6_*, 500 MHz): δH 8.17 (1H, d, *J* = 7.9 Hz), 7.93–7.89 (1H, m), 7.78 (1H, d, *J* = 8.1 Hz), 7.59 (1H, d, *J* = 7.2 Hz), 7.51–7.46 (3H, m), 7.10 (1H, t, *J* = 7.4 Hz), 6.95 (1H, d, *J* = 7.5 Hz), 6.89 (1H, d, *J* = 7.3 Hz), 4.54–4.47 (2H, m), 4.14–4.04 (3H, m), 3.61 (1H, s), 3.10 (1H, dd, *J* = 5.1, 12.0 Hz), 2.56–2.42 (3H, m), 2.14 (3H, s), 2.12–2.06 (1H, m), 1.67–1.54 (3H, m), 1.44 (1H, d, *J* = 14.6 Hz), 1.35–1.27 (5H, m), 1.19 (3H, t, *J* = 7.0 Hz); ^13^C NMR and JMOD (125 MHz, DMSO-*d_6_*): δ = 14.7, 18.7, 22.4, 23.9, 33.9, 44.6, 46.9, 50.2, 50.3, 52.6, 53.7, 57.0, 60.5, 77.9, 80.2, 120.8, 122.9, 123.4, 125.8, 126.1, 126.3, 127.2, 127.8, 128.0, 129.1, 131.3, 133.9, 134.2, 140.4, 142.1, 145.7, 171.7; HR-MS (ESI): *m/z* calcd for C_32_H_38_NO_4_ [M + H]^+^: 500.2723; found: 500.2792.

*Ethyl (7S,8S,9aR,10S)-7,8-dihydroxy-1-methyl-8-((((R)-1-(naphthalen-1-yl)ethyl)amino)methyl)-4b,6,7,8,9,10-hexahydro-5H-7,9a-methanobenzo[a]azulene-10-carboxylate (* **37*)***

Prepared with (*R*)-(+)-1-(1-Naphthyl) ethylamine. Yield: 87%; white powder; m.p.: 69–72 °C, [*α*${]}_{D}^{20}$ = −177.1 (c 0.10, MeOH); ^1^H NMR (DMSO-*d_6_*, 500 MHz): δ = 8.23 (1H, d, *J* = 8.5 Hz), 7.90 (1H, d, *J* = 7.9 Hz), 7.76 (1H, d, *J* = 8.1 Hz), 7.56 (1H, d, *J* = 7.0 Hz), 7.51–7.40 (2H, m), 7.35 (1H, t, *J* = 7.5 Hz), 7.15 (1H, t, *J* = 7.4 Hz), 6.98 (1H, d, *J* = 7.5 Hz), 6.86 (1H, d, *J* = 7.3 Hz), 4.58–4.47 (2H, m), 4.13–4.03 (3H, m), 3.62 (1H, s), 3.09 (1H, dd, *J* = 5.3, 12.1 Hz), 2.56–2.51 (1H, m), 2.46 (1H, d, *J* = 10.1 Hz), 2.38 (1H, d, *J* = 11.3 Hz), 2.17 (3H, s), 2.04–1.94 (1H, m), 1.69 (1H, d, *J* = 14.6 Hz), 1.65–1.55 (1H, m), 1.45 (2H, d, *J* = 15.1 Hz), 1.36 (3H, d, *J* = 6.6 Hz), 1.31 (1H, d, *J* = 10.1 Hz), 1.19 (3H, t, *J* = 6.8 Hz), 1.16–1.07 (1H, m); ^13^C NMR and JMOD (125 MHz, DMSO-*d_6_*): δ = 14.7, 18.7, 22.2, 24.2, 33.9, 44.5, 46.7, 50.1, 50.2, 52.5, 54.3, 57.1, 60.4, 78.1, 80.2, 120.6, 123.1, 123.5, 125.7, 125.8, 126.1, 127.2, 127.8, 127.9, 129.0, 131.3, 134.0, 134.2, 140.4, 141.8, 145.7, 171.7; HR-MS (ESI): *m/z* calcd for C_32_H_38_NO_4_ [M + H]^+^: 500.2723; found: 500.2792.

*Ethyl (7S,8S,9aR,10S)-7,8-dihydroxy-1-methyl-8-(((naphthalen-1-ylmethyl)amino)methyl)-4b,6,7,8,9,10-hexahydro-5H-7,9a-methanobenzo[a]azulene-10-carboxylate (* **38** *)*

Prepared with 1-naphtylmethylamine. Yield: 78%; white powder; m.p.: 61–65 °C, [*α*${]}_{D}^{20}$ = −130.6 (c 0.11, MeOH); ^1^H NMR (DMSO-*d_6_*, 500 MHz): δ = 8.13–8.10 (1H, m), 7.92–7.88 (1H, m), 7.79 (1H, d, *J* = 8.1 Hz), 7.51–7.43 (3H, m), 7.4 (1H, t, *J* = 7.19 Hz), 7.11 (1H, t, *J* = 7.4 Hz), 6.95 (1H, d, *J* = 7.6 Hz), 6.91 (1H, d, *J* = 7.35 Hz), 4.50 (1H, s), 4.14–4.04 (5H, m), 3.60 (1H, s), 3.11 (1H, dd, *J* = 5.2, 11.9 Hz), 2.73 (1H, d, *J* = 11.6 Hz), 2.65–2.58 (1H, m), 2.43 (1H, d, *J* = 9.9 Hz), 2.13 (4H, m), 1.67–1.59 (3H, m), 1.44–1.34 (2H, m), 1.29 (1H, d, *J* = 9.2 Hz), 1.18 (3H, t, *J* = 7.2 Hz); ^13^C NMR and JMOD (125 MHz, DMSO-*d_6_*): δ = 14.7, 18.7, 22.4, 34.0, 44.5, 46.9, 50.2, 51.7, 52.2, 52.6, 57.0, 60.5, 77.8, 80.3, 120.8, 124.3, 125.8, 126.0, 126.2, 126.3, 127.6, 127.8, 128.0, 128.8, 131.8, 133.8, 134.2, 136.8, 140.4, 145.7, 171.7; HR-MS (ESI): *m/z* calcd for C_31_H_36_NO_4_ [M + H]^+^: 486.2566; found: 486.2634.

*Ethyl (4b’S,7’S,9a’R,10’R)-3-benzyl-7’-hydroxy-1’-methyl-4b’,6’,7’,10’-tetrahydro-5’H,9’H-spiro[oxazolidine-5,8’-[7,9a]methanobenzo[a]azulene]-10’-carboxylate (* **24** *)*

To the solution of aminodiol **4** (200 mg, 0.45 mmol) in Et_2_O (5.0 mL), the aqueous formaldehyde 35% (10.0 mL) was added. After being stirred for 1.5 h at room temperature (as monitored by TLC), the mixture was made alkaline with a saturated aqueous solution of NaHCO_3_ (20 mL) then extracted with Et_2_O (3 × 30 mL). The organic layer was then dried over Na_2_SO_4_ and subsequently evaporated in reduced pressure. The crude product was purified by flash column chromatography on silica gel (eluted with CHCl_3_:MeOH = 19:1).

Yield: 60%; white crystals; m.p.: 125–126 °C; [*α*${]}_{D}^{20}$ = −75.4 (c 0.10, MeOH); ^1^H NMR (DMSO-*d_6_*, 500 MHz): δ = 7.32–7.19 (5H, m), 7.09 (1H, t, *J* = 7.4 Hz), 6.96 (1H, d, *J* = 7.6 Hz), 6.90 (1H, d, *J* = 7.3 Hz), 4.48 (1H, s), 4.25 (1H, d, *J* = 4.0 Hz), 4.21 (2H, q, *J* = 6.8 Hz), 4.08 (1H, d, *J* = 4.4 Hz), 3.97 (1H, s), 3.60 (2H, s), 2.85 (2H, q, *J* = 11.6 Hz), 2.73 (1H, dd, *J* = 4.3, 12.0 Hz), 2.38 (1H, d, *J* = 10.2 Hz), 2.20–2.12 (1H, m), 2.08 (3H, s), 1.72 (1H, d, *J* = 15.0 Hz), 1.63 (3H, d, *J* = 9.3 Hz), 1.52 (1H, d, *J* = 15.0 Hz), 1.28 (4H, t, *J* = 7.0 Hz); ^13^C NMR and JMOD (125 MHz, DMSO-*d_6_*): δ = 14.8, 19.8, 22.1, 33.8, 43.7, 46.9, 51.8, 53.5, 54.8, 56.2, 57.9, 60.5, 79.9, 85.5, 86.3, 120.2, 127.3, 127.5, 128.7, 128.9, 134.7, 138.9, 139.8, 144.9, 171.3; HR-MS (ESI): *m/z* calcd for C_28_H_34_NO_4_ [M + H]^+^: 448.2410; found: 448.2485.

### 4.4. General Procedure for Ring-Opening of Epoxide with Azoles

To the solution of epoxide **3** (100 mg, 0.3 mmol) in dry DMF (5.0 mL), K_2_CO_3_ (124 mg, 0.90 mmol, 3 equ.) and appropriate azoles (0.60 mmol, 2 equ.) were added. After treating under reflux conditions at 70–80 °C for 24 h, the reaction mixture was cooled to room temperature, then diluted with 5.0 mL water and subsequently extracted with EtOAc (3 × 10 mL). The organic phase was washed with brine solution (3 × 10 mL), dried over sodium sulfate and then evaporated. The crude product was purified by column chromatography on silica gel (eluted with CHCl_3_:MeOH = 19:1).

*Ethyl (4bS,7S,8S,9aR,10R)-8-((1H-imidazol-1-yl)methyl)-7,8-dihydroxy-1-methyl-4b,6,7,8,9,10-hexahydro-5H-7,9a-methanobenzo[a]azulene-10-carboxylate (* **26** *)*

Prepared with imidazole. Yield: 60%; white crystals; m.p.: 101–103 °C; [*α*${]}_{D}^{20}$ = −57.9 (c 0.13, MeOH); ^1^H NMR (DMSO-*d_6_*, 500 MHz): δ = 7.56 (1H, s), 7.15–7.10 (2H, m), 6.98–6.93 (2H, m), 6.73 (1H, s), 4.99 (1H, s), 4.32 (1H, s), 4.11–4.03 (3H, m), 3.82 (1H, d, *J* = 14.2 Hz), 3.56 (1H, s), 3.16 (1H, dd, *J* = 5.3, 12.1 Hz), 2.52–2.47 (1H, m), 2.27–2.20 (1H, m), 2.10 (3H, s), 1.82–1.75 (2H, m), 1.63–1.49 (2H, m), 1.36 (1H, d, *J* = 10.1 Hz), 1.18 (3H, t, *J* = 7.1 Hz), 1.10 (1H, d, *J* = 14.6 Hz); ^13^C NMR and JMOD (125 MHz, DMSO-*d_6_*): δ = 14.7, 18.7, 22.2, 33.8, 44.4, 44.8, 49.4, 50.3, 52.4, 56.8, 60.5, 78.0, 80.0, 120.9, 121.4, 127.6, 127.8, 128.1, 134.3, 138.3, 140.3, 145.5, 171.7; HR-MS (ESI): *m/z* calcd for C_23_H_29_N_2_O_4_ [M + H]^+^: 397.2049; found: 397.2116.

*Ethyl (4bS,7S,8S,9aR,10R)-8-((1H-benzo[d]imidazol-1-yl)methyl)-7,8-dihydroxy-1-methyl-4b,6,7,8,9,10-hexahydro-5H-7,9a-methanobenzo[a]azulene-10-carboxylate (* **27** *)*

Prepared with benzimidazole. Yield: 66%; white crystals; m.p.: 221–222 °C; [*α*${]}_{D}^{20}$ = −97.2 (c 0.11, MeOH); ^1^H NMR (DMSO-*d_6_*, 500 MHz): δ = 8.22 (1H, s), 7.58–7.55 (1H, m), 7.46–7.43 (1H, m), 7.16–7.07 (3H, m), 7.01 (1H, d, *J* = 7.3 Hz), 6.92 (1H, d, *J* = 7.5 Hz), 5.12 (1H, s), 4.47 (1H, s), 4.39 (1H, d, *J* = 14.5 Hz), 4.12–4.06 (3H, m), 3.51 (1H, s), 3.22–3.16 (1H, m), 2.54–2.49 (1H, m), 2.41–2.33 (1H, m), 2.06 (3H, s), 2.02–1.96 (1H, m), 1.85 (1H, td, *J* = 5.6, 13.1 Hz), 1.78–1.67 (1H, m), 1.58 (1H, d, *J* = 13.9 Hz), 1.40 (1H, d, *J* = 10.1 Hz), 1.18 (3H, t, *J* = 7.1 Hz), 1.12–1.08 (1H, m); ^13^C NMR and JMOD (125 MHz, DMSO-*d_6_*): δ = 14.7, 18.6, 22.6, 33.9, 44.2, 44.9, 47.3, 50.3, 52.5, 56.7, 60.5, 78.6, 80.2, 110.8, 119.6, 120.8, 121.4, 122.3, 127.8, 128.1, 134.3, 135.3, 140.2, 140.2, 143.3, 145.5, 171.6; HR-MS (ESI): *m/z* calcd for C_27_H_31_N_2_O_4_ [M + H]^+^: 447.2206; found: 447.2270.

*Ethyl (4bS,7S,8S,9aR,10R)-8-((1H-1,2,4-triazol-1-yl)methyl)-7,8-dihydroxy-1-methyl-4b,6,7,8,9,10-hexahydro-5H-7,9a-methanobenzo[a]azulene-10-carboxylate (* **28** *)*

Prepared with 1,2,4-triazole. Yield: 75%; white crystals; m.p.: 87–89 °C, [*α*${]}_{D}^{20}$ = −63.6 (c 0.13, MeOH); ^1^H NMR (DMSO-*d_6_*, 500 MHz): δ = 8.39 (1H, s), 7.78 (1H, s), 7.15–7.11 (1H, m), 6.97 (2H, t, *J* = 7.9 Hz), 5.02 (1H, s), 4.30 (1H, s), 4.21 (1H, d, *J* = 14.0 Hz), 4.14–4.02 (3H, m), 3.56 (1H, s), 3.17 (1H, dd, *J* = 5.4, 12.1 Hz), 2.48–2.44 (1H, m), 2.32–2.23 (1H, m), 2.11 (3H, s), 1.90–1.73 (3H, m), 1.58–1.46 (1H, m), 1.36 (1H, d, *J* = 10.1 Hz), 1.18 (3H, t, *J* = 6.9 Hz), 1.07 (1H, d, *J* = 15.0 Hz); ^13^C NMR and JMOD (125 MHz, DMSO-*d_6_*): δ = 14.5, 18.7, 22.4, 33.7, 44.2, 45.1, 50.2, 52.1, 52.5, 56.8, 60.5, 77.4, 79.8, 120.9, 127.9, 128.1, 134.3, 140.3, 145.5, 145.6, 150.9, 171.6; HR-MS (ESI): *m/z* calcd for C_22_H_28_N_3_O_4_ [M + H]^+^: 398.2002; found: 398.207.

*Ethyl (4bS,7S,8S,9aR,10R)-8-((1H-1,2,3-triazol-1-yl)methyl)-7,8-dihydroxy-1-methyl-4b,6,7,8,9,10-hexahydro-5H-7,9a-methanobenzo[a]azulene-10-carboxylate (* **29** *)*

Prepared with 1,2,3-triazole. Yield: 50%; white crystals; m.p: 90–92 °C; [*α*${]}_{D}^{20}$ = −52.5 (c 0.075, MeOH); ^1^H NMR (DMSO-*d_6_*, 500 MHz): δ = 8.01 (1H, s), 7.60 (1H, s), 7.12 (1H, t, *J* = 6.8 Hz), 6.99 (1H, d, *J* = 7.5 Hz), 6.95 (1H, d, *J* = 7.5 Hz), 5.07 (1H, s), 4.53 (1H, d, *J* = 13.9 Hz), 4.38 (1H, s), 4.20 (1H, d, *J* = 14.0 Hz), 4.11–4.04 (2H, m), 3.54 (1H, s), 3.18 (1H, dd, *J* = 5.2, 11.9 Hz), 2.49–2.46 (1H, m), 2.38–2.28 (1H, m), 2.10 (3H, s), 1.90–1.70 (3H, m), 1.61–1.50 (1H, m), 1.38 (1H, d, *J* = 10.2 Hz), 1.18 (3H, t, *J* = 7.0 Hz), 1.01 (1H, d, *J* = 14.8 Hz); ^13^C NMR and JMOD (125 MHz, DMSO-*d_6_*): δ = 14.7, 18.7, 22.4, 33.7, 44.3, 44.8, 50.2, 52.5, 52.6, 56.8, 60.5, 77.8, 80.0, 120.9, 126.7, 127.9, 128.1, 132.9, 134.4, 140.3, 145.5, 171.6; HR-MS (ESI): *m/z* calcd for C_22_H_28_N_3_O_4_ [M + H]^+^: 398.2002; Found: 398.2077.

*Ethyl (4bS,7S,8S,9aR,10R)-8-((1H-benzo[d][1,2,3]triazol-1-yl)methyl)-7,8-dihydroxy-1-methyl-4b,6,7,8,9,10-hexahydro-5H-7,9a-methanobenzo[a]azulene-10-carboxylate (* **30** *)*

Prepared with benzotriazole. Yield: 45%; white crystals; m.p: 103–104 °C; [*α*${]}_{D}^{20}$ = +12.4 (c 0.075, MeOH), ^1^H NMR (DMSO-*d_6_*, 500 MHz): δ = 7.93 (2H, dd, *J* = 2.2, 7.6 Hz), 7.47 (1H, t, *J* = 7.6 Hz), 7.33 (1H, t, *J* = 7.6 Hz), 7.16 (1H, t, *J* = 7.4 Hz), 7.03 (1H, d, *J* = 7.5 Hz), 6.96 (1H, d, *J* = 7.5 Hz), 5.11 (1H, s), 4.82 (1H, d, *J* = 14.5 Hz), 4.54 (1H, d, *J* = 14.5 Hz), 4.48 (1H, s), 4.10–4.01 (2H, m), 3.52 (1H, s), 3.22 (1H, dd, *J* = 6.1, 12.7 Hz), 2.48–2.45 (1H, m), 2.40–2.32 (1H, m), 2.09 (3H, s), 2.05–1.83 (3H, m), 1.76–1.65 (1H, m), 1.42 (1H, d, *J* = 10.2 Hz), 1.16 (3H, t, *J* = 6.8 Hz), 1.04 (1H, d, *J* = 15.0 Hz); ^13^C NMR and JMOD (125 MHz, DMSO-*d_6_*): δ = 14.7, 18.7, 22.5, 34.0, 44.3, 45.0, 50.3, 51.7, 52.5, 56.7, 60.5, 79.1, 80.2, 112.9, 119.0, 120.9, 123.9, 127.1, 127.9, 128.1, 134.4, 134.8, 140.3, 145.3, 145.5, 171.7; HR-MS (ESI): *m/z* calcd for C_26_H_30_N_3_O_4_ [M + H]^+^: 448.2158; Found: 448.2224.

### 4.5. General Procedure for Dipolar Cycloaddition

*Ethyl (4bS,7S,8S,9aR,10R)-8-((((1-benzyl-1H-1,2,3-triazol-4-yl)methyl)amino)methyl)-7,8-dihydroxy-1-methyl-4b,6,7,8,9,10-hexahydro-5H-7,9a-methanobenzo[a]azulene-10-carboxylate (* **31** *)*

To the solution of aminodiol **20** (100 mg, 0.26 mmol) in *t*-BuOH:H_2_O = 2:1, CuSO_4_.5H_2_O (2 mol%), sodium ascorbate (10 mol%) and benzyl azide (47 µL, 0.39 mmol, 1.5 equ.) were added. The mixture was stirred at 25 °C for 1 day (indicated by TLC) before *t*-BuOH was evaporated. The residue was dissolved in water (20 mL) extracted with EtOAc (3 × 30mL). The organic phase was washed with brine, dried over Na_2_SO_4_, and evaporated in reduced pressure. The crude product was purified by column chromatography on silica gel (eluted with CHCl_3_:MeOH = 9:1).

Yield: 40%; brown crystals; m.p.: 122–123 °C, [*α*${]}_{D}^{20}$ = −33.40 (c 0.10, MeOH); ^1^H NMR (DMSO-*d_6_*, 500 MHz): δ = 7.95 (1H, s), 7.39–7.31 (3H, m), 7.26 (2H, d, *J* = 7.9 Hz), 7.09 (1H, t, *J* = 7.3 Hz), 6.94 (1H, d, *J* = 7.5 Hz), 6.87 (1H, d, *J* = 7.5 Hz), 5.54 (2H, s), 4.53 (1H, s), 4.25–4.10 (2H, m), 3.92 (1H, s), 3.86 (1H, s), 3.72 (2H, s), 2.72 (1H, dd, *J* = 4.9, 11.7 Hz), 2.65–2.49 (3H, m), 2.15–2.08 (1H, m), 2.07 (3H, s), 1.71–1.57 (2H, m), 1.48–1.35 (3H, m), 1.31–1.23 (4H, m); ^13^C NMR and JMOD (125 MHz, DMSO-*d_6_*): δ = 14.8, 19.9, 22.2, 33.7, 39.2, 45.0, 45.9, 51.5, 52.0, 53.1, 53.5, 54.9, 60.5, 77.2, 80.0, 120.0, 123.2, 127.4, 128.2, 128.5, 128.5, 128.8, 129.1, 129.1, 134.7, 136.7, 139.0, 145.2, 146.9, 171.2; HR-MS (ESI): *m/z* calcd for C_30_H_37_N_4_O_4_ [M + H]^+^: 517.2737; Found: 517.2818.

*Ethyl (4bS,7S,8S,9aR,10R)-8-((((1-benzyl-1H-1,2,3-triazol-4-yl)methyl)amino)methyl)-7,8-dihydroxy-1-methyl-4b,6,7,8,9,10-hexahydro-5H-7,9a-methanobenzo[a]azulene-10-carboxylate (* **32** *)*

**First step:** To the solution of epoxide **3** (100 mg, 0.3 mmol) in EtOH:H_2_O = 8:2 (15 mL), NaN_3_ (39.0 mg, 0.6 mmol, 2 equ.) and NH_4_Cl (32.0 mg, 0.6mmol, 2 equ.) were added. After treating under reflux conditions for 48 h, the reaction mixture was cooled to room temperature, then ethanol was evaporated. The residue was dissolved in water (20 mL) and extracted with Et_2_O (3 × 20 mL). The organic phase was washed by brine solution, dried over sodium sulfate and then evaporated. The compound was purified by flash column chromatography on silica gel (eluted with CHCl_3_: MeOH = 19:1), then recrystallized in the diisopropyl ether: hexane = 1:1 to provide azido diol.

Yield: 65%; white crystals; m.p.: 108–109 °C; [*α*${]}_{D}^{20}$ = −8.1 (c 0.11, MeOH); ^1^H NMR (DMSO-*d_6_*,, 500 MHz): δ = 7.10 (1H, t, *J* = 7.5 Hz), 6.96 (1H, d, *J* = 7.6 Hz), 6.90 (1H, d, *J* = 7.3 Hz), 4.87 (1H, s), 4.23–4.16 (3H, m), 3.95 (1H, s), 3.28 (1H, d, *J* = 12.6 Hz), 3.18 (1H, d, *J* = 12.8 Hz), 2.76 (1H, dd, *J* = 5.4, 12.0 Hz), 2.54 (1H, d, *J* = 9.9 Hz), 2.21–2.15 (1H, m), 2.07 (3H, s), 1.73 (1H, td, *J* = 6.2, 13.1 Hz), 1.64 (1H, dd, *J* = 5.9, 13.3 Hz), 1.51–1.37 (3H, m), 1.31–1.25 (4H, m); ^13^C NMR and JMOD (125 MHz, DMSO-*d_6_*): δ = 14.8, 19.9, 22.1, 33.4, 38.4, 45.8, 51.8, 53.5, 54.3, 54.8, 60.6, 78.6, 79.7, 120.1, 127.5, 128.9, 134.7, 138.9, 145.0, 171.2; HR-MS (ESI): *m/z* calcd for C_20_H_25_N_3_O_4_Na [M + Na]^+^: 394.1743; Found: 394.1737.

**Second step:** CuSO_4_.5H_2_O (2.6 mg, 2 mol%), sodium ascorbate (10.5 mg, 10 mole%) and phenylacetylene (0.11 mL, 1.06 mmol, 2 equ.) were added to a solution of azido diol (200 mg, 0.53 mmol) in EtOH:H_2_O = 8:2. The mixture was stirred at room temperature for 1 day before EtOH was evaporated. The residue was dissolved in water (20 mL) and extracted with EtOAc (3 × 30mL). The organic phase was washed with brine, dried over Na_2_SO_4_, and then evaporated in low pressure. Then, the crude product was purified by column chromatography on silica gel (eluted with *n*-hexane: EtOAc = 1:2).

Yield: 98%; white crystals; m.p.: 191–192 °C; [*α*${]}_{D}^{20}$ = −17.3 (c 0.12, MeOH); ^1^H NMR (DMSO-*d_6_*, 500 MHz): δ = 8.40 (1H, s), 7.78 (2H, d, *J* = 7.5 Hz), 7.42 (2H, t, *J* = 7.6 Hz), 7.33–7.28 (1H, m), 7.14 (1H, t, *J* = 7.4 Hz), 6.98 (2H, dd, *J* = 7.6, 12.0 Hz), 5.15 (1H, s), 4.57 (1H, d, *J* = 14.3 Hz), 4.26 (1H, d, *J* = 14.2 Hz), 4.15 (1H, s), 4.13–4.08 (2H, m), 3.95 (1H, s), 2.83 (1H, dd, *J* = 5.2, 11.7 Hz), 2.57 (1H, d, *J* = 10.5 Hz), 2.38–2.31 (1H, m), 2.04 (3H, s), 1.95–1.80 (2H, m), 1.74–1.62 (1H, m), 1.59–1.49 (2H, m), 1.16 (3H, t, *J* = 7.3 Hz), 1.07 (1H, d, *J* = 15.5 Hz), ^13^C NMR and JMOD (125 MHz, DMSO-*d_6_*): δ = 14.7, 19.8, 22.3, 33.5, 37.4, 45.6, 51.9, 53.3, 53.6, 54.7, 60.5, 77.4, 79.7, 120.2, 123.3, 125.5, 125.5, 127.6, 128.1, 129.0, 129.3, 129.3, 131.3, 134.7, 138.9, 145.0, 145.9, 171.1; HR-MS (ESI): *m/z* calcd for C_28_H_32_N_3_O_4_ [M + H]^+^: 474.2315; Found: 474.2390.

### 4.6. Determination of Antiproliferative Effects of Aminodiol Derivatives

The growth-inhibitory effects of the gibberellic acid-based aminodiol derivatives were determined by a standard MTT (3-(4,5-dimethylthiazol-2-yl)-2,5-diphenyltetrazolium bromide) assay on a panel containing five cell lines, including Hela and Siha (cervical cancers); MDA-MB-231 and MCF-7 (breast cancers); and A2780 ovarian cancer cells. The most promising agents were additionally tested against NIH/3T3 mouse embryo fibroblast cells. All cell lines were purchased from the European Collection of Cell Cultures (Salisbury, UK) except SiHa, which was obtained from the American Tissue Culture Collection (Manassas, VA, USA) [48]. The cells were maintained in minimal essential medium supplemented with 10% fetal bovine serum, 1% non-essential amino acids, and 1% penicillin-streptomycin at 37 °C in a humidified atmosphere containing 5% CO_2_. All media and supplements were obtained from Lonza Group Ltd., (Basel, Switzerland). Cancer cells were seeded into 96-well plates (5000 cells/well), after an overnight incubation the test compound was added in 2 different concentrations (10 µM and 30 µM) and incubated for another 72 h under cell-culturing conditions. Then, 20 μL of 5 mg/mL MTT solution was added to each well and incubated for a further 4 h. The medium was removed, and the precipitated formazan crystals were dissolved in DMSO during 60 min of shaking at 37 °C. As the final step, the absorbance was measured at 545 nm by using a microplate reader (SPECTROStar Nano, BMG Labtech, Offenburg, Germany). Untreated cells were included as controls. In case of the most effective compounds (i.e., compounds eliciting higher than 50 or 85% at 10 or 30 μM, respectively), the assays were repeated with a set of dilutions (0.1–30.0 μM) to determine the IC_50_ values. These agents were additionally tested against NIH/3T3 fibroblasts to obtain results about their cancer selectivity. Two independent experiments were performed with five wells for each condition. Cisplatin (Ebewe GmbH, Unterach, Austria), a clinically used anticancer agent, was used as a positive control. Calculations were performed utilizing the GraphPad Prism 5.01 software (GraphPad Software Inc., San Diego, CA, USA).

### 4.7. Determination of Antioxidant Effects by Free Radical Scavenging (DPPH Assay)

The antioxidant effects of the aminodiol derivatives were tested on 1,1-diphenyl-2-picrylhydrazyl (DPPH) radicals according to Brand-Williams (1995) [55] and Sánchez-Moreno (2002) [56], with some modifications. A serial dilution of the tested compounds was made in absolute methanol and DPPH solution was added to each concentration in order to have a 0.1 mM concentration DPPH in each well of a 96-well plate. After a 30 min incubation period at room temperature (25 °C) the absorbances were measured at 517 nm by a microplate reader (SPECTROStar Nano, BMG Labtech, Offenburg, Germany). Trolox (3,4-Dihydro-6-hydroxy-2,5,7,8-tetramethyl-2*H*-1-benzopyran-2-carboxylic acid) was used as a positive control. All measurements were performed in duplicate with 5 parallels.

### 4.8. Docking Study

Anaplastic lymphoma kinase (ALK) crystal structure was obtained from PDB (protein data bank). ChemBioDraw Ultra 11.0 was used to draw the tested structures for the docking study. Docking study and in-Silico ADMET prediction were performed by Accelrys discovery studio 2.5 software.

#### 4.8.1. Preparation of the Crystal Structure of ALK

It is well known that the extracted crystal structure from PDB does not have hydrogen atoms, so hydrogen atoms must first be added by applying several force fields (CHARMm). Adding hydrogen atoms leads to steric hindrance and subsequently to a high-energy and unstable molecule, which should be minimized. Minimization of the complex energy was performed by using adopted basis minimization, aiming at finding the most stable structure with the least energy and reducing H-H interactions without affecting the basic protein skeleton atoms. Then, the active site was determined and 10 Å radius sphere surrounded [57].

#### 4.8.2. Docking Study (CDocker)

By using the CDocker method, all possible conformations of the compound in the protein active site could be generated. Then, the results can be evaluated by both the CDocker energy and the number of interactions between the ligand and the active site. This method requires preparing the crystal structure (as mentioned before) and preparing the designed compound by using Accelrys Discovery Studio protocol and applying a force field.

Before starting this study, it is important to emphasize that the method used here is valid by comparing the conformation of the reference compound with its conformations generated by the applied docking method, where RMSD (Root Mean Square Deviation) should not exceed 2 Å (Plot of Polar Surface Area (PSA) vs. LogP is given in Supplementary Materials Figure S96).

#### 4.8.3. In-Silico ADMET Analysis

ADMET properties of compounds **12**, **13**, and **14** were predicted by using ADMET descriptors in Accelrys Discovery studio 2.5. There are six mathematical models used to quantitatively predict properties of a set of compounds. These models contain: aqueous solubility (predict solubility in water at 25 °C, blood–brain barrier (BBB) penetration, cytochrome P450 (CYP450) 2D6 inhibition, human intestinal absorption (HIA), and plasma protein binding (PPB) [58]. An ADMET model was also generated to predict the human intestinal absorption (HIA) and blood–brain barrier (BBB) penetration of tested compounds. The model includes 95 and 99% confidence ellipses in the ADMET_PSA_2D and ADMET_ALogP98 plan. Table S2 containing in silico ADMET properties is given in Supplementary Materials Table S3.

## 5. Conclusions

In summary, starting from commercially available gibberellic acid, a new family of gibberellic acid-derived 3-amino-1,2-diols was designed and synthesized.

The resulting *N*-naphthylmethyl-substituted aminodiols exert marked antiproliferative action on a panel of human cancer cell lines. The in vitro pharmacological studies have clearly shown that the *N*-naphthylmethyl substituent on the aminodiol system seems to be essential for reliable antiproliferative activity.

Finally, molecular docking results exemplified that aminodiols **12**, **13**, and **14** could foster potent affinity by forming significant hydrogen and hydrophobic interactions with ALKwt (PDB ID code: 3AOX). Hence, *N*-naphthylmethyl-substituted aminodiols can be regarded as new potential drug candidates and further studies concerning molecular dynamics will be performed and reported in the future.

Literature revealed that allo-gibberic acid derivatives containing saturated linear amides or substituted benzyl esters showed strong anticancer activities in MTT assay toward a number of human cancer cell lines [27]. Therefore, in the next stage of our project, the modification of carbonyl group in *N*-naphthylmethyl-substituted aminodiols was performed to improve their antiproliferative activities on a panel of different cancer cell lines. For the optimized derivatives, additionally, docking studies and a molecular dynamics study will also be performed to gain insight into the dynamics of ligand interaction.

## Figures and Tables

**Table 1:** Chiral 3-amino-1,2-diol derivatives based on allo-gibberic acid.

Entry	Compound	R^1^	R^2^	Yield (%)
1	**4**	H	Phenylmethyl	70
2	**5**	H	(1*S*)-1-Phenylethyl	88
3	**6**	H	(1*R*)-1-Phenylethyl	72
4	**7**	H	(1*S*)-1-Phenyl-1-propyl	72
5	**8**	H	(1*R*)-1-Phenyl-1-propyl	72
6	**9**	H	(4-Fluorophenyl)methyl	85
7	**10**	H	(1*R*)-1-(4-Fluorophenyl)ethyl	92
8	**11**	H	(4-Methoxyphenyl)methyl	60
9	**12**	H	(1*S*)-1-Naphthalen-2-ylethyl	85
10	**13**	H	(1*S*)-1-Naphthalen-1-ylethyl	93
11	**14**	H	(1*R*)-1-Naphthalen-1-ylethyl	86
12	**15**	H	Naphthalen-1-ylmethyl	75
13	**16**	Methyl	Phenylmethyl	92
14	**17**	Benzyl	Phenylmethyl	95
15	**18**	Ethyl	Ethyl	50
16	**19**	H	*i-*Propyl	80
17	**20**	H	Propargyl	86
18	**21**	Piperidine		80
19	**22**	*N*-Methylpiperazine		55
20	**23**	Morpholine		80

**Table 2:** Allo-gibberic acid-based chiral azole derivatives.

Entry	Compound	R	Yield (%)
1	**26**	Imidazole	60
2	**27**	Benzimidazole	66
3	**28**	1,2,4-Triazole	75
4	**29**	1,2,3-Triazole	50
5	**30**	Benzotriazole	45

**Table 3:** Chiral 3-amino-1,2-diol derivatives based on compound 33.

Entry	Compound	R^1^	R^2^	Yield (%)
1	**35**	H	(1*S*)-1-Naphthalen-2-ylethyl	86
2	**36**	H	(1*S*)-1-Naphthalen-1-ylethyl	60
3	**37**	H	(1*R*)-1-Naphthalen-1-ylethyl	87
4	**38**	H	Naphthalen-1-ylmethyl	78

## Supplementary Materials

The following supporting information can be downloaded at: https://www.mdpi.com/article/10.3390/ijms231810366/s1.
