# Supplementary material for: Stereoselective Synthesis and Application of Gibberellic Acid-Derived Aminodiols
Source: Int J Mol Sci. 2022 Sep 8;23(18):10366. doi: 10.3390/ijms231810366 (PMC9499365; doi:10.3390/ijms231810366)
Supplement: Supplementary file 1 [file ijms-23-10366-s001.zip › ijms-1884883-supplementary.pdf]

Supporting information

for

Stereoselective Synthesis and Application of Gibberellic Acid-Derived Aminodiols

Zein Alabdeen Khdar [1], Tam Minh Le [1,2], Zsuzsanna Schelz [3], István Zupkó [3] and Zsolt Szakonyi [1,*]

[1] Institute of Pharmaceutical Chemistry, University of Szeged, Interdisciplinary Excellent Center, Eötvös utca 6, H-6720 Szeged, Hungary

[2] Stereochemistry Research Group of the Hungarian Academy of Sciences, Eötvös utca 6, H-6720 Szeged, Hungary

[3] Institute of Pharmacodynamics and Biopharmacy, University of Szeged, Interdisciplinary Excellent Center, H-6720 Szeged, Hungary

* Correspondence: szakonyi.zsolt@szte.hu

Contents

1. Investigation of antiproliferative activity of gibberellic acid-based aminodiols ……………..S3 – S4

2. Investigation of antioxidant activity of gibberellic acid-based aminodiols ……………..S5 – S6

3. ^{1}H-, ^{13}C- NMR spectra of new aminodiol derivatives S7 – S100

4. Docking study S101 – S102

1. Investigation of anti-proliferative activity of gibberellic acid -based aminodiols

Table S1. Anti-proliferative activity of gibberellic acid -based aminodiol derivatives

Entry	Compounds	Concentration (μM)	Growth inhibition (%) ± SEM				
			HeLa	SiHa	MDA-MB-231	A2780	NIH/3T3
1	**4**	10	26.93 ± 1.55	23.34 ± 0.63	< 20	< 20	n.d.
		30	25.90 ± 3.20	56.97 ± 1.16	31.65 ± 1.49	49.88 ± 1.76	
2	**5**	10	22.33 ± 5.01	< 20	< 20	30.42 ± 0.22	
		30	51.04 ± 9.48	48.32 ± 4.04	64.91 ± 1.91	51.58 ± 2.69	
3	**6**	10	21.36 ± 1.70	< 20	< 20	25.31 ± 0.27	
		30	63.14 ± 1.81	65.87 ± 2.48	85.10 ± 1.38	61.89 ± 1.25	
4	**7**	10	< 20	28.91 ± 1.16	< 20	< 20	
		30	44.96 ± 0.97	94.29 ± 1.48	70.38 ± 0.07	65.04 ± 2.44	
5	**8**	10	27.53 ± 0.41	29.68 ± 1.54	< 20	39.47 ± 1.27	
		30	98.05 ± 0.07	98.38 ± 0.44	97.52 ± 0.67	96.38 ± 0.13	
6	**9**	10	< 20	26.61 ± 1.02	< 20	< 20	
		30	30.62 ± 4.19	53.60 ± 1.92	< 20	45.80 ± 1.62	
7	**10**	10	38.52 ± 2.02	39.12 ± 1.82	31.20 ± 0.77	22.24 ± 1.93	
		30	97.93 ± 0.11	97.08 ± 0.08	94.51 ± 1.05	96.46 ± 0.76	
8	**11**	10	< 20	30.70 ± 0.82	< 20	31.69 ± 2.82	
		30	60.77 ± 2.44	53.74 ± 0.76	78.68 ± 1.47	53.40 ± 1.68	
9	**12**	10	97.69 ± 0.28	97.41 ± 0.15	95.00 ± 0.69	96.29 ± 0.59	90.43 ± 0.15
		30	97.62 ± 0.55	97.06 ± 0.20	93.72 ± 0.88	96.28 ± 0.82	91.03 ± 0.05
		**IC_{50} (μM)**	**4.14 ± 0.38**	**4.17 ± 0.32**	**7.19 ± 1.48**	**5.45 ± 0.31**	**4.57 ± 0.23**
10	**13**	10	97.16 ± 0.49	96.05 ± 0.46	94.86 ± 0.83	91.20 ± 1.96	40.50 ± 0.86
		30	96.87 ± 0.47	96.74 ± 0.27	96.00 ± 0.92	96.74 ± 1.17	88.10 ± 0.16
		**IC_{50} (μM)**	**4.38 ± 0.08**	**4.69 ± 0.17**	**7.49 ± 1.26**	**5.30 ± 0.41**	**10.88 ± 0.16**
11	**14**	10	98.08 ± 0.19	96.28 ± 2.13	96.57 ± 0.37	96.53 ± 0.73	88.58 ± 0.97
		30	97.40 ± 0.34	97.84 ± 0.42	96.77 ± 0.16	95.87 ± 1.06	88.65 ± 0.98
		**IC_{50} (μM)**	**4.66 ± 0.21**	**5.25 ± 0.86**	**8.04 ± 0.61**	**5.15 ± 0.28**	**5.02 ± 0.26**
12	**15**	10	28.19 ± 0.88	28.61 ± 0.15	< 20	21.91 ± 1.50	n.d.
		30	96.70 ± 1.54	97.95 ± 0.63	95.96 ± 0.69	95.48 ± 0.65	
13	**16**	10	< 20	< 20	< 20	< 20	
		30	36.00 ± 2.89	22.96 ± 0.39	25.01 ± 0.99	21.55 ± 0.85	

14	**17**	10	40.84 ± 1.19	< 20	< 20	< 20	
		30	45.65 ± 1.48	47.13 ± 1.74	36.87 ± 1.58	78.87 ± 1.12	
15	**24**	10	< 20	< 20	< 20	< 20	
		30	53.94 ± 2.21	31.90 ± 1.28	42.37 ± 1.46	34.15 ± 1.77	
16	**25**	10	< 20	< 20	< 20	< 20	
		30	< 20	< 20	< 20	< 20	
17	**26**	10	< 20	< 20	< 20	< 20	
		30	< 20	25.88 ± 2.53	< 20	< 20	
18	**27**	10	< 20	26.25 ± 0.46	< 20	< 20	
		30	45.52 ± 1.87	44.44 ± 1.84	< 20	28.86 ± 1.27	
19	**30**	10	< 20	< 20	< 20	< 20	
		30	< 20	29.52 ± 2.00	< 20	26.76 ± 3.79	
20	**31**	10	< 20	< 20	< 20	< 20	
		30	40.17 ± 5.59	42.92 ± 0.84	50.75 ± 3.01	41.40 ± 1.13	
21	**32**	10	< 20	< 20	< 20	< 20	
		30	< 20	31.00 ±1.43	< 20	< 20	
22	**35**	10	40.15 ± 1.21	44.58 ± 0.74	29.39 ± 1.66	42.68 ± 2.63	
		30	95.90 ± 2.21	98.41 ± 0.74	96.81 ± 0.82	95.40 ± 0.48	
23	**36**	10	31.09 ± 2.01	31.41 ± 1.21	46.86 ± 1.05	46.73 ± 1.46	
		30	95.37 ± 2.87	97.69 ± 0.58	95.53 ± 0.83	94.81 ± 0.11	
24	**37**	10	35.80 ± 0.42	34.52 ± 1.89	< 20	35.87 ± 0.89	
		30	97.35 ± 0.69	97.58 ± 0.47	96.64 ± 0.17	96.05 ± 0.99	
25	**38**	10	26.85 ± 0.53	27.95 ± 0.93	< 20	28.63 ± 2.66	
		30	96.46 ± 1.72	97.63 ± 0.80	96.17 ± 0.76	95.23 ± 0.66	
	**Cisplatin**	10	42.61 ± 2.33	88.64 ± 0.50	67.51 ± 1.01	83.57 ± 1.21	73.88 ± 1.63
		30	99.93 ± 0.26	90.18 ± 1.78	87.75 ± 1.10	95.02 ± 0.28	97.10 ± 0.15
		**IC_{50} (μM)**	**12.43**	**7.84**	**3.74**	**1.30**	**5.49**

n.d.: not determined

2. Figure S1 Investigation of antioxidant activity of gibberellic acid-based aminodiols. DPPH radical scavenging activity is represented by inhibition (%). Trolox (3,4-Dihydro-6-hydroxy-2,5,7,8-tetramethyl-2*H*-1-benzopyran-2-carboxylic acid) was used as reference antioxidant with a 20.15 μM IC_{50} value.

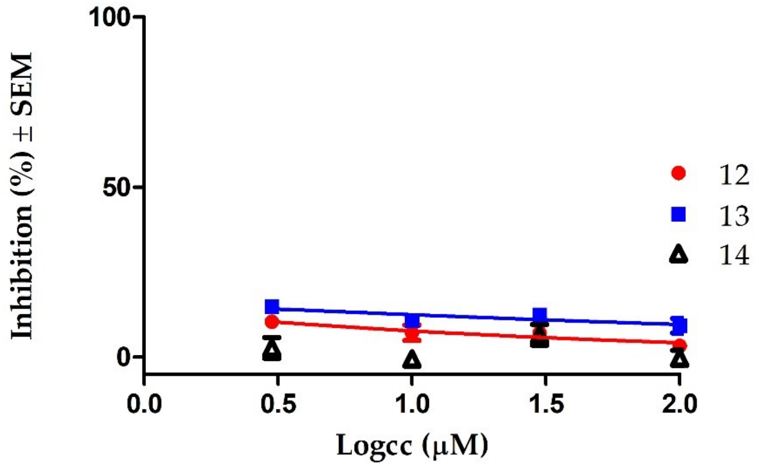

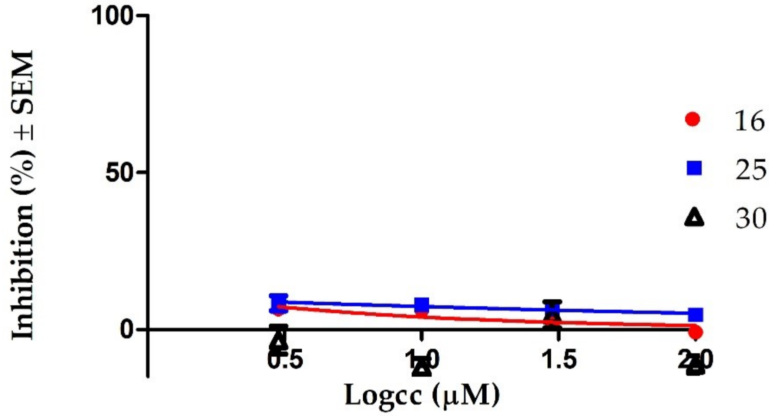

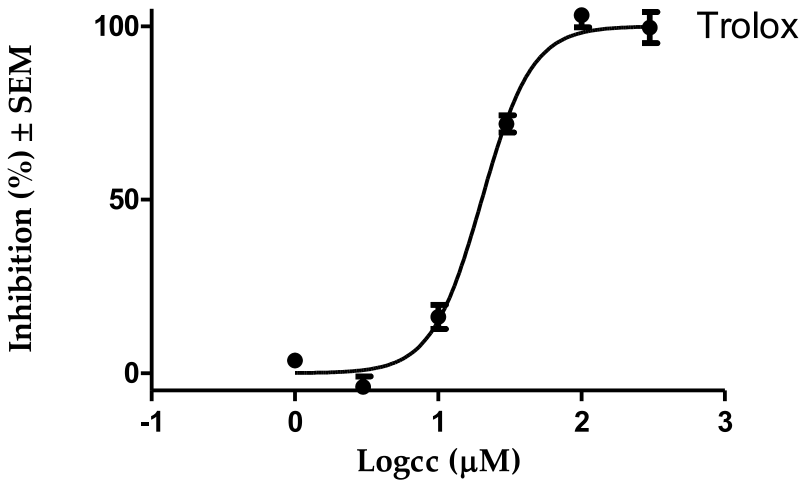
Trolox
Inhibition (%) ± SEM
100
50
0
-1
0
1
2
3
Logcc (μM)

2. ^{1}H-, ^{13}C- NMR spectra of new aminodiol derivatives

Figure S 2: ^{1}H-NMR of compound 2

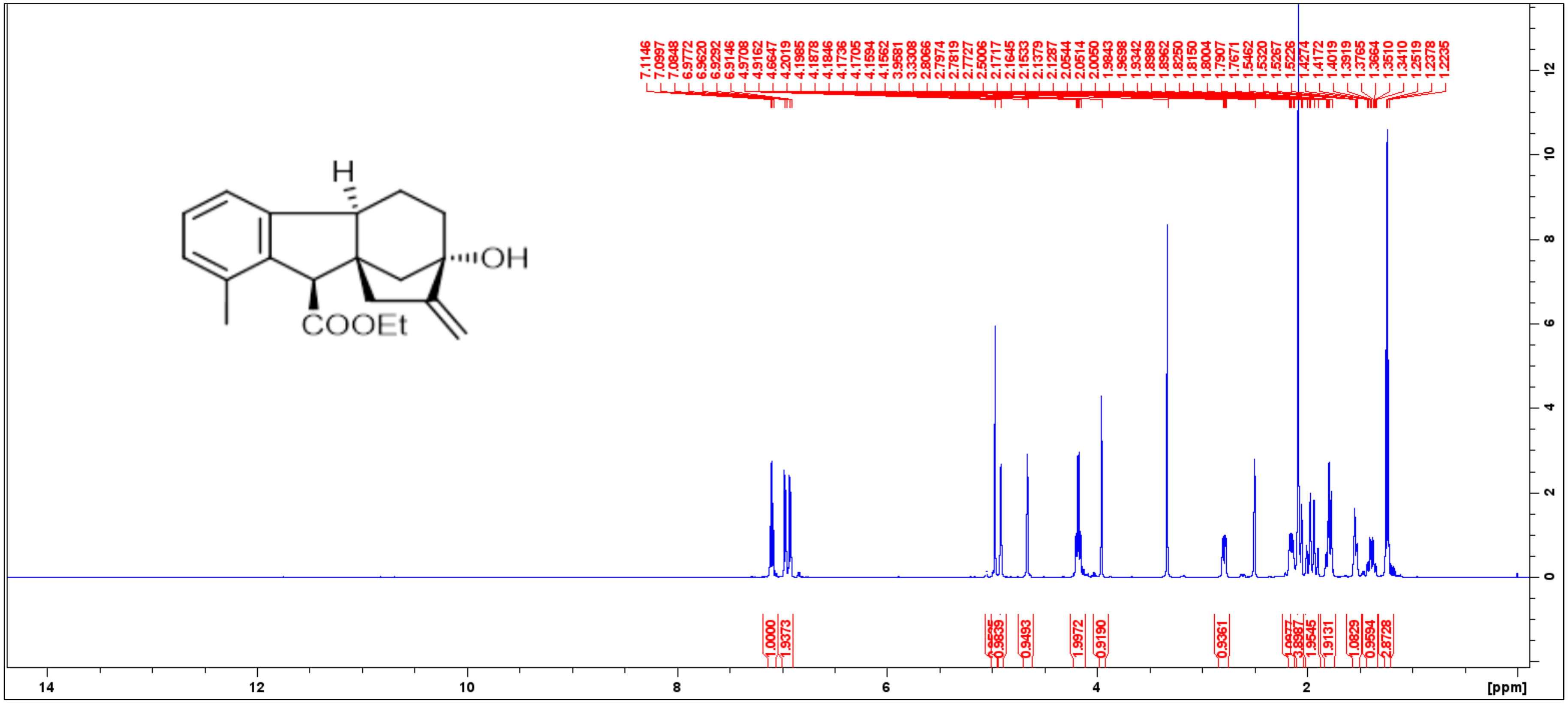

Figure S 3: ^{13}C-NMR (JMOD) (JMOD) of compound 2

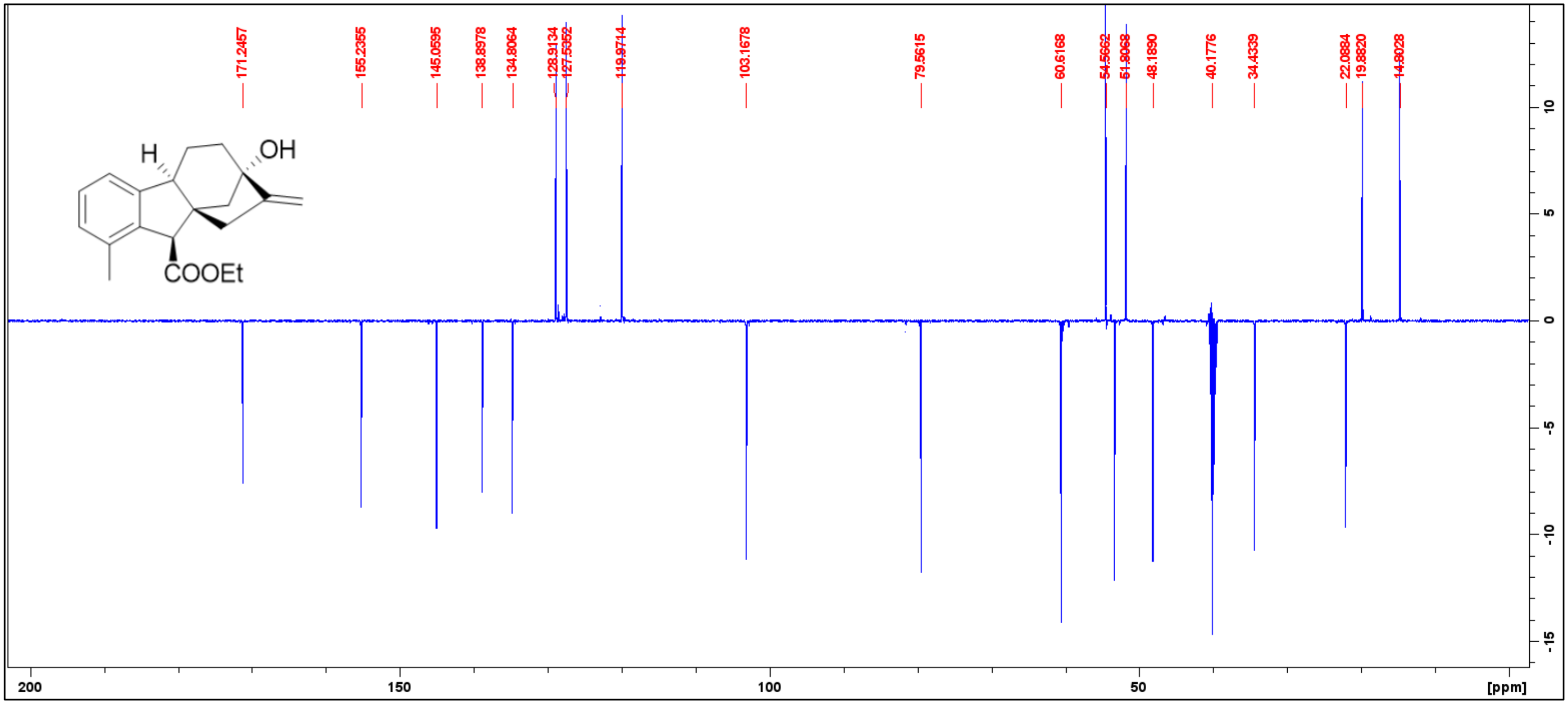

Figure S 4: ^{1}H-NMR of compound 3

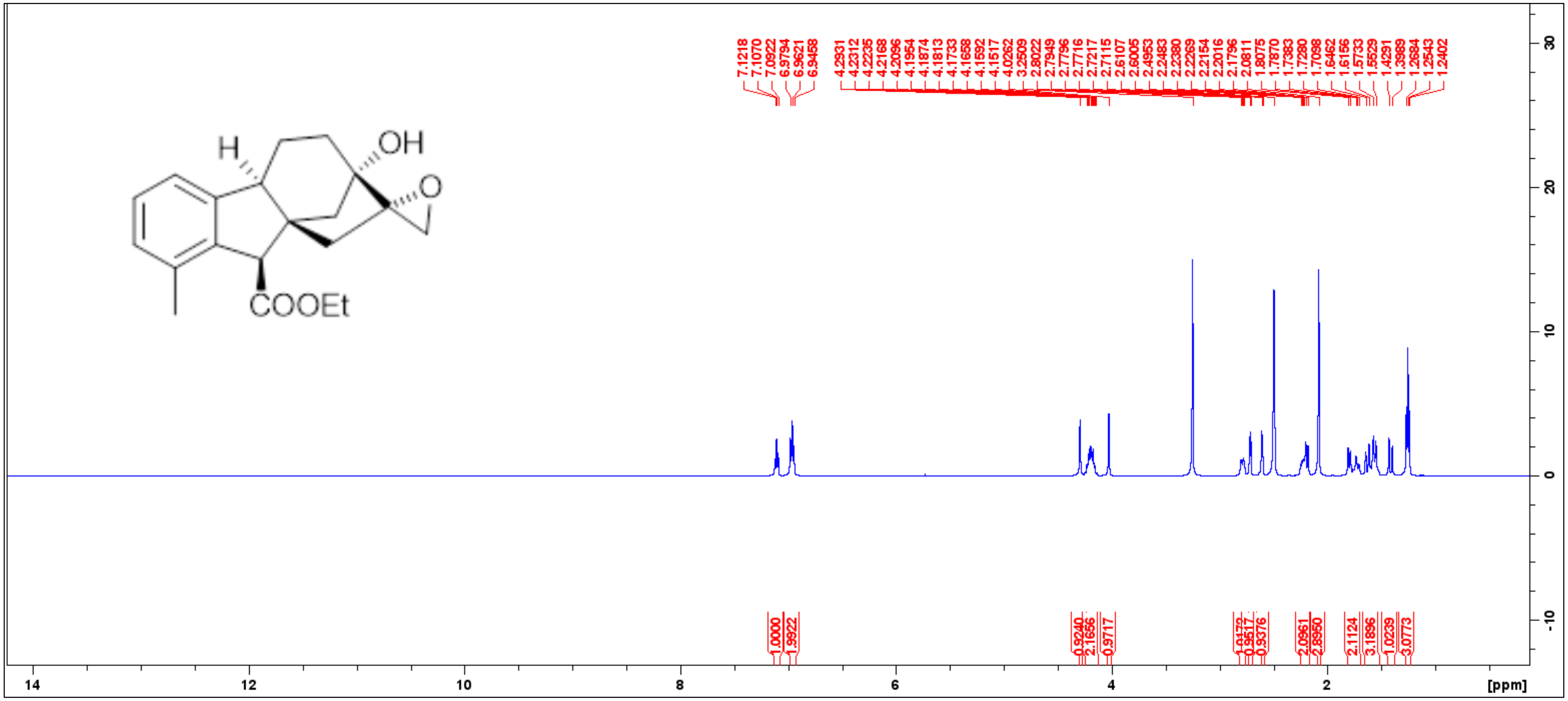

Figure S 5: ^{13}C-NMR (JMOD) of compound 3

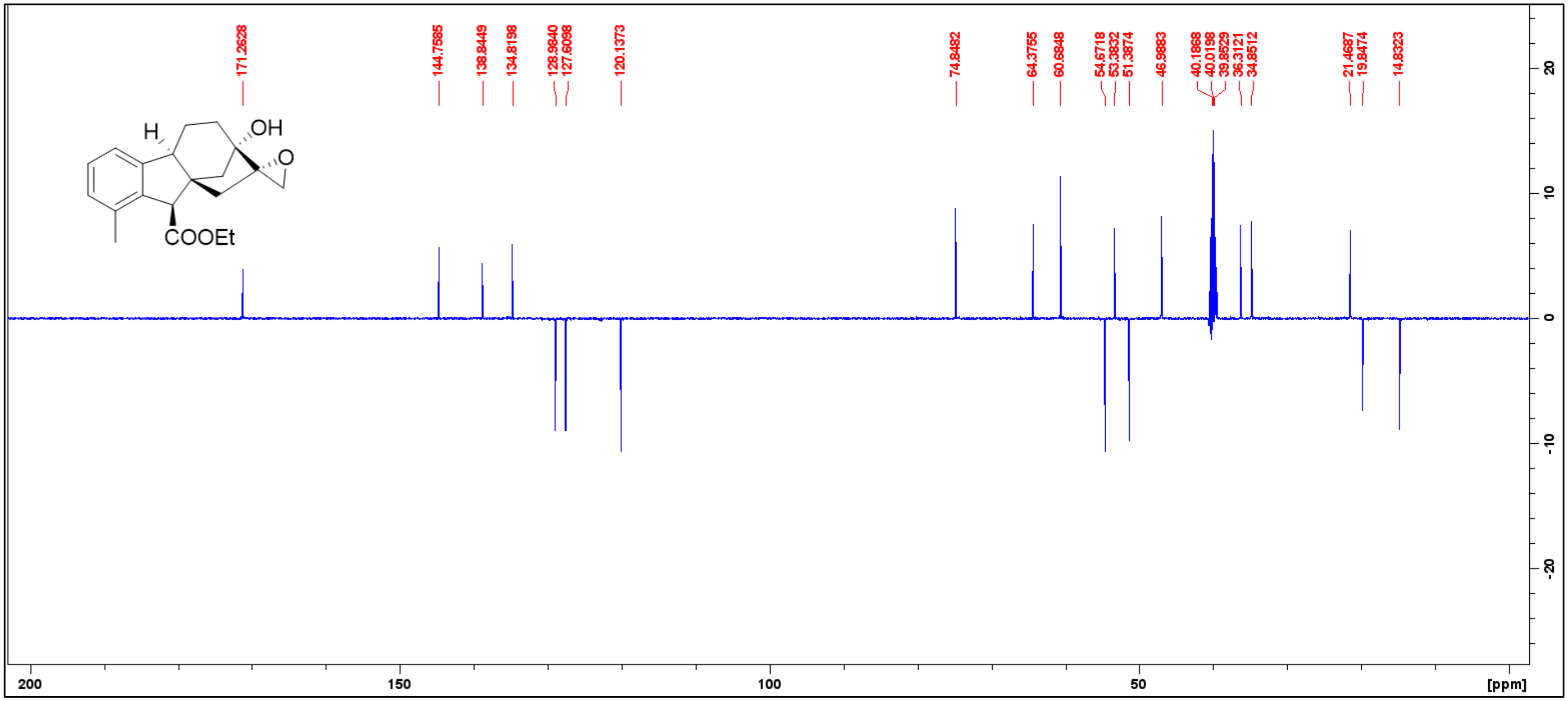

Figure S 6: COSY of compound 3

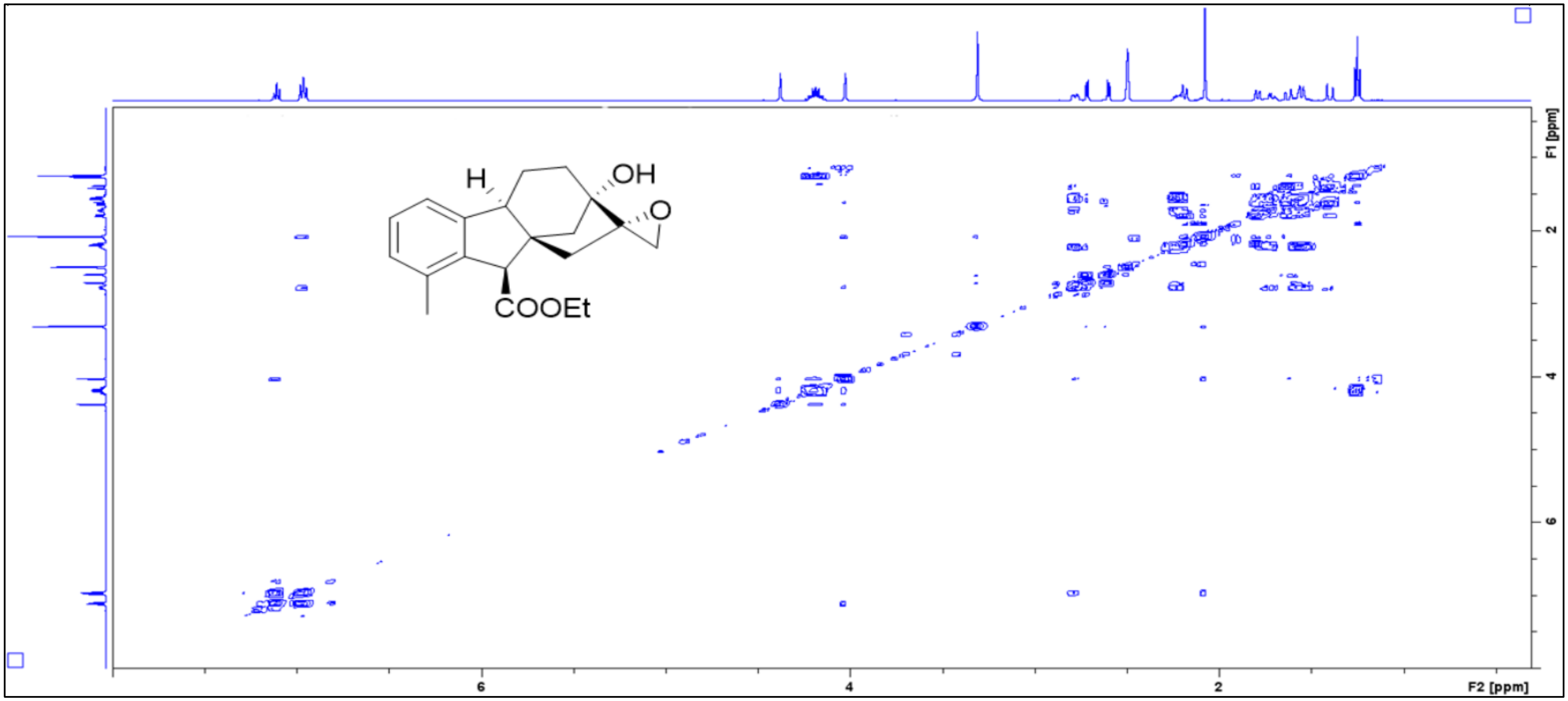

Figure S 7: NOESY of compound 3

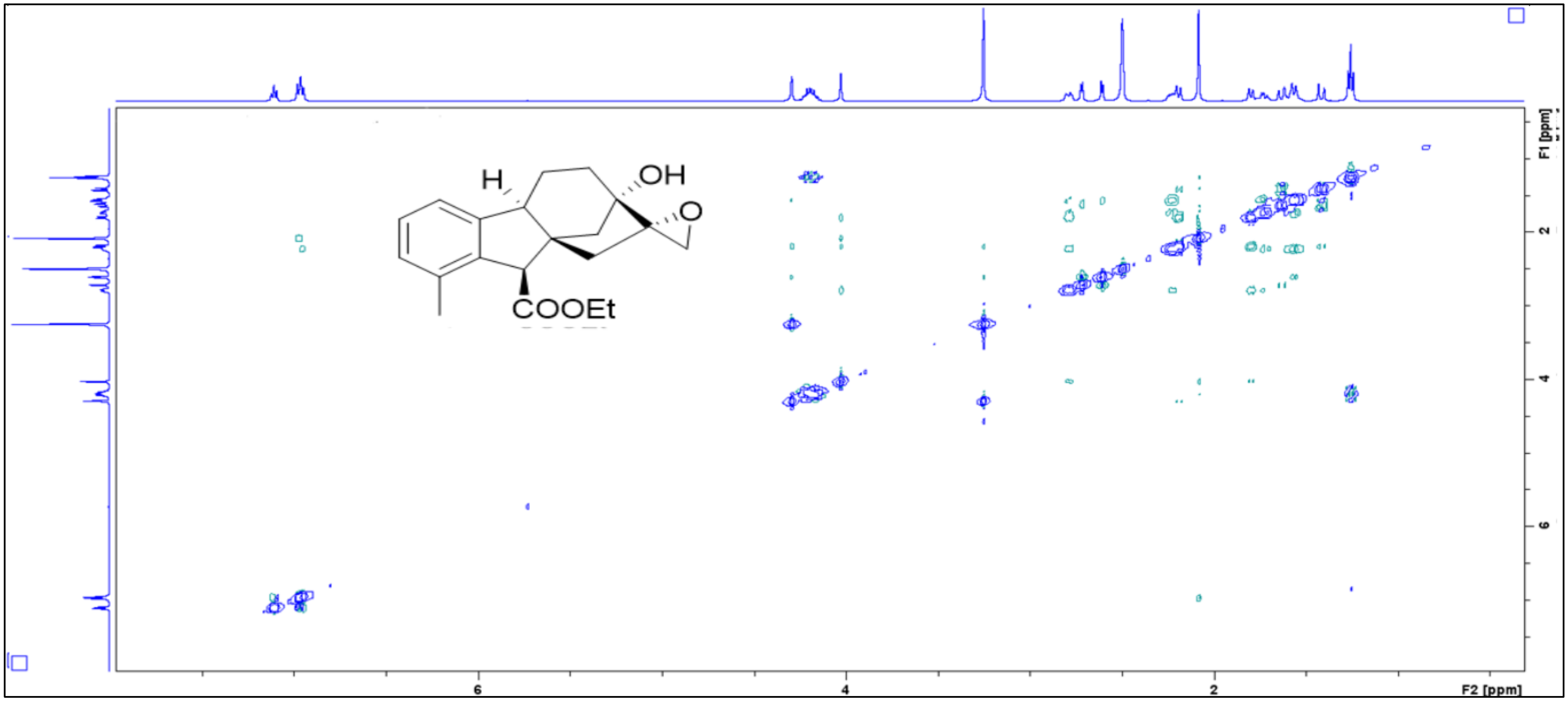

Figure S 8: ^{1}H-NMR of compound 4

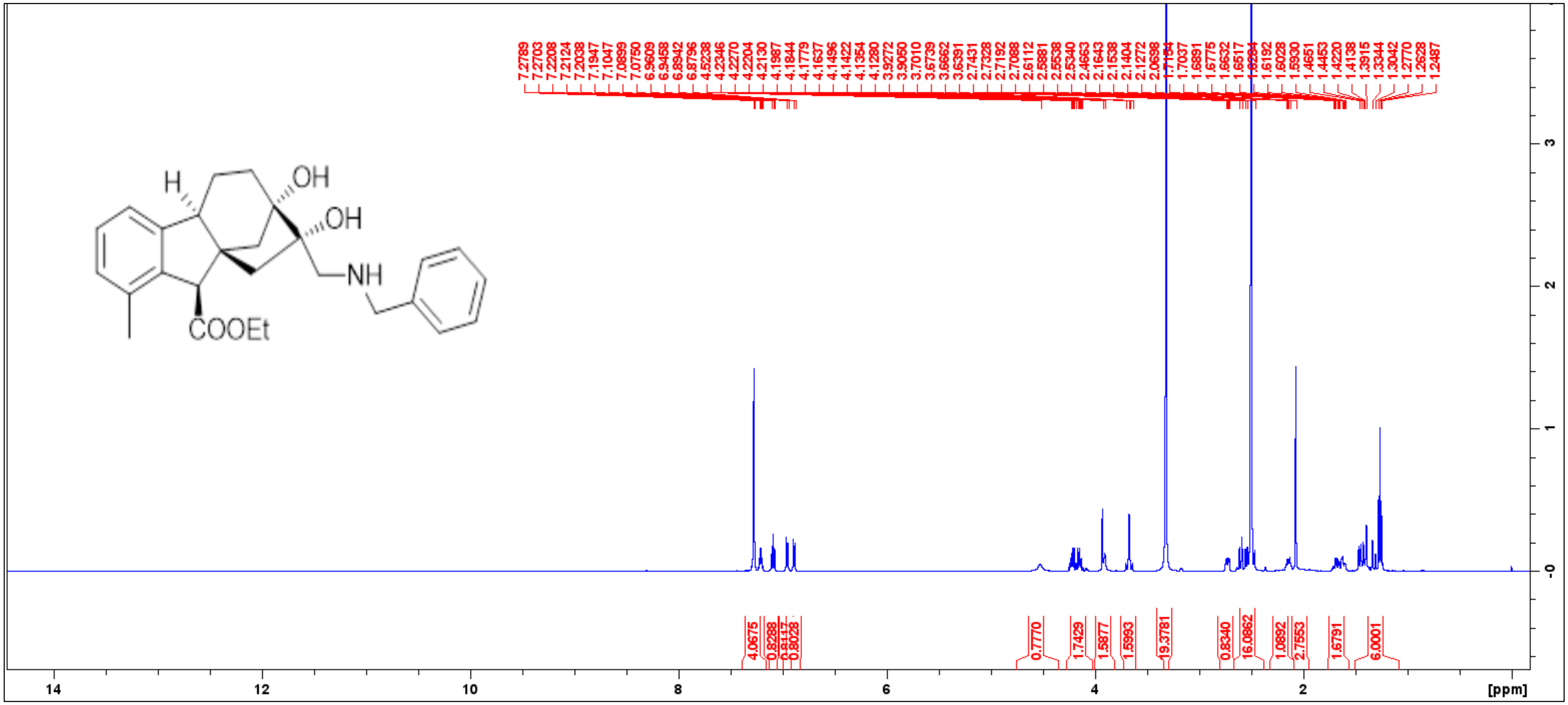

Figure S 9: ^{13}C-NMR (JMOD) of compound 4

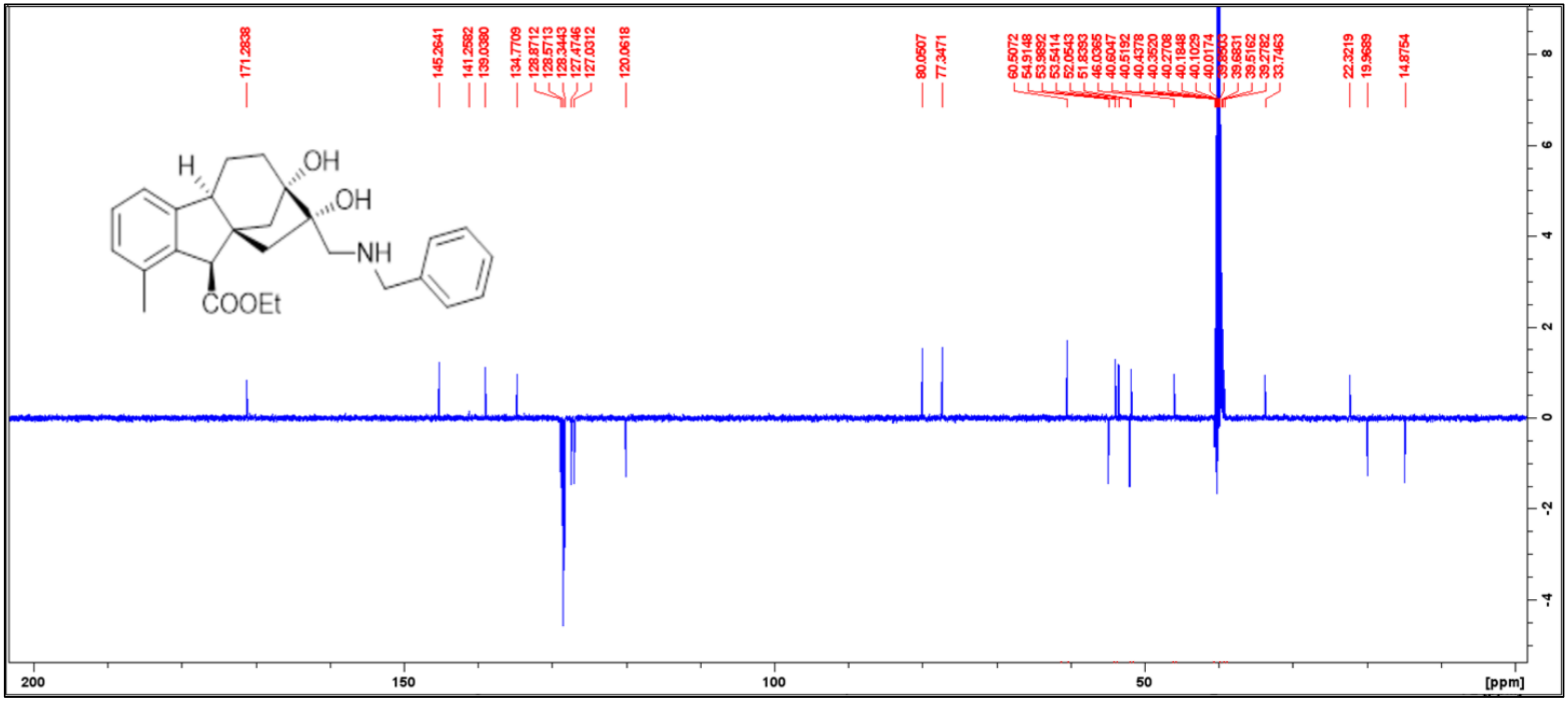

Figure S 10: COSY of compound 4

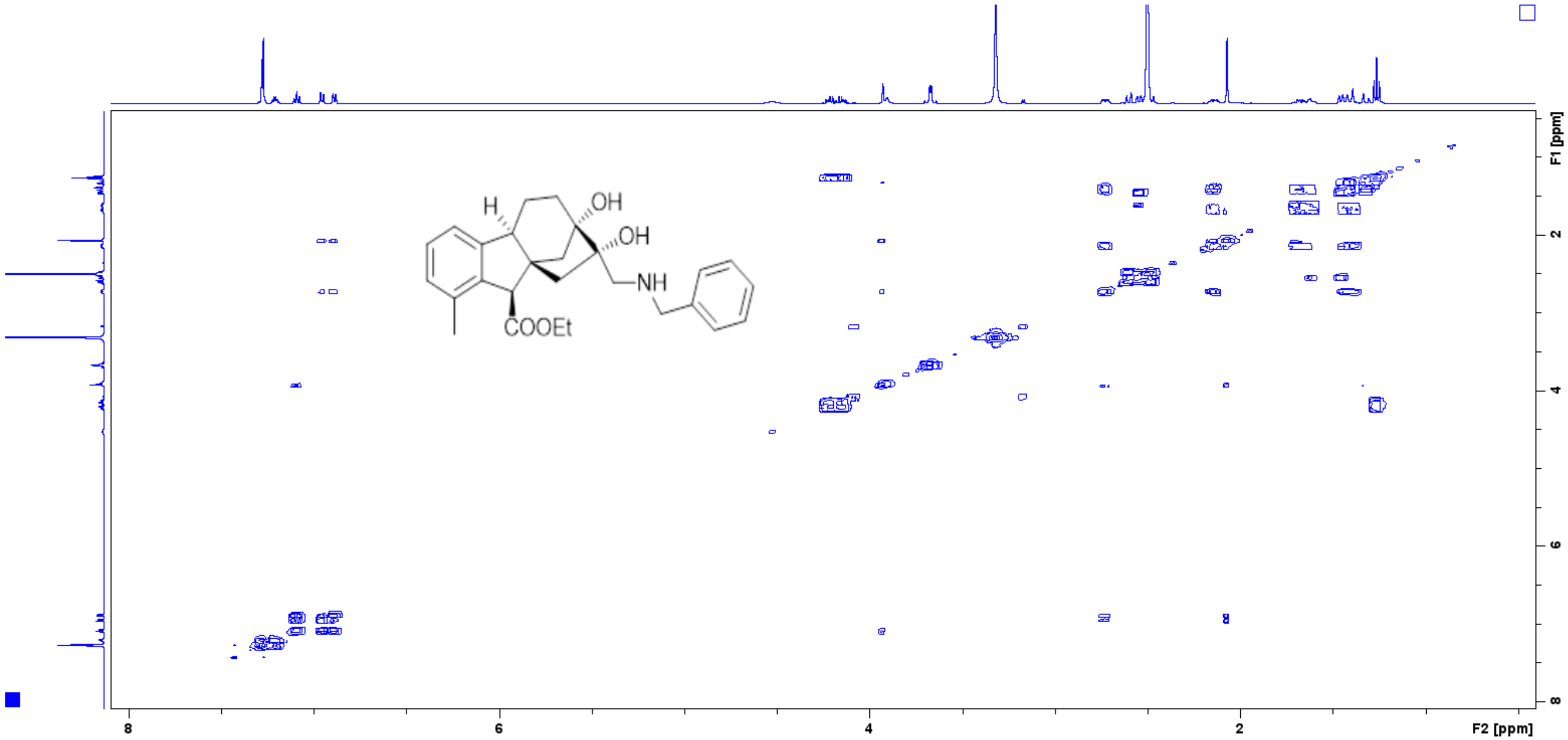

Figure S 11: NOESY of compound 4

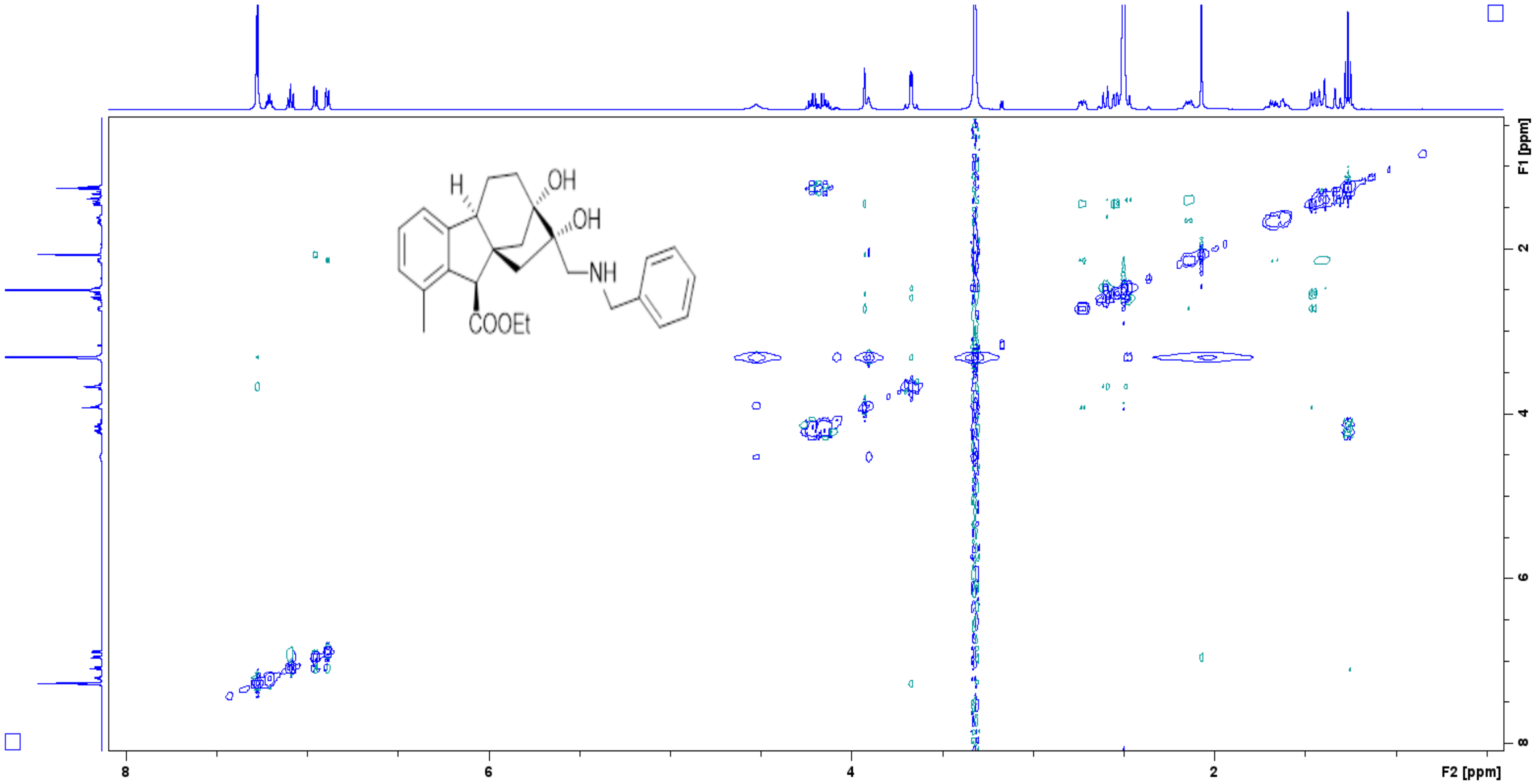

Figure S 12: ^{1}H-NMR of compound 5

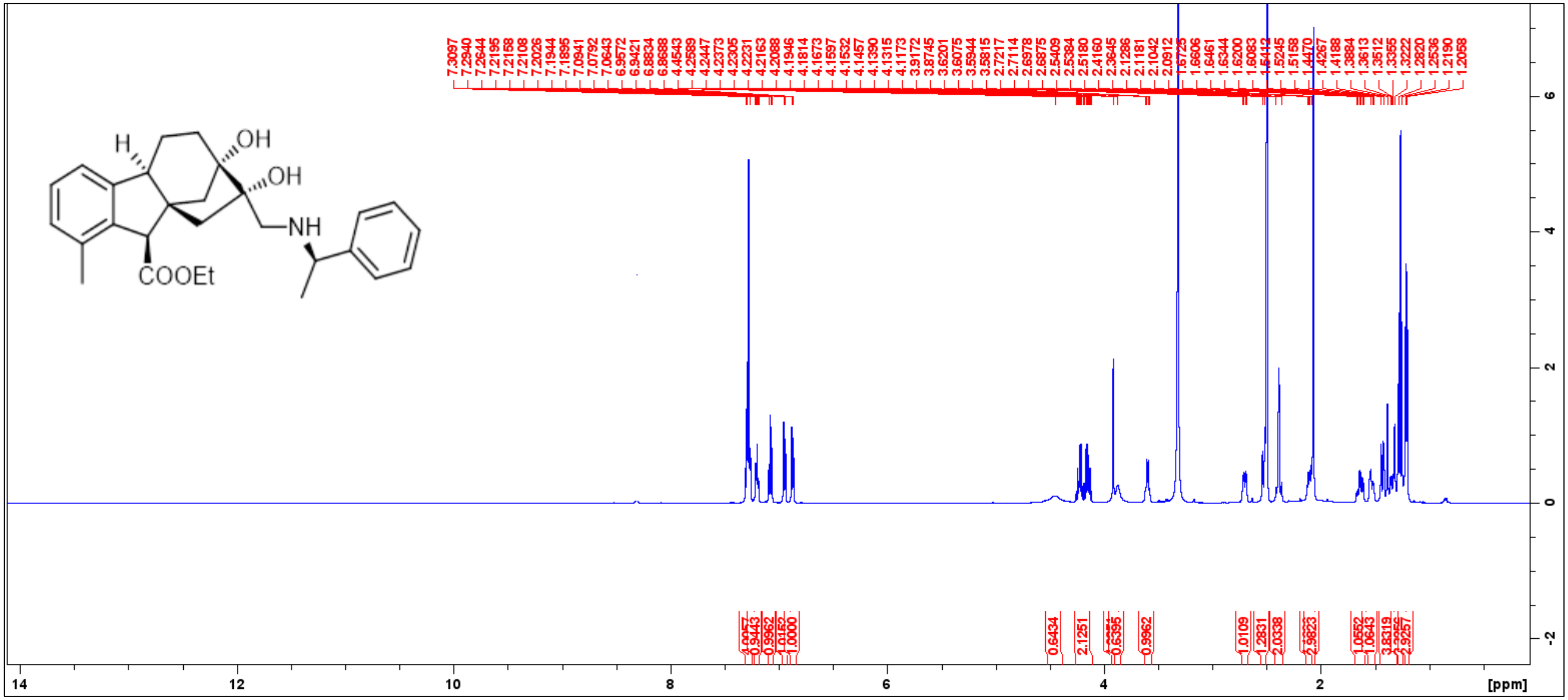

Figure S 13: ^{13}C-NMR (JMOD) of compound 5

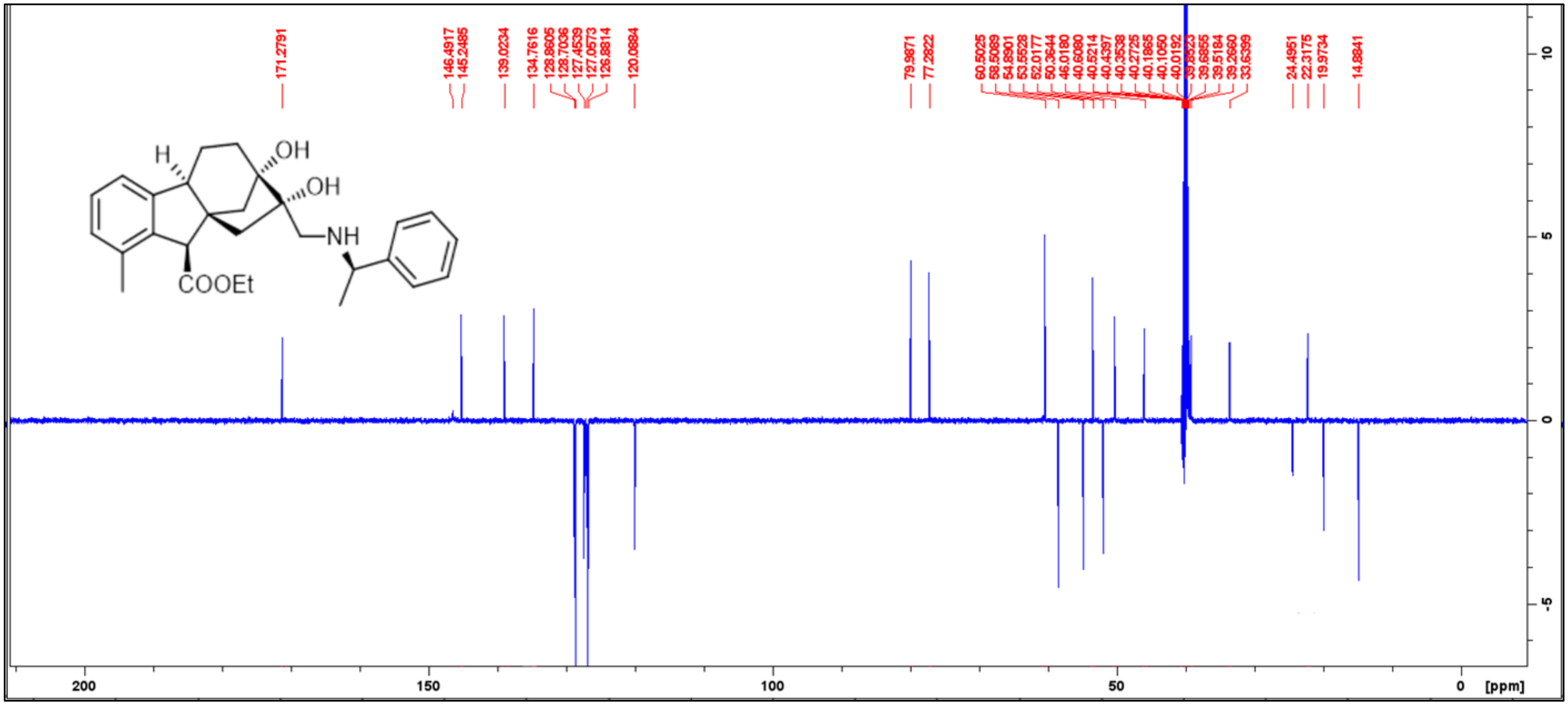

Figure S 14: ^{1}H-NMR of compound 6

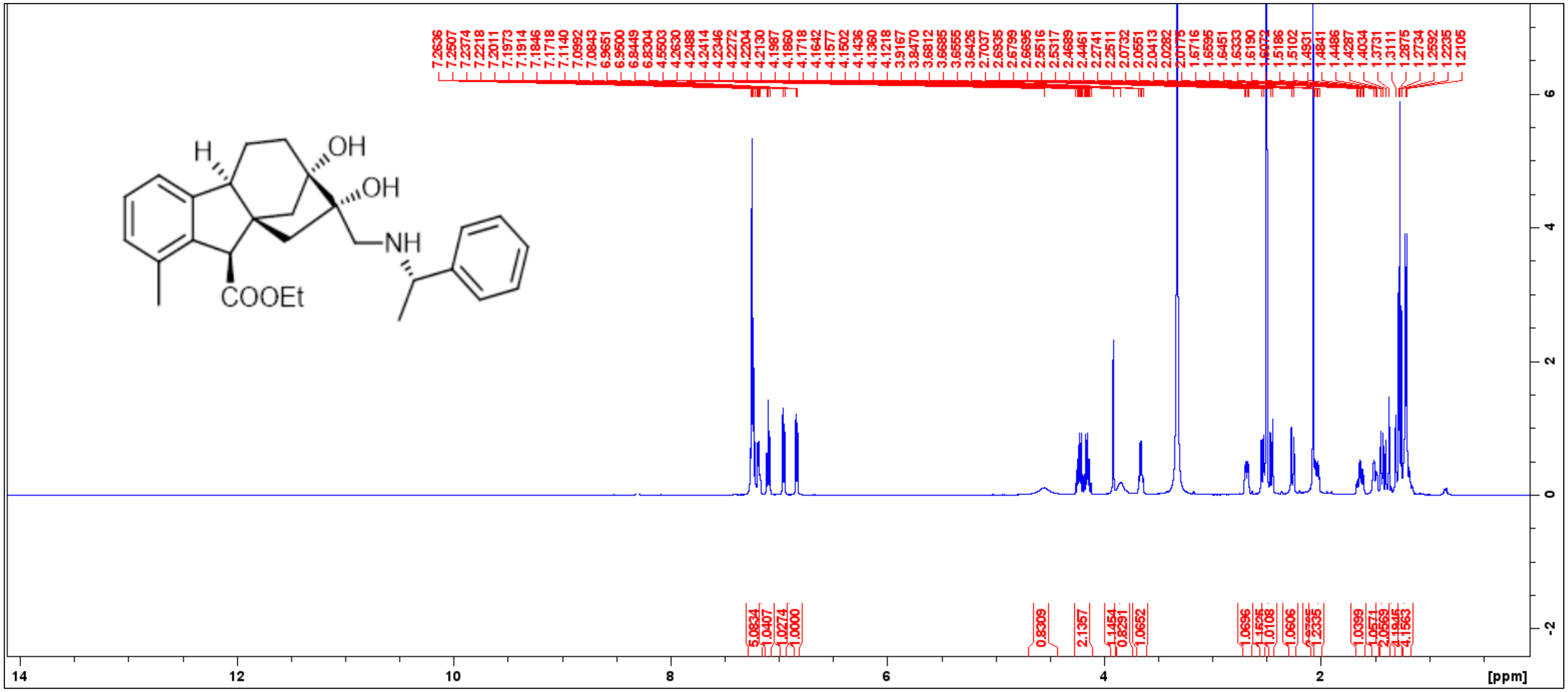

Figure S 15: ^{13}C-NMR (JMOD) of compound 6

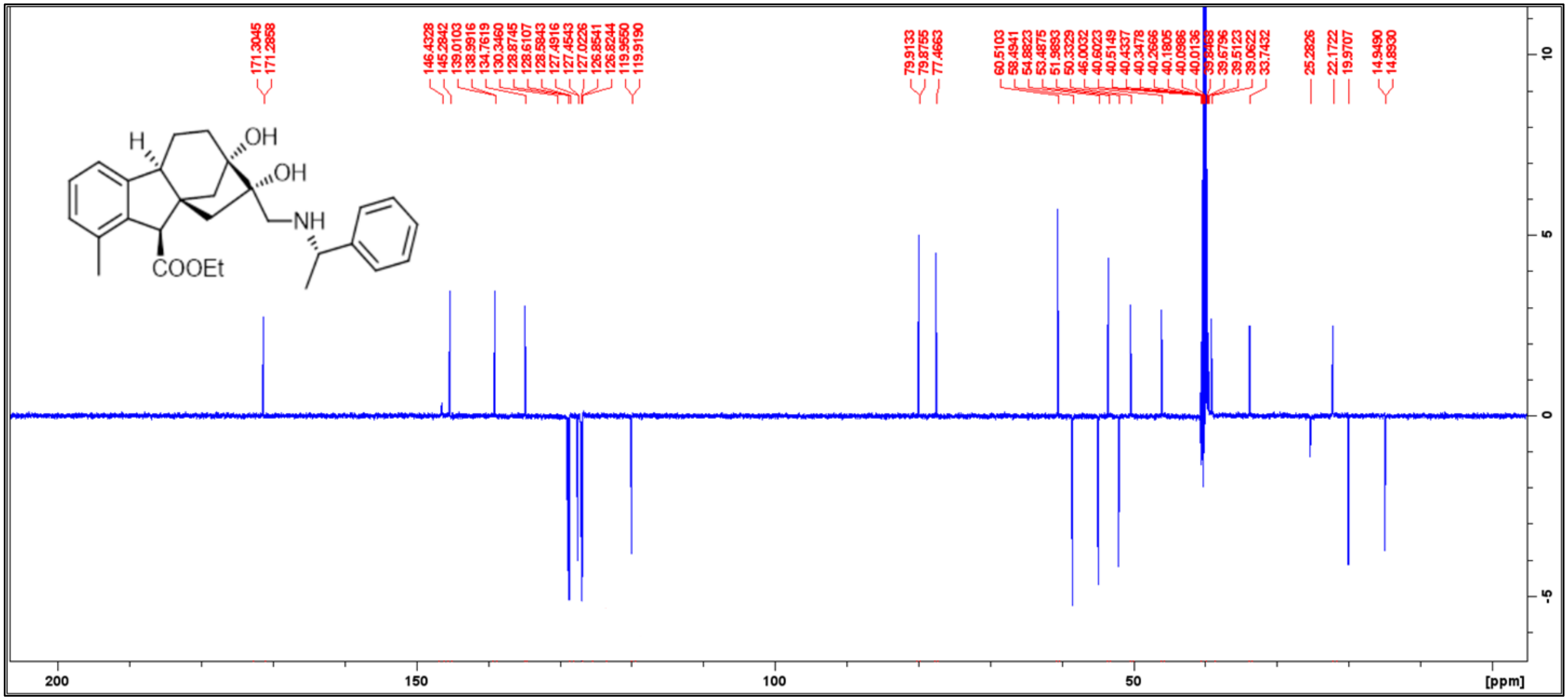

Figure S 16: ^{1}H-NMR of compound 7

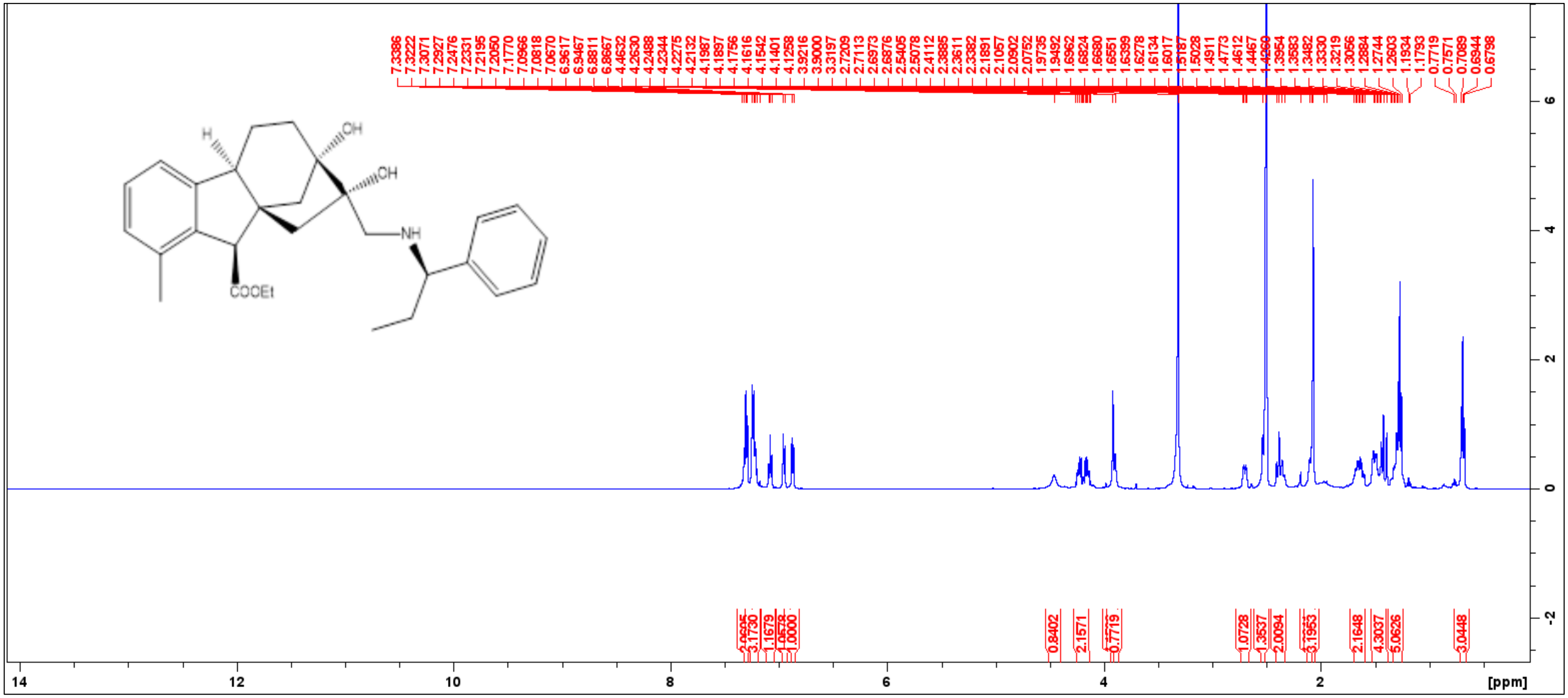

Figure S 17: ^{13}C-NMR (JMOD) of compound 7

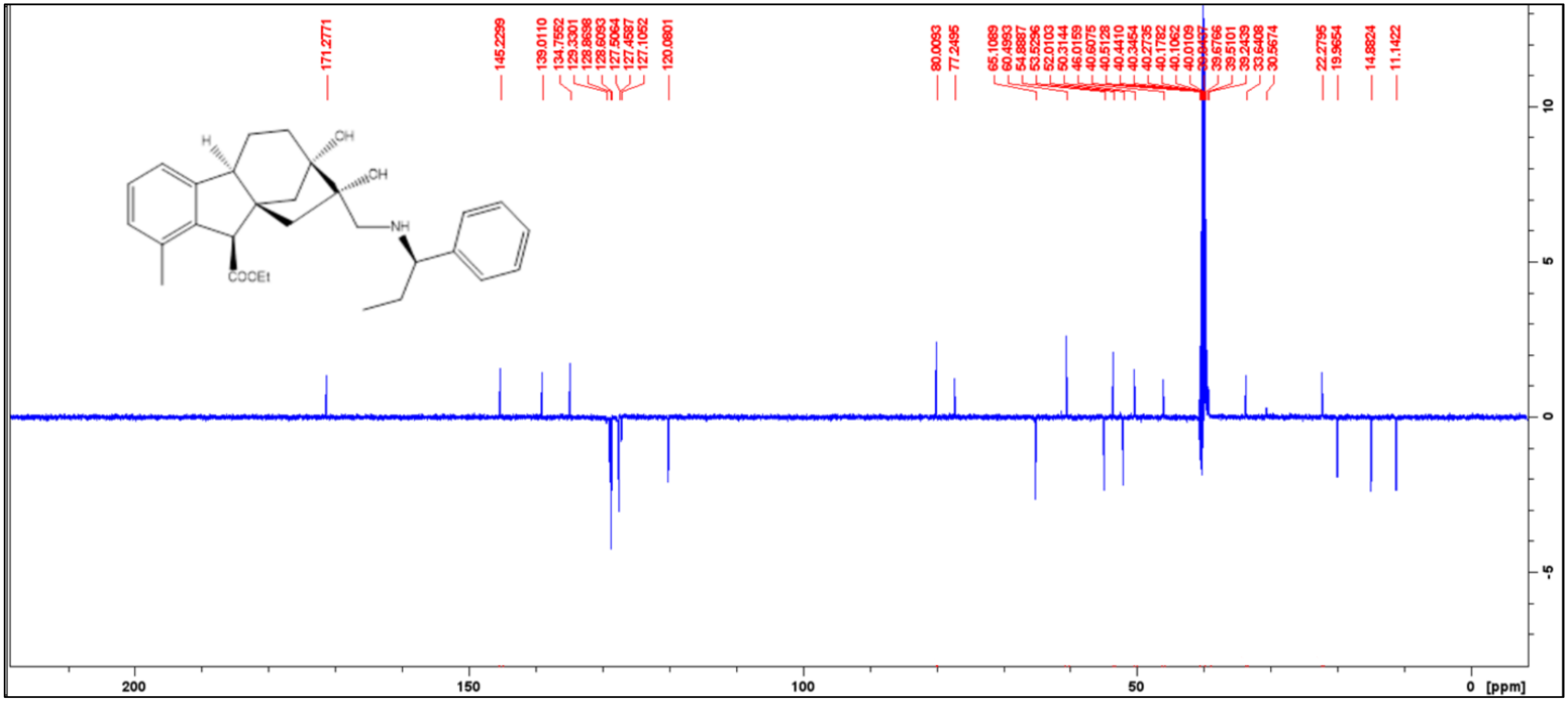

Figure S 18: ^{1}H-NMR of compound 8

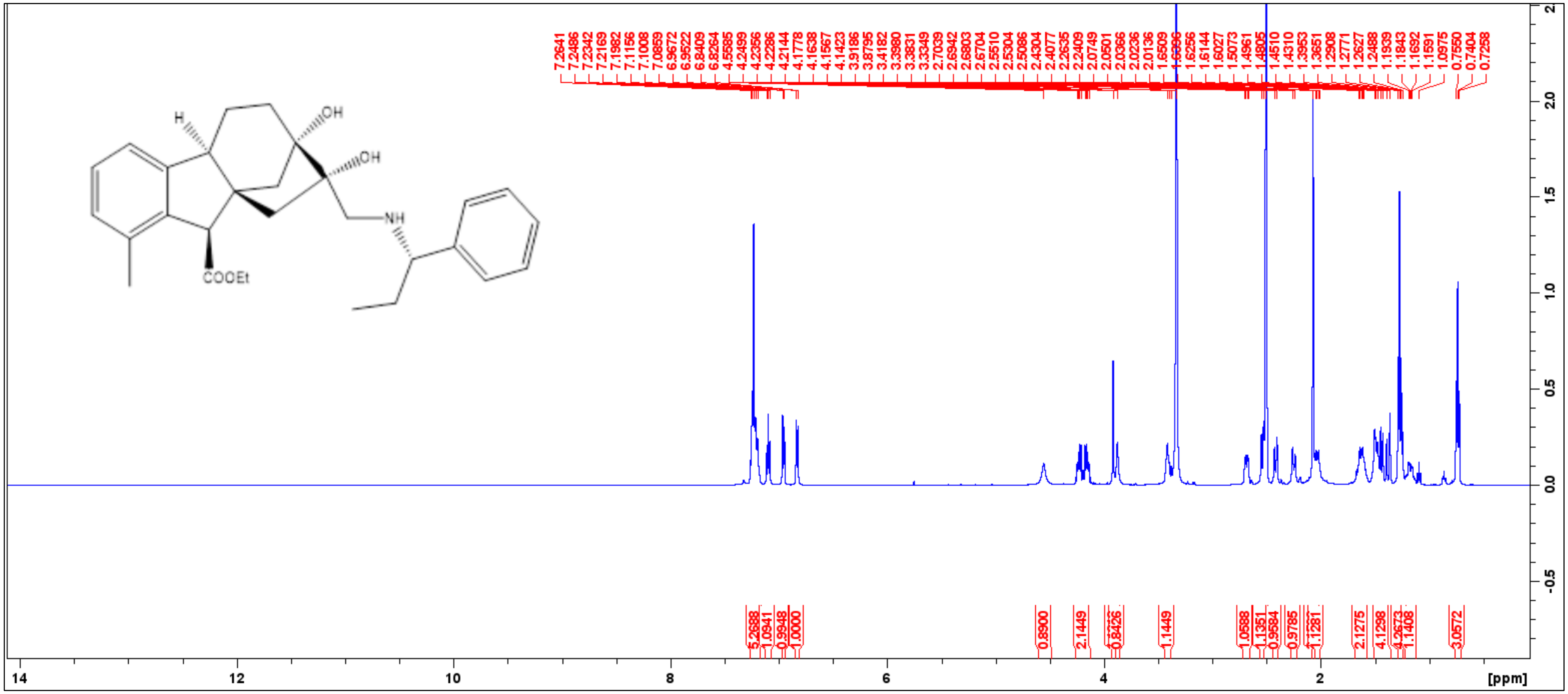

Figure S 19: ^{13}C-NMR (JMOD) of compound 8

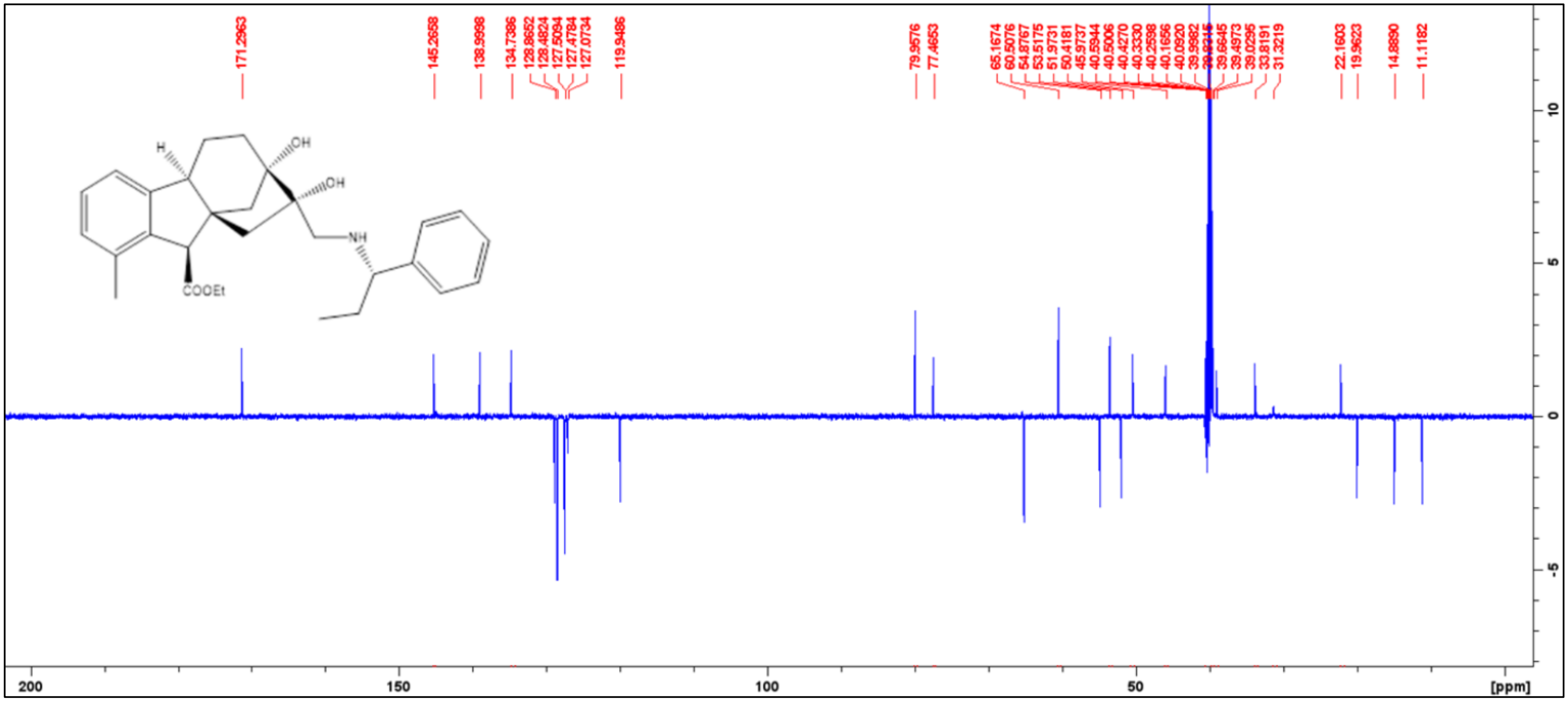

Figure S 20: ^{1}H-NMR of compound 9

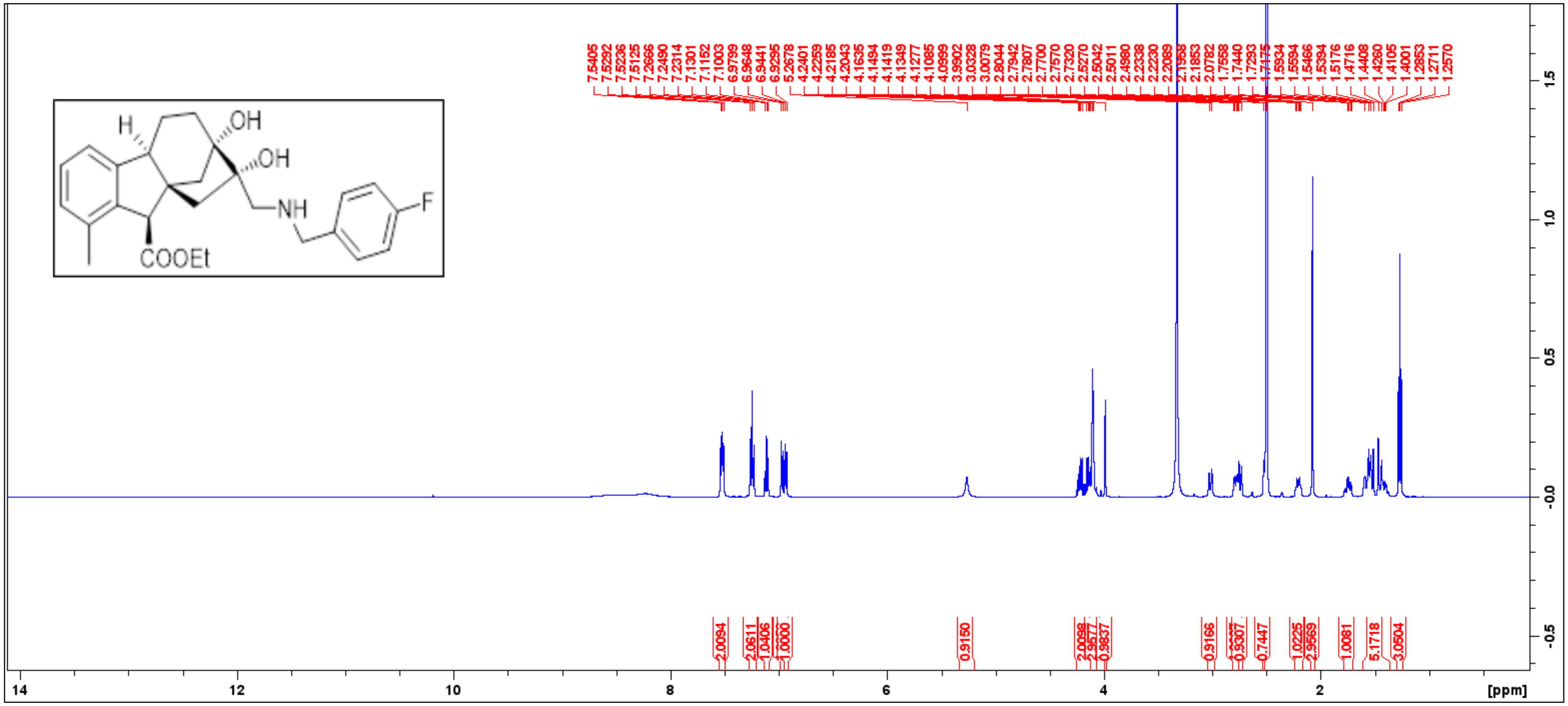

Figure S 21: ^{13}C-NMR (JMOD) of compound 9

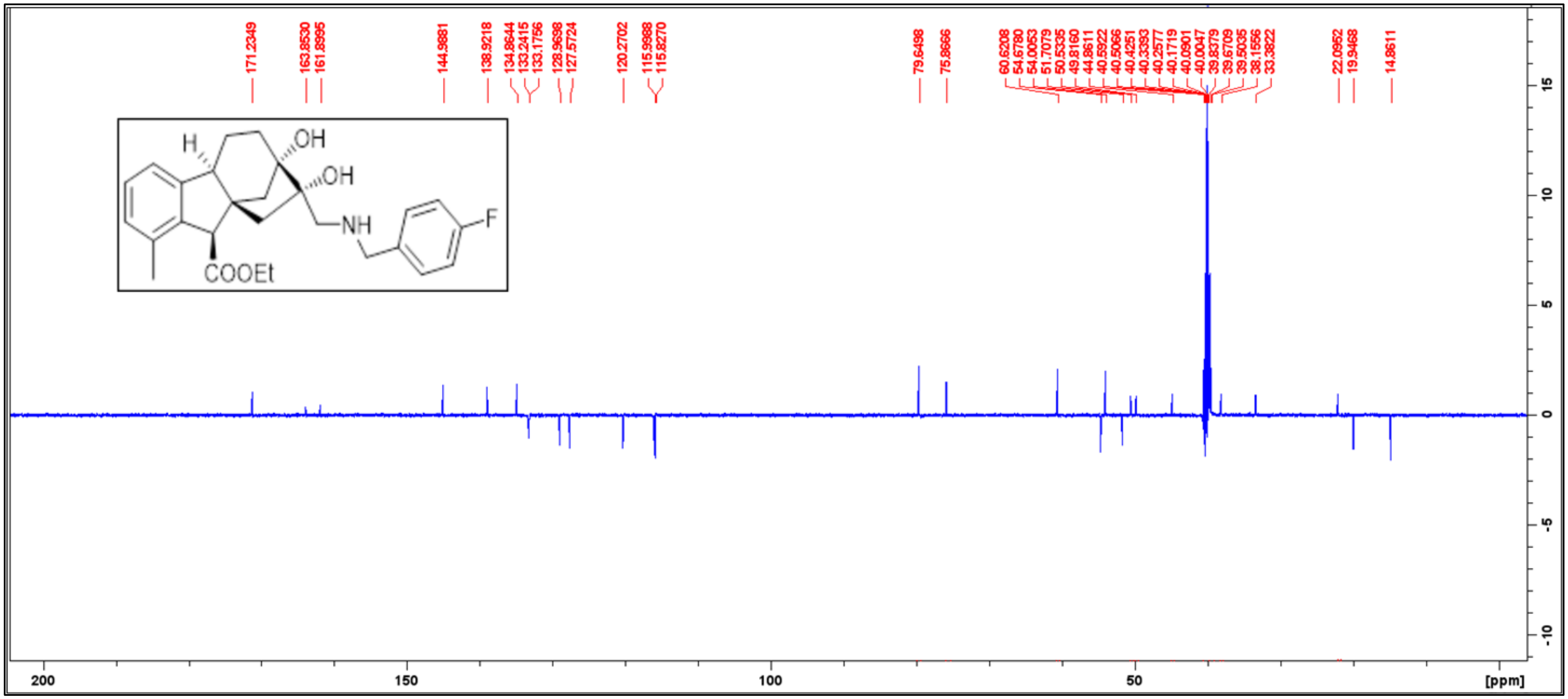

Figure S 22: ^{1}H-NMR of compound 10

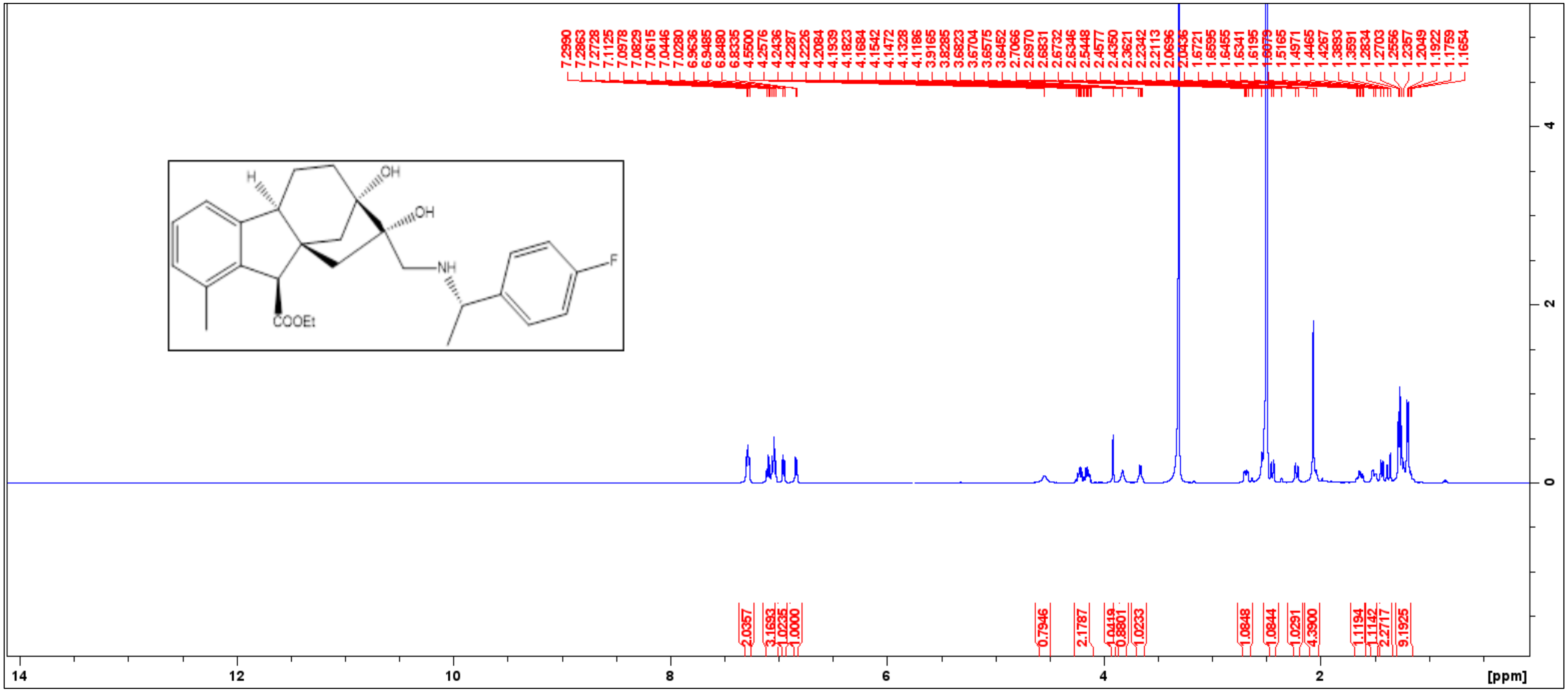

Figure S 23: ^{13}C-NMR (JMOD) of compound 10

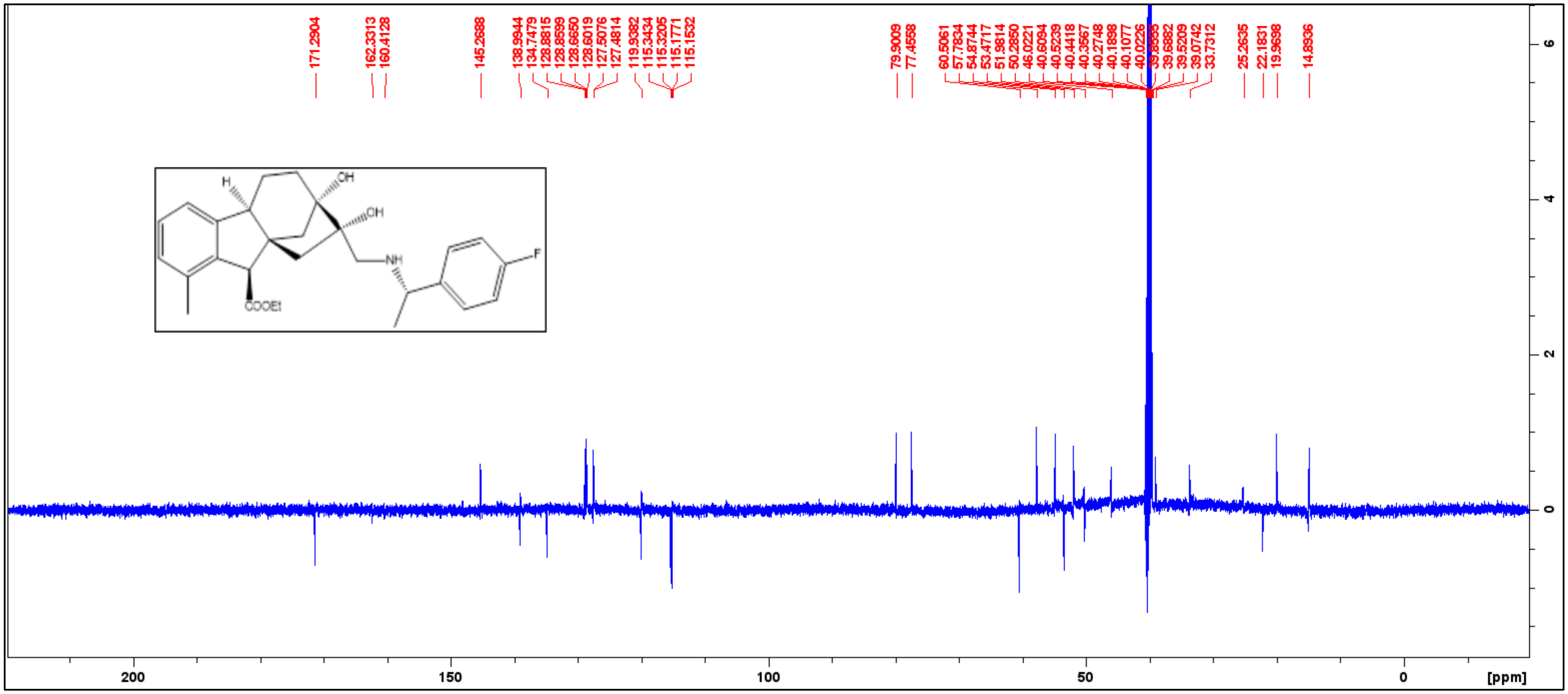

Figure S 24: HSQC of compound 10

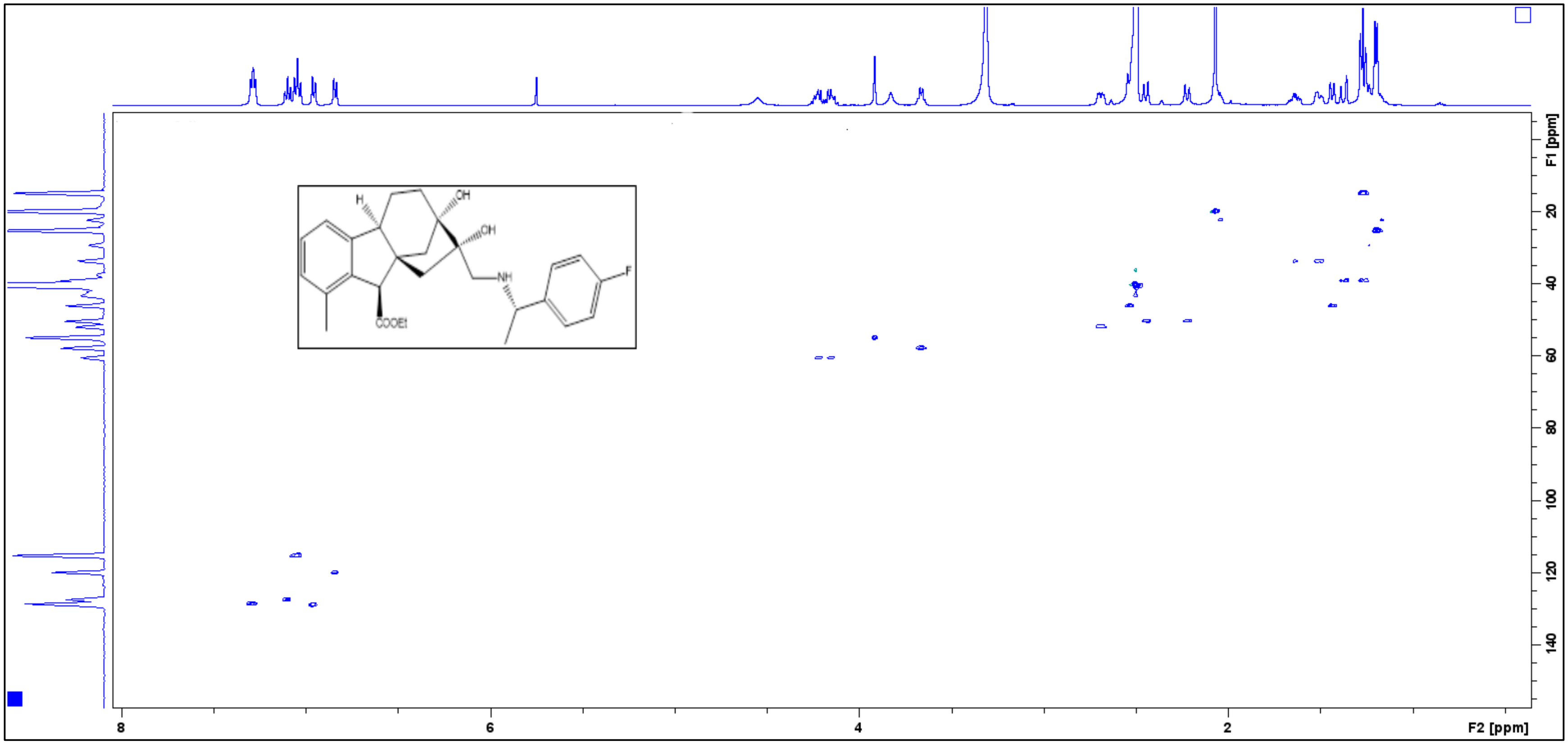

Figure S 25: HMBC of compound 10

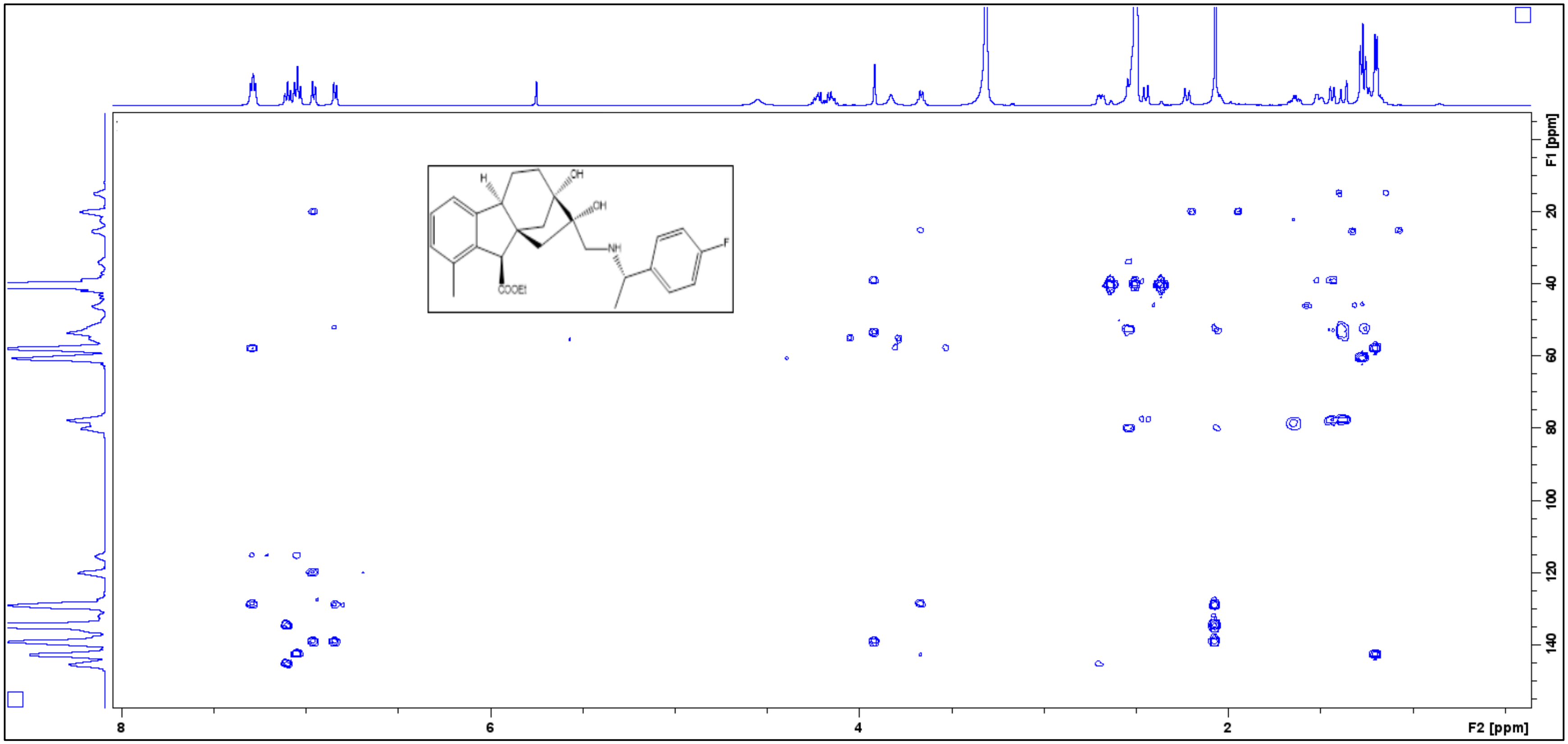

Figure S 26: ^{1}H-NMR of compound 11

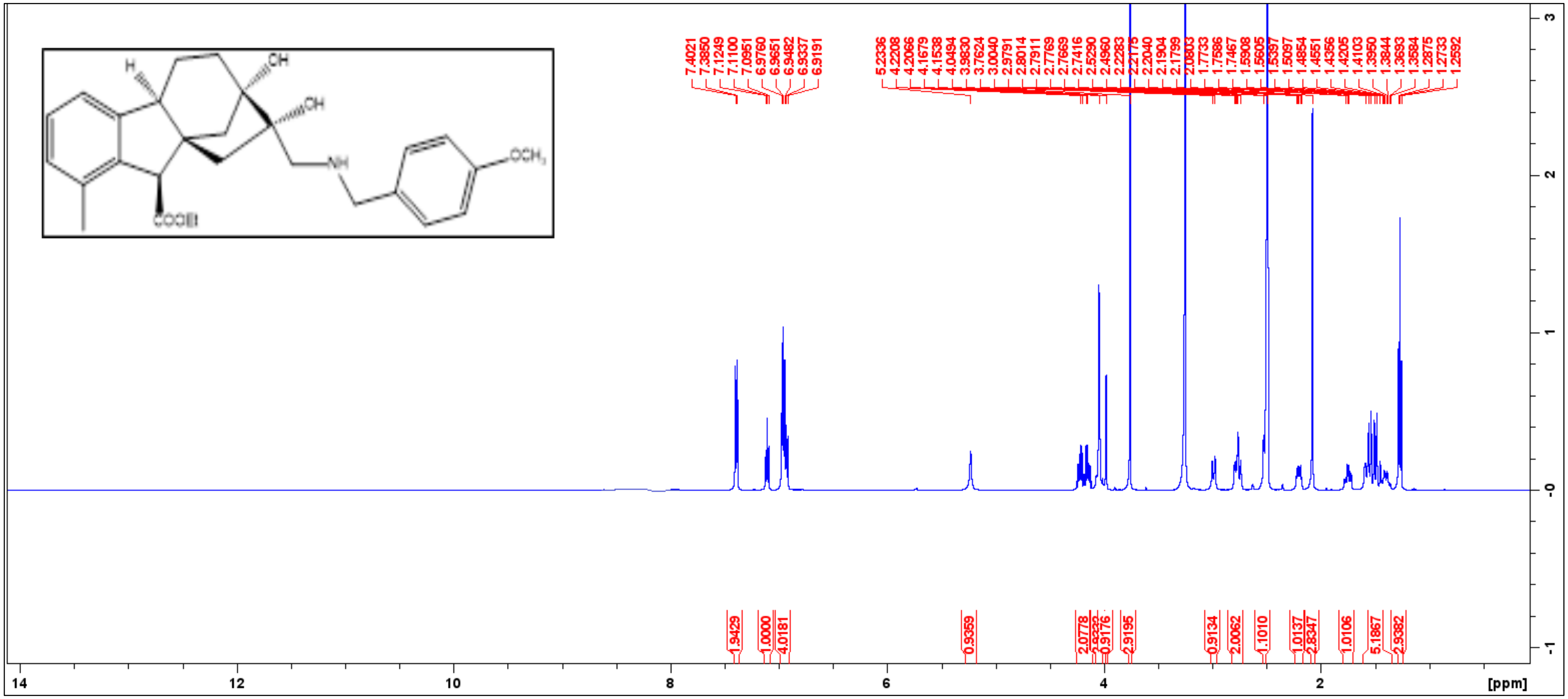

Figure S 27: ^{13}C-NMR (JMOD) of compound 11

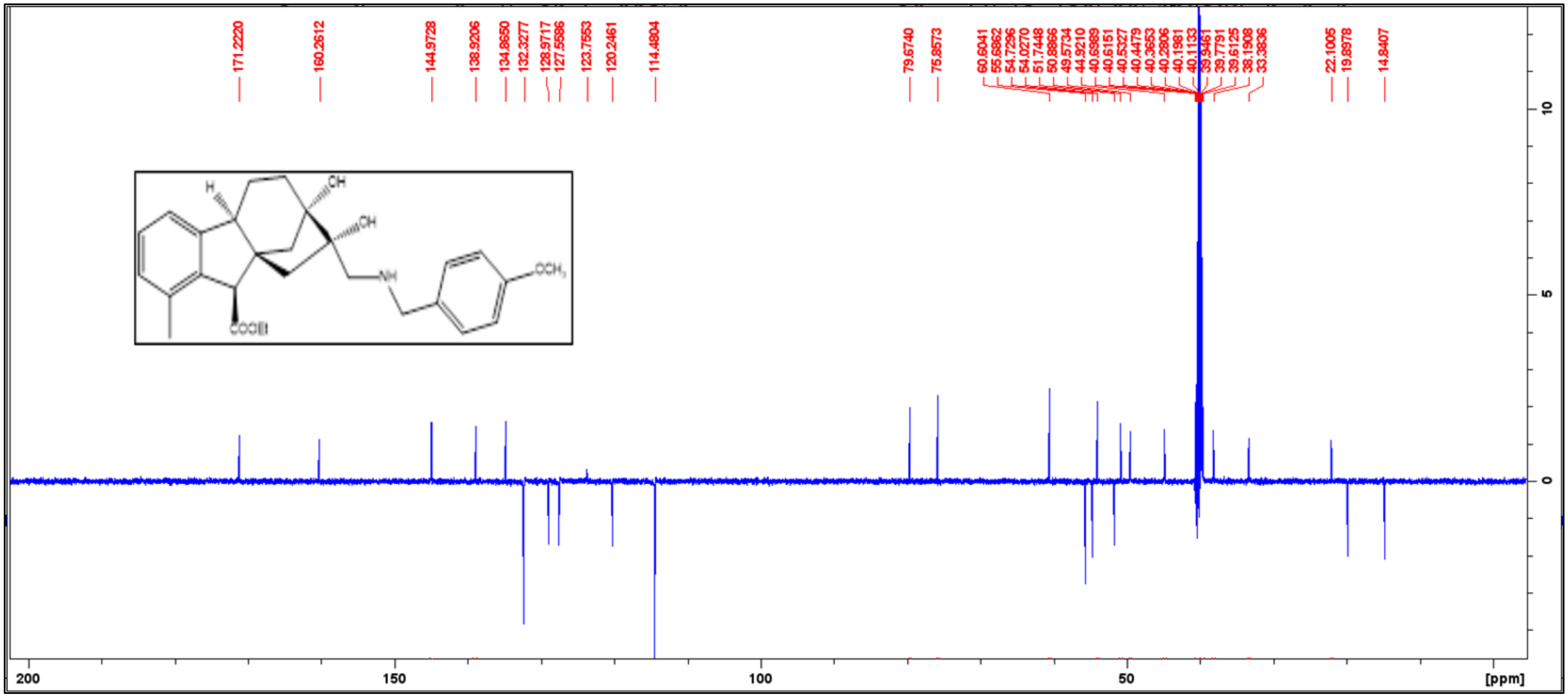

Figure S 28: ^{1}H-NMR of compound 12

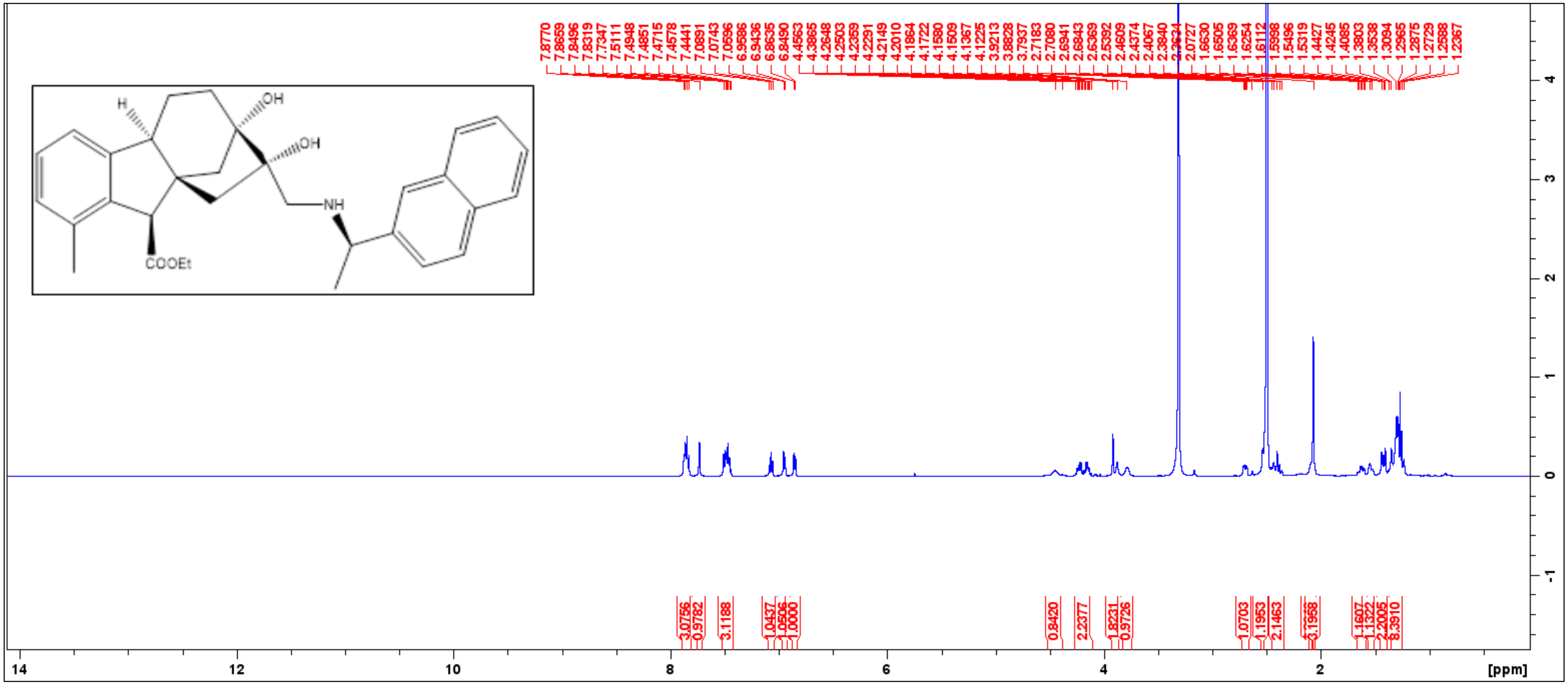

Figure S 29: ^{13}C-NMR (JMOD) of compound 12

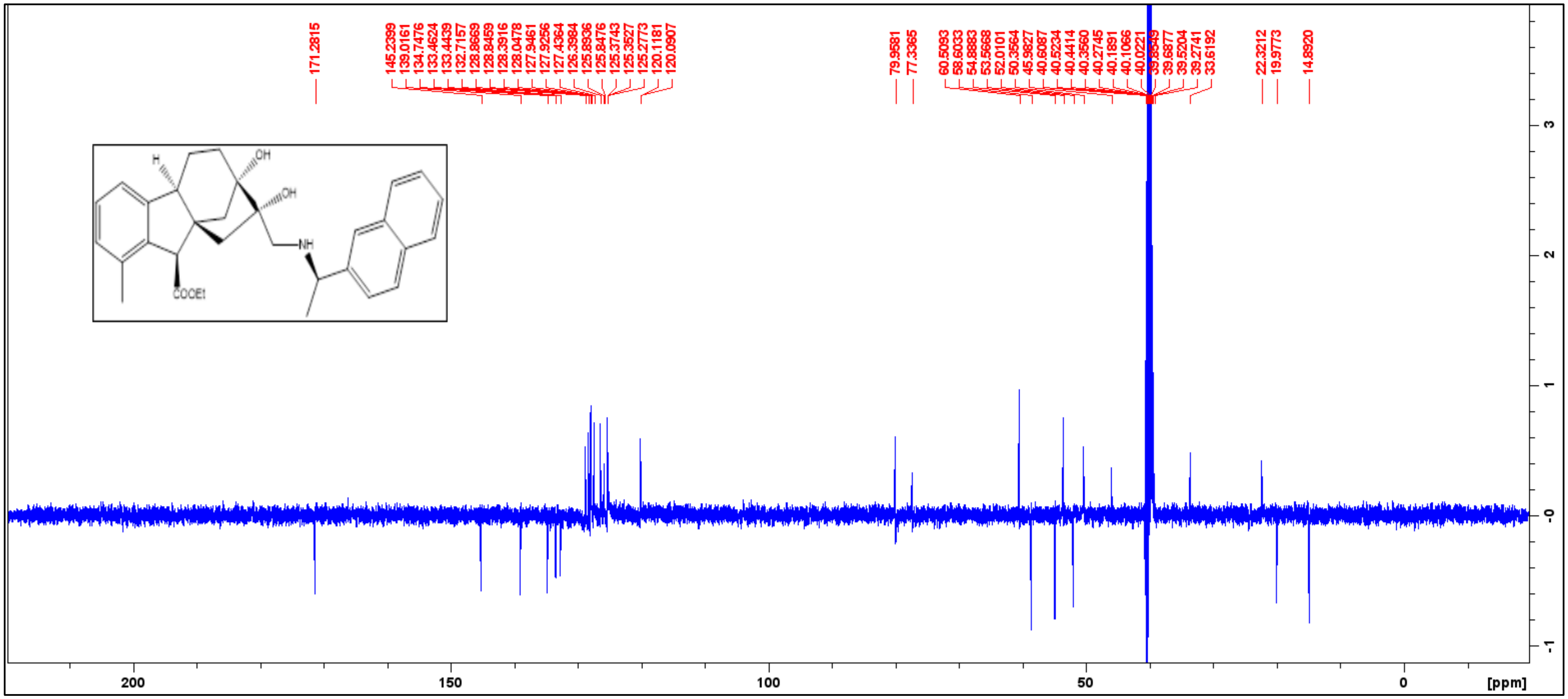

Figure S 30: HSQC of compound 12

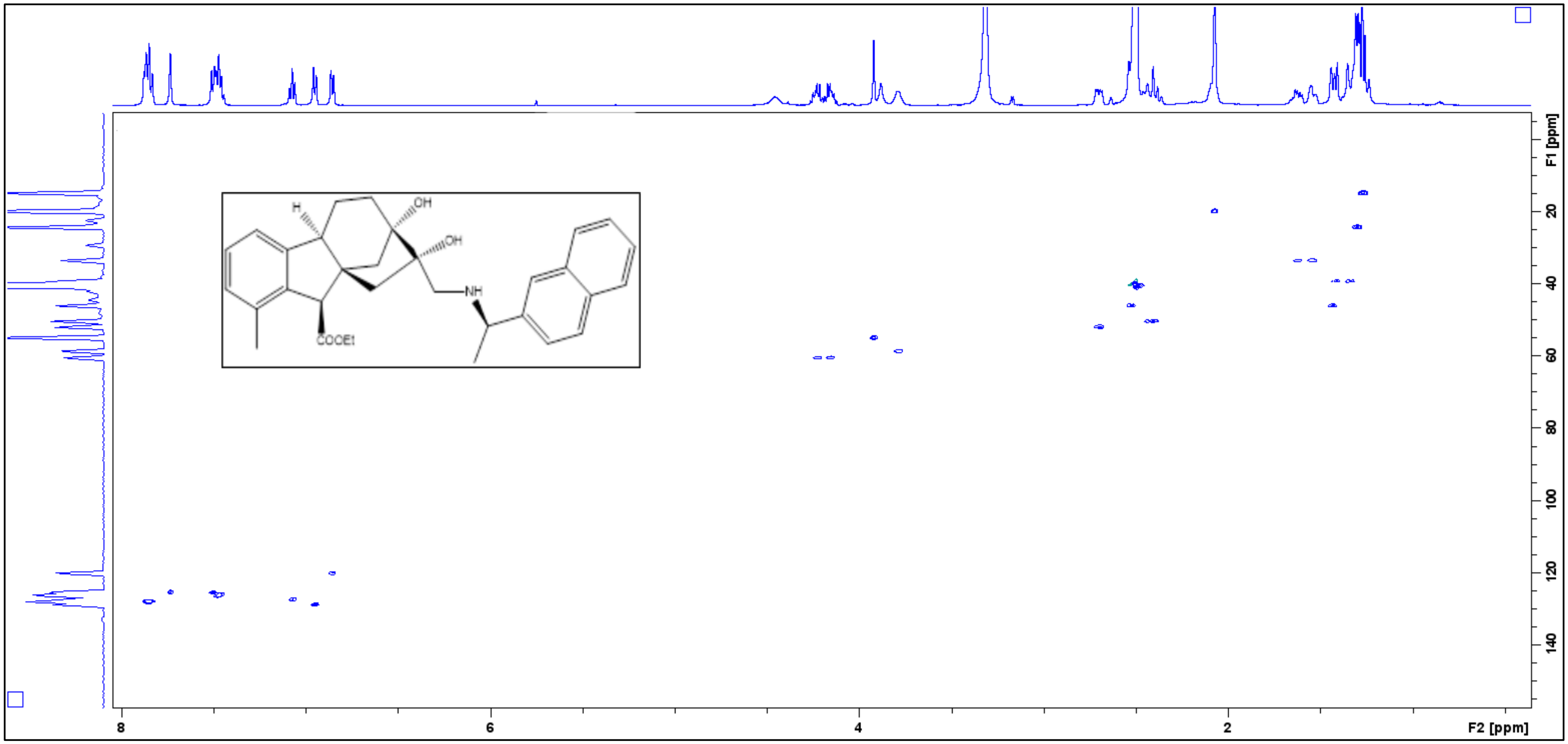

Figure S 31: HMBC of compound 12

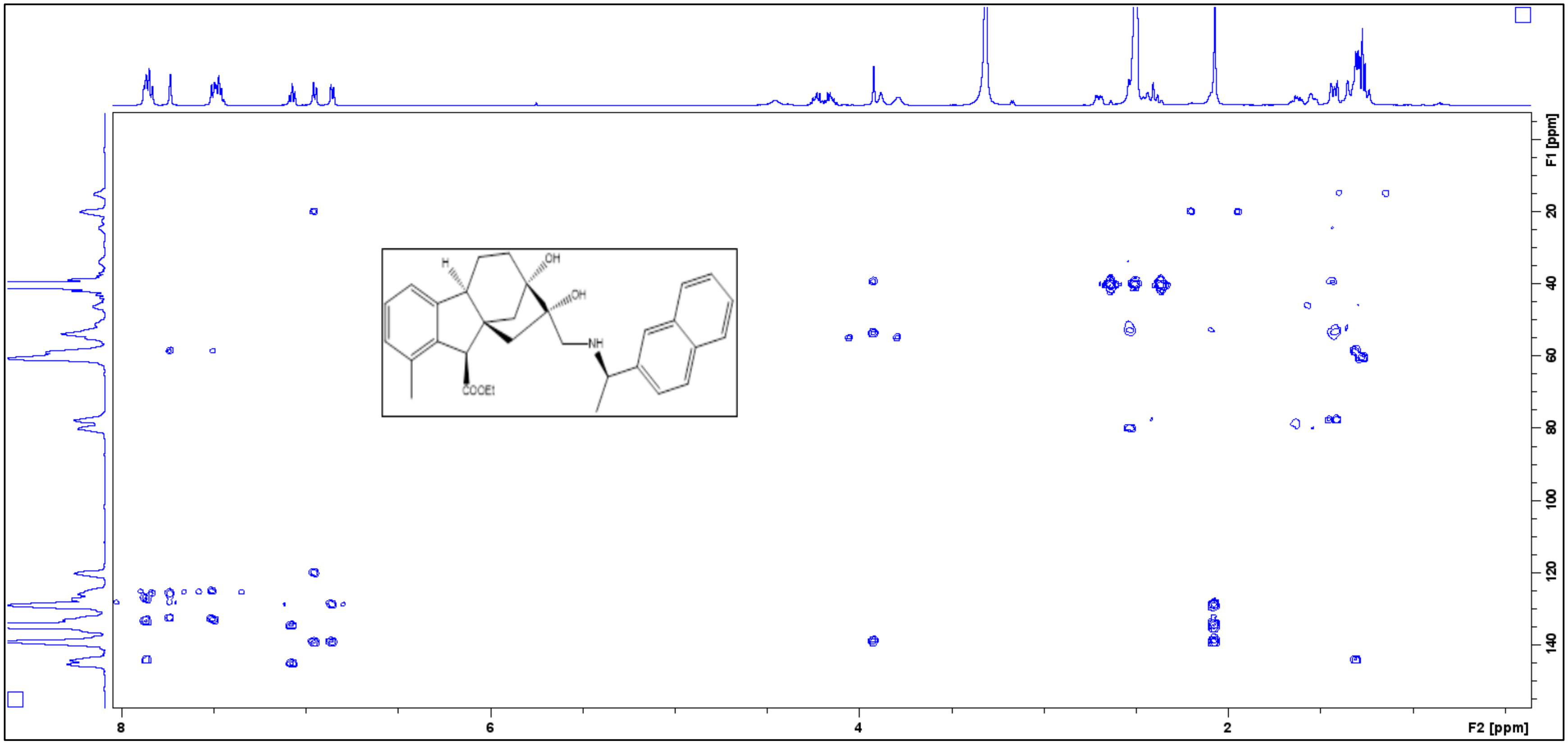

Figure S 32: ^{1}H-NMR of compound 13

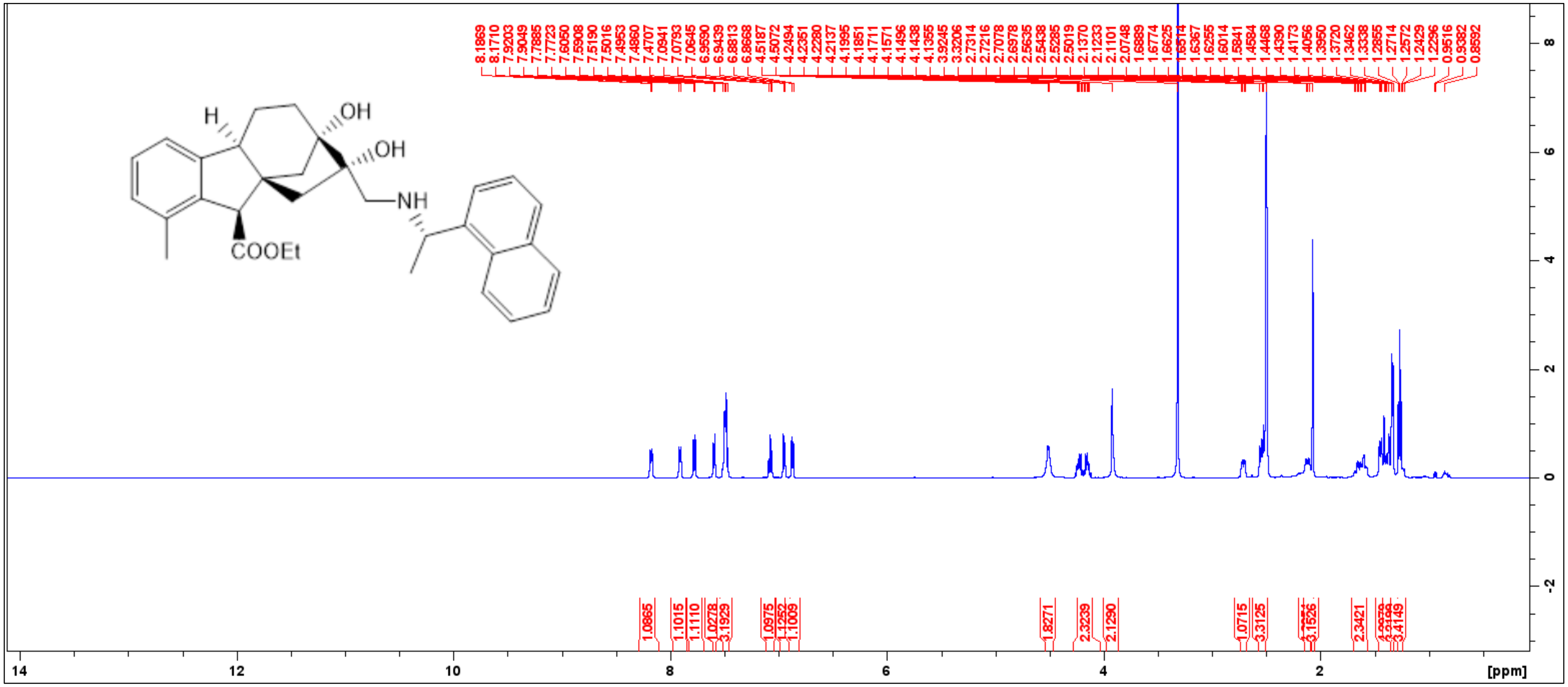

Figure S 33: ^{13}C-NMR (JMOD) of compound 13

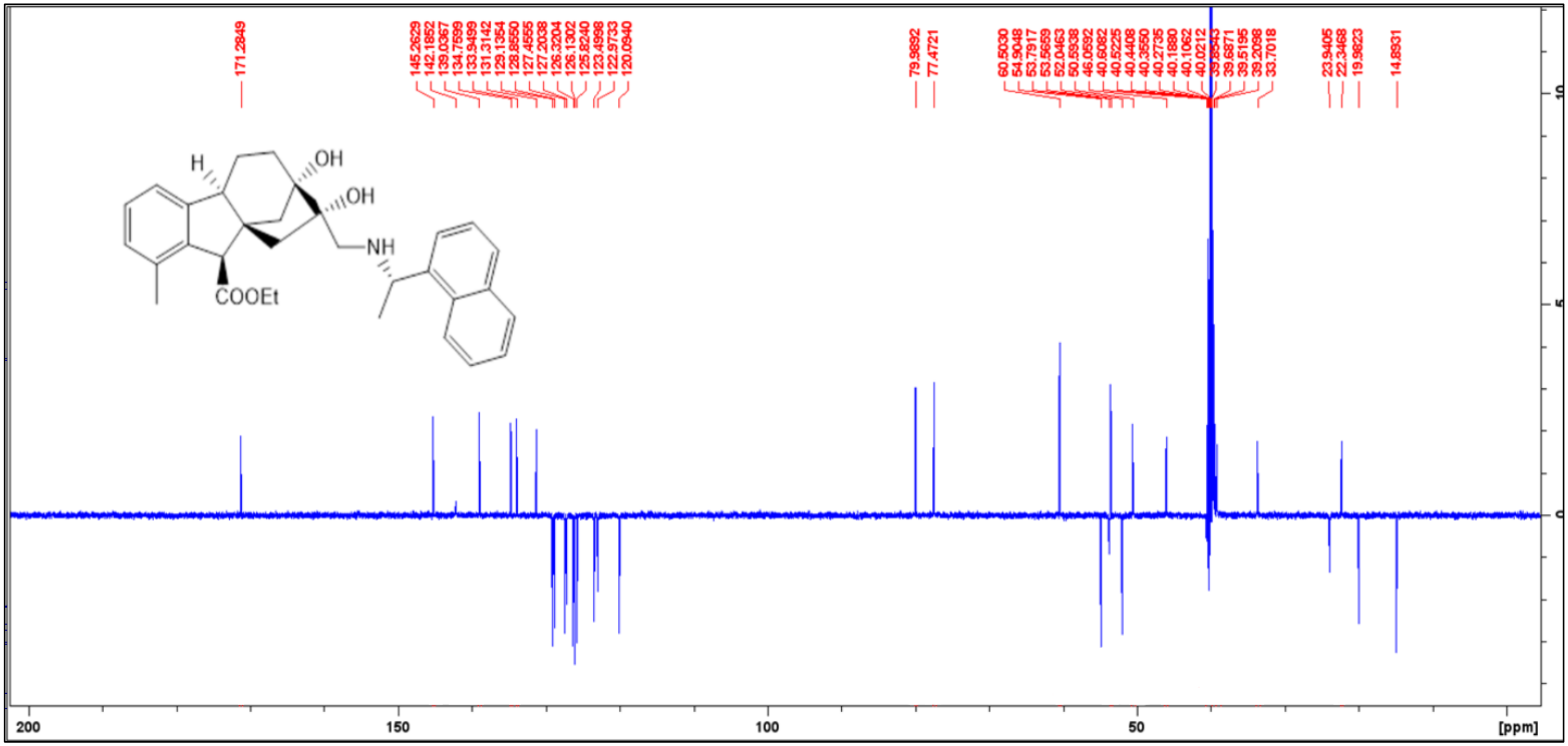

Figure S 34: ^{1}H-NMR of compound 14

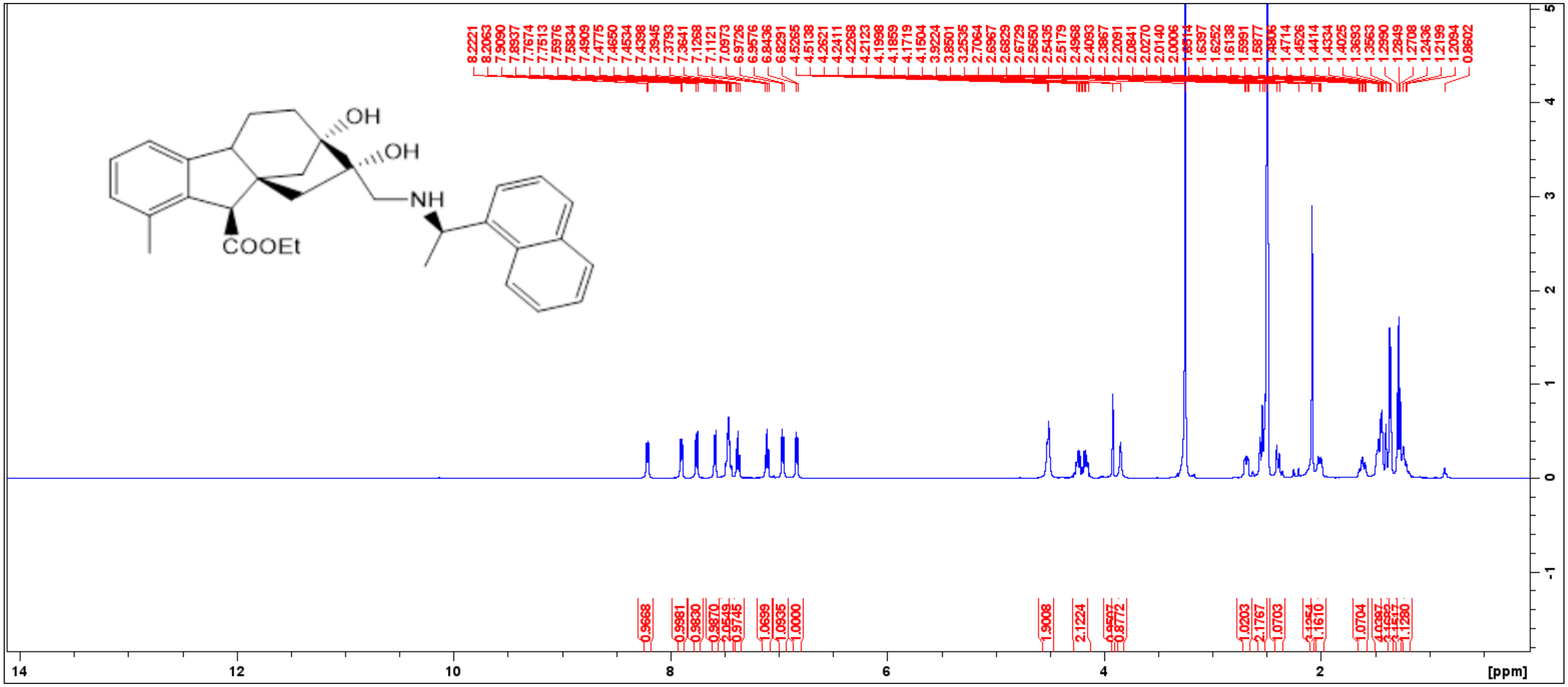

Figure S 35: ^{13}C-NMR (JMOD) of compound 14

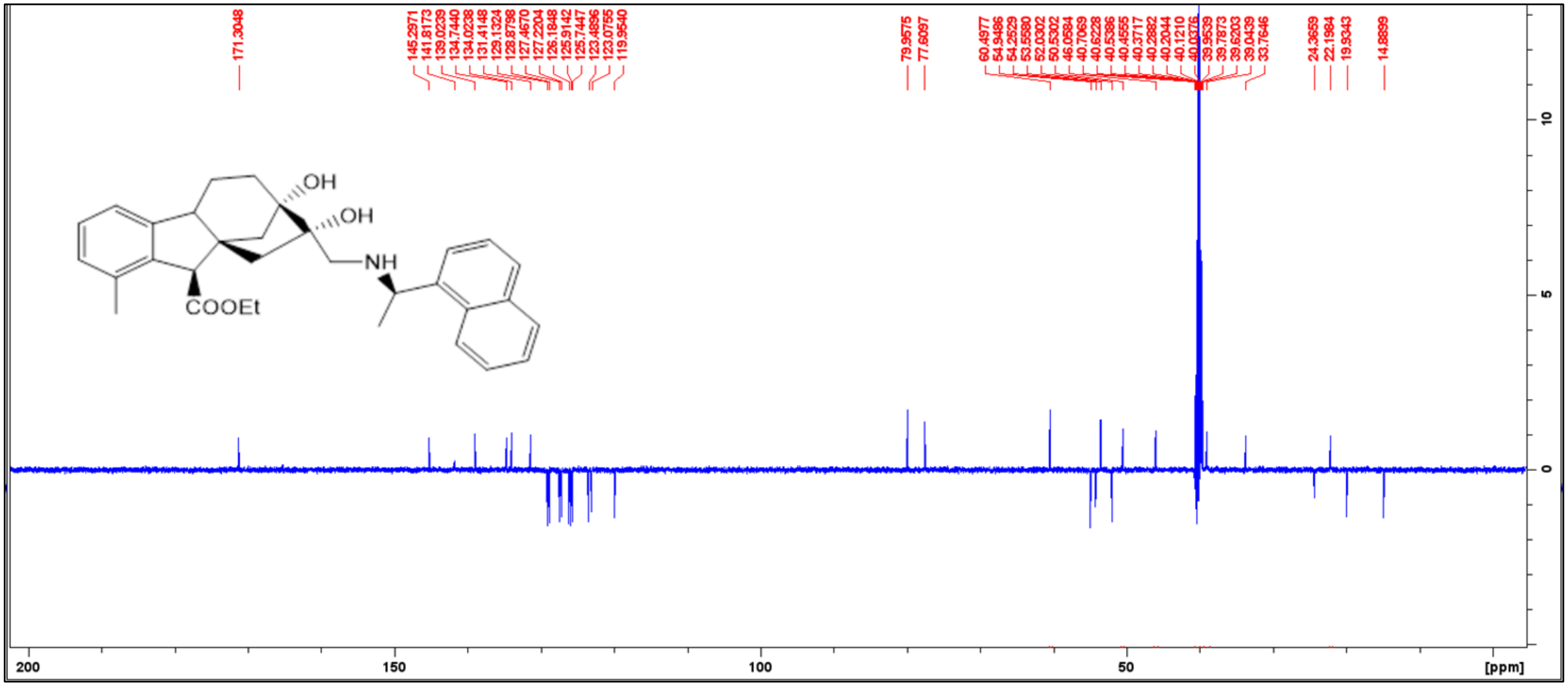

Figure S 36: ^{1}H-NMR of compound 15

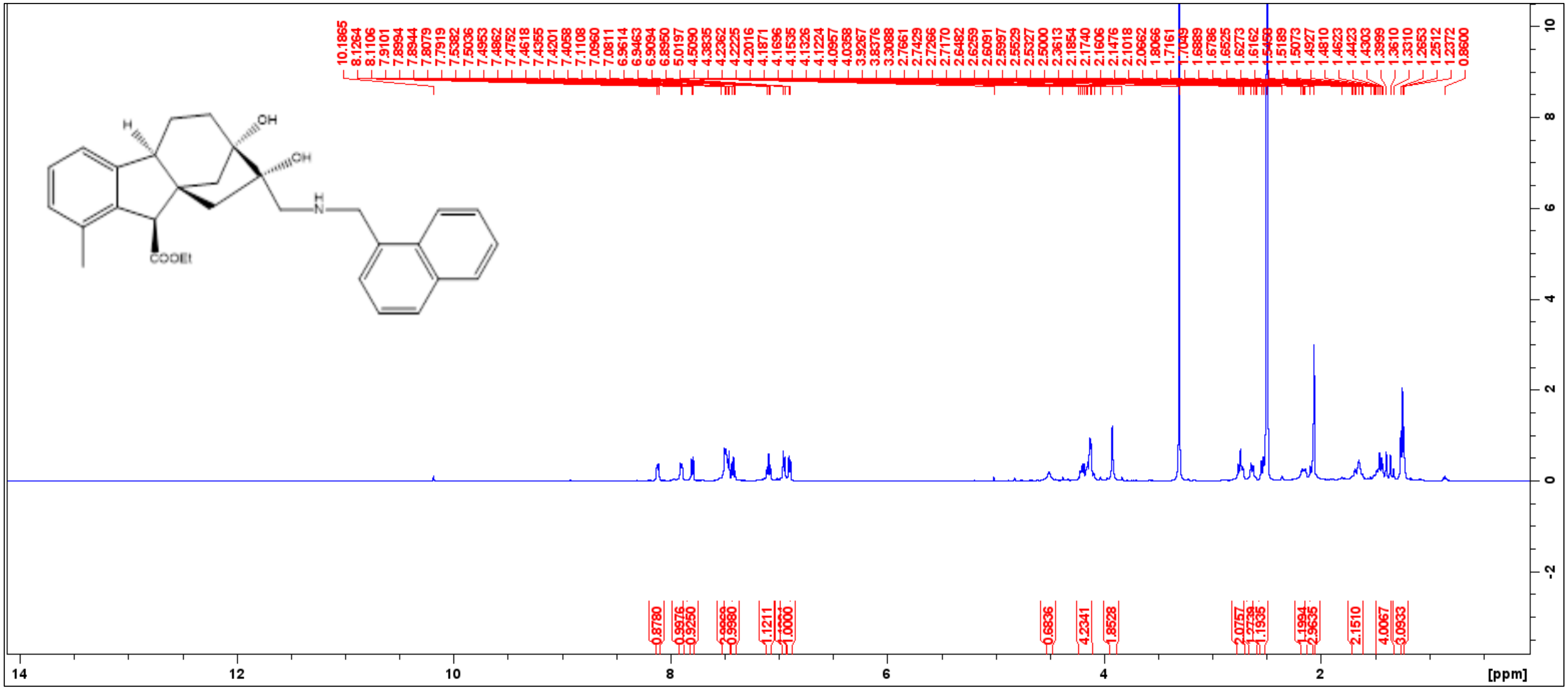

Figure S 37: ^{13}C-NMR (JMOD) of compound 15

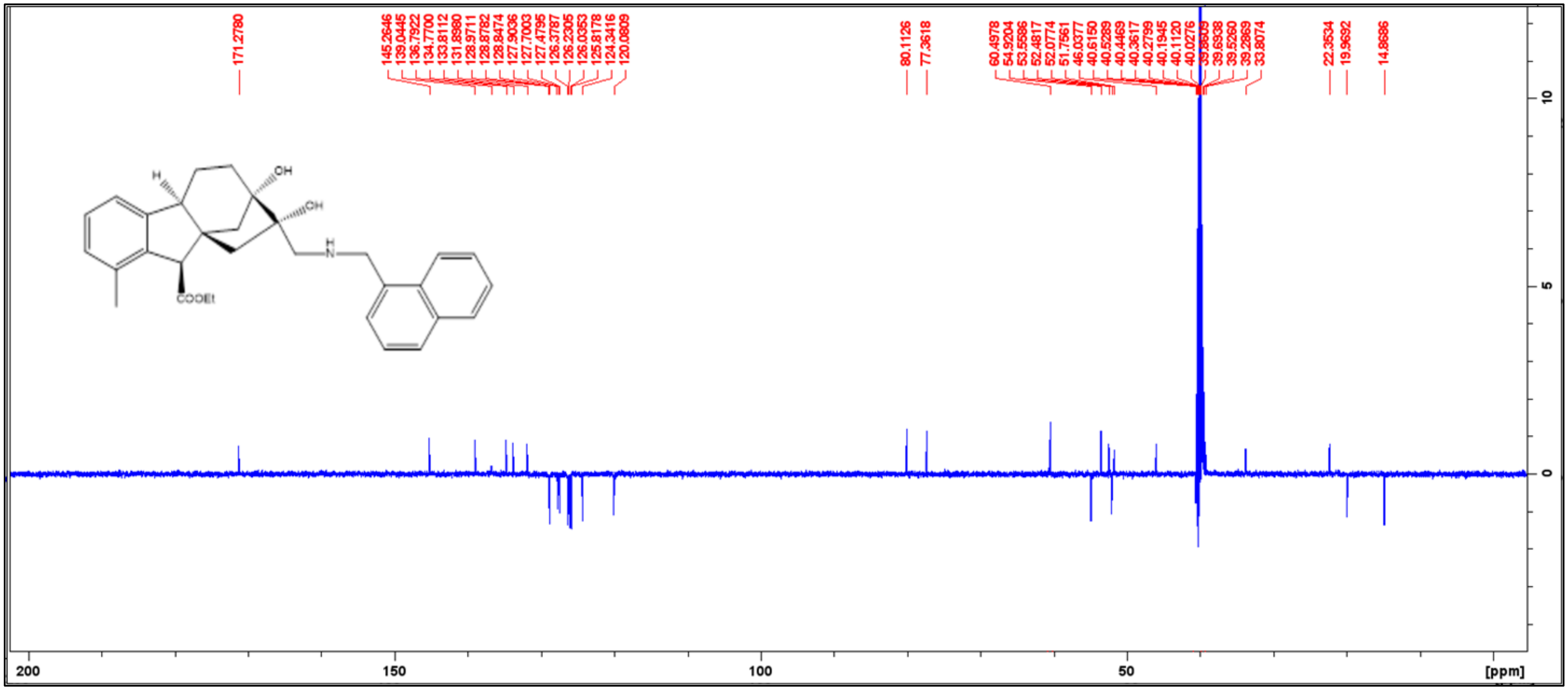

Figure S 38: ^{1}H-NMR of compound 16

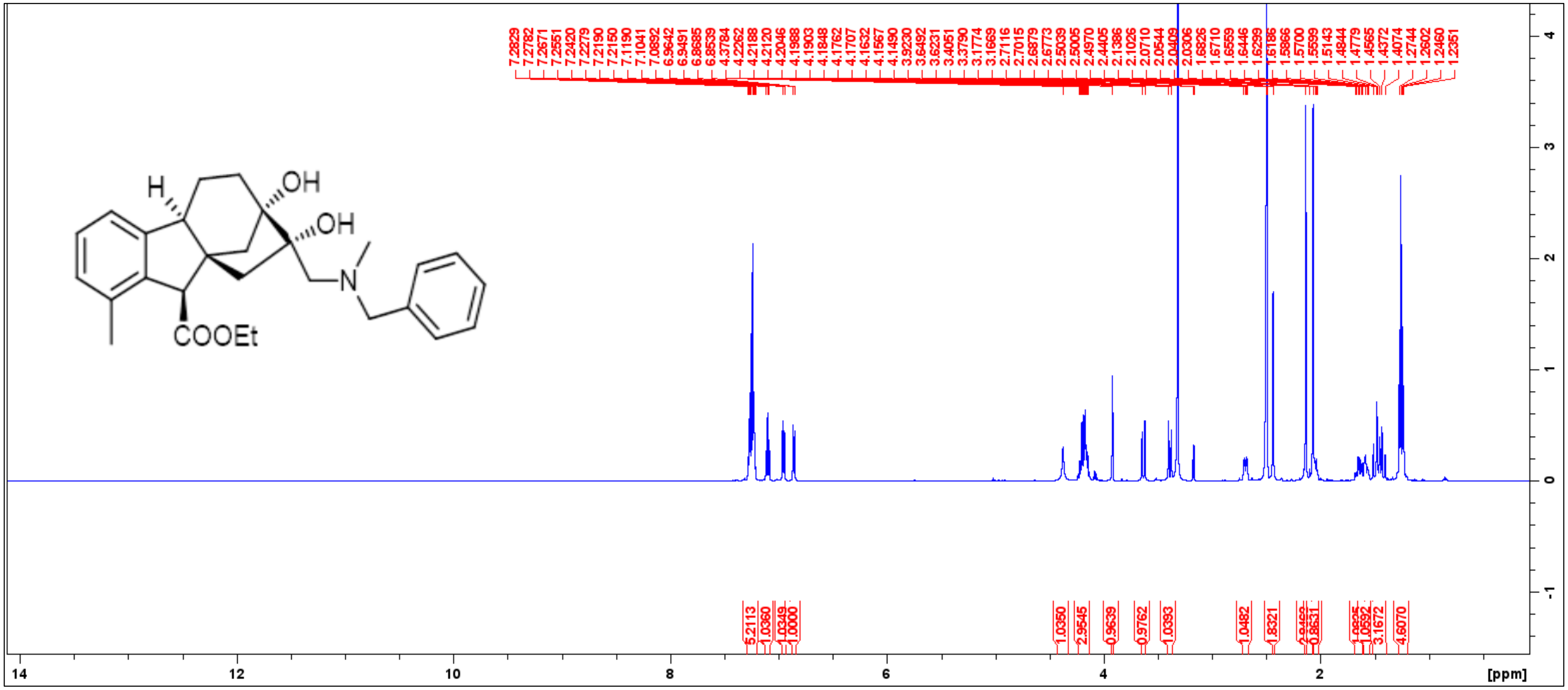

Figure S 39: ^{13}C-NMR (JMOD) of compound 16

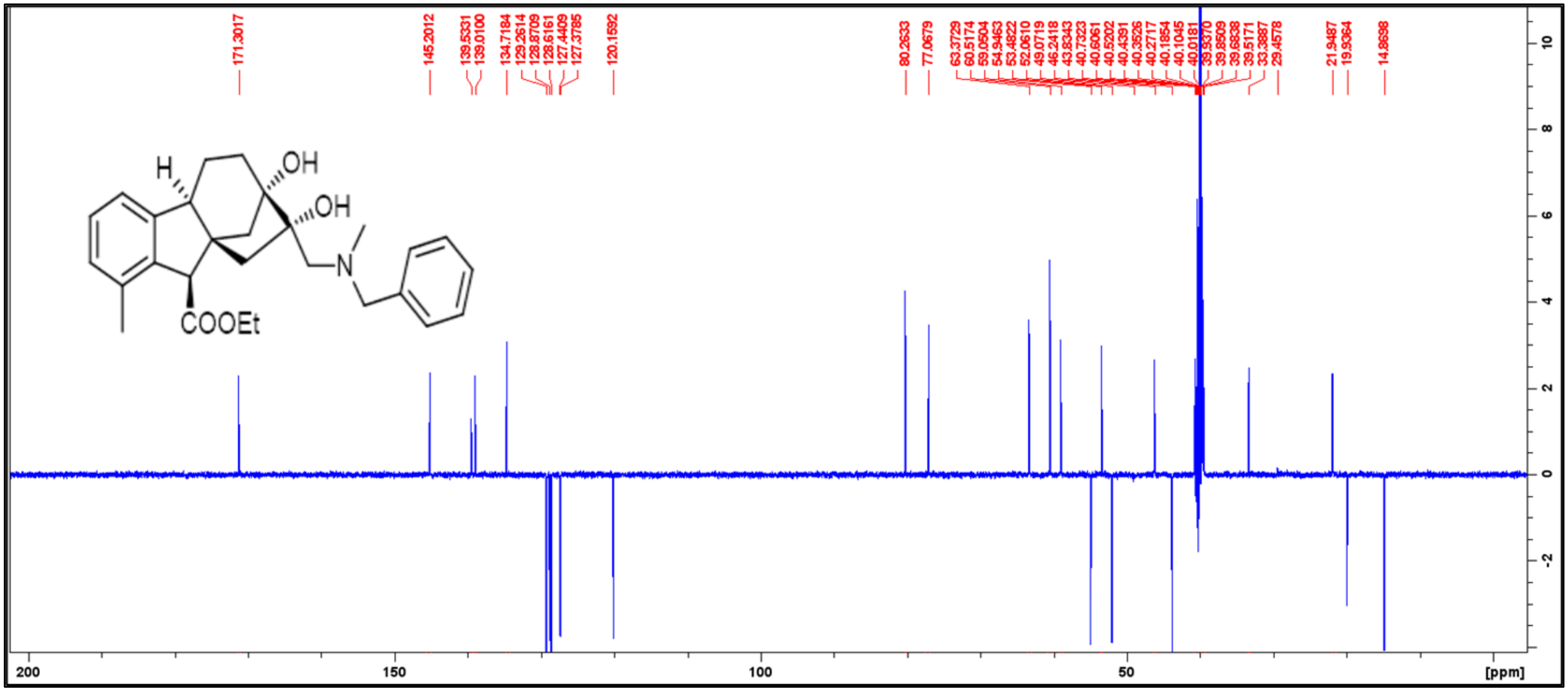

Figure S 40: ^{1}H-NMR of compound 17

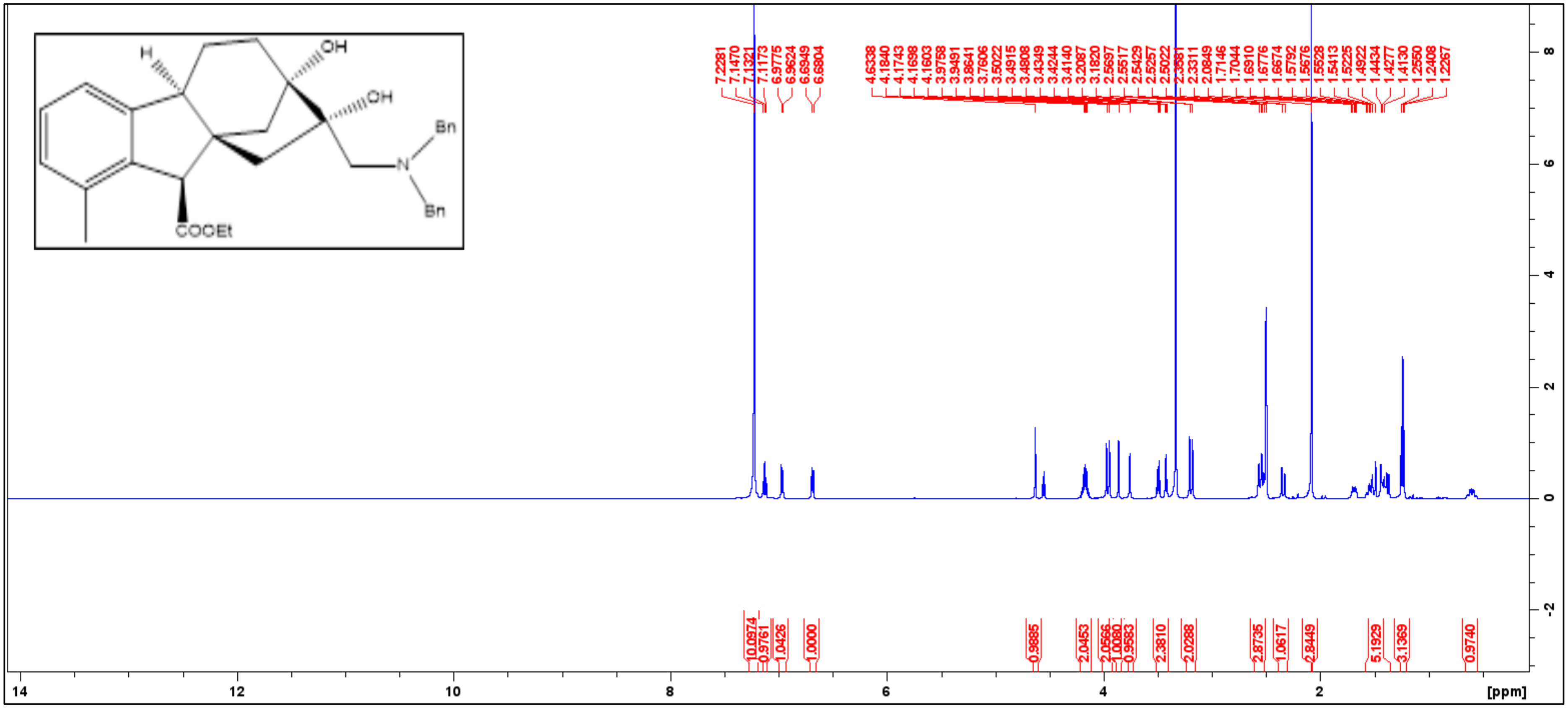

Figure S 41: ^{13}C-NMR (JMOD) of compound 17

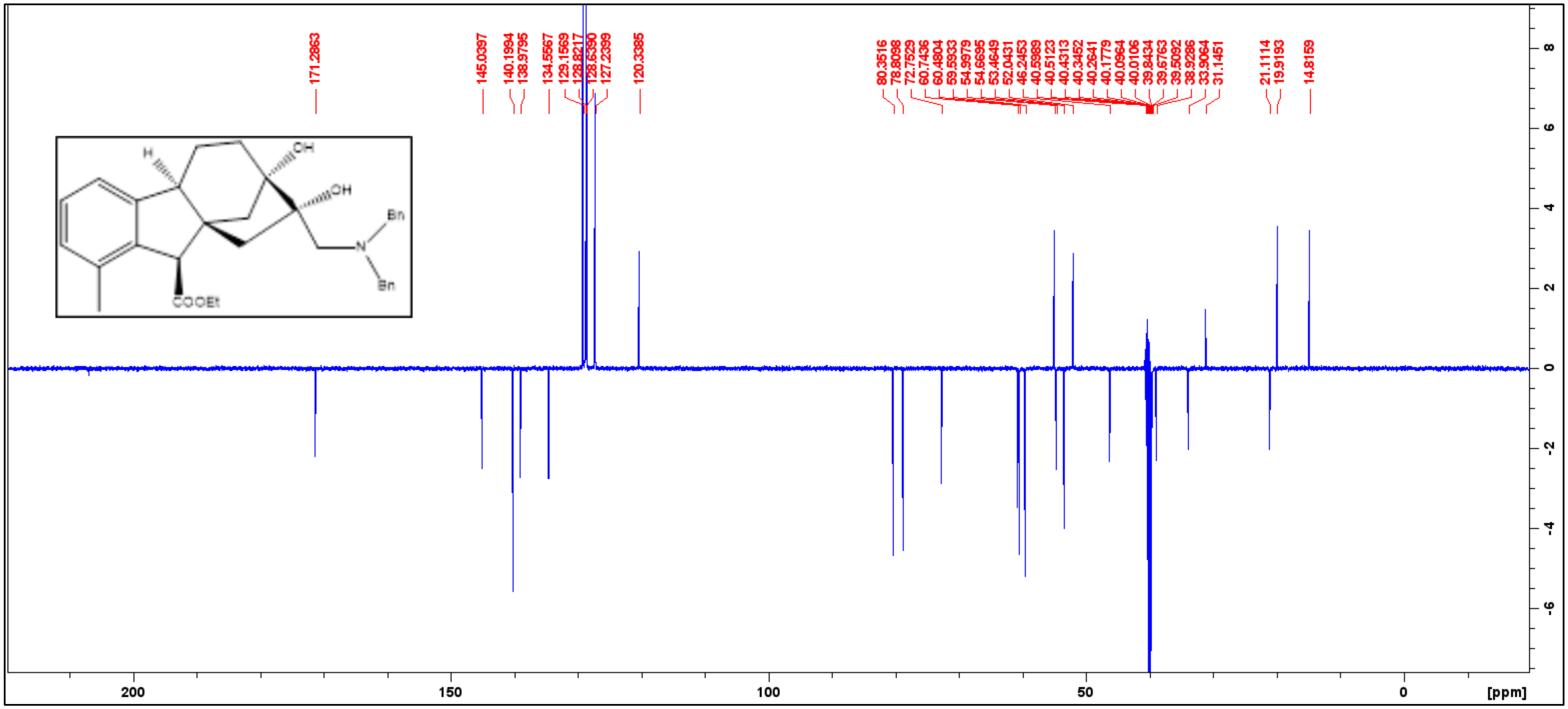

Figure S 42: ^{1}H-NMR of compound 18

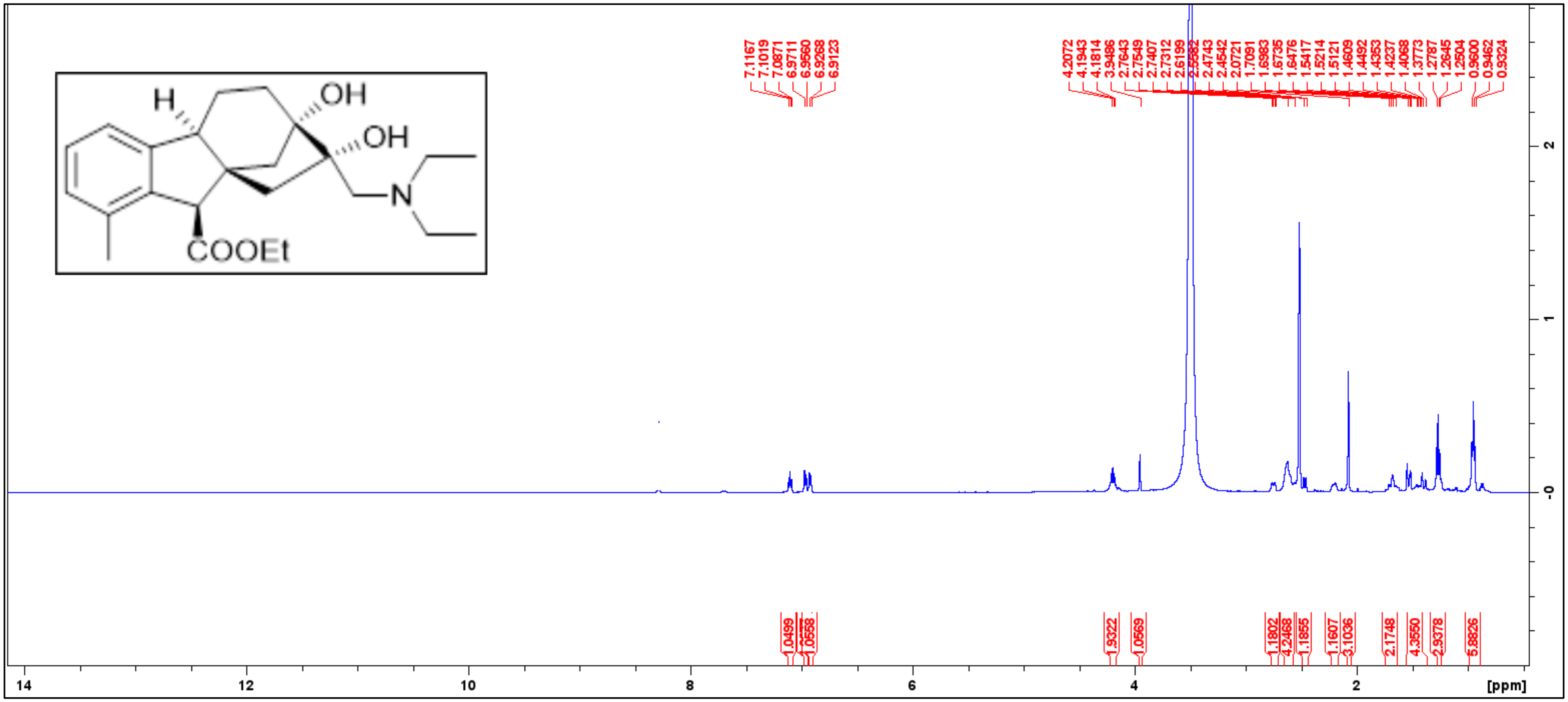

Figure S 43: ^{13}C-NMR (JMOD) of compound 18

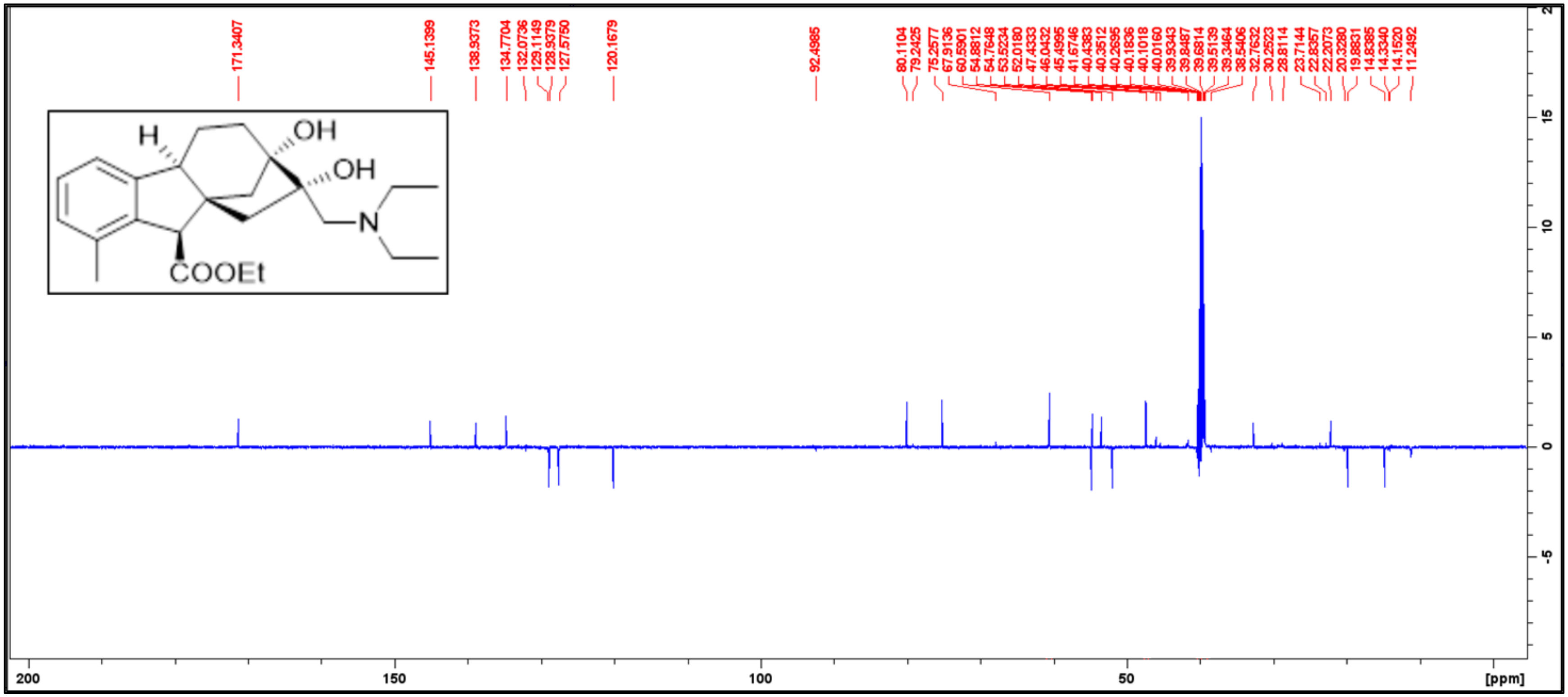

Figure S 44: ^{1}H-NMR of compound 19

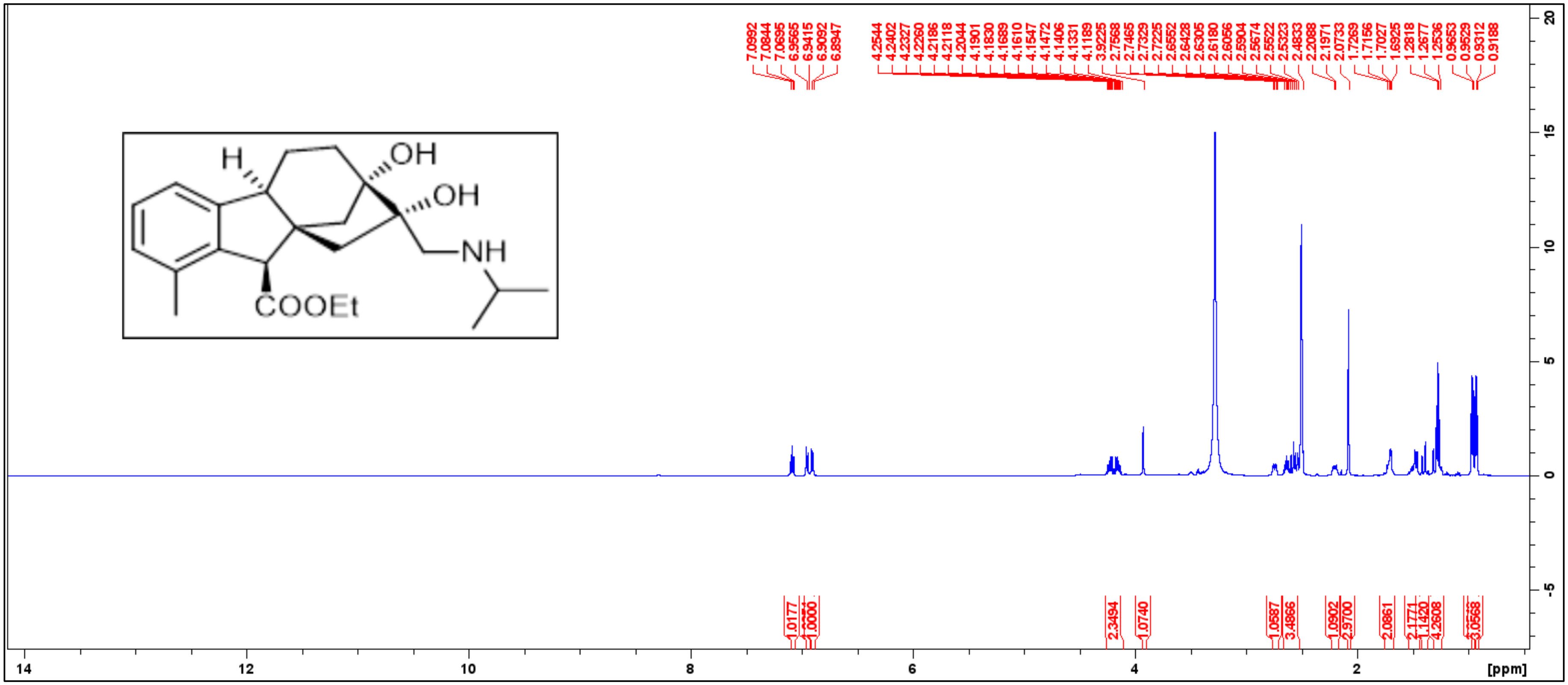

Figure S 45: ^{13}C-NMR (JMOD) of compound 19

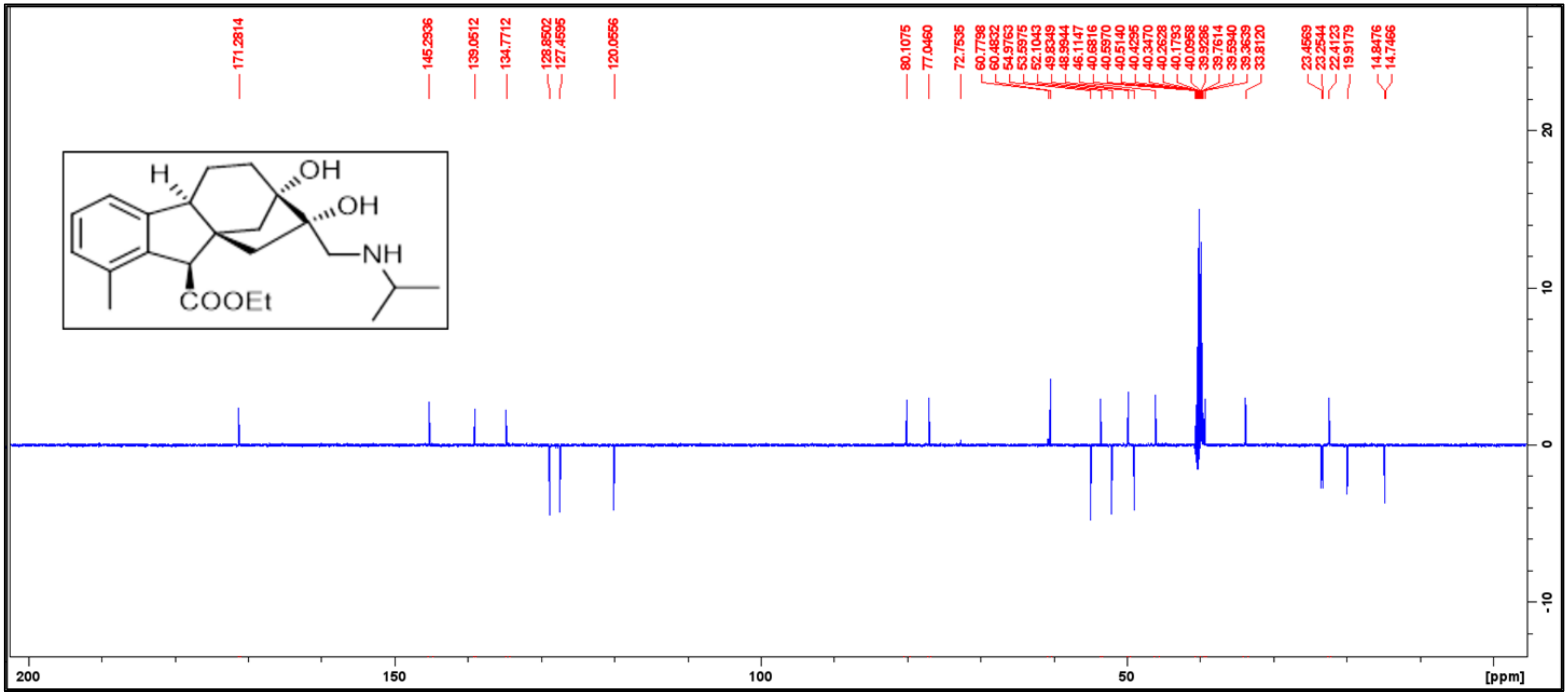

Figure S 46: ^{1}H-NMR of compound 20

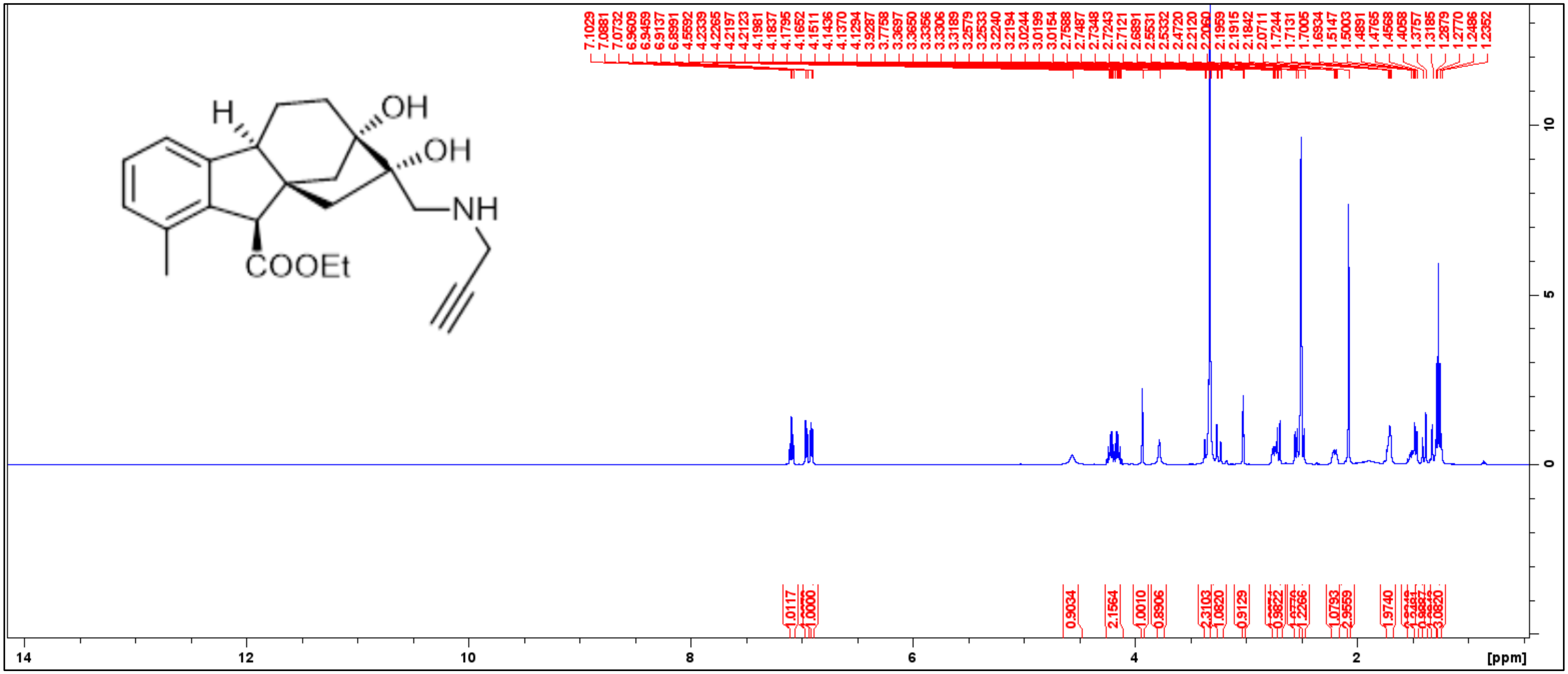

Figure S 47: ^{13}C-NMR (JMOD) of compound 20

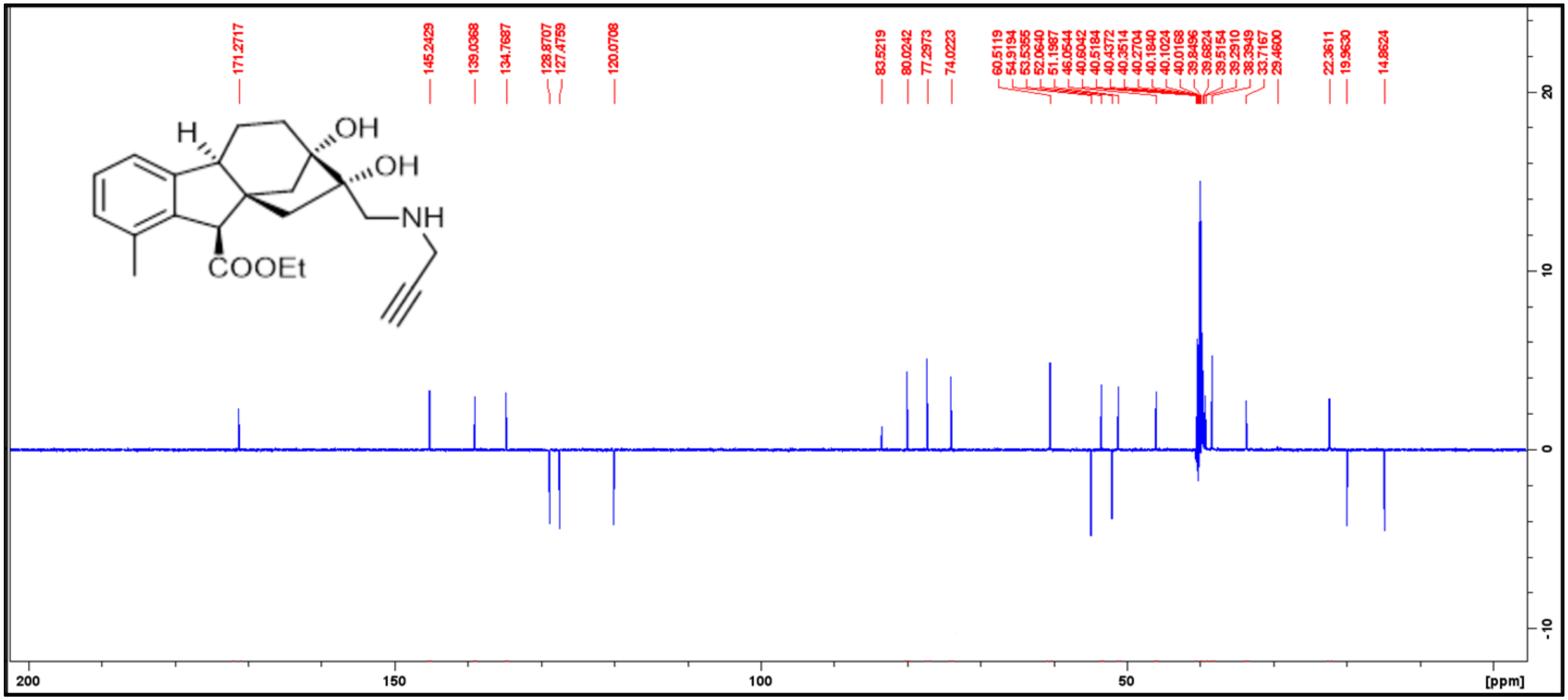

Figure S 48: ^{1}H-NMR of compound 21

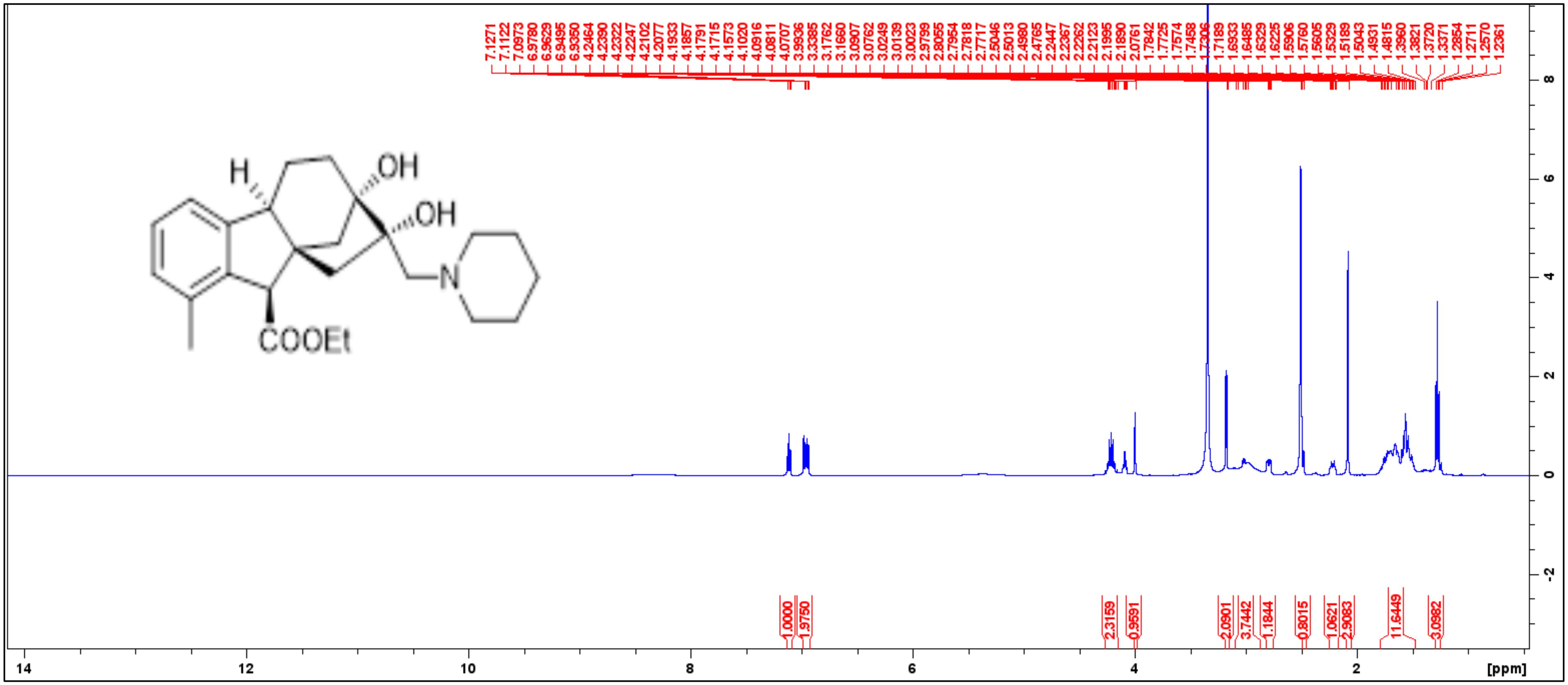

Figure S 49: ^{13}C-NMR (JMOD) of compound 21

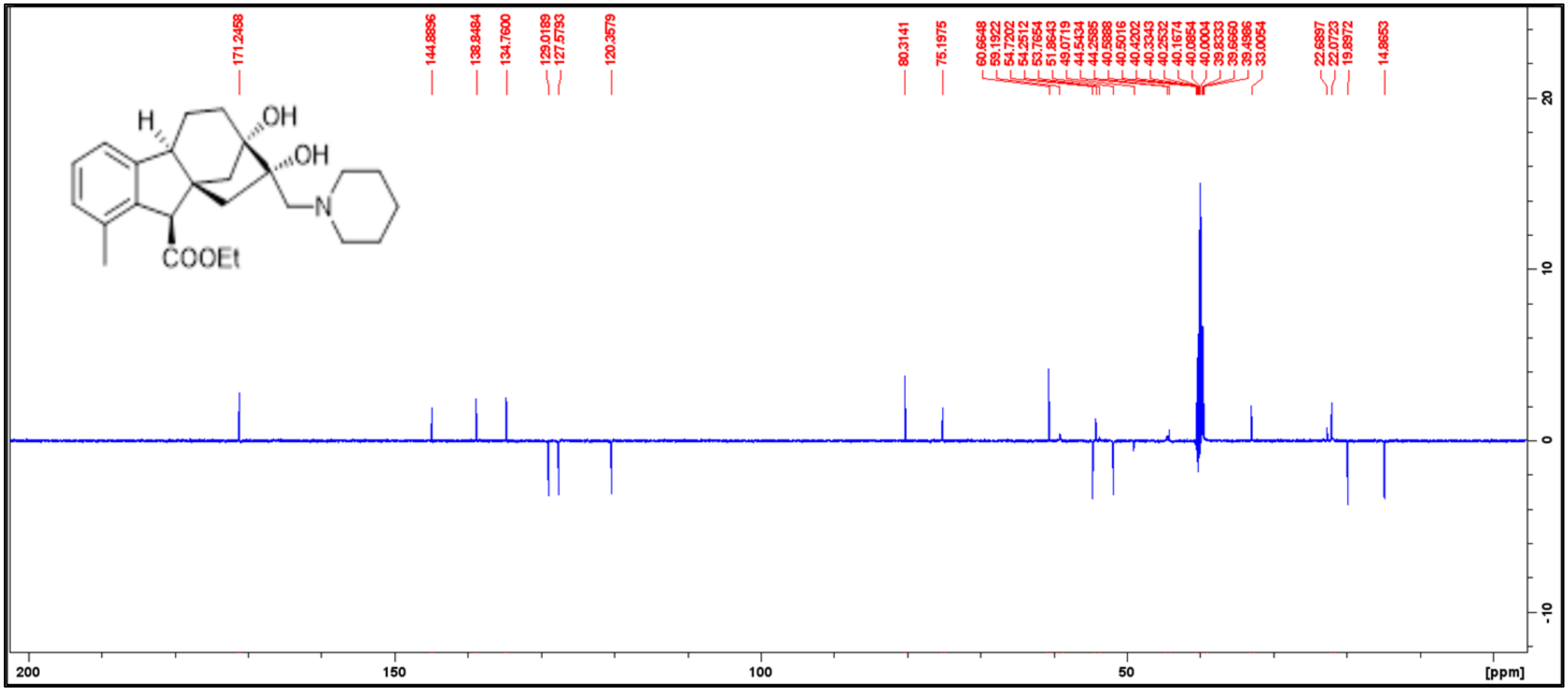

Figure S 50: ^{1}H-NMR of compound 22

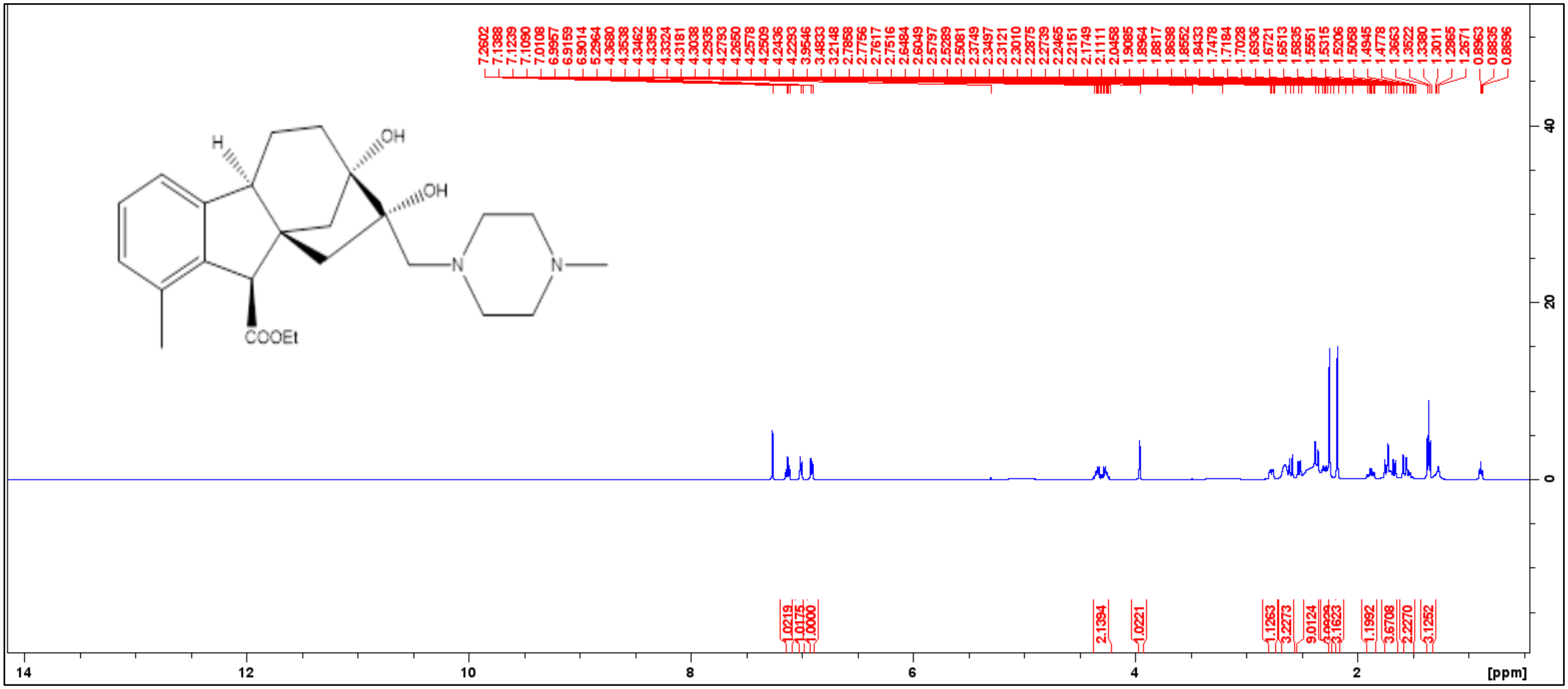

Figure S 51: ^{13}C-NMR (JMOD) of compound 22

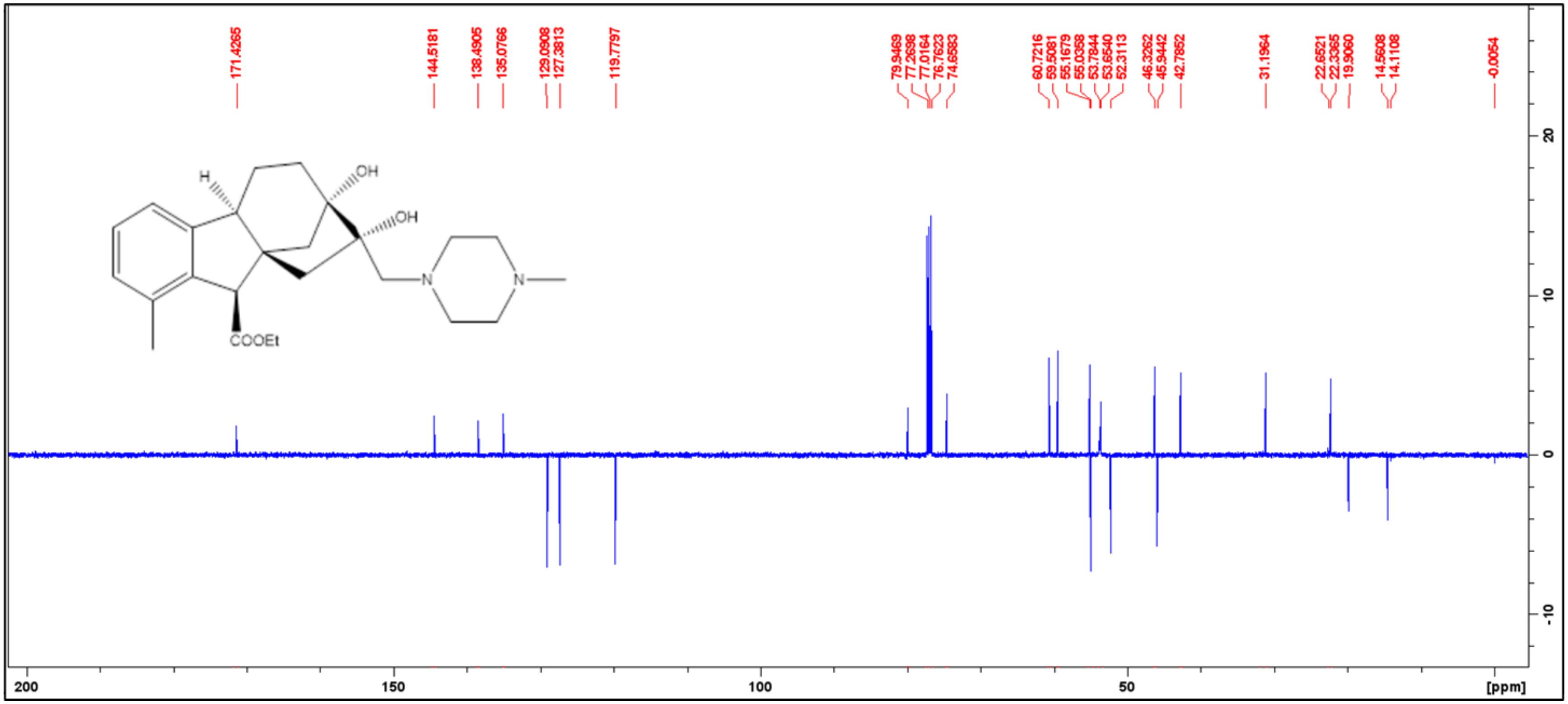

Figure S 52: ^{1}H-NMR of compound 23

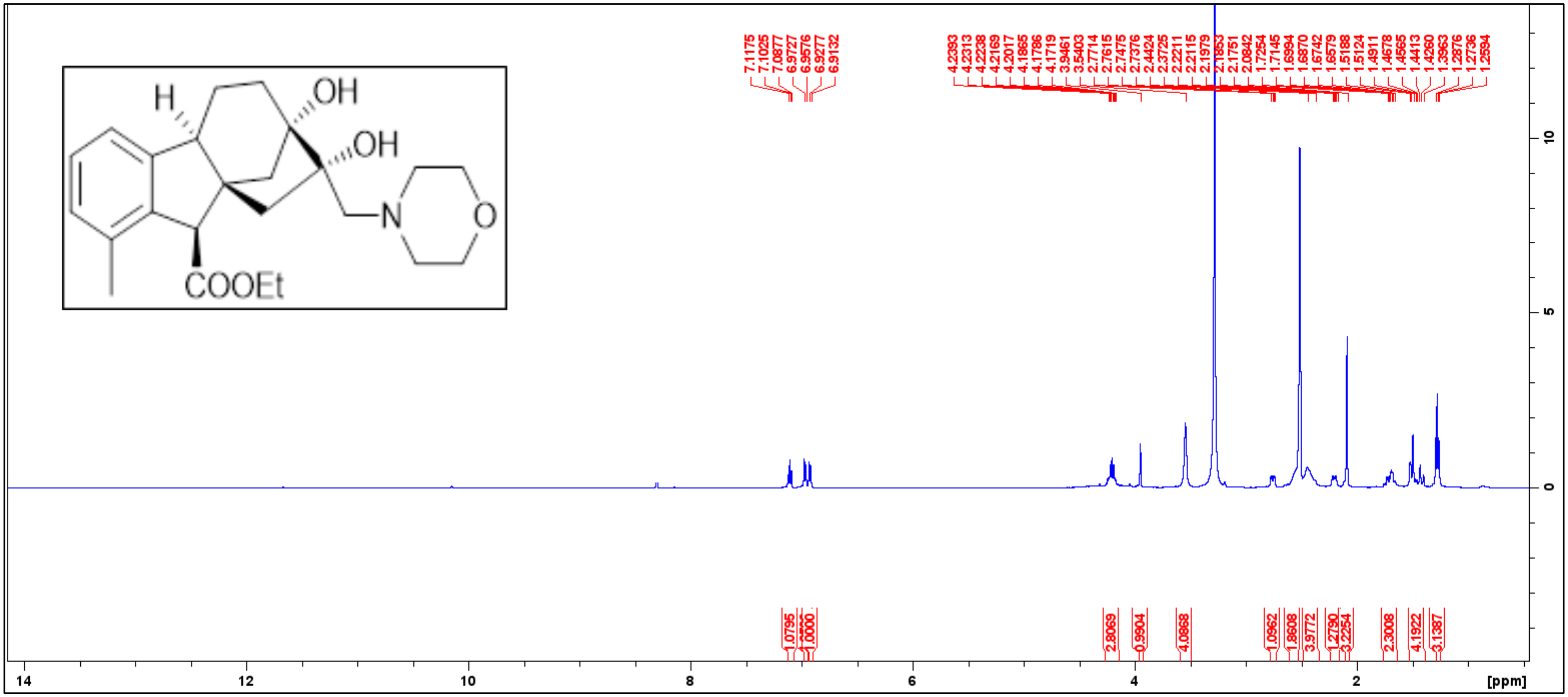

Figure S 53: ^{13}C-NMR (JMOD) of compound 23

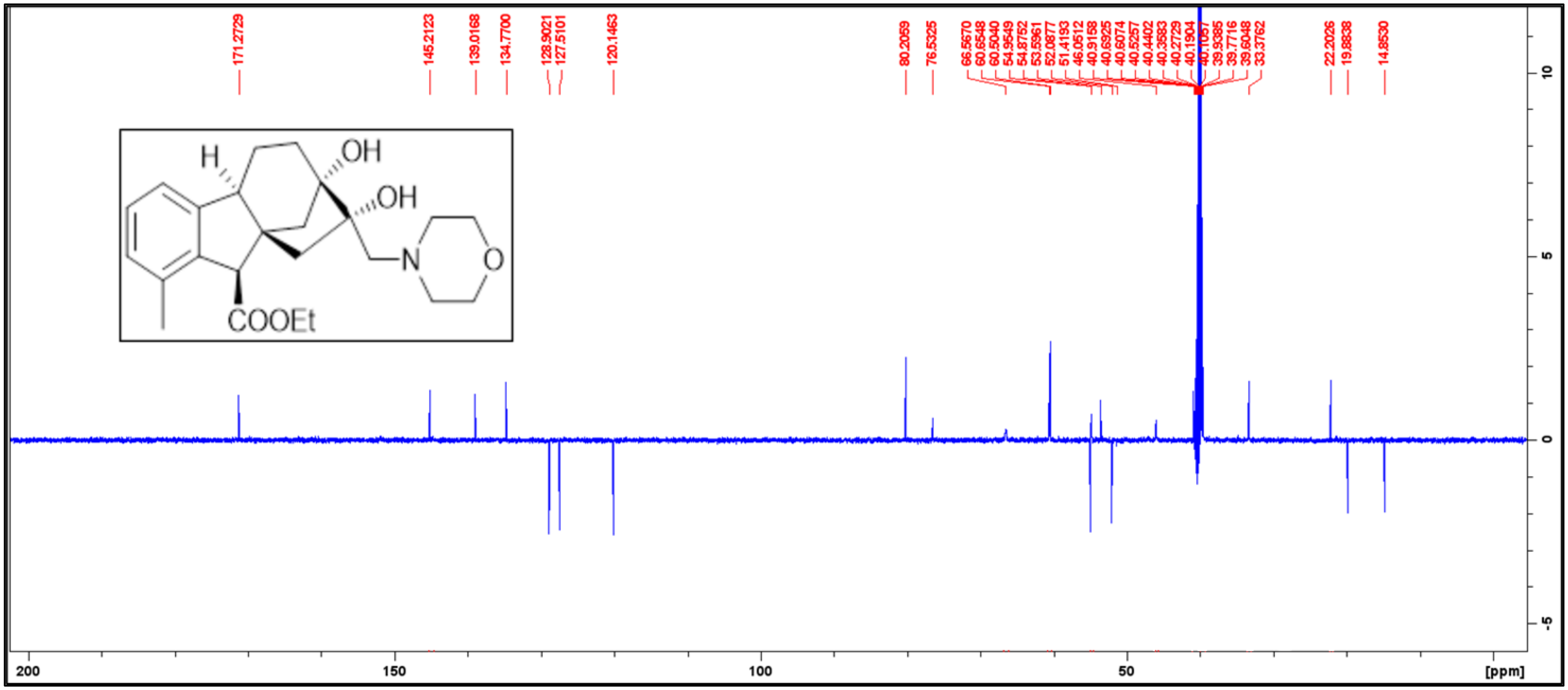

Figure S 54: ^{1}H-NMR of compound 24

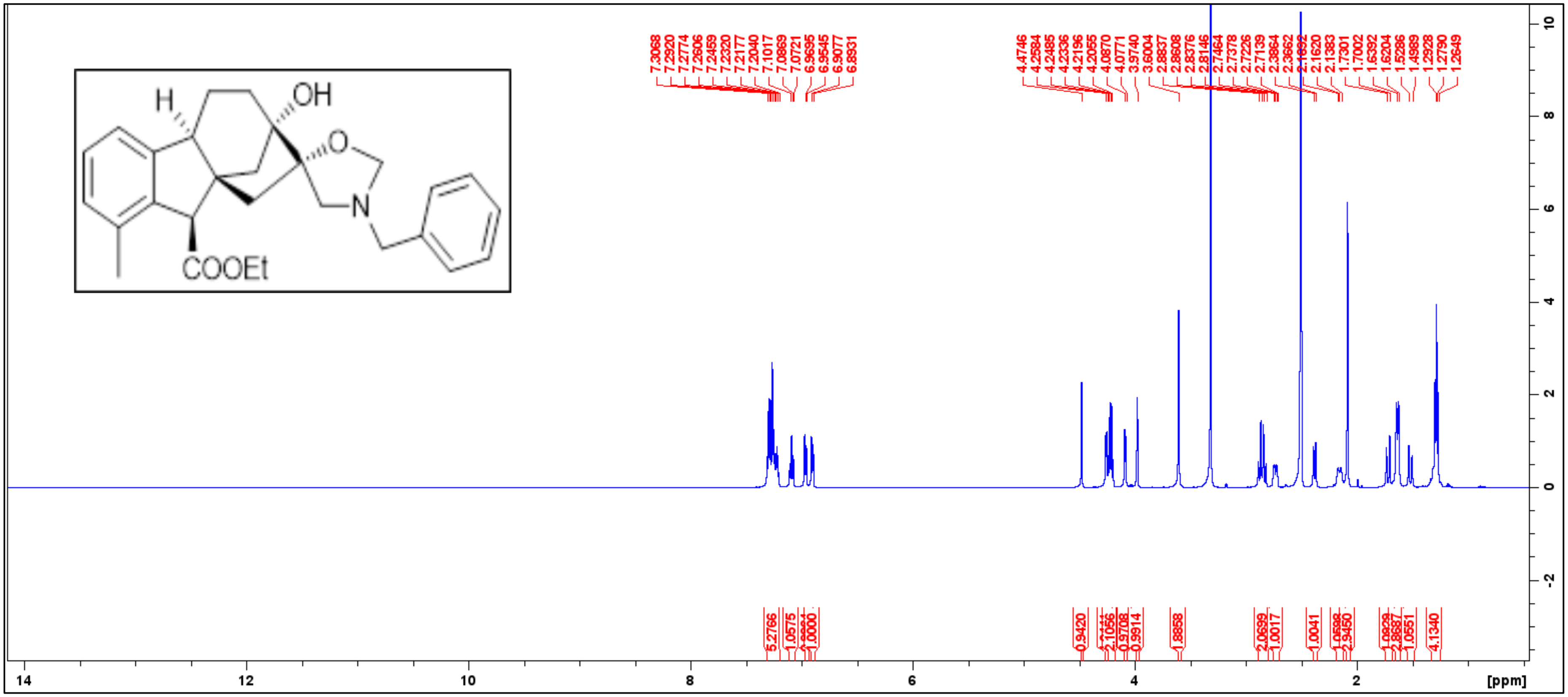

Figure S 55: ^{13}C-NMR (JMOD) of compound 24

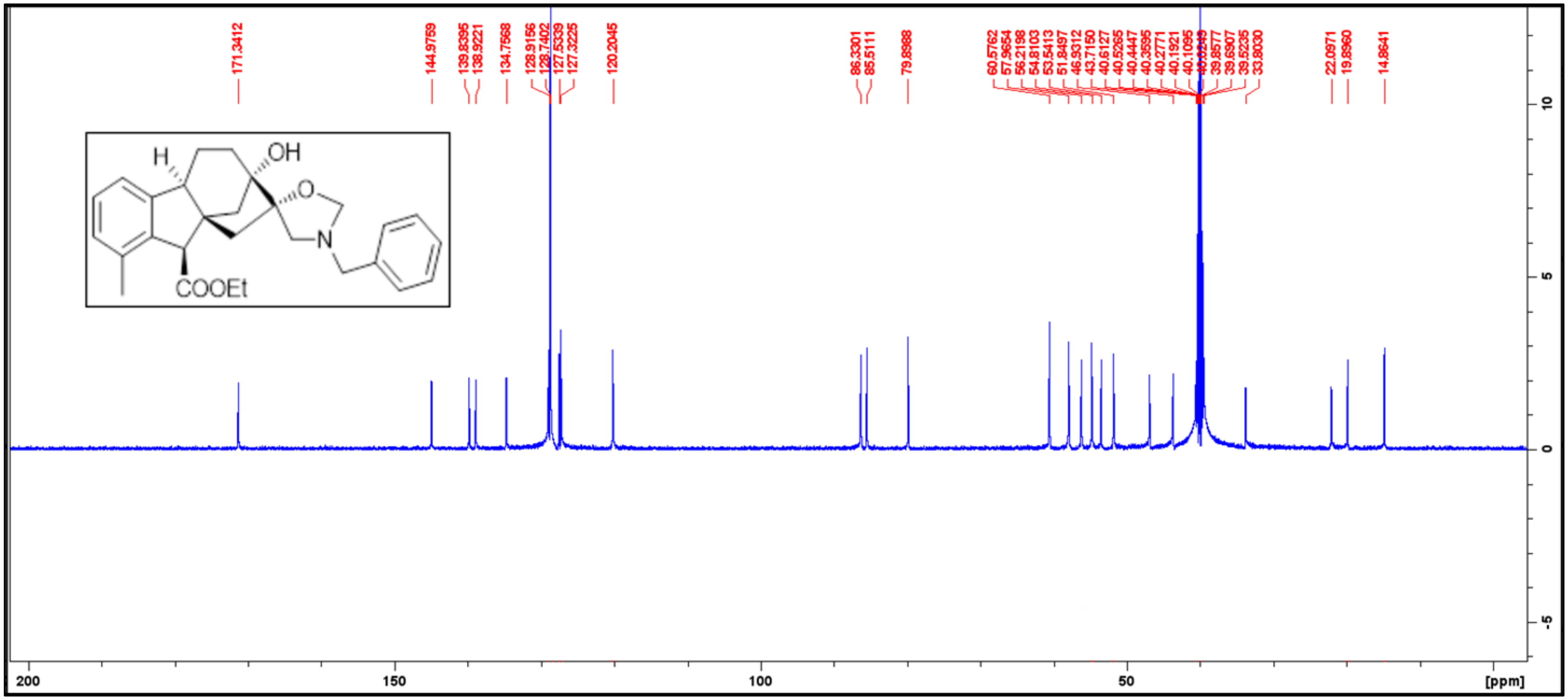

Figure S 56: ^{1}H-NMR of compound 25

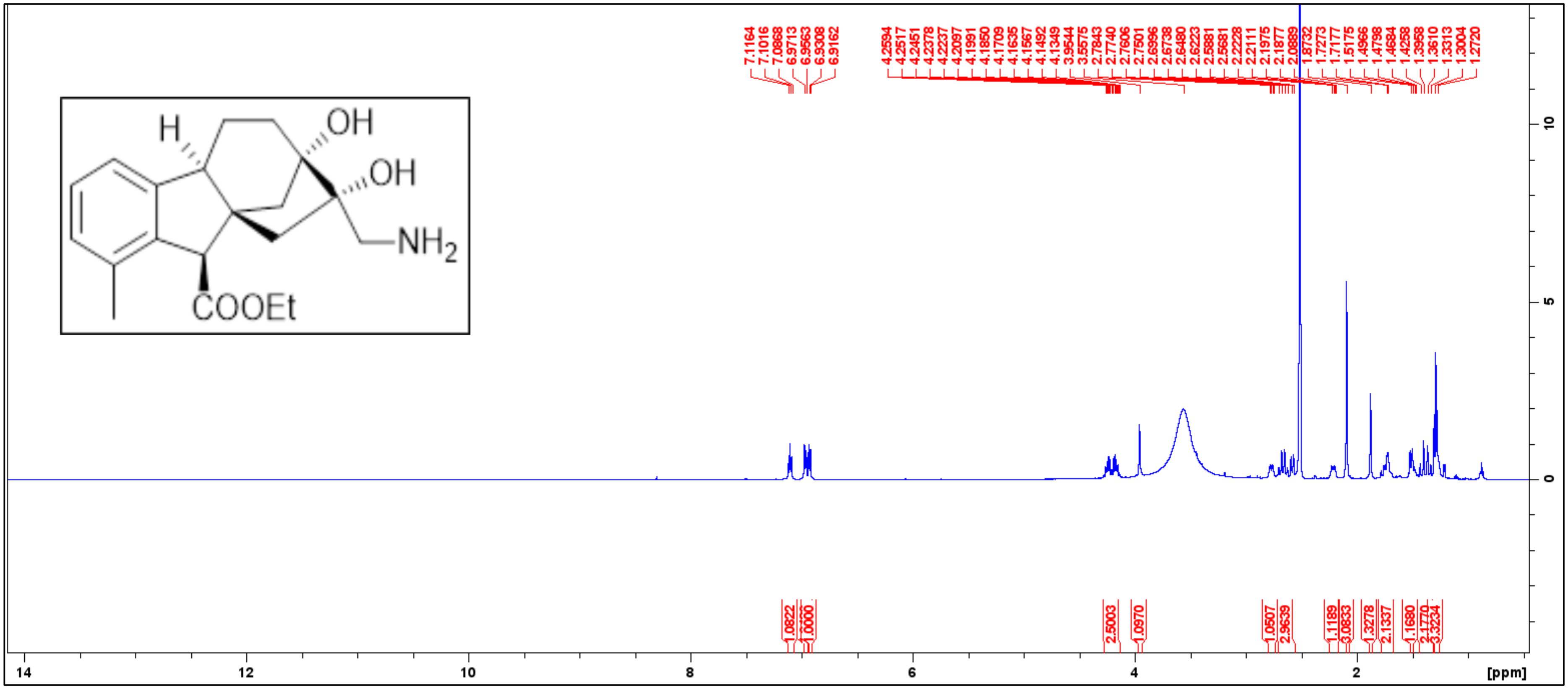

Figure S 57: ^{13}C-NMR (JMOD) of compound 25

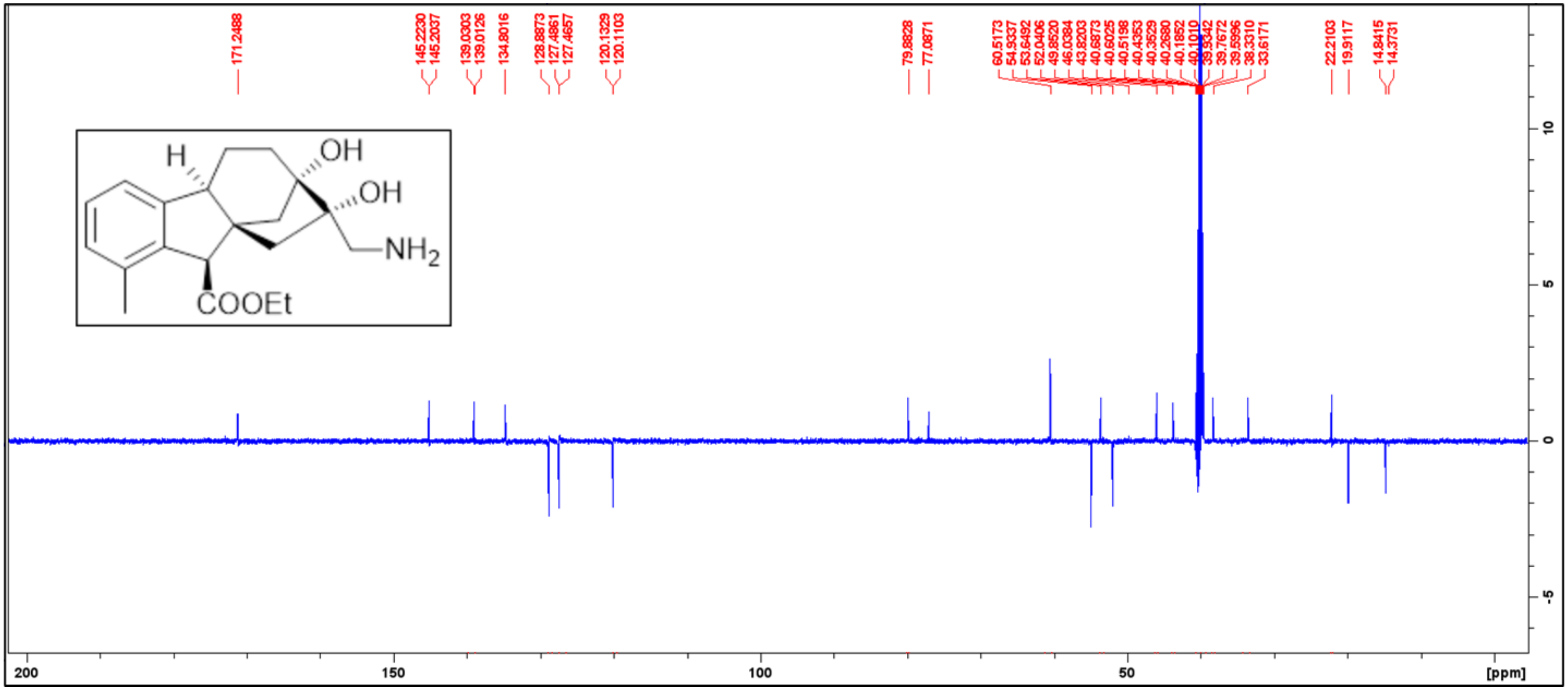

Figure S 58: ^{1}H-NMR of compound 26

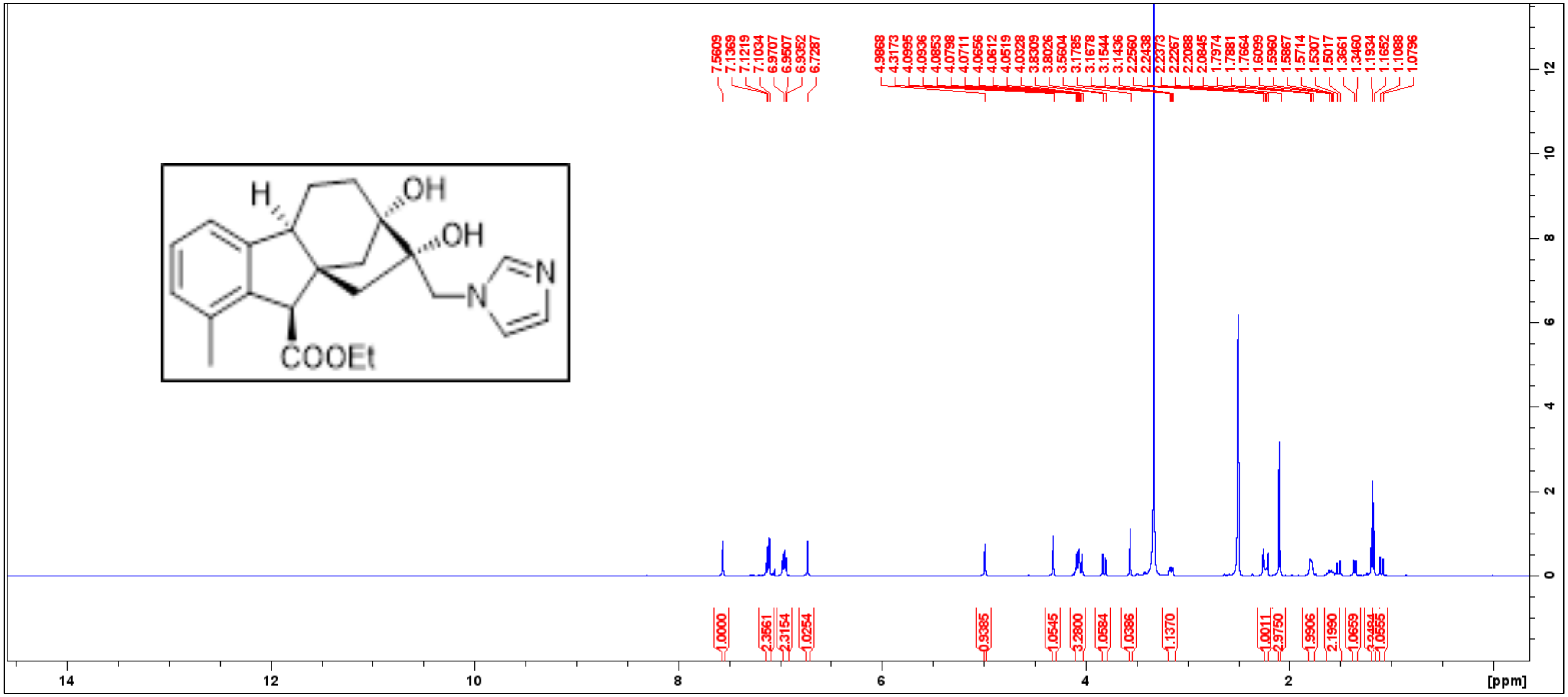

Figure S 59: ^{13}C-NMR (JMOD) of compound 26

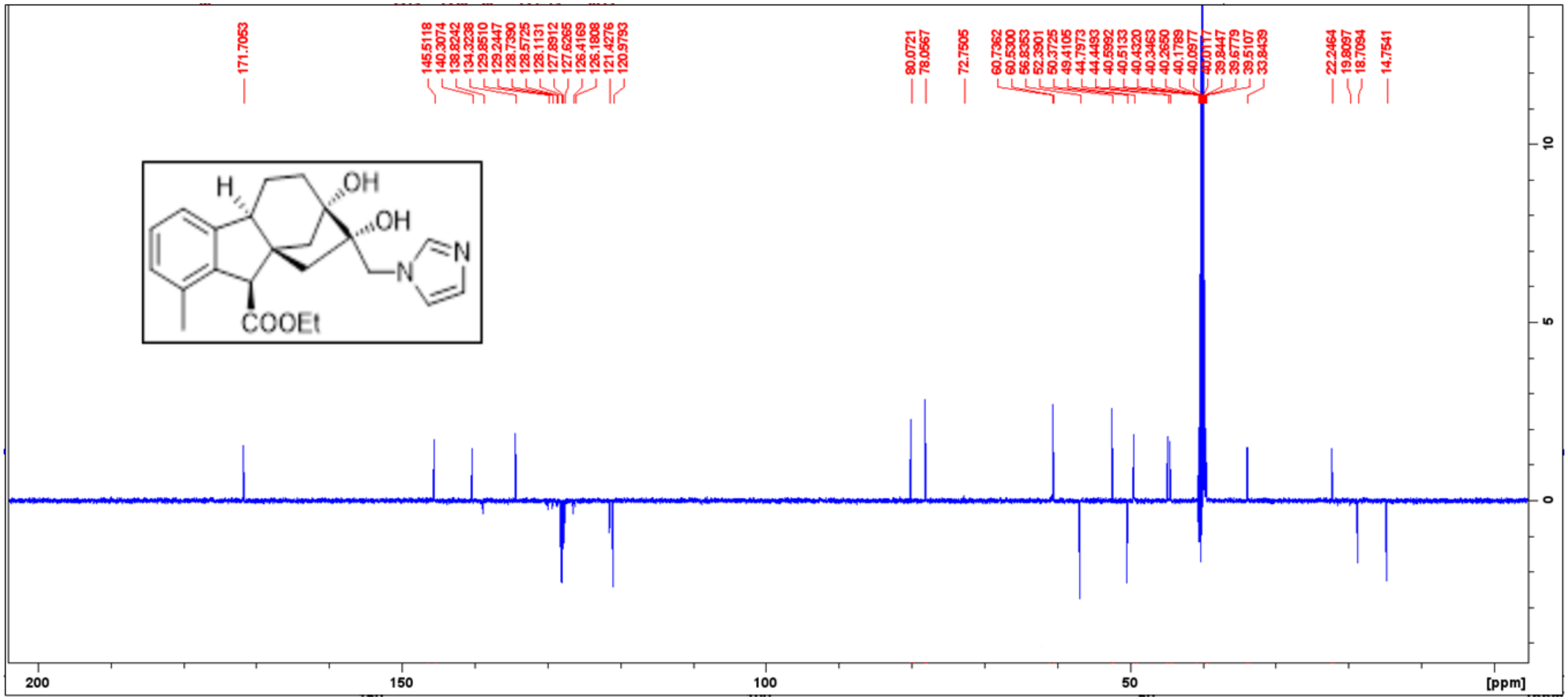

Figure S 60: ^{1}H-NMR of compound 27

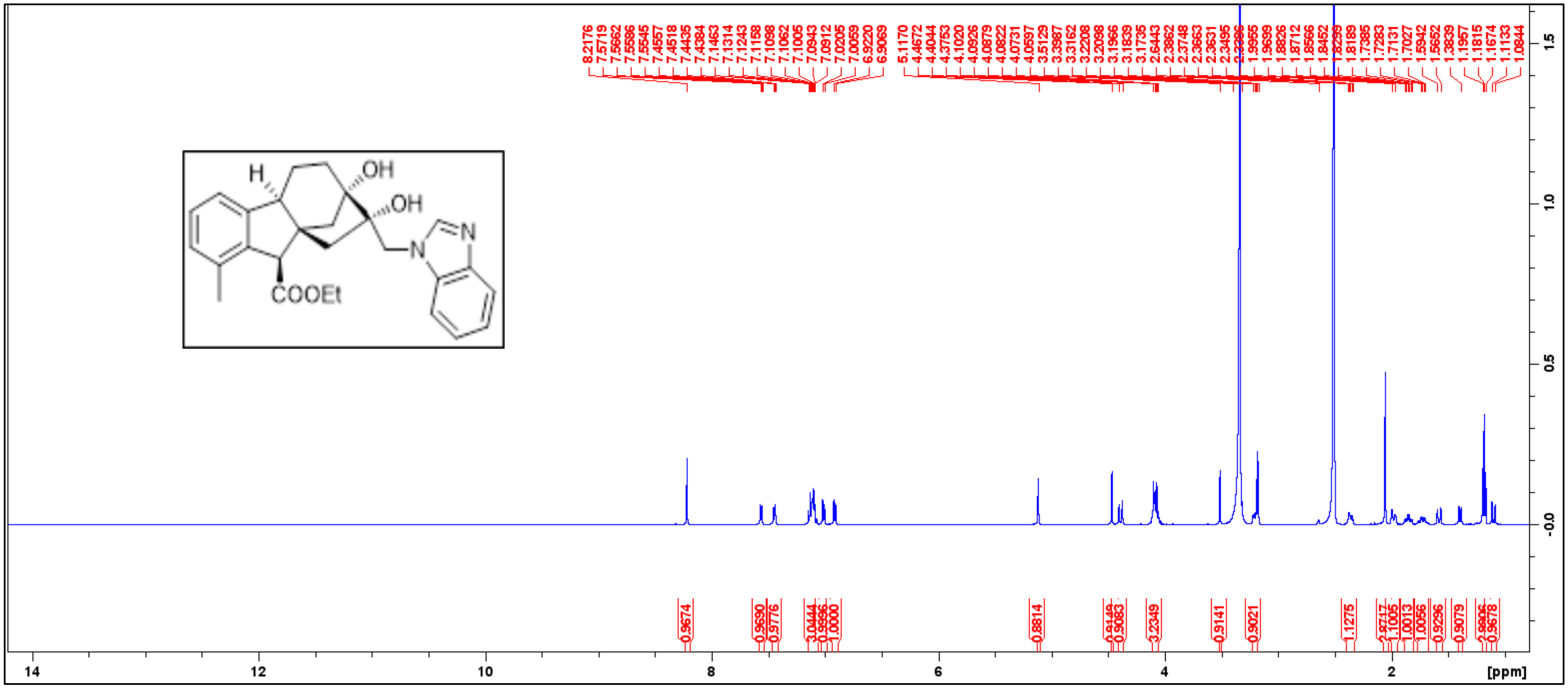

Figure S 61: ^{13}C-NMR (JMOD) of compound 27

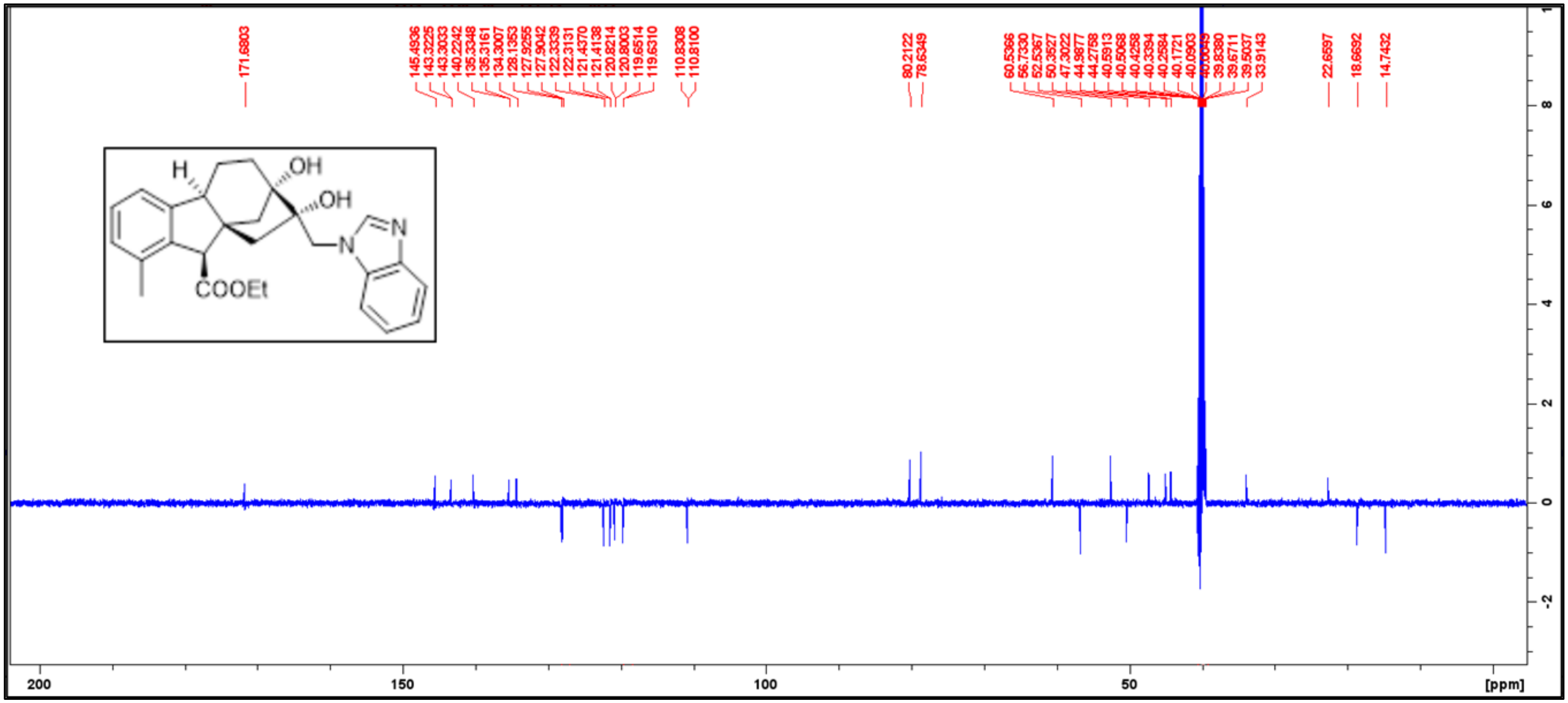

Figure S 62: ^{1}H-NMR of compound 28

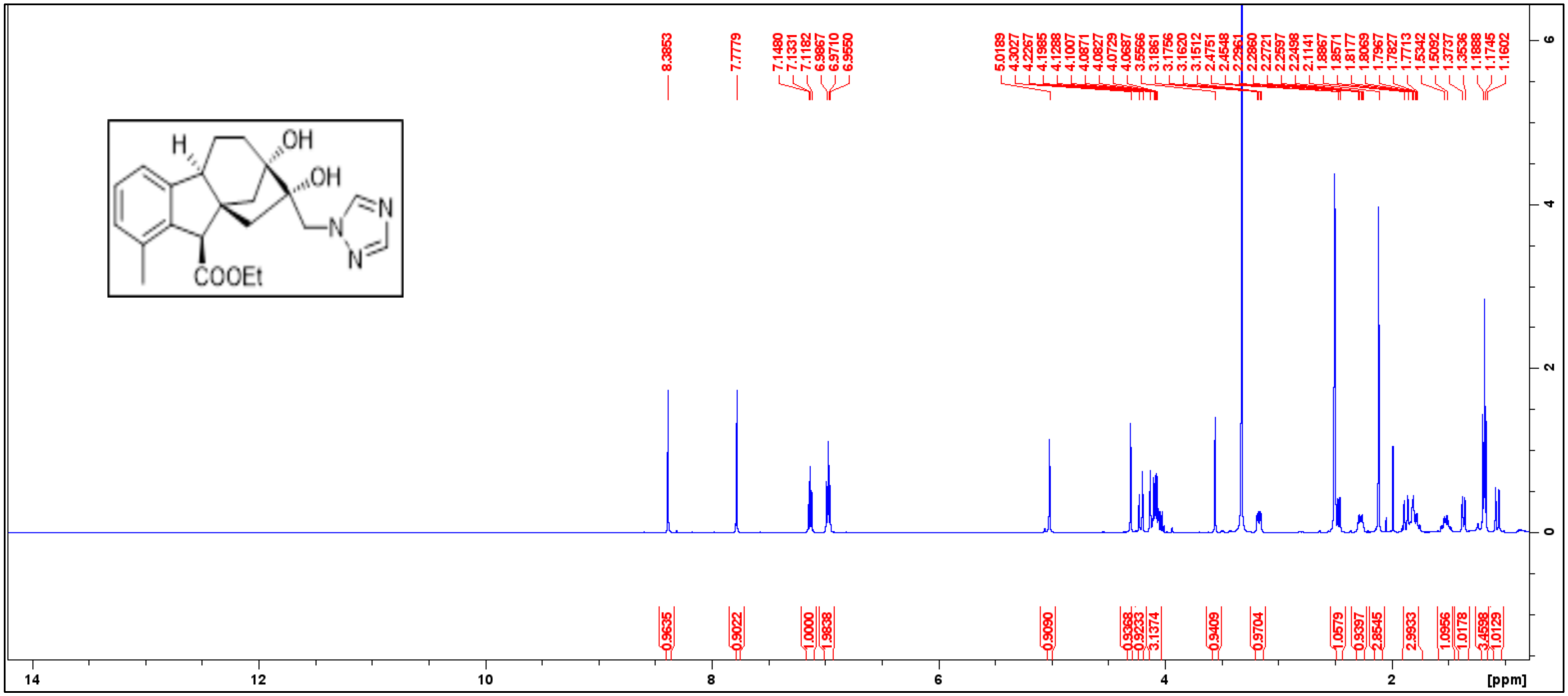

Figure S 63: ^{13}C-NMR (JMOD) of compound 28

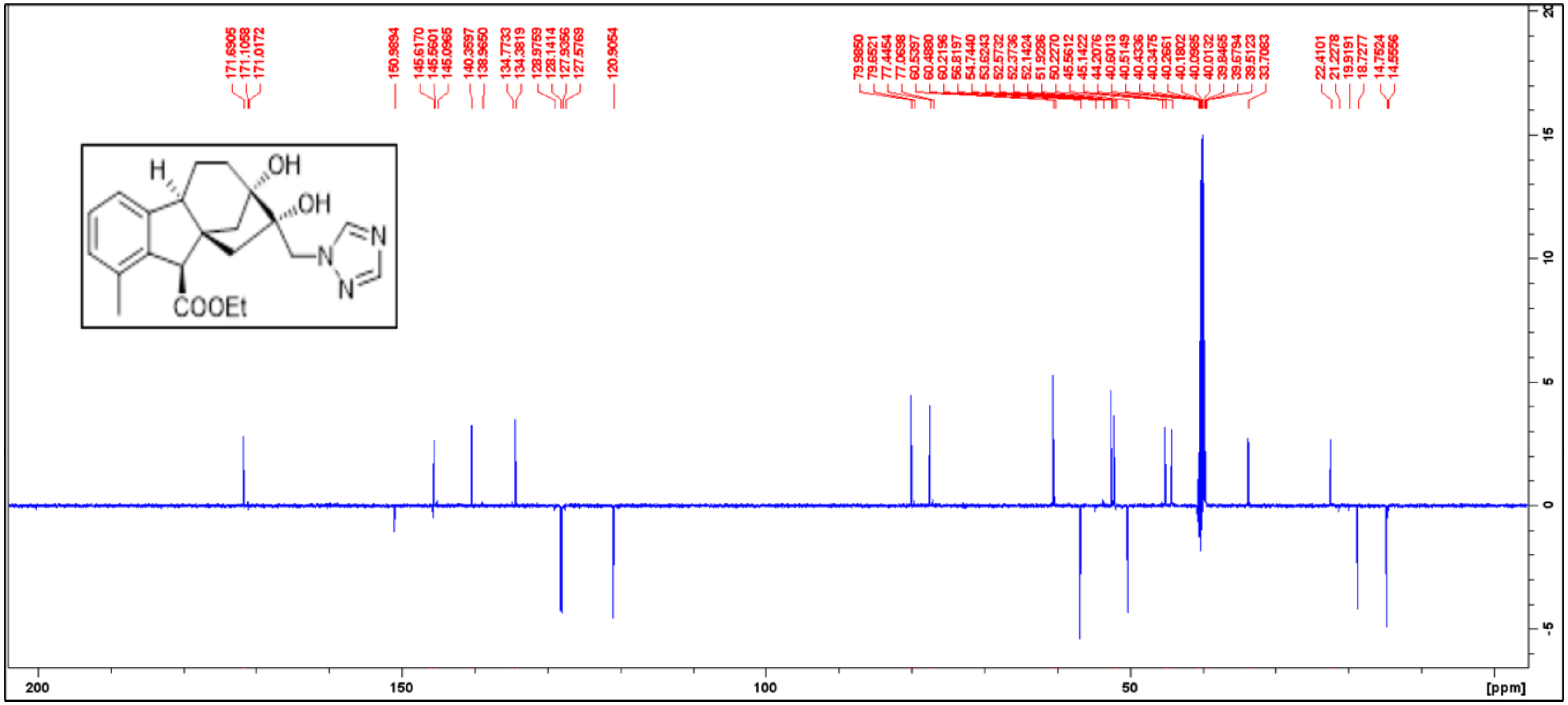

Figure S 64: ^{1}H-NMR of compound 29

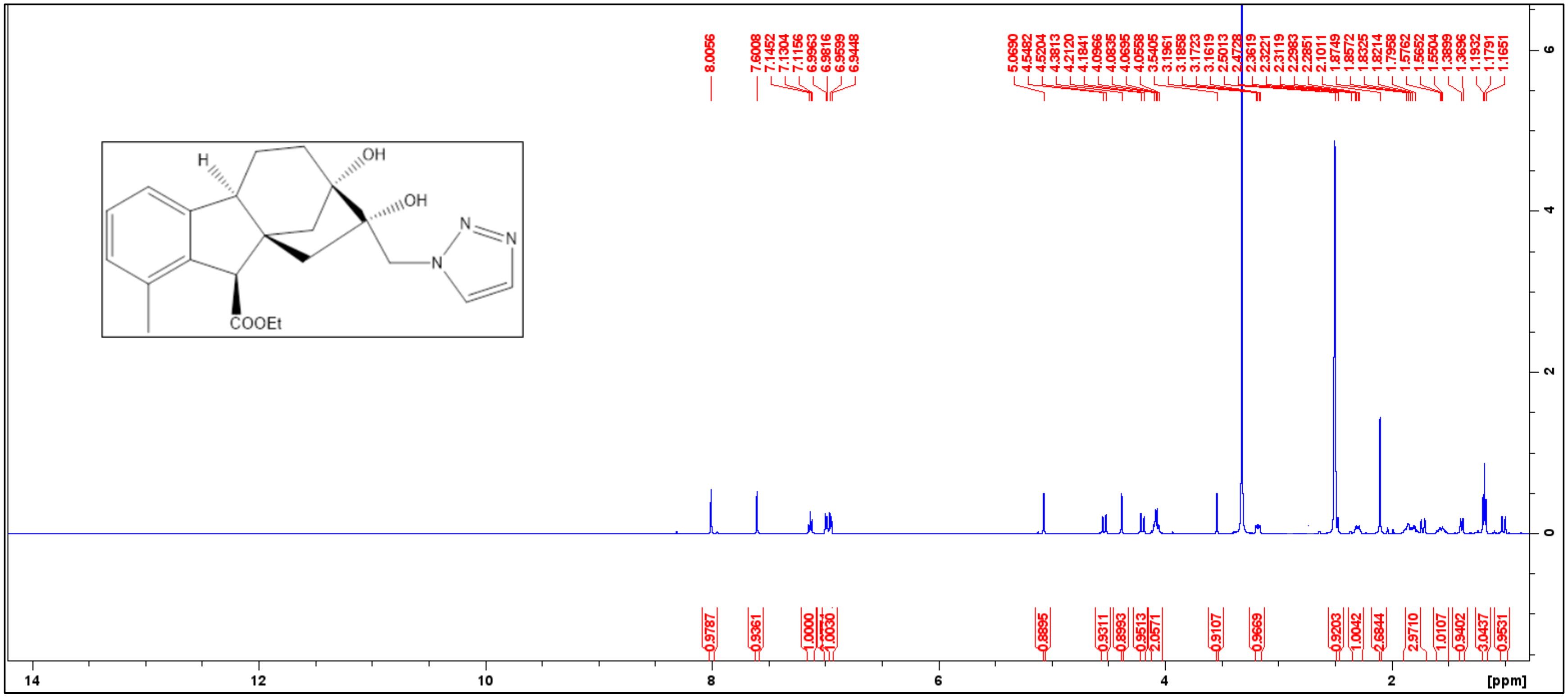

Figure S 65: ^{13}C-NMR (JMOD) of compound 29

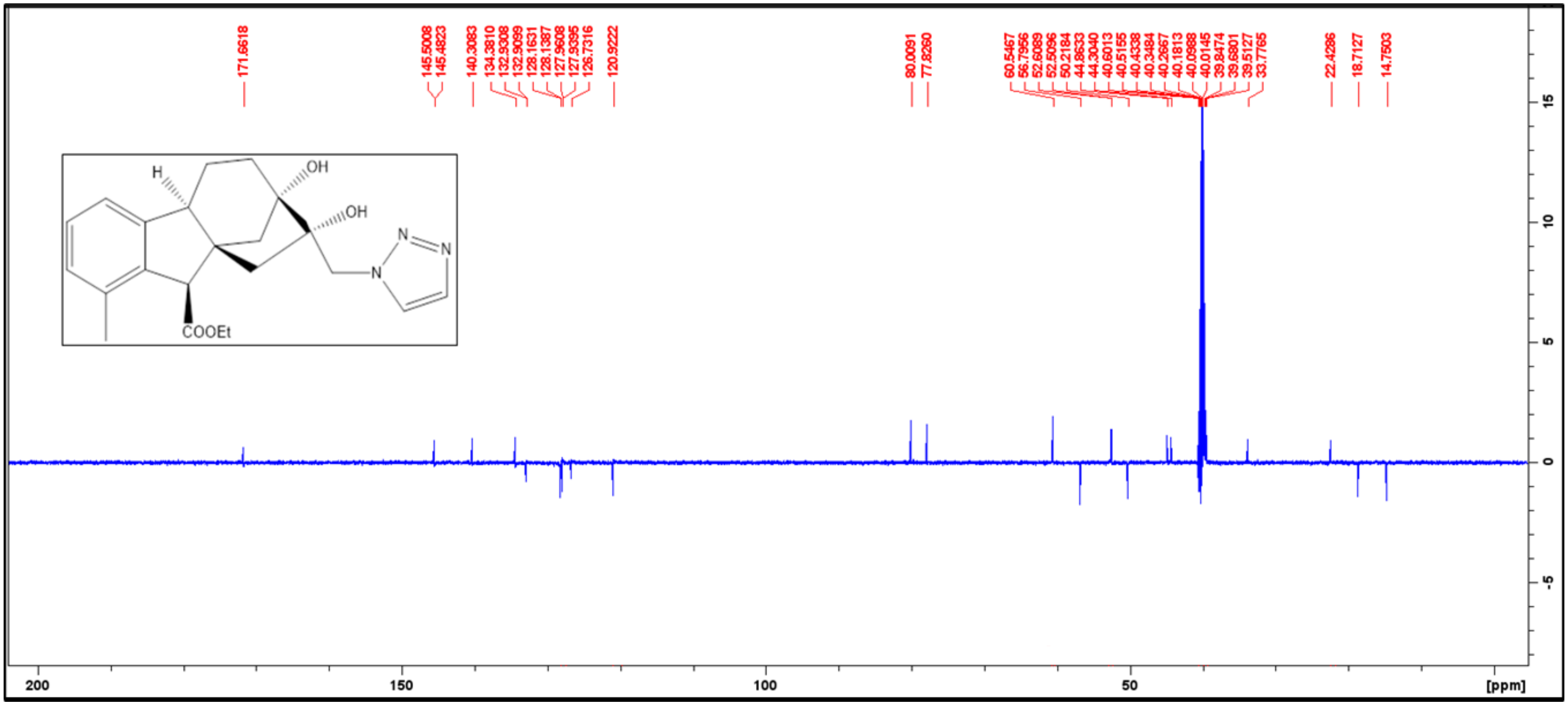

Figure S 66: ^{1}H-NMR of compound 30

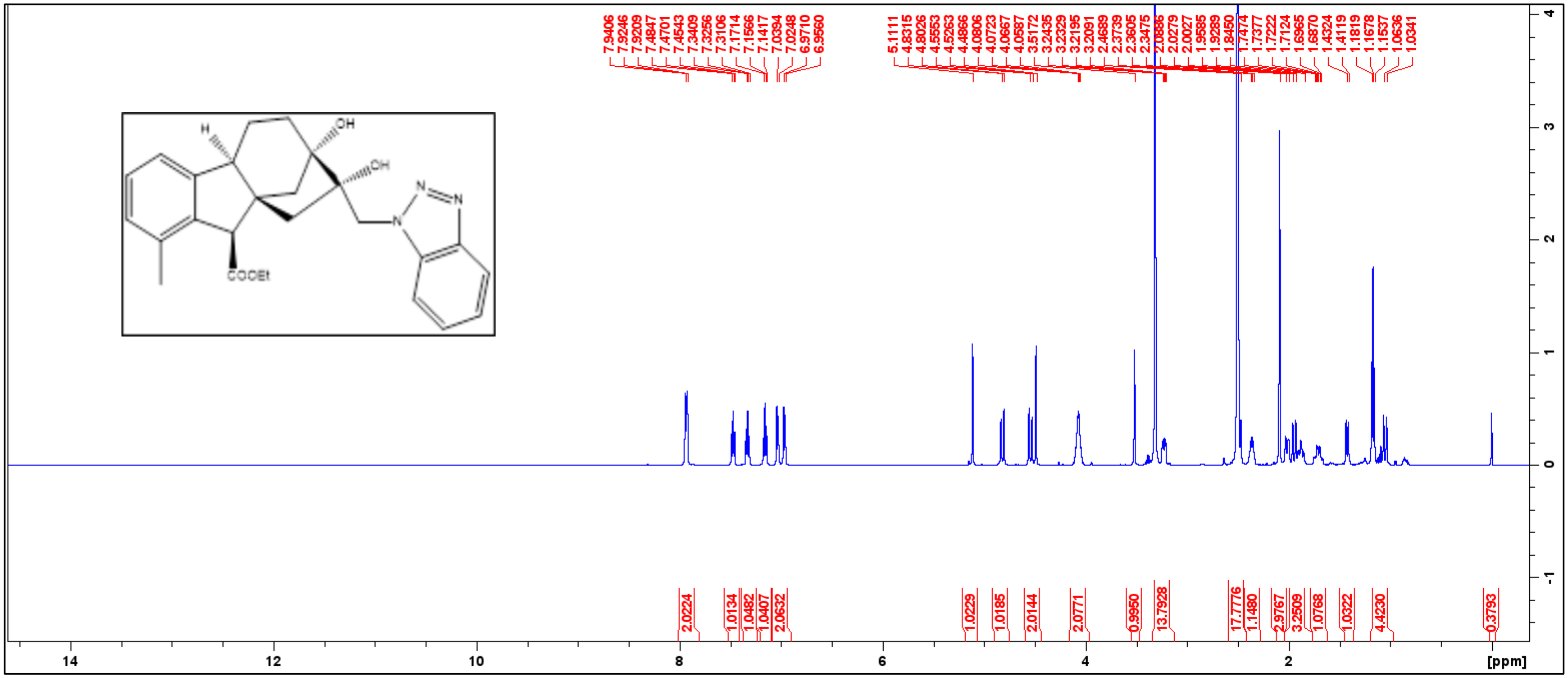

Figure S 67: ^{13}C-NMR (JMOD) of compound 30

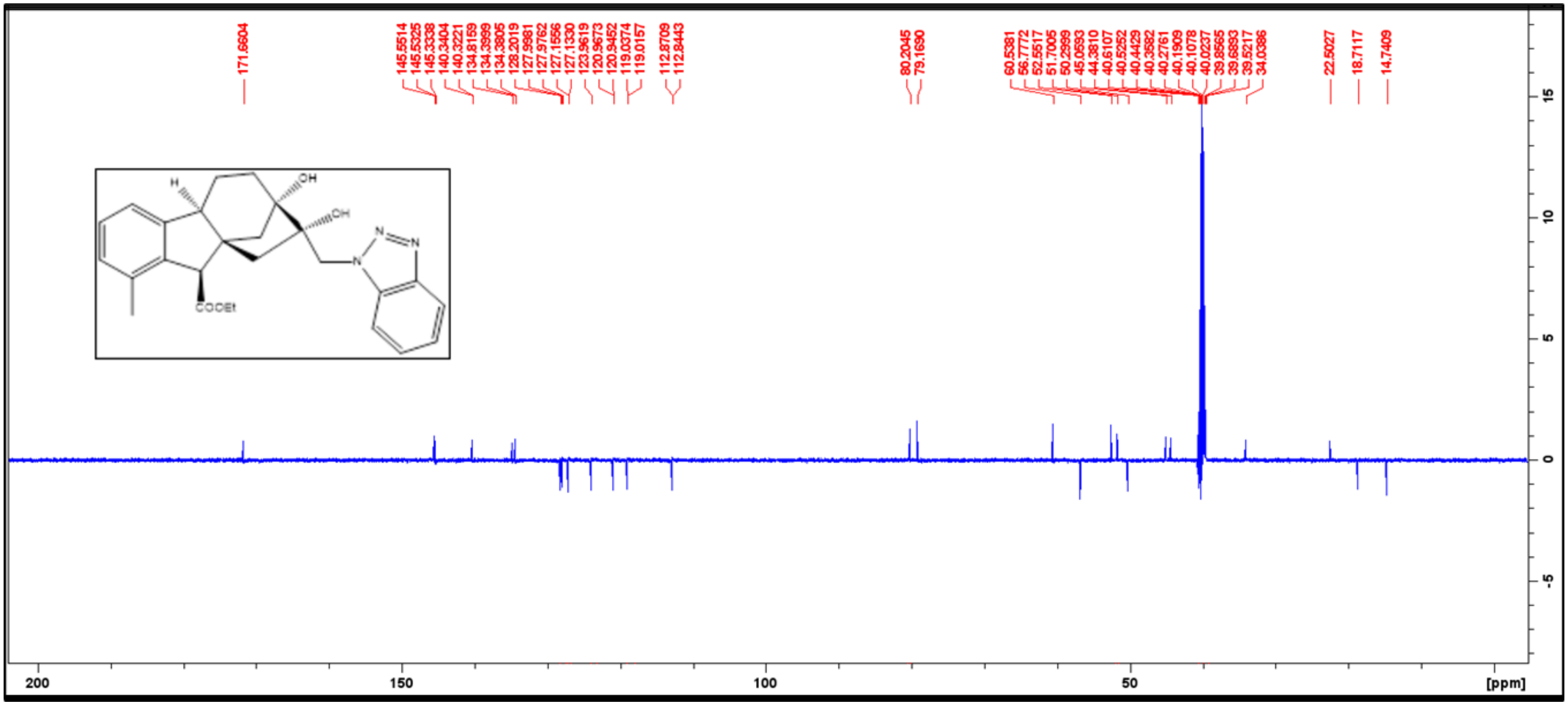

Figure S 68: ^{1}H-NMR of compound 31

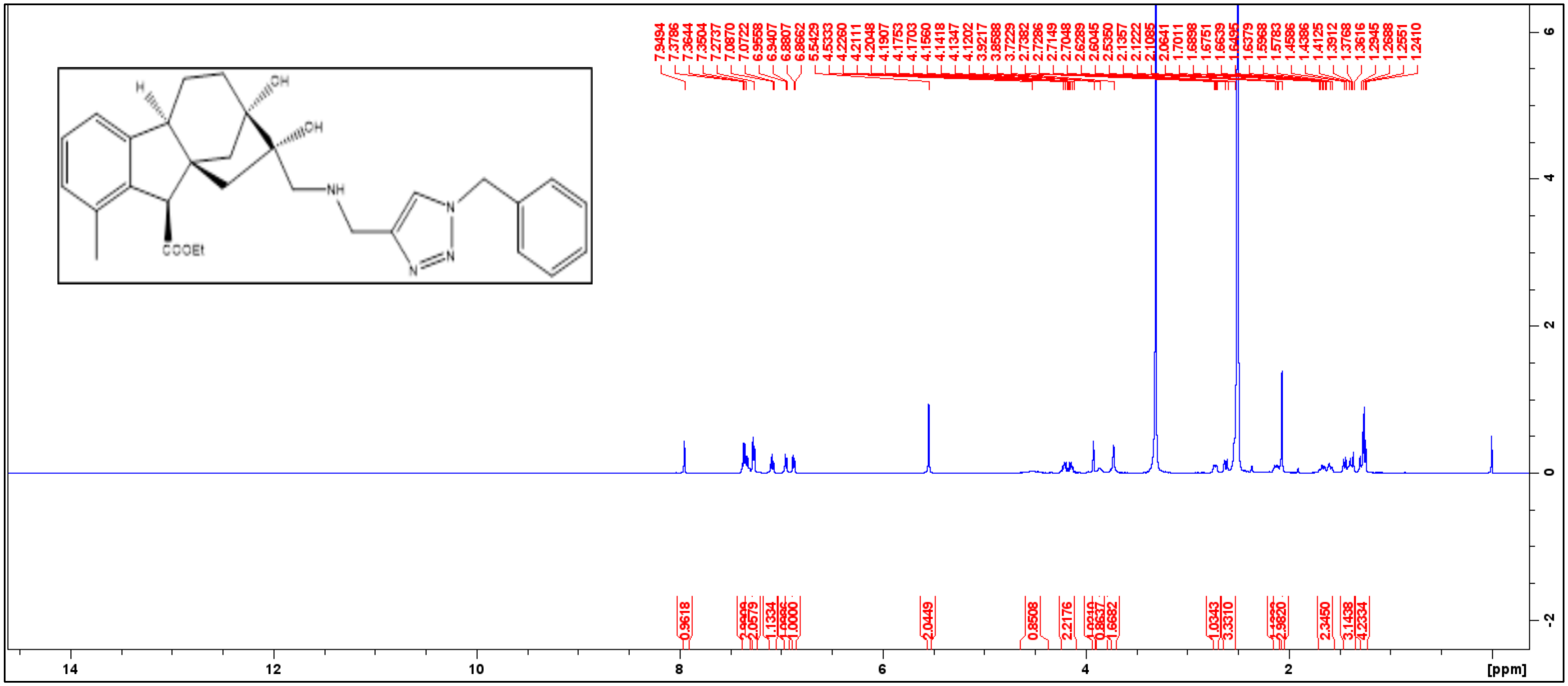

Figure S 69: ^{13}C-NMR (JMOD) of compound 31

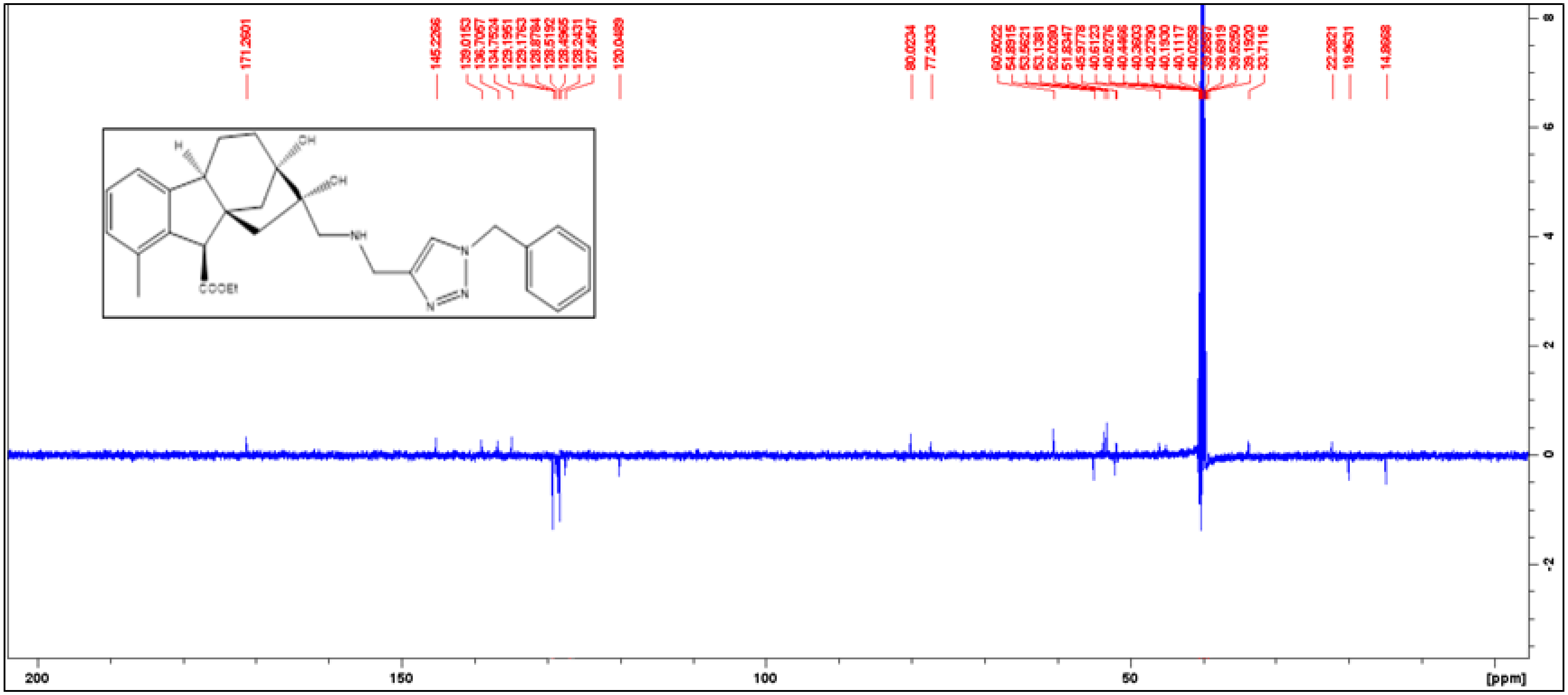

Figure S 70: HSQC of compound 31

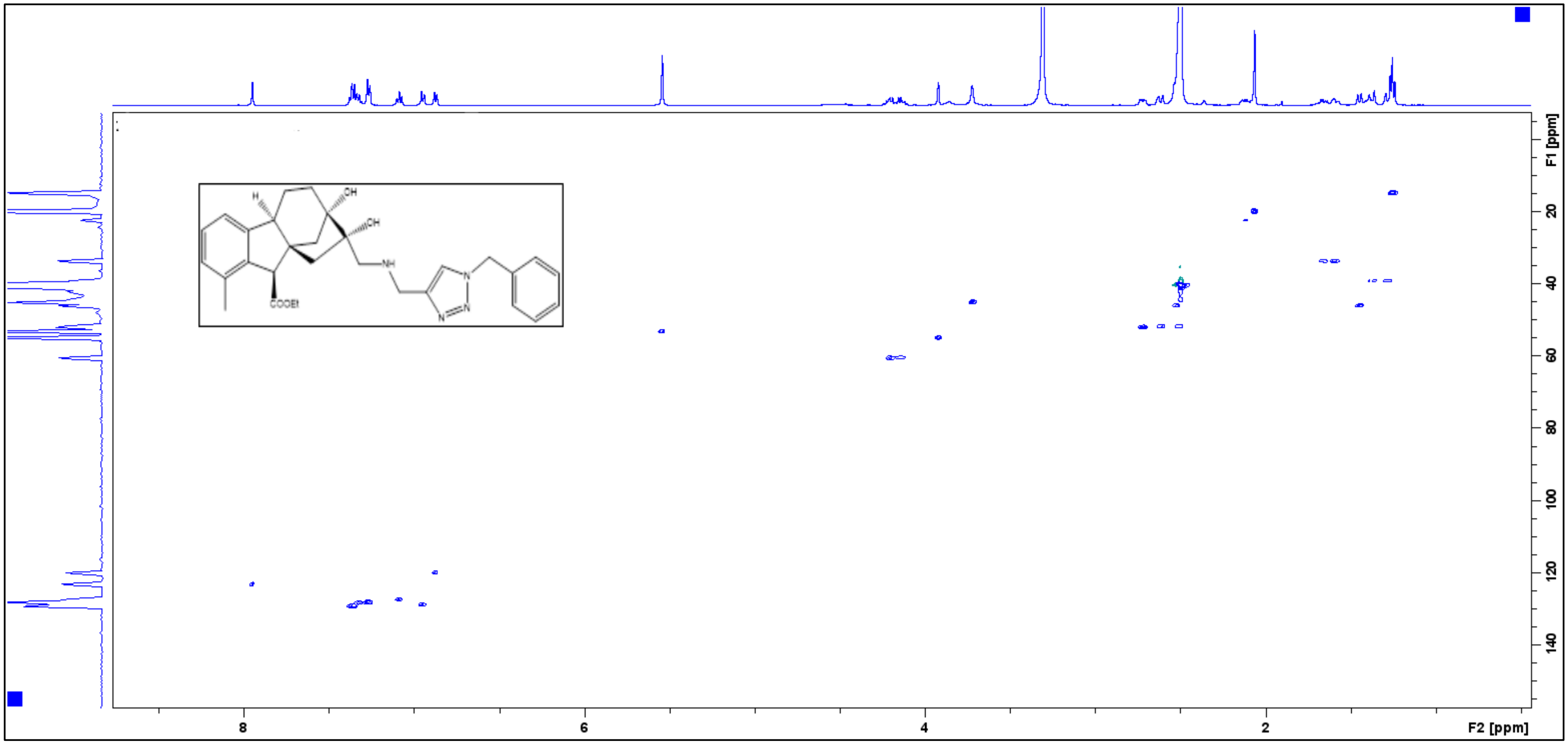

Figure S 71: HMBC of compound 31

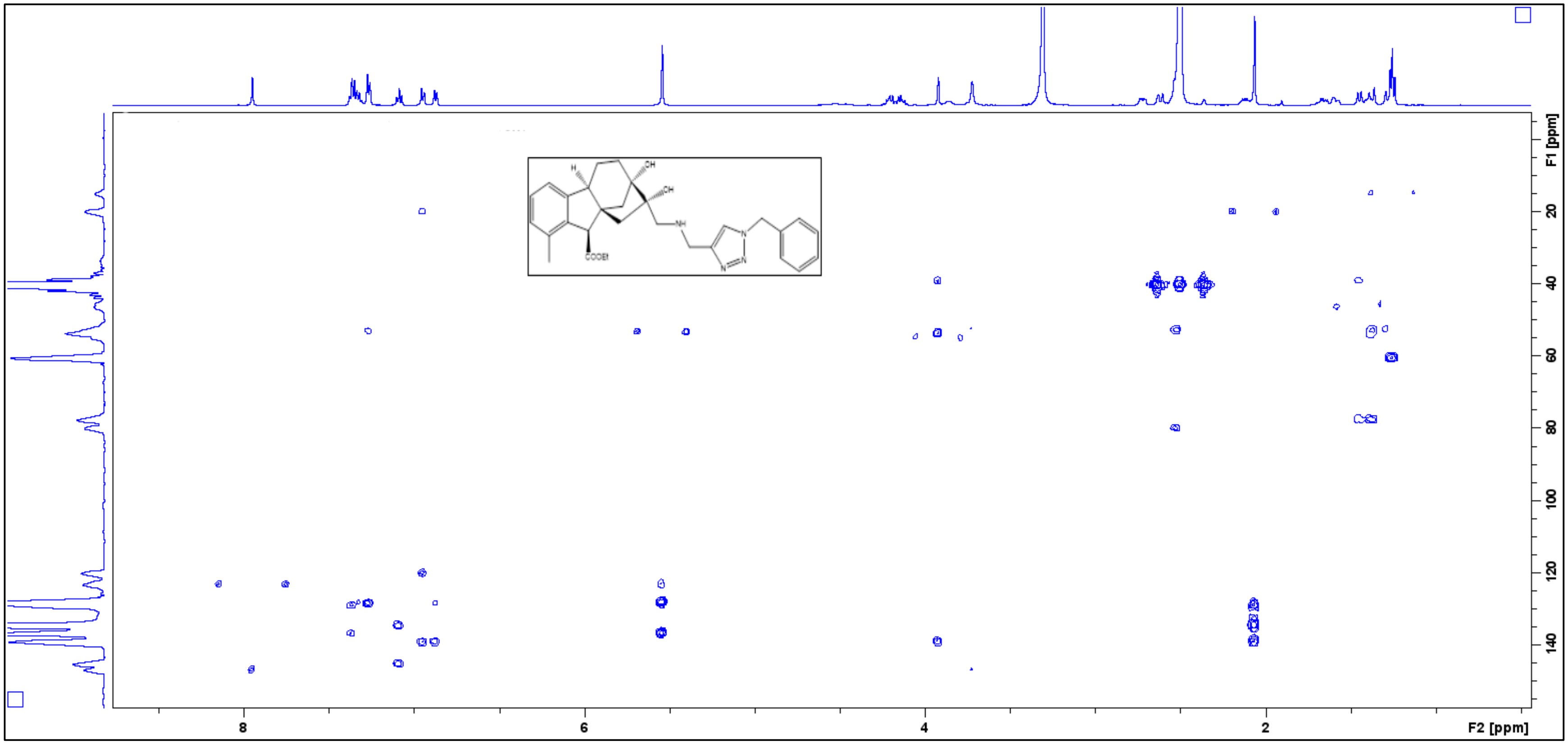

Figure S 72: ^{1}H-NMR of Azido diol derivative

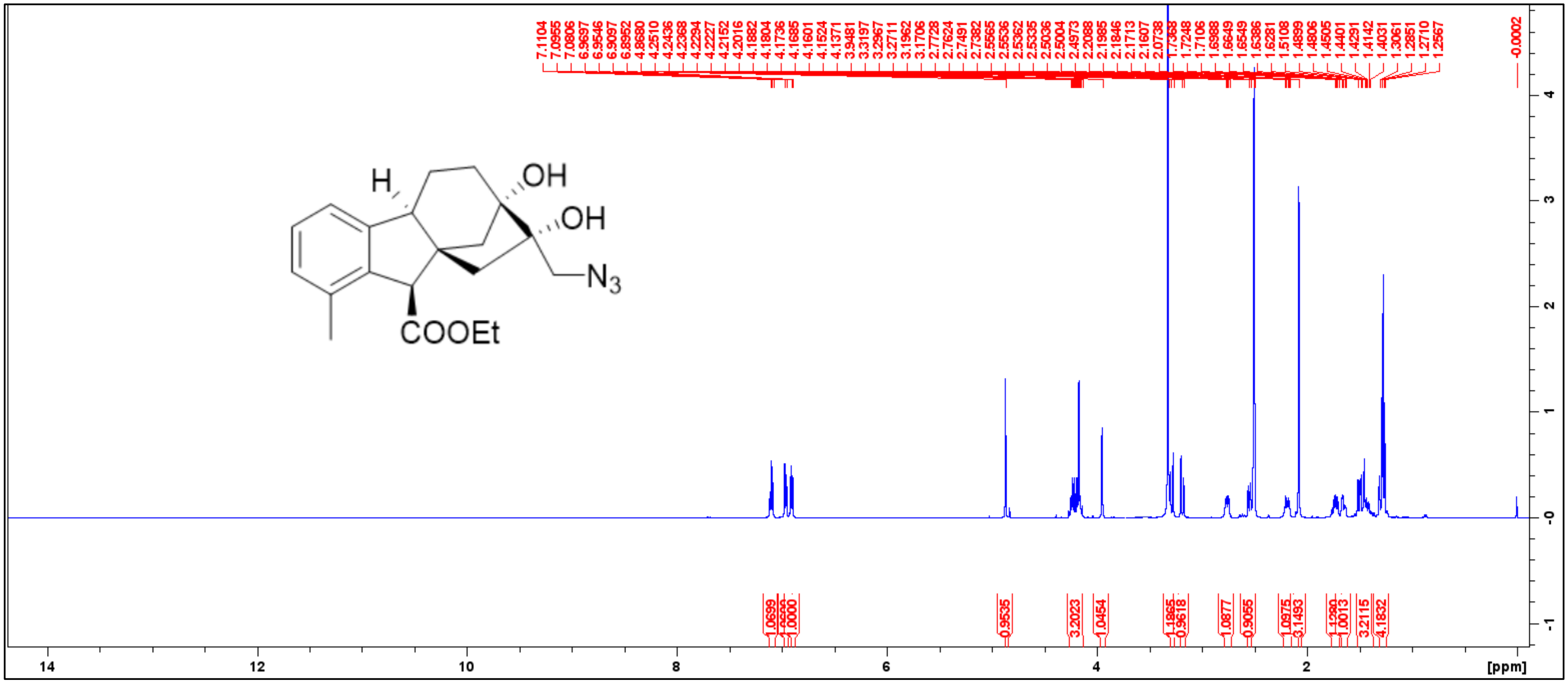

Figure S 73: ^{13}C-NMR (JMOD) of Azido diol derivative

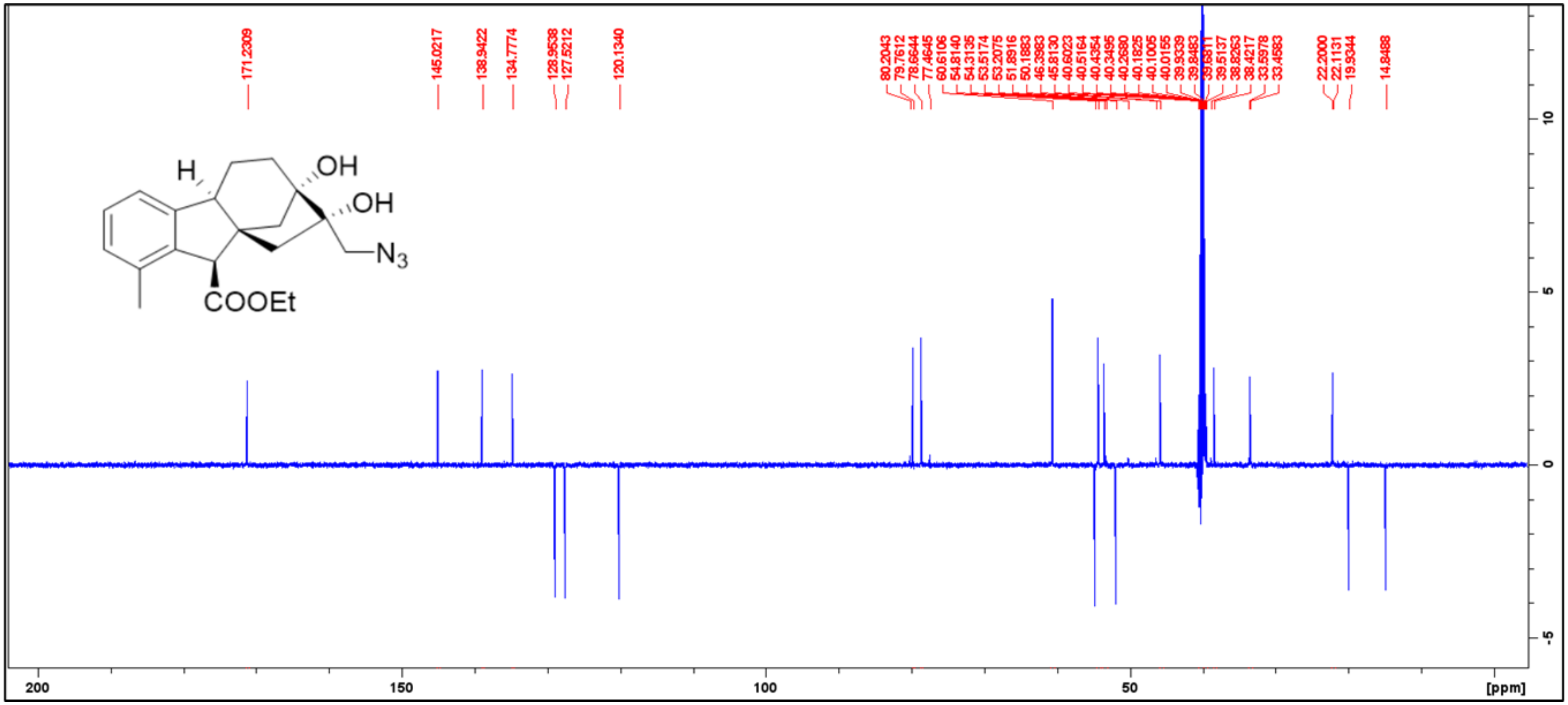

Figure S 74: ^{1}H-NMR of compound 32

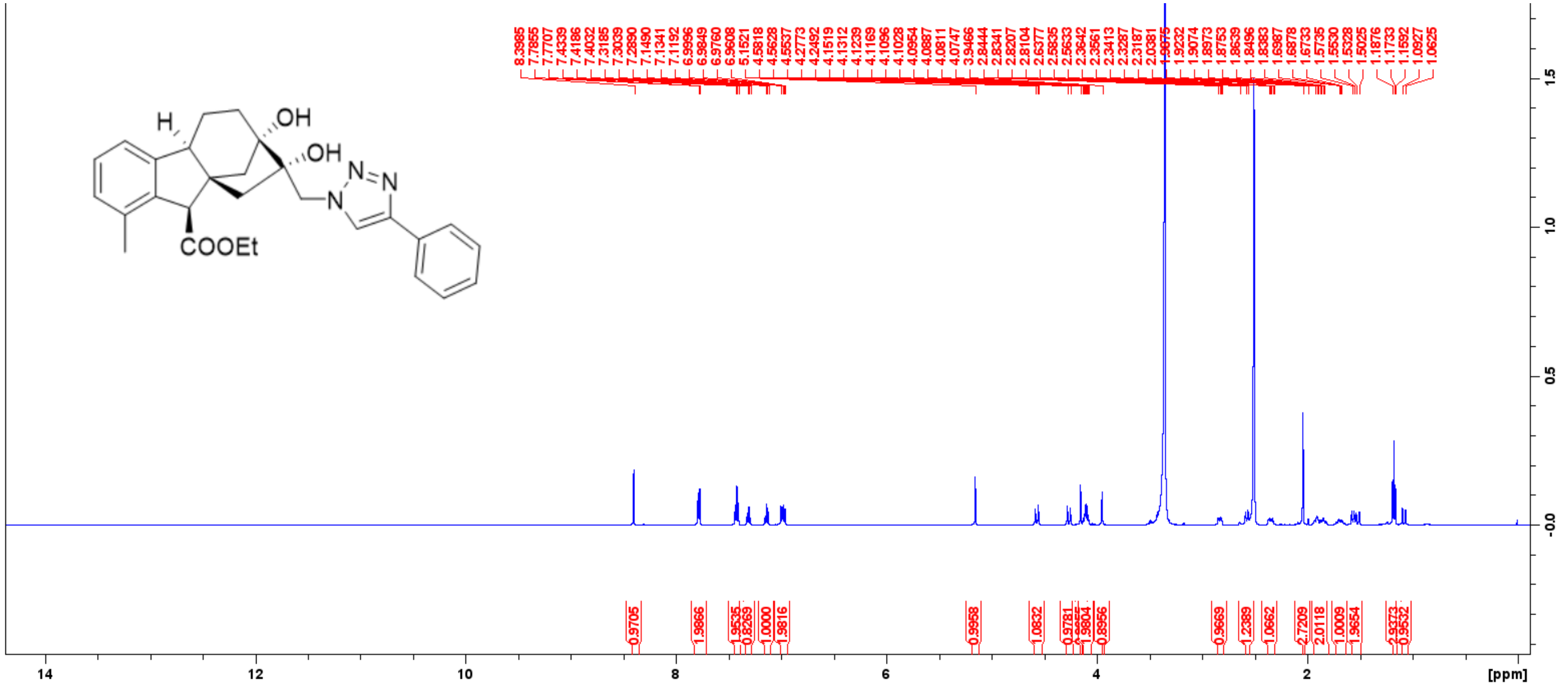

Figure S 75: ^{13}C-NMR (JMOD) of compound 32

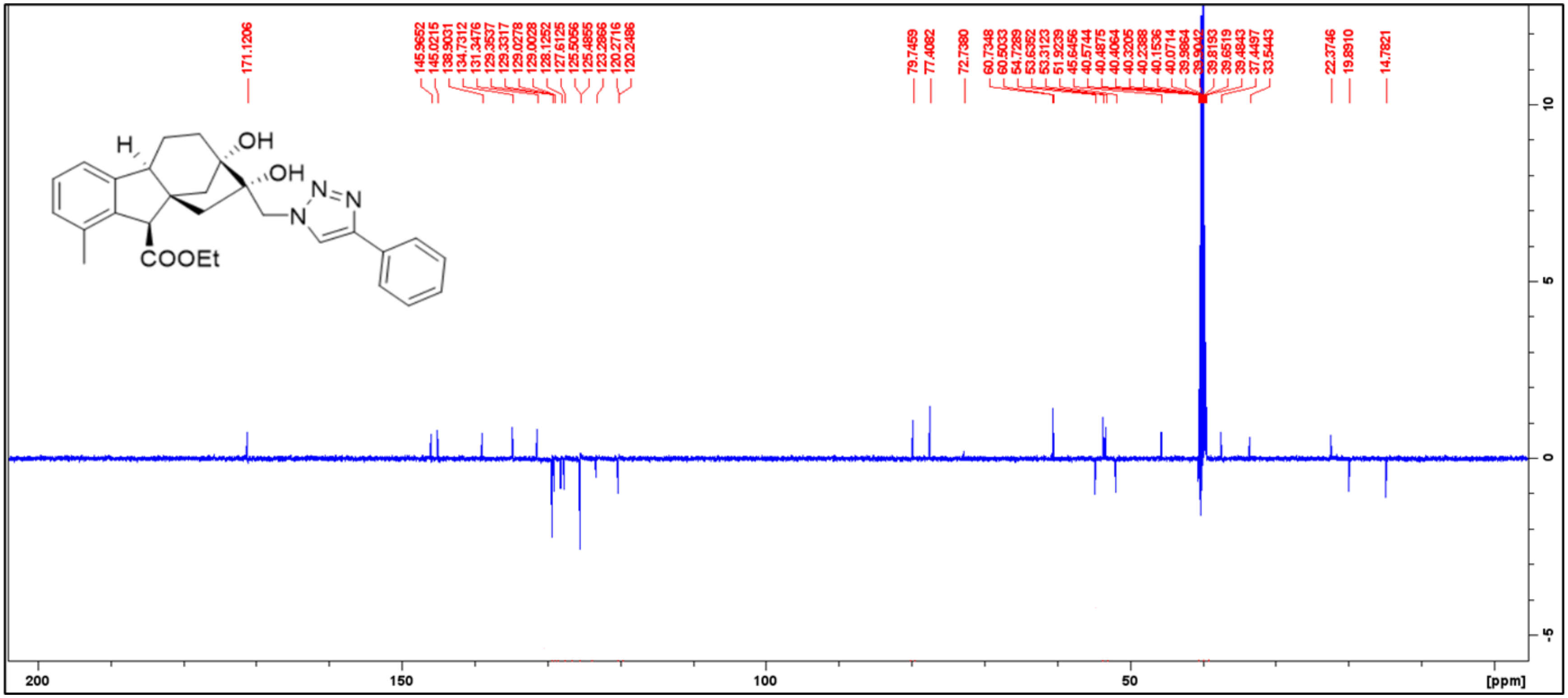

Figure S 76: ^{1}H-NMR of compound 33

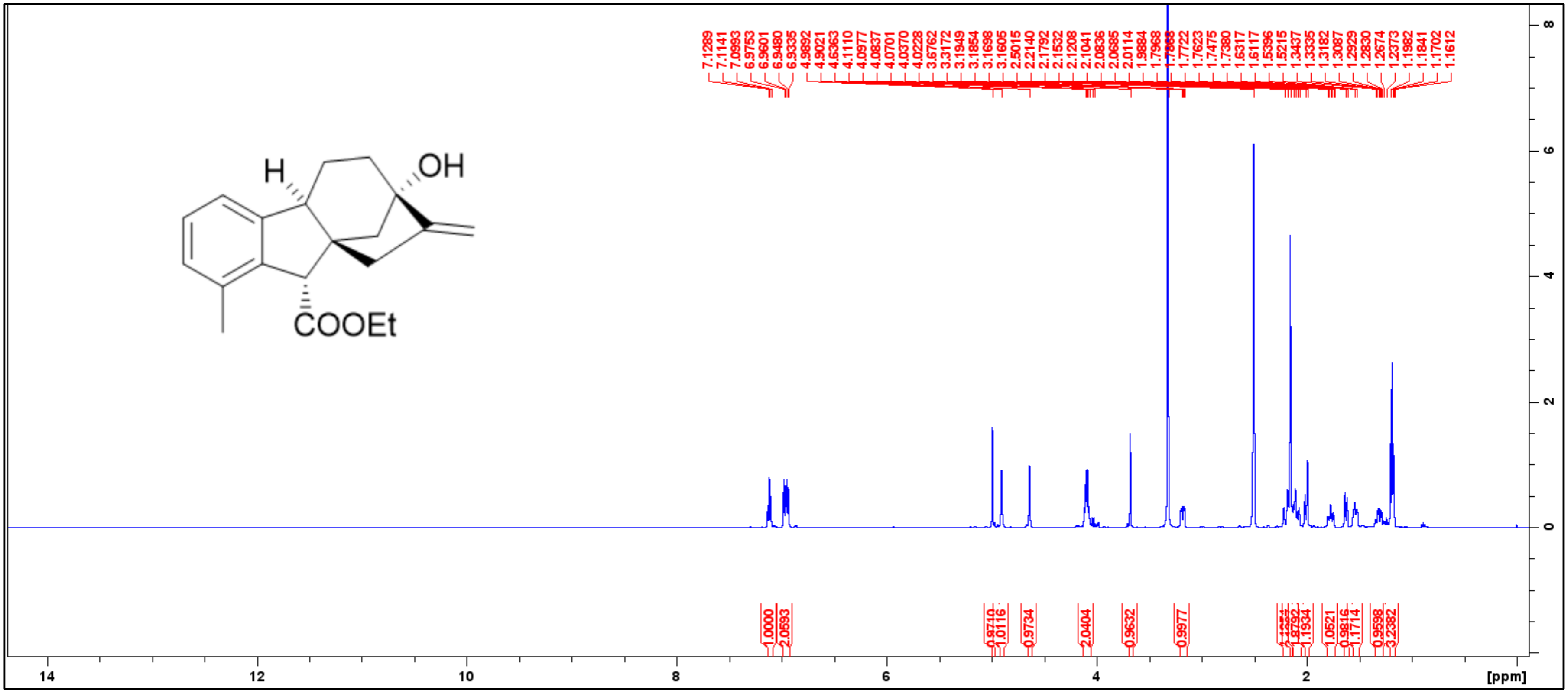

Figure S 77: ^{13}C-NMR (JMOD) of compound 33

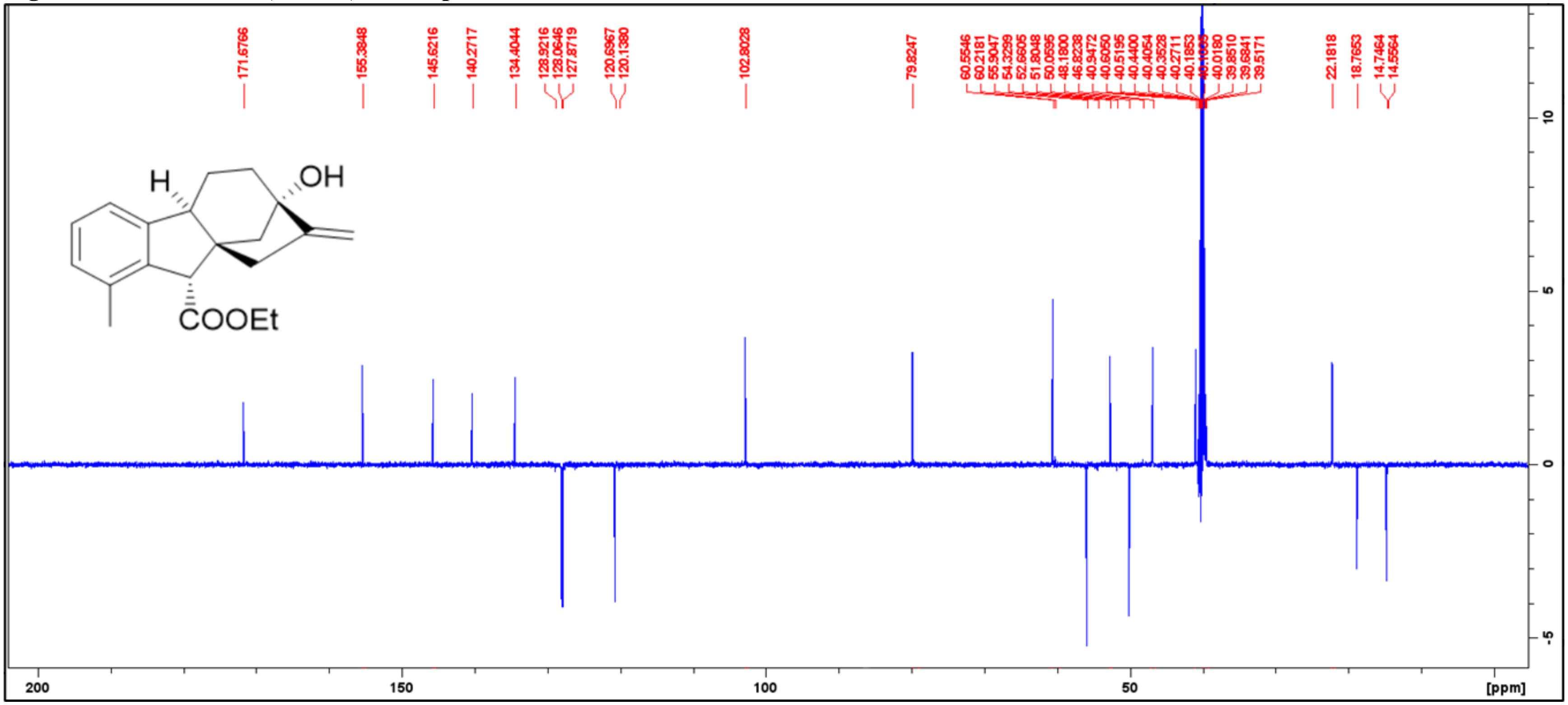

Figure S 78: COSY of compound 33

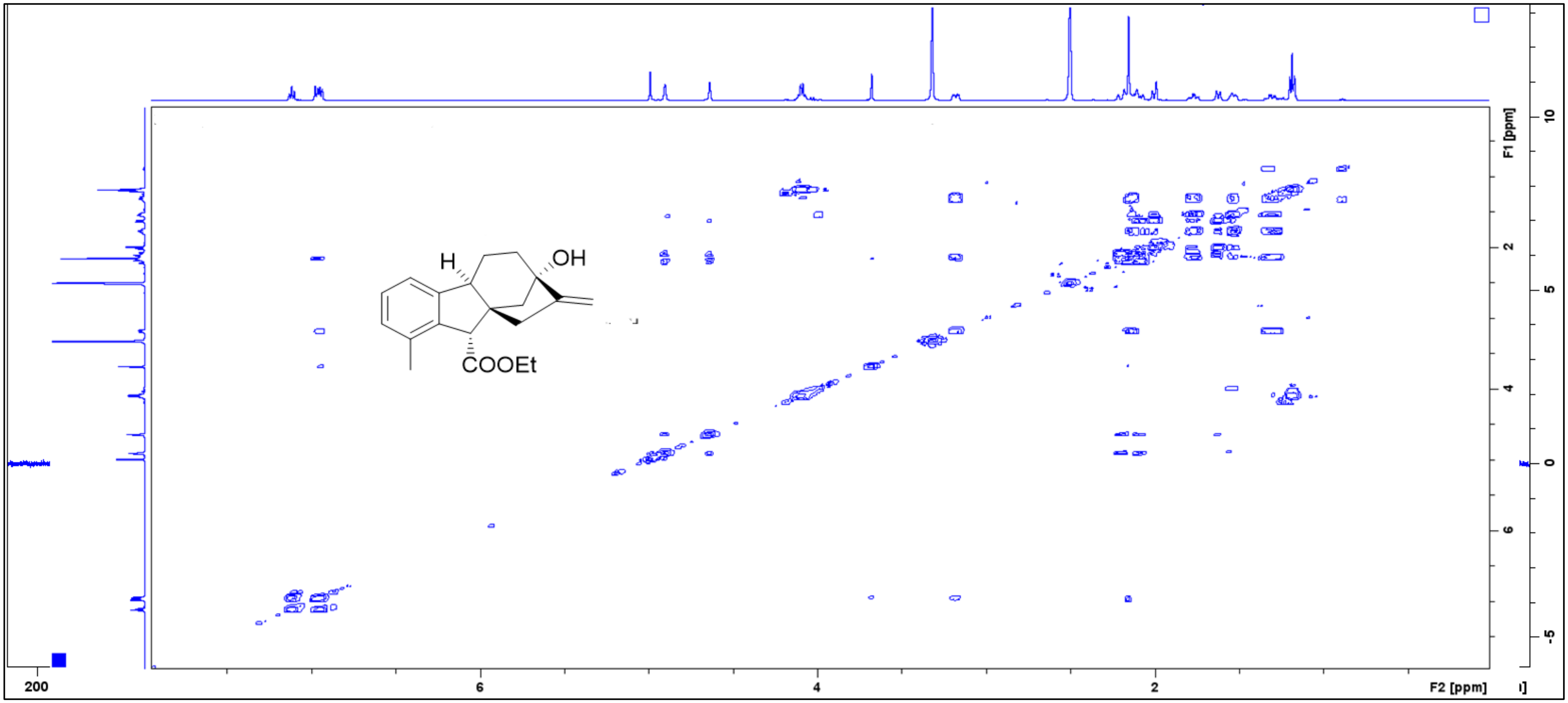

Figure S 79: NOESY of compound 33

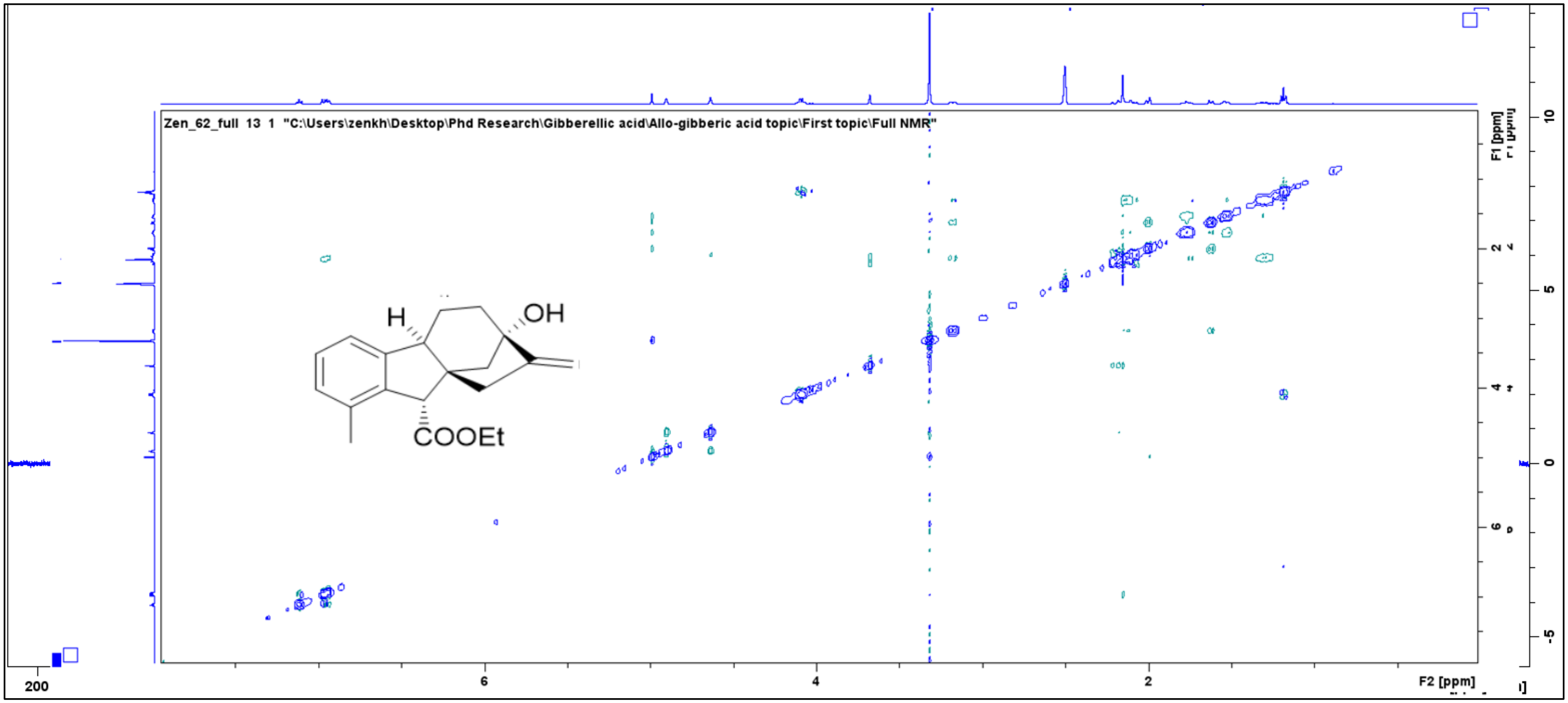

Figure S 80: HSQC of compound 33

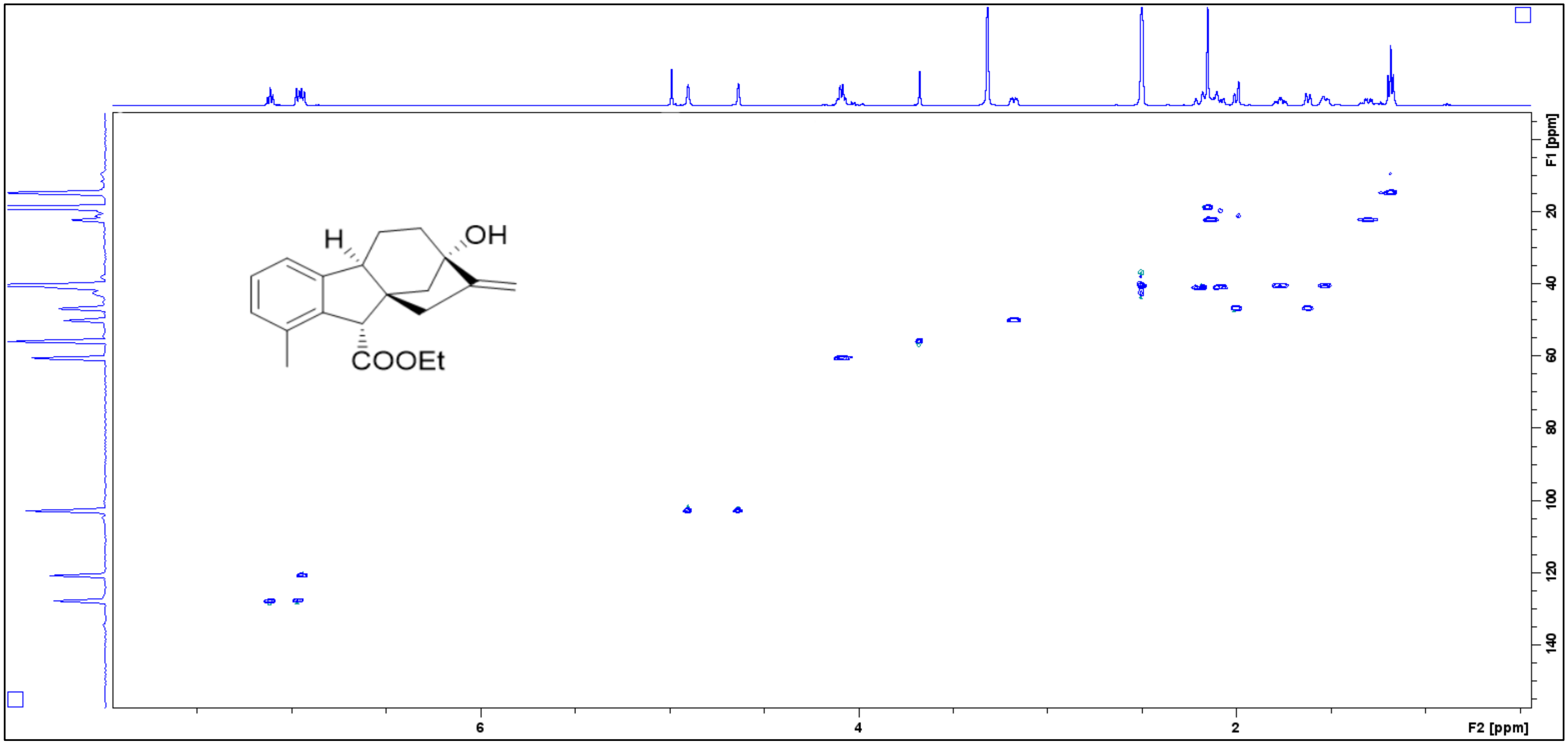

Figure S 81: HMBC of compound 33

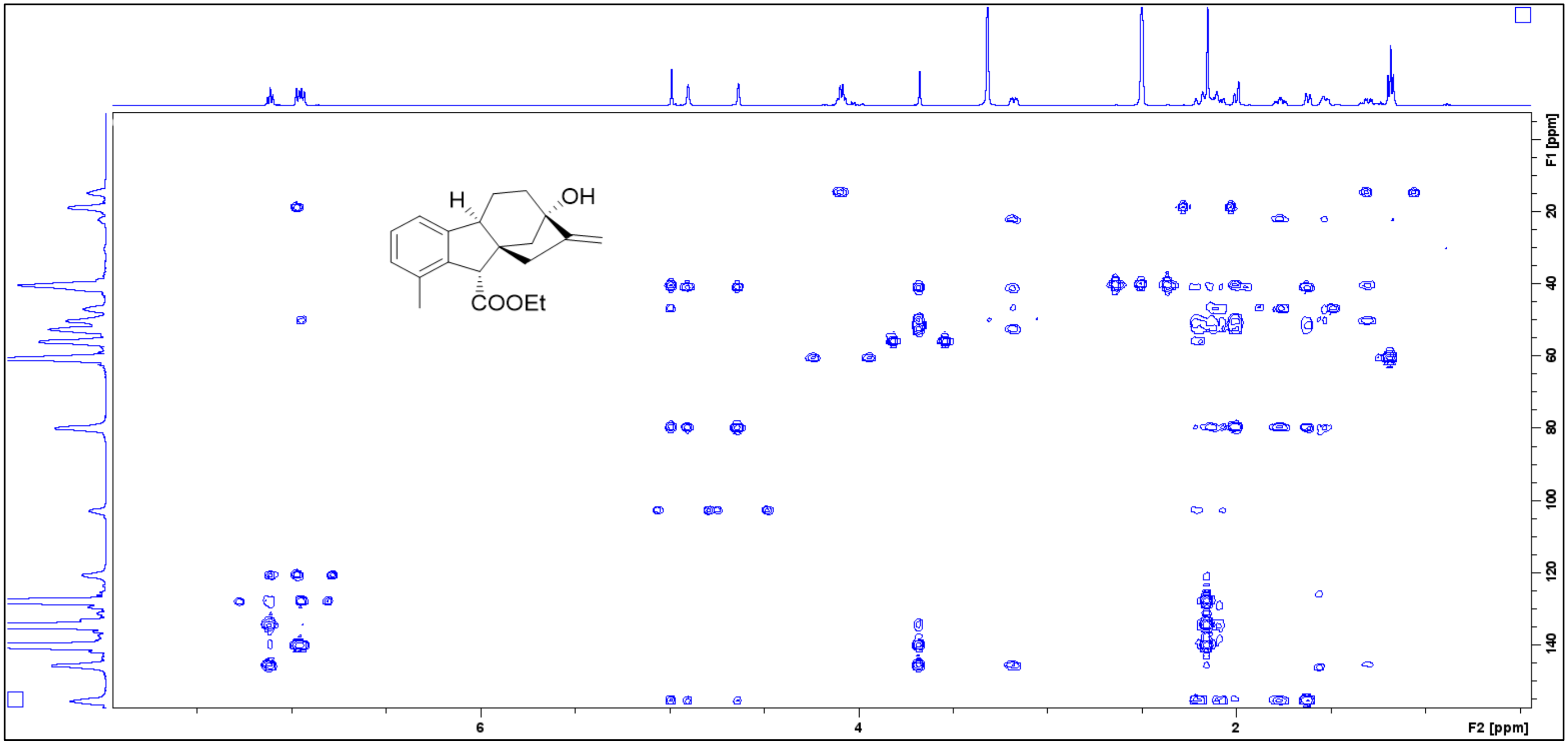

Figure S 82: ^{1}H-NMR of compound 34

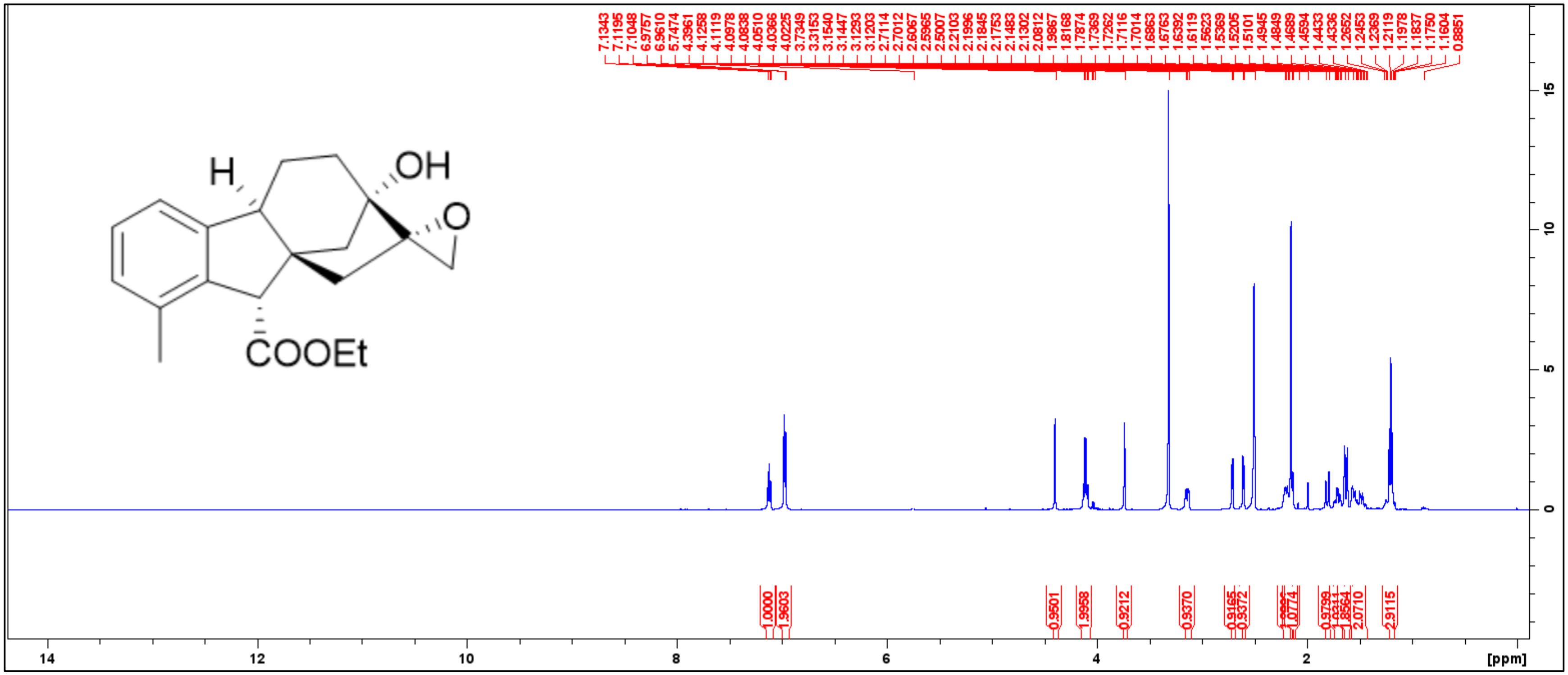

Figure S 83: ^{13}C-NMR (JMOD) of compound 34

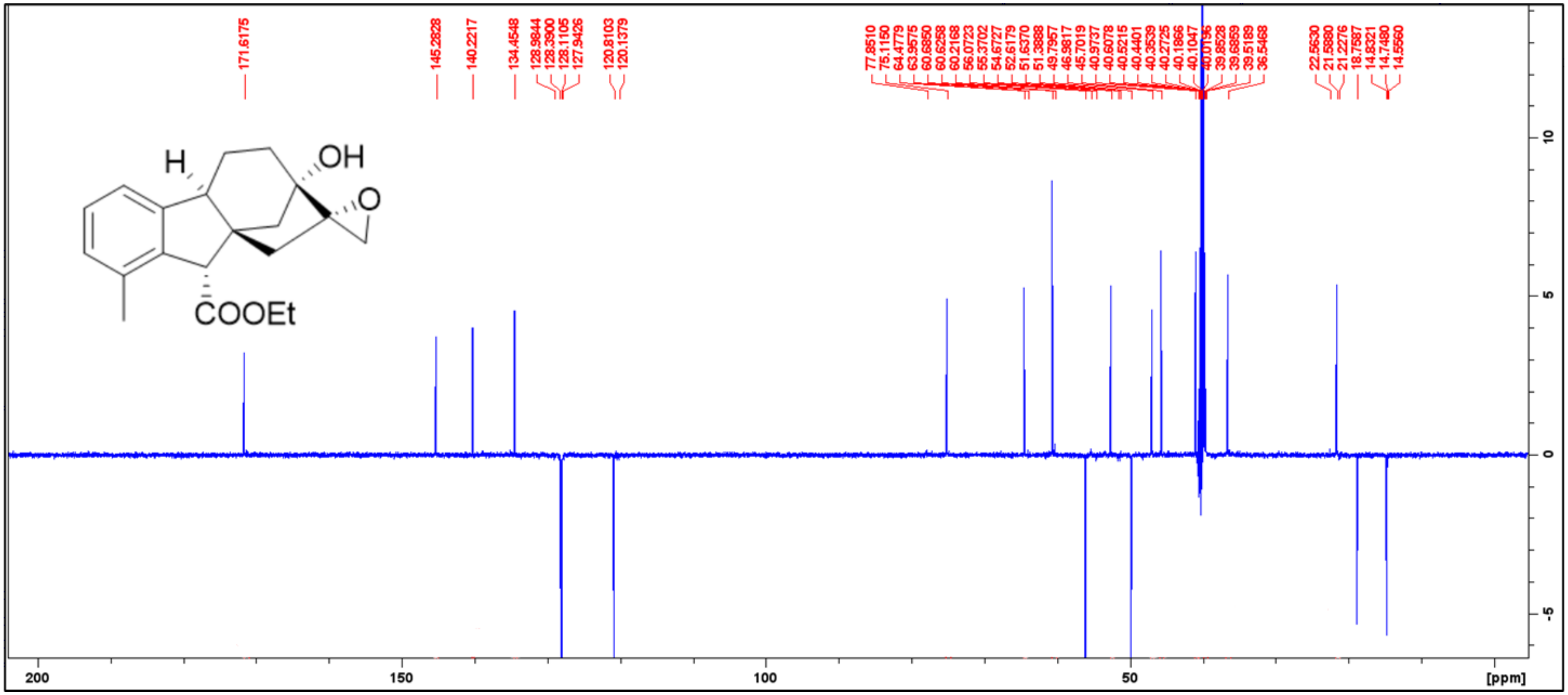

Figure S 84: ^{1}H-NMR of compound 35

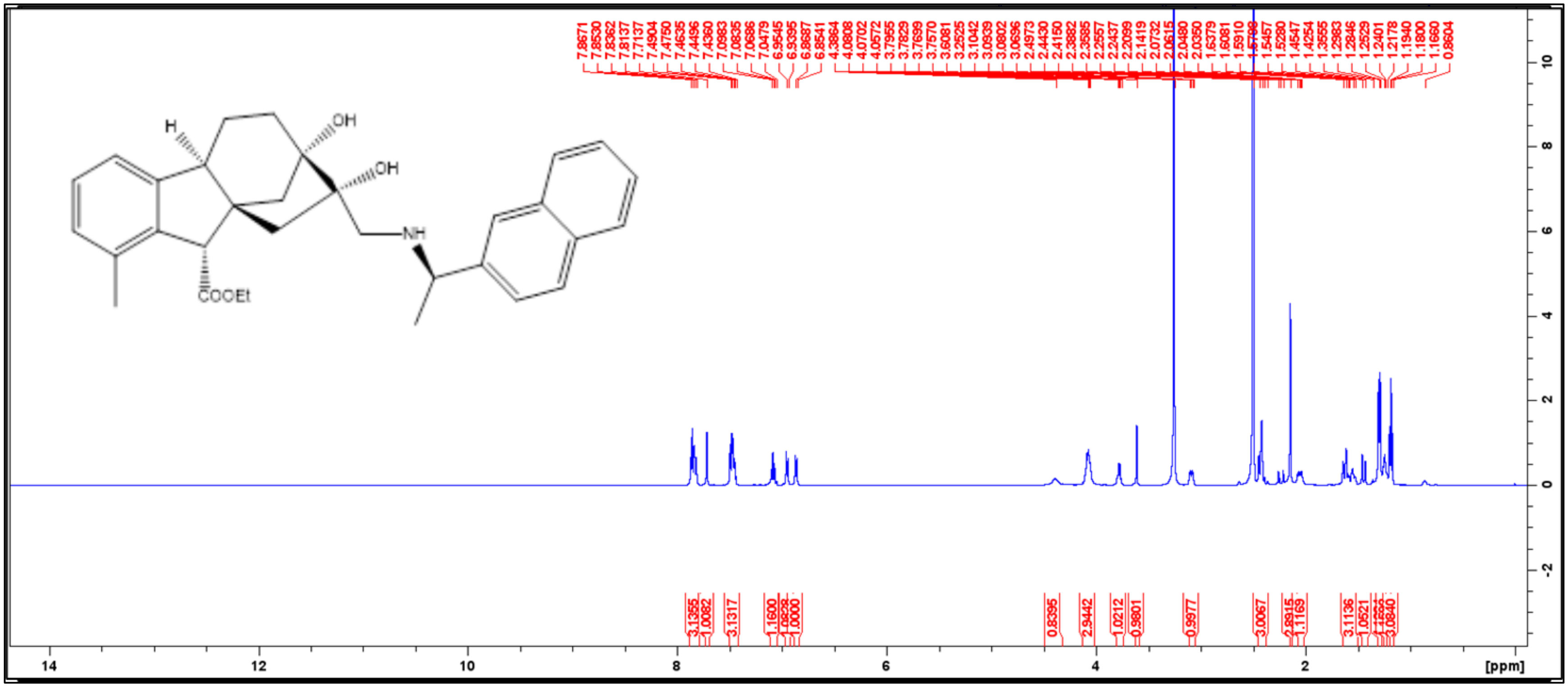

Figure S 85: ^{13}C-NMR (JMOD) of compound 35

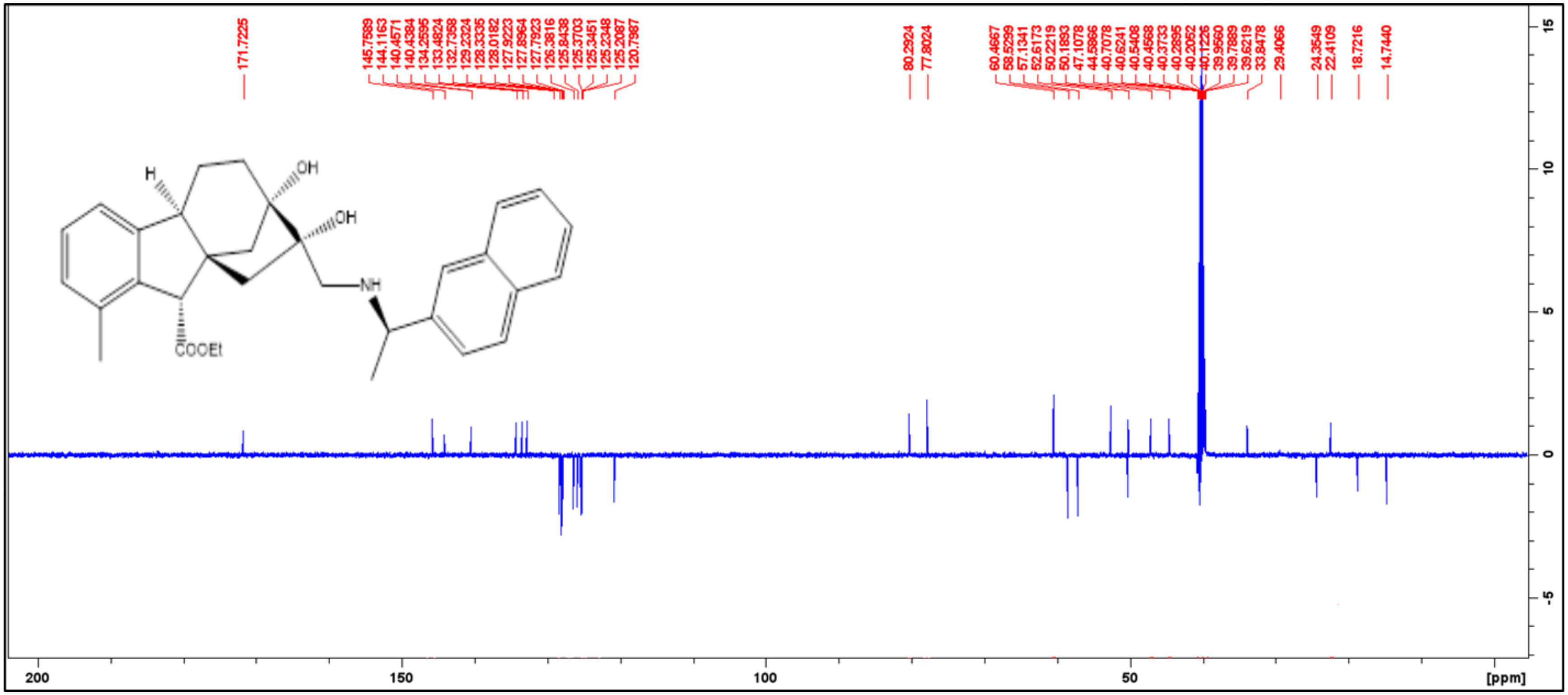

Figure S 86: ^{1}H-NMR of compound 36

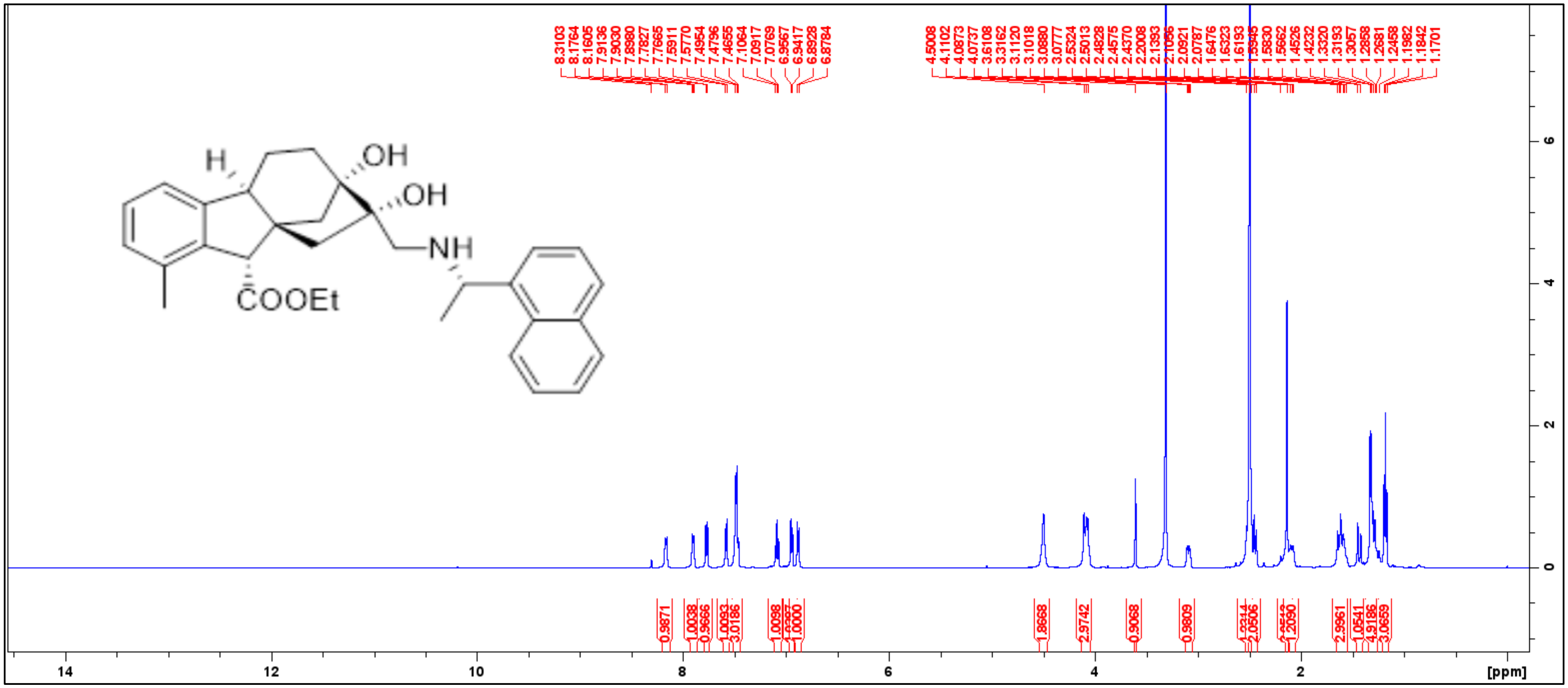

Figure S 87: ^{13}C-NMR (JMOD) of compound 36

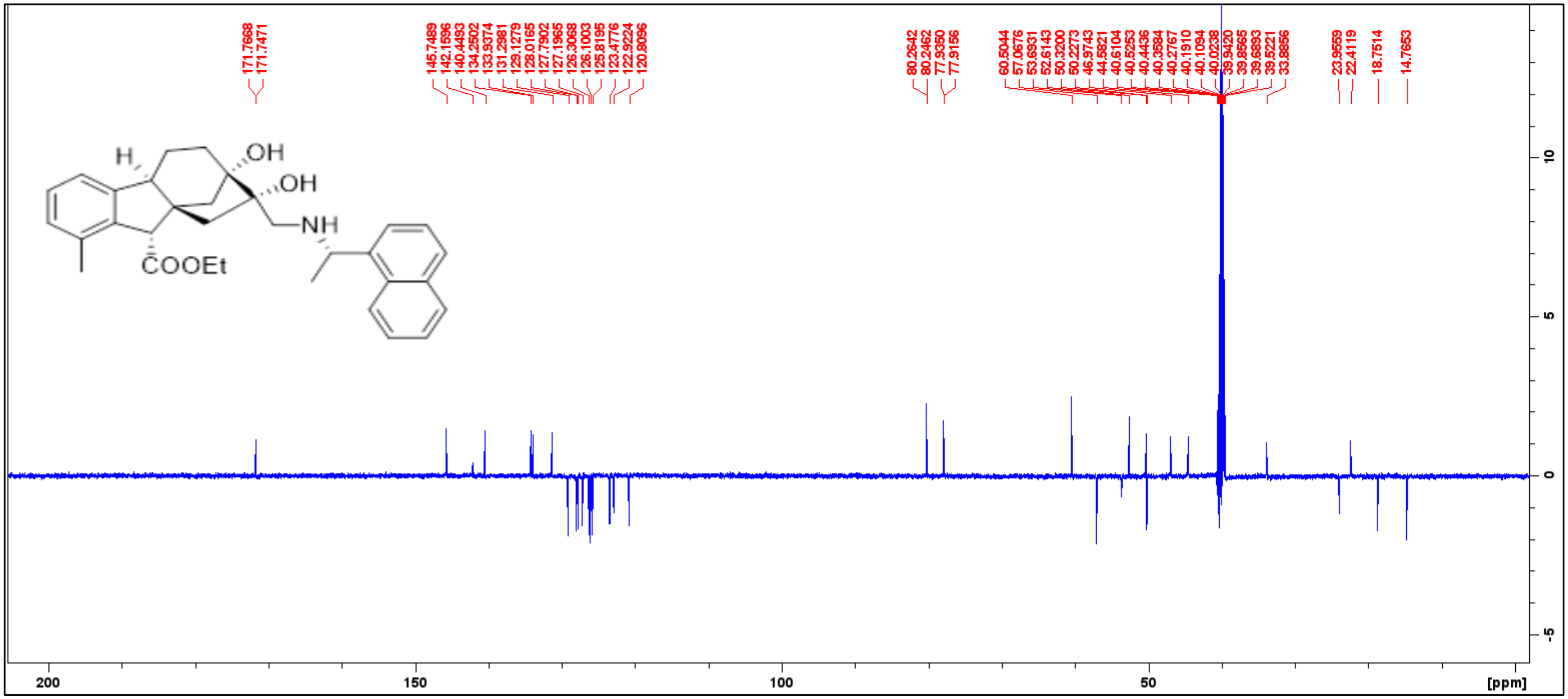

Figure S 88: ^{1}H-NMR of compound 37

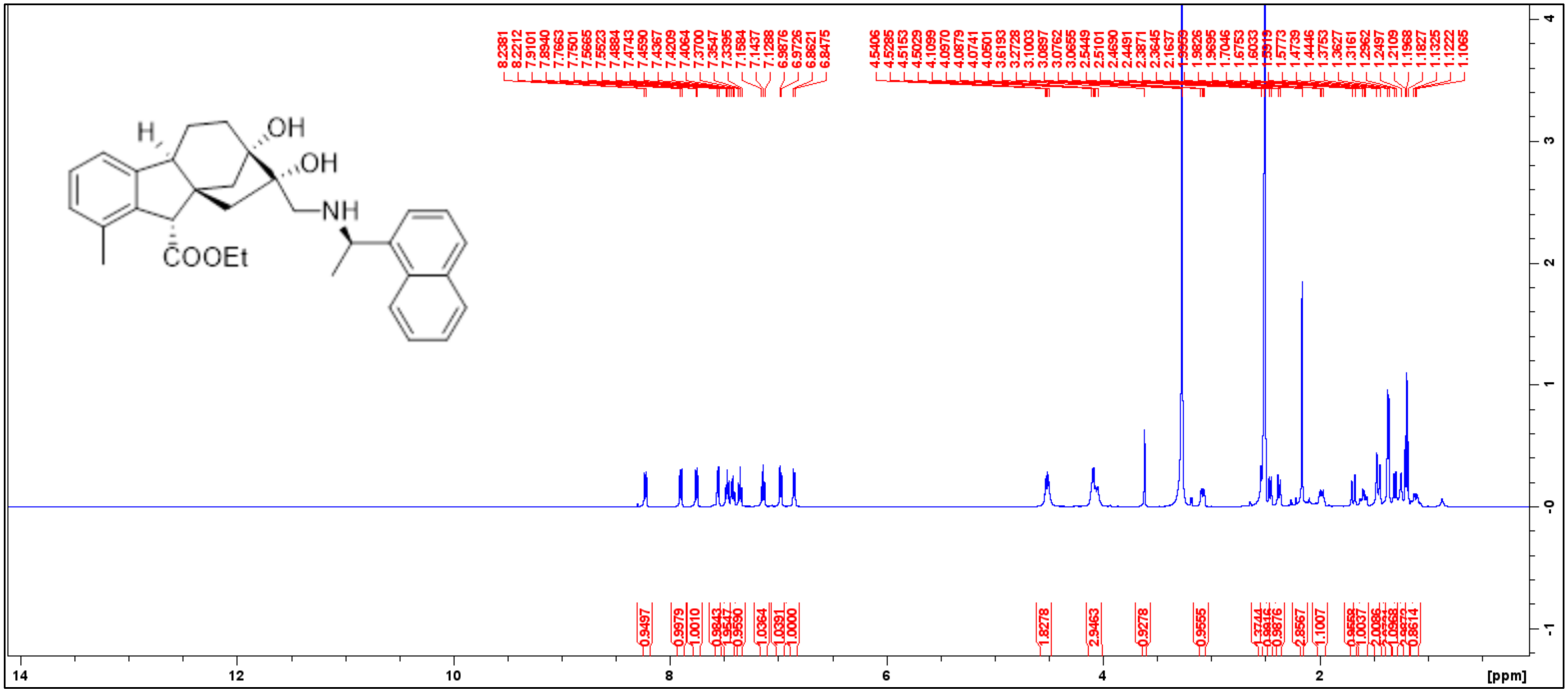

Figure S 89: ^{13}C-NMR (JMOD) of compound 37

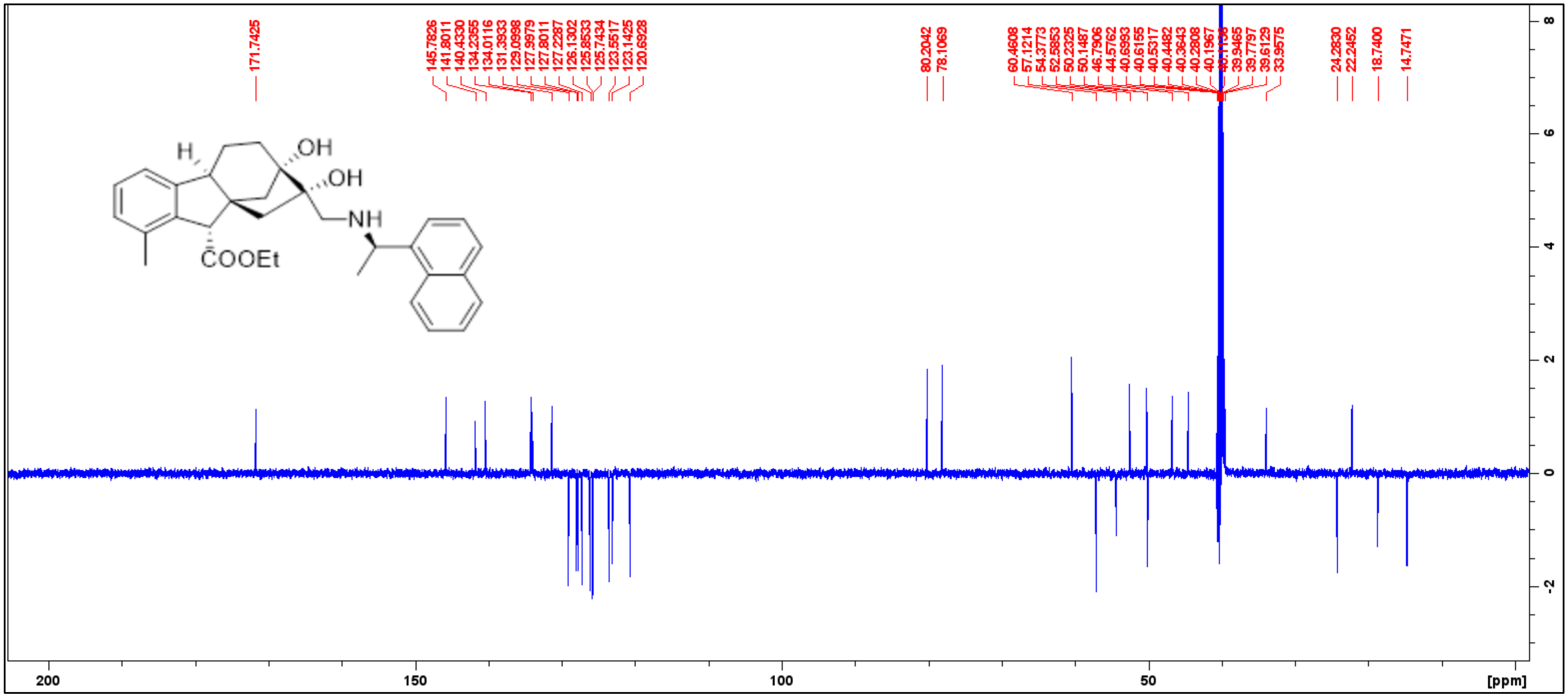

Figure S 90: ^{1}H-NMR of compound 38

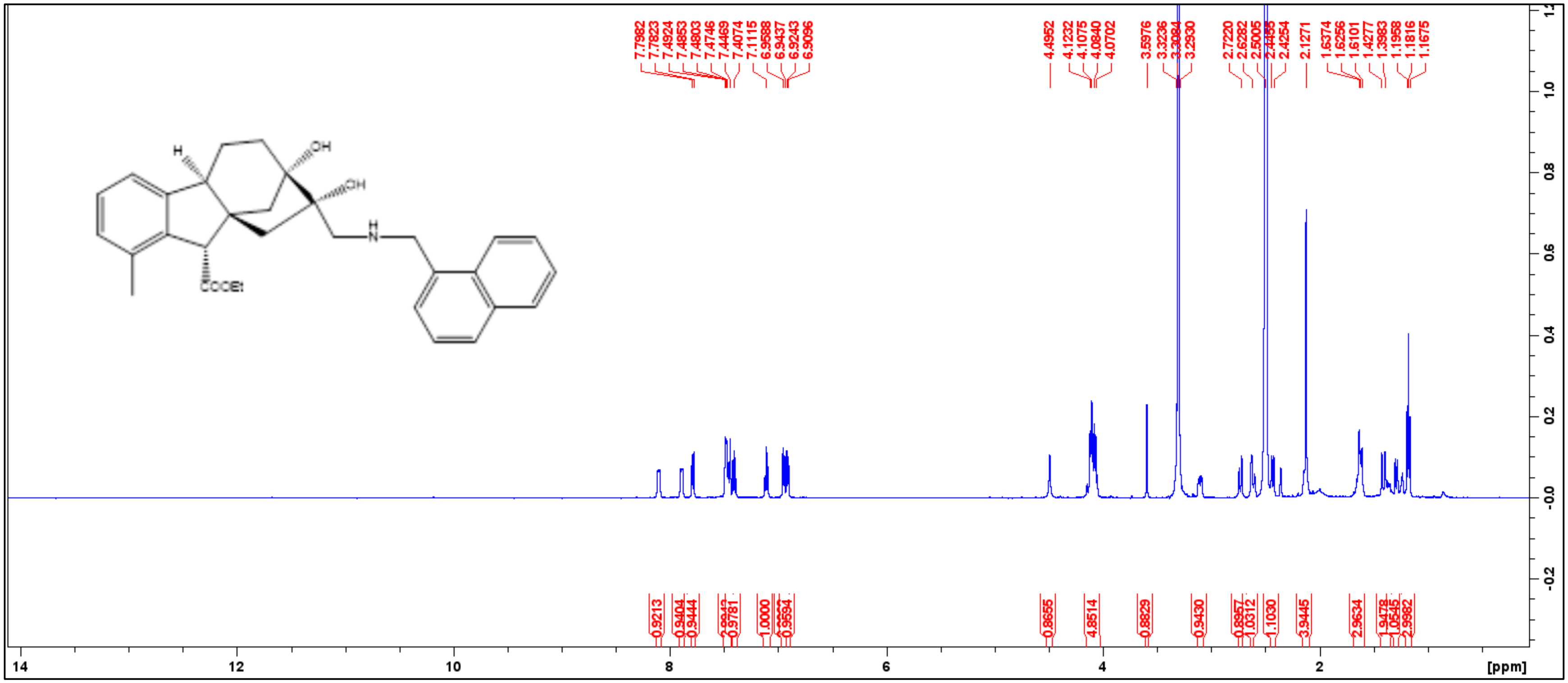

Figure S 91: ^{13}C-NMR (JMOD) of compound 38

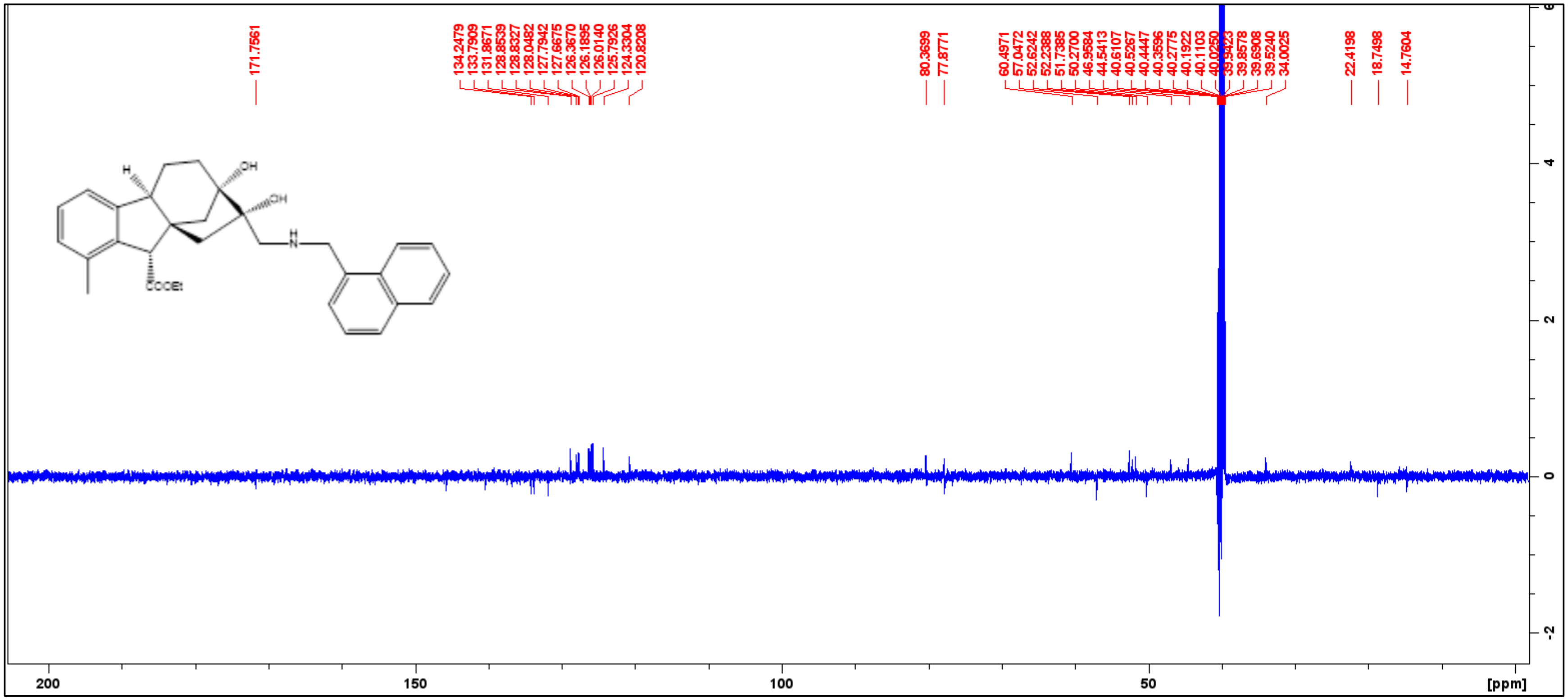

Figure S 92: NOESY of compound 38

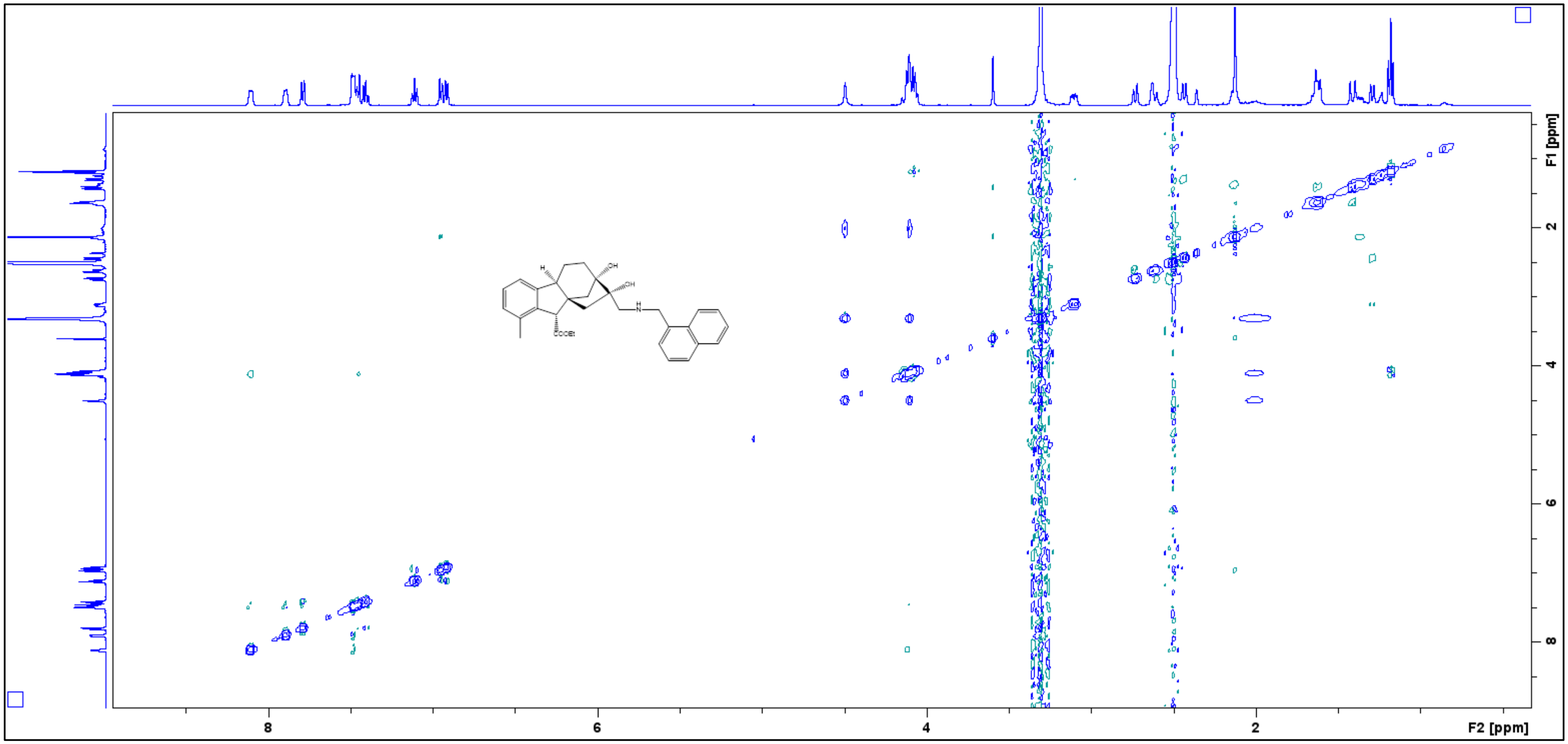

Figure S 93: COSY of compound 38

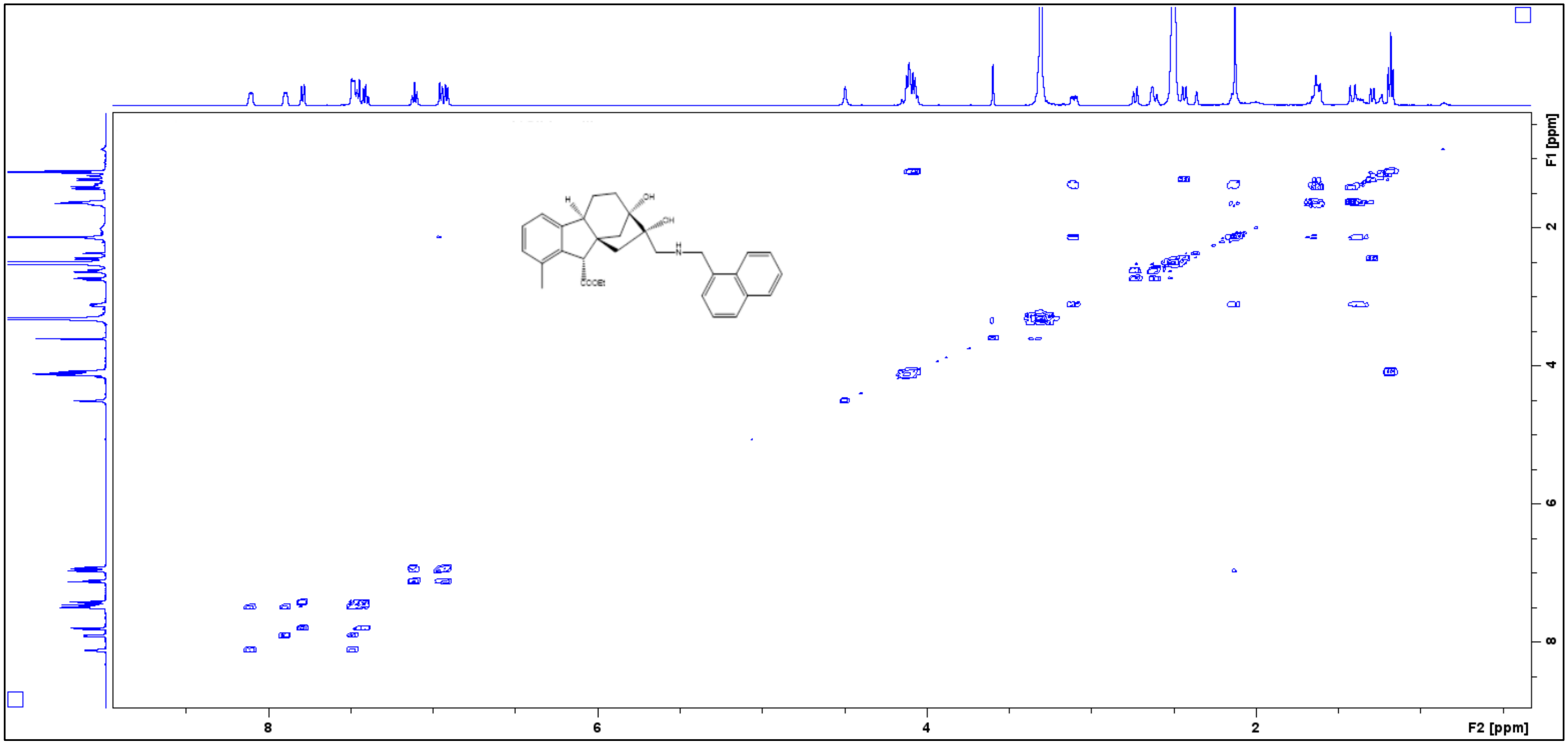

Figure S 94: HSQC of compound 38

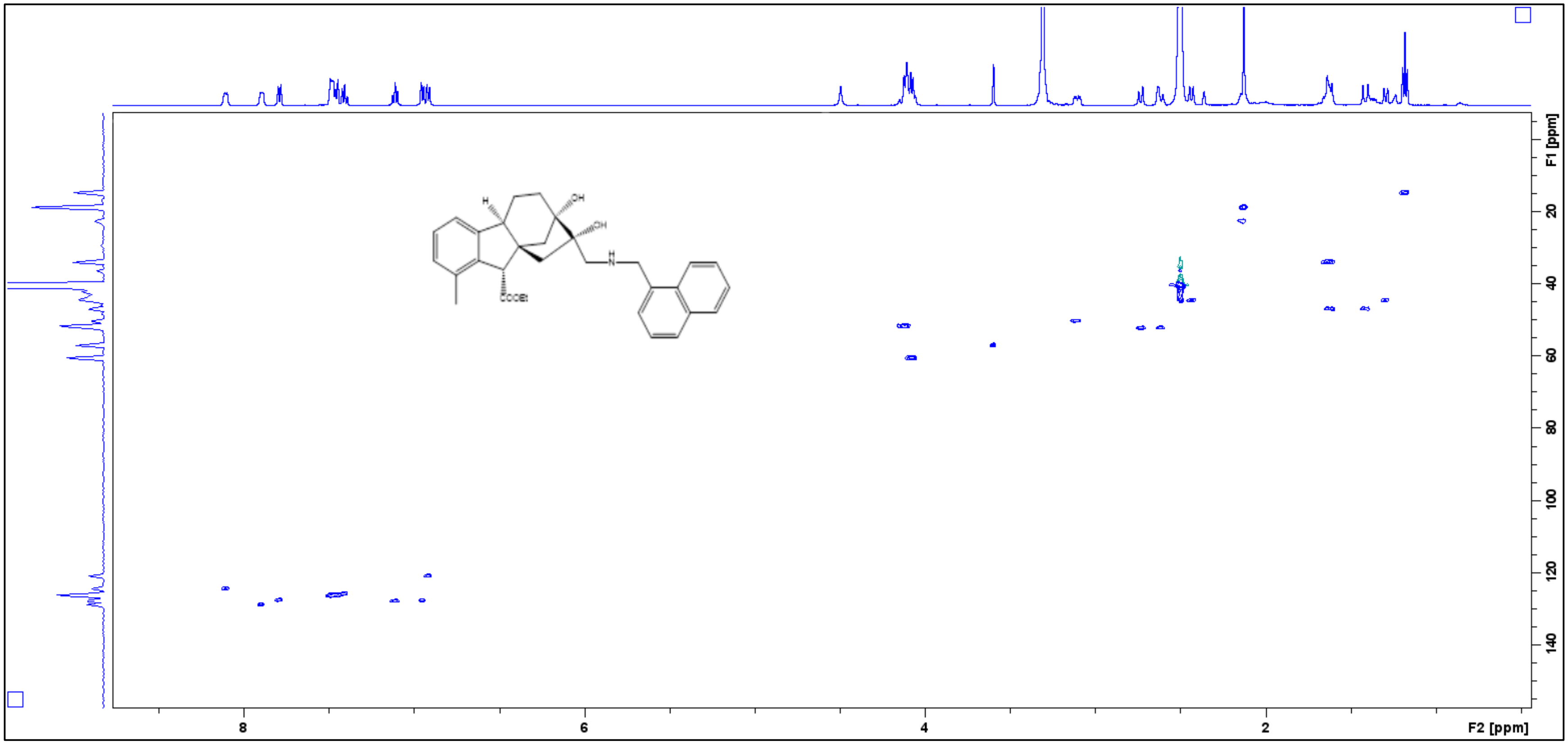

Figure S 95: HMBC of compound 38

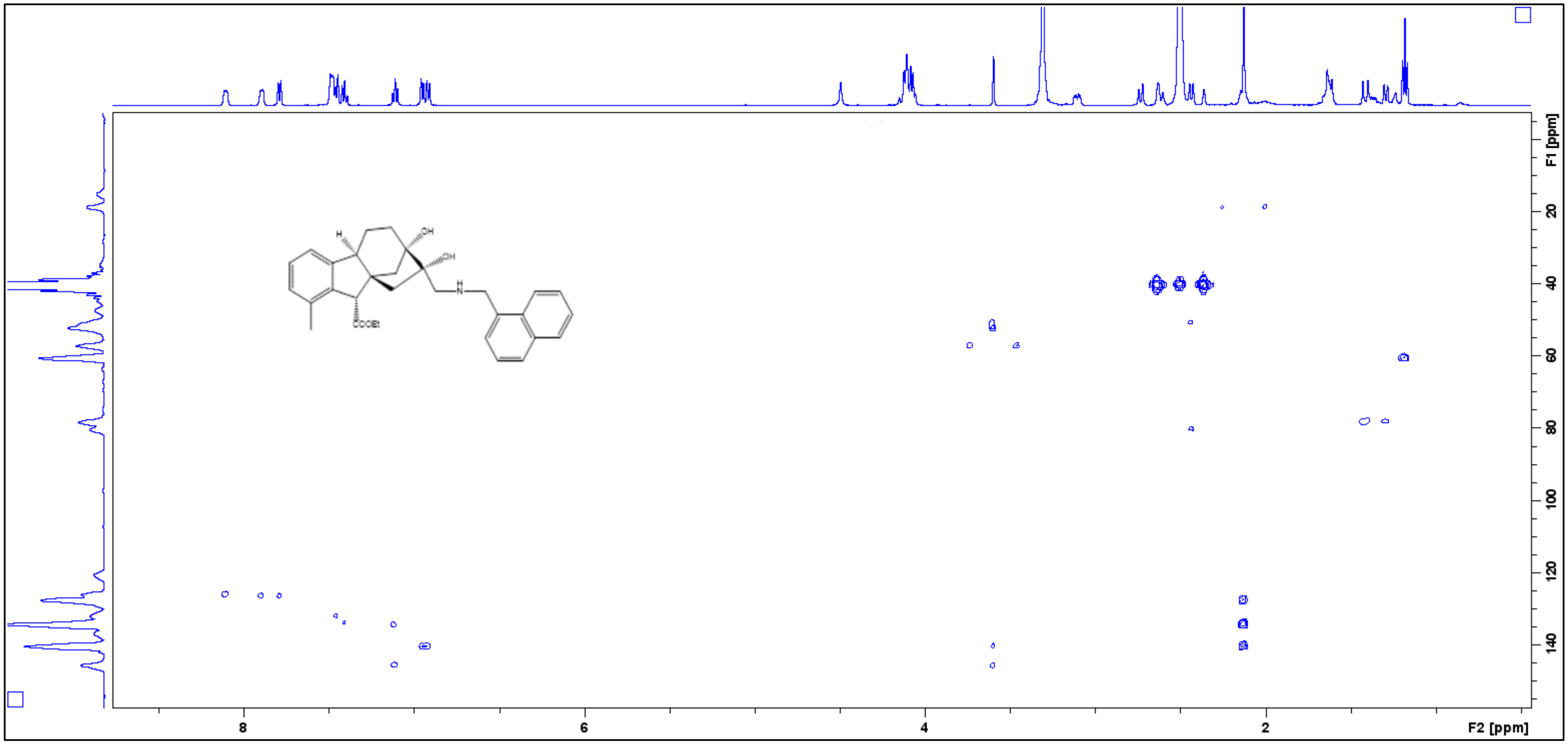

Table S2: CDocker energy values for compounds 12, 13 and 14

	Pim-1		AKT		MAP		RAFI		ALK	
	CDocker energy	No. interactions	CDocker energy	No. interactions	CDocker energy	No. interactions	CDocker energy	No. interactions	CDocker energy	No. interactions
12	-11.2234	1	-13.4305	1	-23.4897	0	-16.4574	0	-43.4401	8
13	-9.1276	0	-10.7763	1	-21.4457	0	-14.6789	1	-46.6684	5
14	-10.8743	0	-9.7745	0	-19.7651	0	-15.6872	0	-45.2367	5

Figure S 96: Plot of Polar Surface Area (PSA) vs. LogP for a standard and test set showing the 95% and 99% confidence limit ellipses corresponding to the Blood Brain Barrier and Intestinal Absorption models of compounds 12, 13 and 14

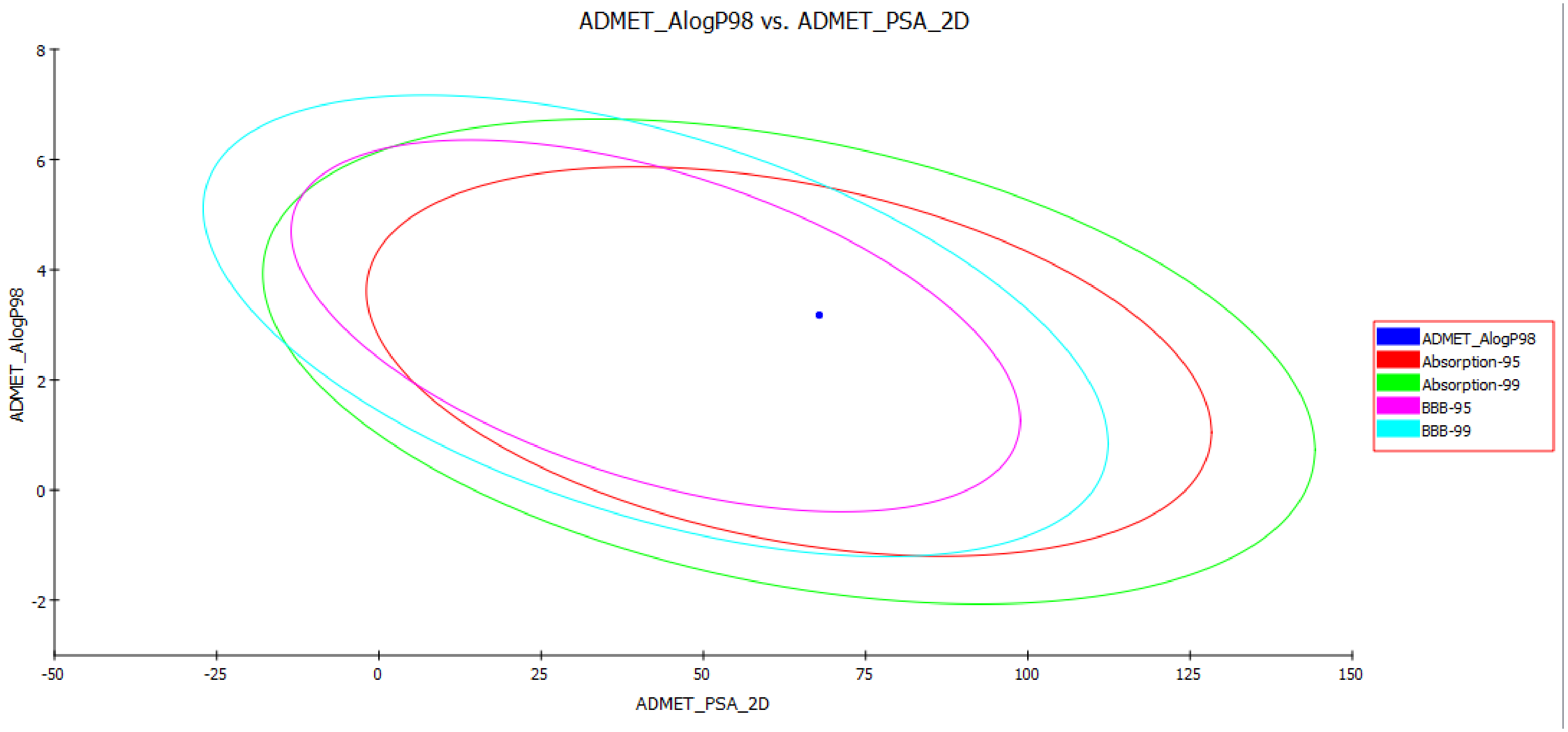

Table S3: *In silico* ADMET properties

Compounds	*In silico* ADMET properties				
	BBB level	Absorption level	Solubility level	CYP2D6 inhibition	PPB level
12	2 (medium)	0 (good)	2 (low)	0 (non-inhibitor)	1 (>90%)
13	2 (medium)	0 (good)	2 (low)	0 (non-inhibitor)	2 (>95%)
14	2 (medium)	0 (good)	2 (low)	0 (non-inhibitor)	2 (>95%)
